# Structure–function relationships in aryl diazirines reveal optimal design features to maximize C–H insertion†

**DOI:** 10.1039/d1sc03631a

**Published:** 2021-08-10

**Authors:** Stefania F. Musolino, Zhipeng Pei, Liting Bi, Gino A. DiLabio, Jeremy E. Wulff

**Affiliations:** Department of Chemistry, University of Victoria Victoria BC V8W-3V6 Canada wulff@uvic.ca; Department of Chemistry, University of British Columbia Kelowna BC V1V-1V7 Canada gino.dilabio@ubc.ca

## Abstract

Diazirine reagents allow for the ready generation of carbenes upon photochemical, thermal, or electrical stimulation. Because carbenes formed in this way can undergo rapid insertion into any nearby C–H, O–H or N–H bond, molecules that encode diazirine functions have emerged as privileged tools in applications ranging from biological target identification and proteomics through to polymer crosslinking and adhesion. Here we use a combination of experimental and computational methods to complete the first comprehensive survey of diazirine structure–function relationships, with a particular focus on thermal activation methods. We reveal a striking ability to vary the activation energy and activation temperature of aryl diazirines through the rational manipulation of electronic properties. Significantly, we show that electron-rich diazirines have greatly enhanced efficacy toward C–H insertion, under both thermal and photochemical activation conditions. We expect these results to lead to significant improvements in diazirine-based chemical probes and polymer crosslinkers.

## Abstract 2

Electron-rich aryl diazirines have lower activation temperatures and a longer *λ*_max_ than electron-poor analogues, and undergo C–H insertion up to ten-fold more efficiently—suggesting improved performance for biological probes and polymer crosslinkers.

## Graphical abstract

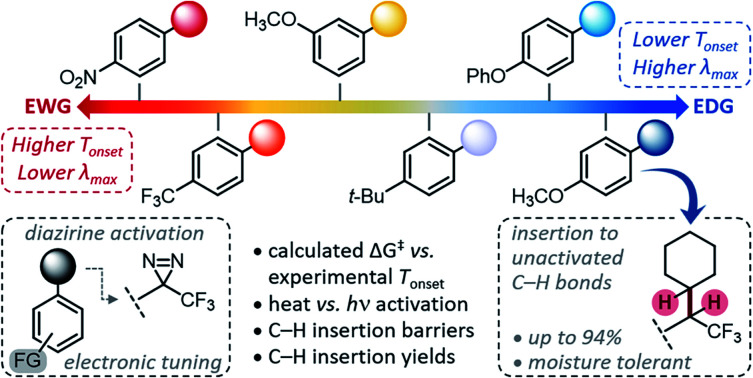

## Introduction

The insertion of high-energy carbene or nitrene species into unactivated C–H, O–H and N–H bonds constitutes a powerful strategy for forming new bonds in complex biological, chemical, or mechanical mixtures.^1,2^ The ability to rapidly and controllably generate carbenes from stable and readily accessed precursor molecules allows one to label (or ‘tag’) binding partners,^3^ isolate reaction products,^4^ or crosslink polymer materials.^5–7^

**Fig. 1:**
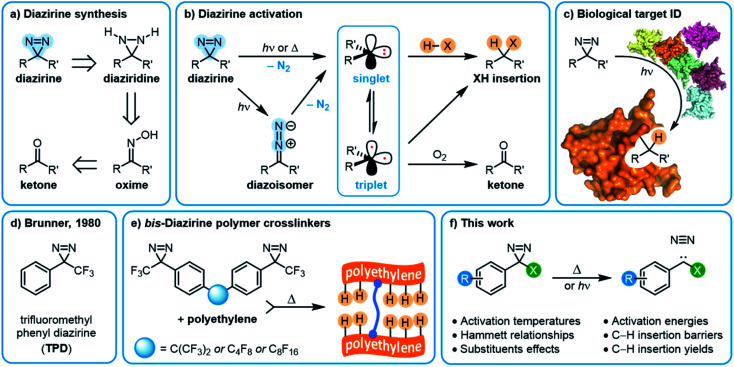
Overview of diazirine synthesis, activation, and applications. (a) Synthetic strategies for producing aryl diazirines. (b) Diazirine activation and carbene formation. (c) The use of diazirines for biological target identification. (d) Trifluoromethyl phenyl diazirine (TPD). (e) bis-Diazirine polymer crosslinkers based upon the TPD monomeric structure. (f) This work: structure–function relationships within trifluoromethyl aryl diazirines.

In recent years, diazirine motifs have enjoyed enormous popularity as carbene precursors, for applications ranging from biological target identification through to adhesion of commodity plastics (Fig. 1). Part of this popularity can be attributed to their small size and ready synthetic access.^8,9^ Of course, for any reactive reporter group the most important criteria are that the reporter be stable under any required synthesis, handling, and experimental conditions, yet be easy to activate (ideally with good control over spatial localization) when needed. Diazirines excel when measured against these criteria as well; most diazirines are stable to commonly used synthetic reaction conditions (including strong Brønsted^5^ and Lewis^10,11^ acids), and many diazirine-containing molecules are thermally stable. When desired, diazirines can be readily activated using light (*ca.* 350–365 nm),^8^ heat (typically 110–130 °C),^5,12^ resonance energy transfer^13^ or electrical potential,^14^ resulting in the expulsion of nitrogen gas and the formation of the corresponding carbene. The long wavelength of light required for photochemical activation (outside the window where biological and polymer materials absorb) and the low temperature required for thermal activation (below the melting temperature of many commodity polymers) are critical factors that have contributed to the broad uptake of diazirines by research groups around the world.

The substituents on either side of the CN_2_ core are known to influence the properties of both the parent diazirine molecule and the product carbene. For example, dialkyl diazirines generally give rise to ground-state singlet carbenes, due to hyperconjugative donation of filled C–H orbitals into the unoccupied p orbital of the singlet carbene group.^15,16^ Carbenes generated from phenyl diazirines (or from the isomeric linear diazo compound) can exist in either singlet or triplet forms; the triplet is generally found to be the ground state for these species,^17^ but in many cases the singlet carbene is believed to be produced initially,^18,19^ after which intersystem crossing (ISC) facilitates relaxation to the observed triplet (Fig. 1b).^17^ The energy gap between the singlet and triplet states is crucially important, since the two types of carbenes undergo different types of reactions with X–H bonds. Triplet reactivity is dominated by stepwise hydrogen abstraction/radical recombination pathways that can lead to undesirable side reactions, while singlet X–H insertion is believed to proceed by a concerted—but two-phase—pathway wherein the empty carbene p orbital first receives electron donation from the X–H σ bonding orbital, and then the lone pair on the carbene back-donates into the X–H σ* orbital.^16^ Further complicating matters, triplet carbenes are known to undergo rapid reactions with adventitious O_2_ (ultimately leading to the production of ketone byproducts) while singlet carbenes appear to be immune to this undesirable reaction pathway.^20^

As is the case with nitrene species, diazirines and carbenes can undergo unwanted rearrangement reactions; Brunner showed in 1980 that the installation of an α-trifluoromethyl group reduced the likelihood of such rearrangements, leading to improved overall stability and more controllable reactivity.^8^

Since Brunner's seminal report, the trifluoromethyl phenyl diazirine group (TPD; Fig. 1d) has become the most widely employed carbene precursor in the chemical literature. Trifluoromethyl aryl diazirines are now used in a host of biological target identification experiments (Fig. 1c)^3,21–25^ where they are found to outperform alternative labeling groups like nitrenes or benzophenones,^26,27^ and the TPD motif has been genetically encoded into protein structures using expanded genome techniques.^28^ TPD has also been used to map the localized protein environment on T cells using an innovative μ-mapping technique facilitated by Dexter energy transfer,^13^ and trifluoromethyl aryl diazirines have been designed into bio-adhesives to expedite wound closure.^14^ In polymer science, trifluoromethyl aryl diazirines have been conjugated to fluorophores and photosensitizers in order to imbue nylon^29^ and spunbond polypropylene^30^ materials with new functionality. Silicon and silicon nitride surfaces can be similarly functionalized.^31^ TPD groups have also been used on surfaces to capture small molecule reaction products^4^ and drug molecules,^32^ and have been incorporated into polymer crosslinkers that can be used to strengthen woven polyethylene fabric^5^ or provide adhesion between polypropylene or polyethylene surfaces (Fig. 1e).^12^ These latter applications using commodity aliphatic polymers provide a particularly stringent test of a diazirine's ability to support C–H insertion, since (to a first approximation, at least) such polymers only contain C–C and C–H bonds, and therefore lack anything that would be traditionally viewed as a reactive functional group.

Given the extensive utility of TPD groups, it is surprising that linear free energy relationships in trifluoromethyl aryl diazirines have received very little attention. (By contrast, Hammett relationships in α-chloro diazirines have been extensively studied, but these species are known to participate in a variety of reactions that are not available to the much more important α-trifluoromethyl congeners.^33^) As a result, incorporation of a trifluoromethyl aryl diazirine group into a molecule of interest (whether for biological or materials science applications) has typically been done in an ad hoc manner, without any consideration of how the electronics of the aryl diazirine group will influence the generation and reactivity of the resulting carbene. While a variety of electron-rich, electron-poor, and electron-neutral trifluoromethyl aryl diazirines have been described in the literature, only in very rare cases has the influence of electron-donating or electron-withdrawing groups on reaction outcome (especially under thermal activation conditions) been discussed, and the conclusions from these reports are somewhat contradictory.

In 2011, Song and Sheridan reported a study of *meta*-methoxy *vs. para*-methoxyphenyl trifluoromethyl diazirine, and showed that the *para*-methoxy substituent was capable of supporting a singlet carbene ground state (at 7 K in a frozen nitrogen matrix) due to resonance stabilization.^20^ Two years later, Raimer and Lindel studied this same *para*-methoxy trifluoromethyl diazirine molecule, and found that activation in the presence of phenols (including tyrosine derivatives) led to production of the desired singlet carbene, which could then undergo formal O–H and C–H insertions with the substrate.^34^ Interestingly, however, these reactions were shown in all cases to be the result of initial protonation of the singlet carbene to generate a benzylic cation, which could then either be quenched by reaction with the phenol (or phenoxide anion) or else undergo Friedel–Crafts reaction with the electron-rich aromatic ring.^34^ Various lines of evidence were used to rule out the existence of any direct C–H insertion reactions. While the Sheridan and Lindel papers provide important precedent in favour of the hypothesis that electron-rich trifluoromethyl aryl diazirines might provide an improved reactivity profile relative to electron-poor or electron-neutral analogues, neither report demonstrated direct C–H insertion reactions with aliphatic substrates. As a result, it remains an open question whether electron-rich aryl diazirines would be preferred or not, in the applications described above.

To further complicate matters, a recent report by Kumar, Tipton and Manetsch provided compelling evidence that electron-poor diazirines are superior, due to improved stability under ambient light, and that this lack of electron density does not impair the molecules' ability to undergo C–H insertion.^35^ Meanwhile, Kanaoka and co-workers have advocated for the use of diazirines bearing *both* an electron-donating alkoxy group *and* an electron-withdrawing nitro substituent, due to improved optical properties.^36^ While the Kanaoka group showed the performance of their molecules in reacting with cyclohexane (an important benchmarking experiment for diazirine reactivity) they did not compare the efficacy of their structures to that of the parent TPD molecule.

These sparse reports leave the experimentalist who might want to employ a diazirine for a particular application in a quandary. Should an electron-rich or electron-poor trifluoromethyl aryl diazirine be used if one wants to maximize the potential for C–H insertion under standard laboratory conditions? If an electron-rich system is preferred in order to stabilize a singlet carbene intermediate, how electron rich does it need to be? Given that the optimized electronic properties will likely increase the synthetic challenge in preparing the molecule, is the improved performance sufficient to justify the increased synthetic effort and/or increased cost of reagents?

The situation becomes even more complex when activation temperature is taken into account. The few structure–function studies on trifluoromethyl aryl diazirines alluded to above all focused on photochemical activation, since this is the dominant excitation method used for biological studies. For materials science work, however, thermal activation is generally preferred;^5,12^ while commodity polymers like polyethylene and polypropylene do not *absorb* light in the window used for photochemical diazirine activation, they do very efficiently *scatter* light and are therefore visibly opaque or white solids—making the use of photochemical activation techniques a substantial challenge for real-world samples. An understanding of how activation temperature changes with diazirine electronics is therefore critically important to the design of new polymer crosslinkers and adhesives.^37^ Moreover, the thermal activation temperature dictates the explosivity (or lack thereof) for the diazirine molecule being used:^5,38^ an understanding of how activation temperature can be tuned is therefore necessary for designing safe and effective diazirine-based reagents for all manner of applications.

In this work (Fig. 1f) we use a combination of experimental and computational methods to conduct an extensive structure–function study of the properties and reactivity of aryl diazirines—especially those bearing an α-trifluoromethyl substituent. We particularly focus our experimental work on the measurement of activation temperatures by differential scanning calorimetry (DSC), and also explicitly compare the ability of electron-rich, electron-neutral, and electron-poor molecules to engage in C–H insertion reactions under both thermal and photochemical activation conditions. Ultimately we anticipate that the conclusions from this work will inform the design of molecular probes, small-molecule capture agents, and polymer crosslinkers with improved efficacy.

## Results and discussion

### Tunability of optical absorbance characteristics

We synthesized (or, in a few cases, purchased) several representative trifluoromethyl aryl diazirines, incorporating electron-rich, electron-neutral, and electron-withdrawing groups on the aromatic ring (see Table 1 for substituents).

**Table 1:** Effect of aryl substituent electronics on activation temperature and on activation free energy

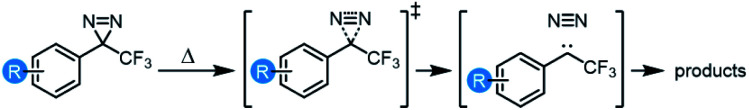				
Entry	R	*T*_onset_^a^ (°C)	*T*_peak_^b^ (°C)	Δ*G*^‡^^c^ (kJ mol^−1^)
1	4-OCH_3_	88.0 ± 0.5	113.0 ± 0.7	141.6
2	4-OPh	90.2 ± 0.2	116.6 ± 0.1	140.3
3	4-*t*-Bu	100.0 ± 0.1	125.5 ± 1.5	147.0
4	H	103.3 ± 0.5	127.4 ± 0.7	149.8
5	4-Br	105.6 ± 0.5	132.4 ± 0.6	147.8
6	4-CH_2_OH	106.8 ± 0.2	132.2 ± 0.3	149.1
7	4-CH_2_Br	102.0 ± 0.2	133.0 ± 0.2	148.1
8	3-OCH_3_	108.7 ± 0.4	135.5 ± 0.1	152.5
9	3-OH	110.5 ± 0.2	135.8 ± 0.1	152.1
10	3,5-OCH_3_	112.5 ± 0.1	137.9 ± 0.1	149.3
11	4-CF_3_	115.7 ± 0.4	138.9 ± 0.6	150.7
12	4-CHO	113.2 ± 0.5	139.1 ± 0.3	153.2
13	4-NO_2_	117.6 ± 1.0	143.9 ± 0.8	154.3

a Experimentally determined by extrapolation of the tangent of the upward slope observed in the DSC experiment, to the fitted baseline of the plot. Data are presented as the average of three independent measurements ± standard deviation.

b Experimentally determined from the peak maxima in the DSC experiment. Data are presented as the average of three independent measurements ± standard deviation.

c Calculated free energy (298.15 K, 1 atm) of activation (M06-2X-D3/6-31+G(d,p)//M06-2X-D3/6-31G(d,p)) associated with conversion of the diazirine into the corresponding carbene in vacuum.

**Fig. 2:**
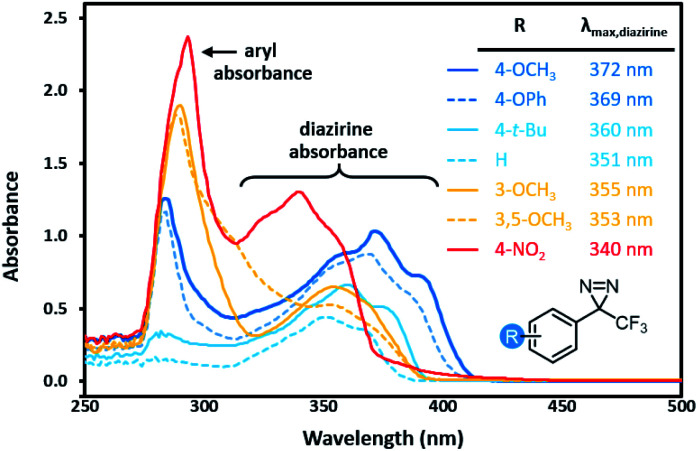
Variation in UV/Vis absorption spectra for representative trifluoromethyl aryl diazirines. All data were acquired using 5 mM samples in *n*-hexane. Refer to Fig. S1† for comparative spectra obtained in methanol.

Characterization of archetypal electron-rich (4-OMe-C_6_H_4_, 4-OPh-C_6_H_4_), electron-neutral (C_6_H_5_, 4-*t*-Bu-C_6_H_4_), and electron-poor (3-OMe-C_6_H_4_, 3,5-(OMe)_2_-C_6_H_3_, 4-NO_2_-C_6_H_4_) trifluoromethyl aryl diazirines by UV/Vis spectroscopy revealed interesting trends. As shown in Fig. 2, electron-rich and highly electron-poor diazirines both showed strong absorbances in the diazirine activation window of *ca.* 320–410 nm. However, electron-neutral and moderately electron-poor trifluoromethyl aryl diazirines had attenuated absorbances in this range. It would seem, therefore, that strongly electron-rich or electron-poor diazirines might be preferred for photochemical activation, simply on the basis of their improved extinction coefficients. This is an important observation, given that the vast majority of biological target identification studies in which diazirines have been used have featured electron-neutral substrates.

Of equal importance, we observed a >30 nm shift to higher wavelengths when moving from electron-poor to electron-rich trifluoromethyl aryl diazirines. The ability to shift the diazirine activation bands to longer wavelength is useful both in terms of evading background absorbance and allowing the use of cheaper and more readily purchased light sources.

### Tunability of activation temperature and activation free energy

Each of the trifluoromethyl aryl diazirine molecules was analyzed by DSC (refer to the ESI† for representative traces) to measure the onset temperature for diazirine activation (*T*_onset_)^39^ and the peak temperature (*T*_peak_; the temperature at which the diazirine activation exotherm reaches maximal heat flow). At the same time, we computationally determined the activation free energy (Δ*G*^‡^) for the diazirine activation reaction.

**Fig. 3:**
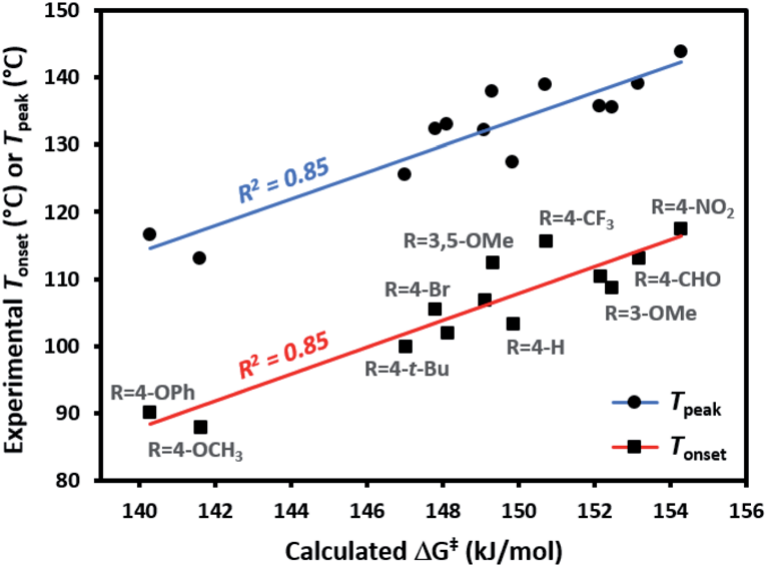
Comparison of experimentally determined *T*_onset_ and *T*_peak_ (°C) with calculated activation free energies (kJ mol^−1^) for the conversion of representative trifluoromethyl aryl diazirines into the corresponding carbene.

The data (Table 1) revealed a difference of *ca.* 30 °C in thermal activation temperatures, as we moved from electron-rich trifluoromethyl aryl diazirines (*e.g.* R = 4-OCH_3_; *T*_onset_ = 88 °C; *T*_peak_ = 113 °C) to electron-poor analogues (*e.g.* R = 4-NO_2_; *T*_onset_ = 118 °C; *T*_peak_ = 144 °C). This substantial difference in activation temperature corresponded to an equally significant difference in calculated activation free energy (ΔΔ*G*^‡^ > 12 kJ mol^−1^). Indeed, a strong positive correlation between all of the experimentally determined *T*_onset_ and *T*_peak_ values and the corresponding calculated activation free energies was observed (Fig. 3). This agreement between theory and experiment, confirming that *T*_onset_ is a reflection of the reaction barrier, is a necessary prerequisite for using computational methods to interrogate features of the reaction that cannot be easily observed experimentally.

The 30 °C span in activation temperatures is important from the perspective of experimental planning. For some applications where increased thermal stability is required (*e.g.* application of diazirine crosslinkers to polymer melts, or certain biological experiments where size limitations on a molecule may preclude the addition of molecular weight necessary to mitigate the explosivity risk that would otherwise accompany a low activation temperature) electron-poor diazirines with high activation temperatures will be preferred. For many applications, however (especially for polymer adhesion applications where one does not wish to melt the underlying polymer substrate), lower activation temperatures are highly desirable. In this context, the observation that electron-rich trifluoromethyl aryl diazirines can be activated at <90 °C is particularly relevant to the development of improved reagents for materials science.

### Quantification of linear free-energy relationships

The observed variation in activation temperatures can also shed light onto the mechanism of diazirine activation. To explore this, we plotted the data from Table 1 against various empirically derived Hammett *σ* values available from prior studies of reactions that are known to occur by polar or radical mechanisms. These included: (1) Hammett's original *σ* parameters derived from the study of *para*-substituted benzoic acid ionization;^40^ (2) Brown's *σ*^+^_p_ parameters derived from observation of solvolysis of substituted *t*-cumyl chlorides;^41^ (3) Arnold's 
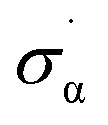
 parameters derived from the study of EPR hyperfine coupling of benzyl radicals;^42^ (4) Creary's 
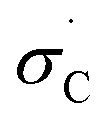
 parameters derived from the rearrangement of methylenecyclopropane ring systems;^43^ and (5) Jiang and Ji's 
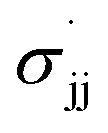
 parameters derived from the cyclodimerization of trifluorostyrenes.^44^ In comparisons of this type, a strong positive correlation to *σ*^+^ is generally taken as evidence in favour of some degree of carbocation character in the transition state, while correlation with any of the various *σ*˙ parameters is taken as evidence of radical character.^45^

**Fig. 4:**
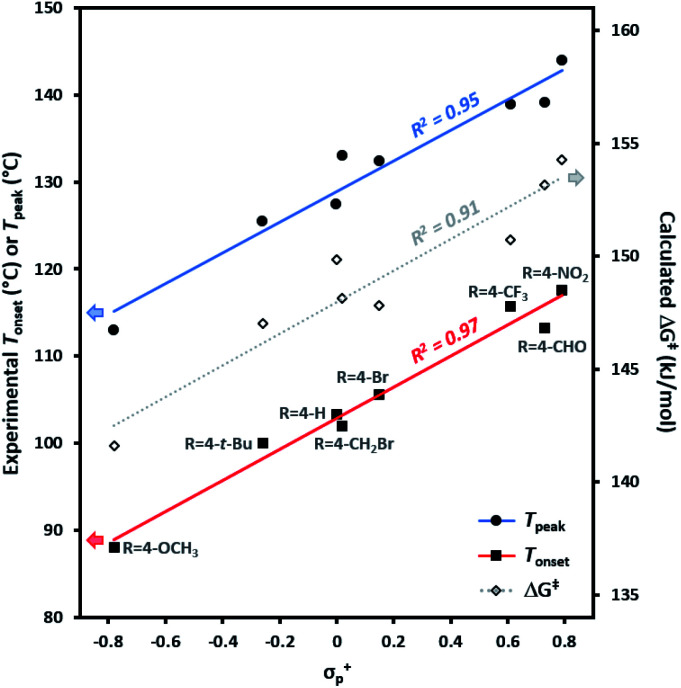
Variation in trifluoromethyl aryl diazirine activation temperatures and energies with the Hammett parameter *σ*^+^_p_. *T*_onset_, *T*_peak_, and Δ*G*^‡^ are defined as in Table 1. The strong correlations and positive slopes are indicative of carbocation character in the transition state resulting from diazirine activation.

Experimental *T*_onset_ and *T*_peak_ data were found to give robust linear fits (*R*^2^ ≥ 0.95) to empirically derived *σ*^+^_p_ values (Fig. 4). Calculated Δ*G*^‡^ values likewise showed a strong correlation to *σ*^+^_p_ (*R*^2^ = 0.91). By contrast, very poor fits (*R*^2^ < 0.1) were observed to empirically derived *σ*˙ values from Creary or Jiang (see Fig. S2 in the ESI† for plots of *T*_onset_ and *T*_peak_*vs.* all *σ* parameters). Together, these results argue in favour of an emerging vacant orbital at the benzylic centre during the transition state, and against the presence of any significant spin density at this position. These data provide compelling additional experimental support for the hypothesis that trifluoromethyl aryl diazirines initially form singlet carbenes upon activation, and that triplet carbenes are formed *via* a subsequent relaxation step.

As discussed above, trifluoromethyl aryl diazirines have dominated diazirine research ever since Brunner's publication in 1980. Perhaps because of this dominance, there are few direct comparisons of α-trifluoromethyl diazirines to diazirines containing different α-substituents. In an effort to partially address this deficiency, we surveyed five other α-substituents and compared their *T*_onset_ and *T*_peak_ values (Table 2).

**Table 2:** Effect of α-substituent electronics on activation temperature and activation free energy^a^

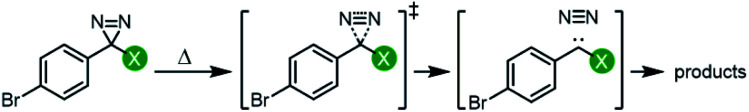				
Entry	X	*T*_onset_^b^ (°C)	*T*_peak_^c^ (°C)	Δ*G*^‡^^d^ (kJ mol^−1^)
1	Cl	76.5 ± 0.1	99.6 ± 0.2	152.7
2	H	84.6 ± 1.8	104.5 ± 0.5	147.2
3	CH_3_	93.5 ± 0.4	120.2 ± 0.1	153.8
4	CF_3_	105.6 ± 0.5	132.4 ± 0.6	147.8
5	F	108.4 ± 0.3	133.4 ± 0.4	156.8
6	OCH_3_	n.d.^e^	n.d.^e^	116.2

a In order to increase the molecular weight of the substrate (and thereby reduce volatility) experiments were carried out on *p*-brominated aryl diazirines.

b Experimentally determined by extrapolation of the tangent of the upward slope observed in the DSC experiment, to the fitted baseline of the plot. Data are presented as the average of three independent measurements ± standard deviation.

c Experimentally determined from the peak maxima in the DSC experiment. Data are presented as the average of three independent measurements ± standard deviation.

d Calculated free energy (298.15 K, 1 atm) of activation (M06-2X-D3/6-31+G(d,p)//M06-2X-D3/6-31G(d,p)) associated with conversion of the diazirine into the corresponding carbene in vacuum.

e The α-methoxy diazirine was too unstable to be analyzed by DSC, which is consistent with the low Δ*G*^‡^ that was found computationally, and with results from a different group.^46^

Lower activation temperatures were observed for α-H, α-chloro and α-methyl diazirines relative to α-CF_3_, while a representative α-fluoro diazirine had slightly higher *T*_onset_ and *T*_peak_ temperatures. The only α-methoxy compound studied was too unstable to properly study experimentally, but appeared to have a very low decomposition temperature.^46^ Preliminary multi-reference calculations indicate that there are varying degrees of multi-reference character in the transition states of the alpha-substituted species. The poor treatment of multi-reference systems by M06-2X leads to a weaker correlation between the calculated barrier heights and *T*_onset_ for the data in Table 2 than was seen for the equivalent data in Table 1.

### Expanded *in silico* Hammett studies

The robust linear free energy relationship observed in Fig. 4 stimulated several additional questions:

1. Would aryl diazirines with alpha substituents other than CF_3_ reveal similar tunability in activation temperature through the addition of electron donating or electron withdrawing groups on the aromatic ring? We were particularly interested here to study molecules with π donor groups at the alpha position (*e.g.* OMe or F). By donating into the unfilled p orbital of the singlet carbene, such groups could in principle alter the electronic preferences of the carbene intermediate, as well as the transition state energies.

2. Is it possible through judicious tuning of the electronics of an aryl diazirine to stabilize the singlet carbene sufficiently well that it becomes the lower energy state for the molecule? As discussed above, data from Sheridan and Lindel strongly implicate the involvement of singlet carbenes following activation of trifluoromethyl 4-methoxyphenyl diazirine, but it was not clear how this molecule fits into a larger trend.^47^

3. If a singlet state were favoured, would this improve the ability of the molecule to undergo C–H insertion with aliphatic substrates? Or would the stabilization that is necessary to lower the energy of the singlet state below that of the triplet necessarily then raise the activation barrier for C–H insertion, such that this becomes a less efficient process?

4. Given the very low activation barrier for α-methoxy diazirines, would these species be better or worse reagents from the perspective of C–H insertion? Alkoxy diazirines are valuable intermediates in glycoside synthesis,^48^ but less is known about how their properties compare to the more commonly exploited trifluoromethyl diazirines.^46,49^

Some of these questions are challenging to study experimentally. For example, the difficulty of working with α-methoxy diazirines (as noted above) makes it a challenge to derive analytically precise data describing their activation. Similarly, exploring the farther reaches of the Hammett plot by studying the inclusion of stronger electron donating groups like NMe_2_ (*σ*^+^_p_ = –1.7) or NH_2_ (*σ*^+^_p_ = –1.3) was ruled out as potentially hazardous to laboratory personnel, since these molecules would have significantly lower activation temperatures. Even studying the difference in performance for a *p*-OH group (*σ*^+^_p_ = –0.92) relative to the *p*-OMe substituent (*σ*^+^_p_ = –0.78) was ruled out experimentally, due to concerns that hydrogen bonding for the phenol in the solid state might complicate our analysis. Moreover, Hatanaka and co-workers have previously reported that this phenol is unstable and difficult to isolate.^10^

Of course, none of the above concerns apply to *in silico* experiments. We therefore sought to explore the above questions computationally, using an expanded Hammett series (R = 4-NMe_2_, 4-NH_2_, 4-OH, 4-OMe, 4-*t*-Bu, 4-CH_3_, 4-H, 4-Cl, 4-CF_3_, 4-CN and 4-NO_2_) and four different alpha substituents (X = CF_3_, Cl, F, and OCH_3_).

**Fig. 5:**
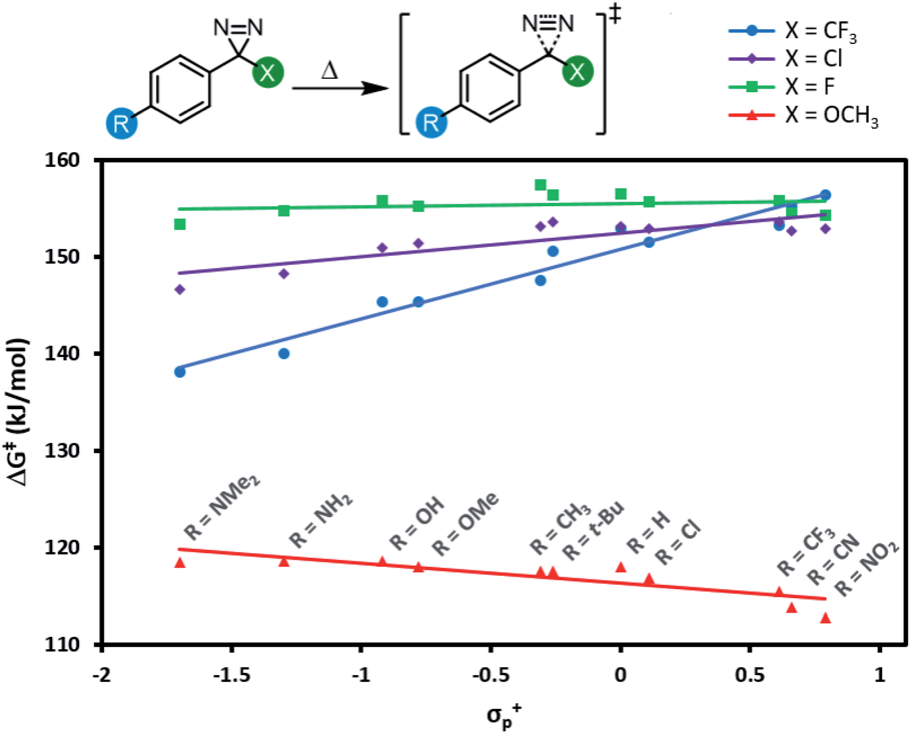
Variation in diazirine activation free energy (M06-2X-D3/6-31G(d,p)) with the Hammett parameter *σ*^+^_p_ for four different series of aryl diazirines.

All 44 diazirine activation reactions were modelled using M06-2X-D3/6-31G(d,p) methods, and the calculated activation free energies (Δ*G*^‡^) are shown in Fig. 5 (see Table S3† for complete tabulated data). The series of α-trifluoromethyl diazirines gave the largest positive slope of any of the four families of substrates. Because we know from Fig. 3 and 4 that diazirine activation energy tightly corresponds to *T*_onset_ and *T*_peak_ temperatures, this means that activation temperature would be much less tunable for α-fluoro, α-chloro and α-methoxy diazirines. In fact, the slope is essentially zero across the Hammett series when X = F (see Table S3† for tabulated slopes), and becomes negative when X = OCH_3_.

A series of computed isodesmic reactions (Fig. S3†) suggested that the trends observed in Fig. 5 are principally due to (de)stabilization of the transition states, rather than alteration of the ground-state diazirine energies. These data indicate that when X = CF_3_, electron-donating groups located across the aromatic ring from the diazirine centre can help to stabilize the empty p orbital that is evolving within the transition state. Once again this is consistent with a transition structure that is singlet-like, rather than triplet-like, and provides additional data in support of the hypothesis that singlet carbenes are initially formed following diazirine activation. This effect is blunted by incorporation of groups with increasing π-donating ability (X = Cl < F < OCH_3_) since these groups can themselves donate electron density to help stabilize the evolving empty p orbital. By the time we reach the strongest π-donor (X = OCH_3_), the evolving empty p orbital is fully stabilized, and we therefore observe no change in transition-state stabilization with differing substituents on the aromatic ring.

### Assessment of singlet–triplet gaps

**Fig. 6:**
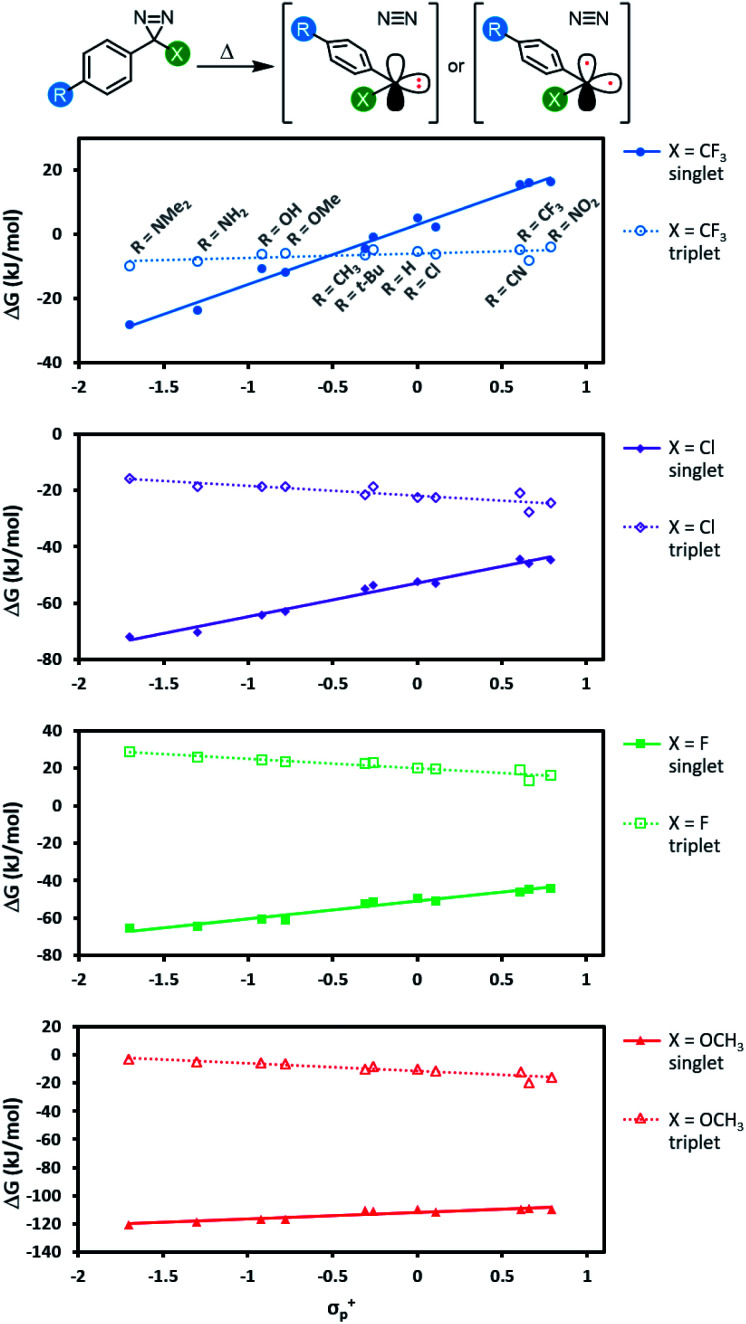
Free energy of reaction (DLPNO-CCSD(T)/cc-pV(D-T)Z CBS//M06-2X-D3/6-31G(d,p)) leading from diazirine starting materials to the corresponding singlet or triplet carbene. Refer to Fig. S4† for an equivalent plot where the same *y*-axis is used for each panel.

We next calculated the singlet–triplet energy gaps for our four Hammett series using domain based local pair natural orbital (DLPNO) coupled cluster methods (Fig. 6; see Table S6† for comparative data using different levels of theory).^50^

For each of the π-donors included in our study (X = Cl, F, or OCH_3_), the singlet was always the lowest energy state for the carbene, regardless of the identity of the *para* substituent (R) on the aromatic ring. As we saw with transition state energies, the ability of the α-substituent to stabilize the singlet carbene through the donation of electron density into the empty p orbital evidently outweighs other effects due to resonance donation by the distal R group.

For the α-trifluoromethyl series, we saw more interesting behaviour, wherein the singlet energy was dramatically affected by the ability of the R group to donate electron density, while the triplet energy remained essentially constant across the Hammett series. The result is a switch in the energy ordering of the two possible carbenes—when the aryl group was substituted with electron-neutral or electron-poor groups at the *para*-position, the triplet carbene was the lower energy species, but when a strong electron-donating group was added to the *para*-position of the aromatic ring, the singlet carbene became lower in energy. When R = OCH_3_, the singlet was calculated to be 6.9 kJ mol^−1^ lower in free energy (DLPNO-CCSD(T)/cc-pV(T-Q)Z//M06-2X-D3/6-31G(d,p)).

The data in Fig. 6 indicate two different options for producing aryl carbenes in which the singlet state is predominant: one may either employ an electron-donating α substituent such as an alkoxy group (in which case the electronics of the aromatic ring are largely immaterial) or else one can maintain the α-CF_3_ group that is widely used in literature crosslinking studies but add a sufficiently electron-donating *para* substituent to the aromatic ring.

### Calculation of C–H insertion barriers

Applications of diazirine groups in chemical biology or materials science demand that the carbene generated *in situ* be able to undergo efficient C–H insertion (or occasionally O–H insertion). However, the percent conversion for this process is typically quite poor (often <10%) because of the occurrence of side reactions. One particularly troublesome side-reaction for carbenes—often accounting for >50% of the mass balance in model studies of C–H insertion reactions—is quenching by adventitious oxygen to produce undesired ketone side products. It is thought that this mainly occurs through reaction of ^3^O_2_ with the triplet carbene. As such, the use of stabilized singlet carbenes (wherein little if any triplet is ever formed) may provide improved C–H insertion yields. On the other hand, if the singlet carbenes are too stabilized, then a higher energy barrier will be required for C–H insertion.

**Fig. 7:**
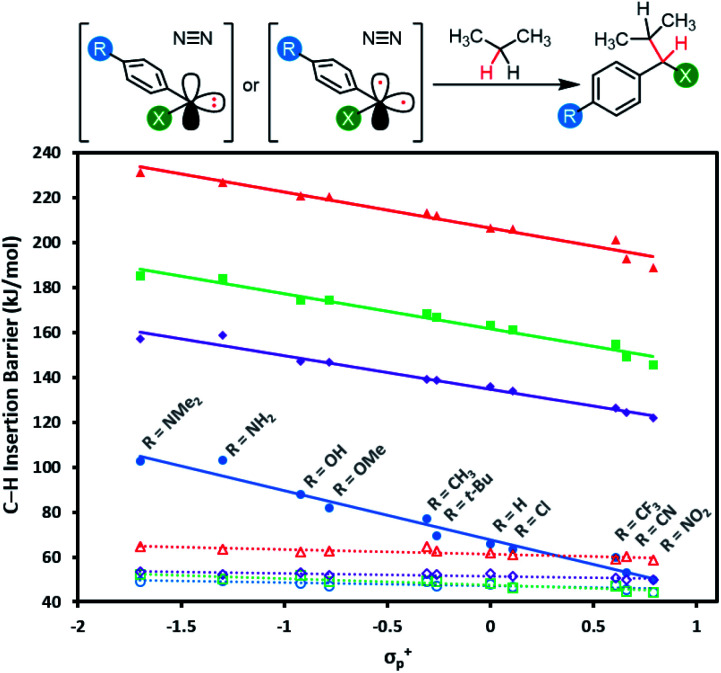
Calculated free energy barriers (M06-2X-D3/6-31G(d,p)) for insertion of singlet or triplet carbenes into the central C–H bond of propane. Data points and linear fits are reported as in Fig. 6: blue circles: X = CF_3_; purple diamonds: X = Cl; green squares: X = F; red triangles: X = OCH_3_; solid lines: data and fits for singlet species; dashed lines: data and fits for triplets. Refer to Fig. S5† for plots showing singlet and triplet insertions on separate axes.

Mindful of the potential benefits of improved C–H insertion reactions elicited by an ability to favour a singlet carbene intermediate, we computed the energy barriers for aliphatic C–H insertion (using the central CH_2_ motif of propane as a model of a typical aliphatic group), for all 88 singlet and triplet carbenes derived from aryl diazirines where X = CF_3_, Cl, F, or OCH_3_, and R = 4-NMe_2_, 4-NH_2_, 4-OH, 4-OMe, 4-*t*-Bu, 4-CH_3_, 4-H,4-Cl, 4-CF_3_, 4-CN or 4-NO_2_. Complete numerical data are tabulated in Table S3,† and are summarized graphically in Fig. 7.

The data reveal that for all four series of carbenes, the triplet C–H insertion barriers are always lower in energy than the corresponding singlet barriers, although the numbers are relatively close for electron-poor trifluoromethyl aryl diazirines (Δ[Δ*G*] = 5.5 kJ mol^−1^ when R = NO_2_). The lowest singlet C–H insertion barriers are found for the trifluoromethyl aryl diazirine series. Critically, these barriers are all lower than the Δ*G*^‡^ for the initial production of carbenes from the diazirine parent compounds (*ca.* 140–155 kJ mol; see Table 1 and Fig. 5).

Therefore, although the singlet C–H insertion barriers shown in Fig. 7 are higher than the barriers for the corresponding triplets, they must still be energetically achievable if the system had enough energy for diazirine activation. In other words, if a singlet α-CF_3_ carbene is produced, it should be able to undergo insertion with nearby C–H bonds; the rate-determining step for the process will be loss of N_2_ from the diazirine, and the subsequent insertion will be kinetically invisible.

This is not the case for the other carbene series. For α-OCH_3_ carbenes in particular, the singlet C–H insertion barriers are very high in free energy, which removes any possible benefit associated with the facile diazirine activation for these molecules.

### Optimal C–H insertion efficiency from an electron-rich diazirine

Taken together, the above data suggest that trifluoromethyl aryl diazirines bearing an electron-donating substituent at the *para*-position of the aromatic ring may be optimal: they will require a lower temperature for thermal activation, they should favour a singlet ground state for the carbene intermediate, and that singlet carbene should have sufficient energy to undergo C–H insertion. But what would be the most appropriate electron-donating group to use, when seeking to balance a desirable reactivity profile with good handling characteristics? Extrapolation of the red line in Fig. 4 and estimation of initiation temperature from *T*_onset_ (ref. 39) suggests that *p*-amino trifluoromethyl phenyl diazirines would be unstable above *ca*. 40 °C. In addition to obvious concerns around shock sensitivity or explosive propagation^5,38^ simply making and handling such a molecule on large scale could be problematic.

We therefore identified a *p*-alkoxy group as the optimal substituent to attach to the trifluoromethyl aryl diazirine scaffold. Our calculations suggest that it is sufficiently electron donating to favour the singlet ground state in the carbene (*i.e.* it falls just to the left of the intersection point in the top panel of Fig. 6), and yet our experimental measurements indicate that it is not so electron-donating as to result in an impractically low activation temperature. As an added benefit, *p*-alkoxy trifluoromethyl aryl diazines will have a longer wavelength *λ*_max_ and increased extinction coefficient associated with the diazirine activation band in the UV/Vis spectrum (Fig. 2).

Notwithstanding occasional earlier reports on the use of *p*-alkoxy trifluoromethyl aryl diazirines by Sheridan,^20^ Lindel,^34^ Kanaoka^36^ and Hashimoto,^51^ direct comparisons of efficacy between electron-rich, electron-neutral, and electron-poor aryl diazirines in their ability to insert into challenging (*i.e.* high energy) C–H bonds are lacking in the literature. Moreover, the few literature examples where electron-rich trifluoromethyl aryl diazirines are used are limited to photochemical activation methods. These examples are therefore relevant to chemical biology applications (where diazirines are almost always excited photochemically or else through energy transfer) but are less relevant to materials science applications, where thermal activation tends to predominate.

We therefore conducted a series of thermally and photochemically promoted C–H insertions with representative electron-rich, electron-neutral, and electron-poor trifluoromethyl aryl diazirines, employing cyclohexane as a substrate (C–H bond dissociation energy = 416 kJ mol^−1^).^52^ Consistent with our prediction from Fig. 6 that electron-donating groups at the *para*-position of the aromatic ring would stabilize a singlet carbene and allow for more selective C–H insertion reactions, we found that the installation of 4-OPh or 4-OCH_3_ substituents dramatically improved the isolated yield of the desired product (Table 3). This was particularly true for thermal activation conditions, in which the best-performing diazirine substrate was >10-fold more efficacious toward C–H insertion than the worst substrate. Regardless of the activation method, the 4-OCH_3_ group was found to be superior to the 4-OPh group, consistent with the improved electron-donating ability of the former substituent.

**Table 3:** Effect of aryl substituent electronics on cyclohexane C–H insertion

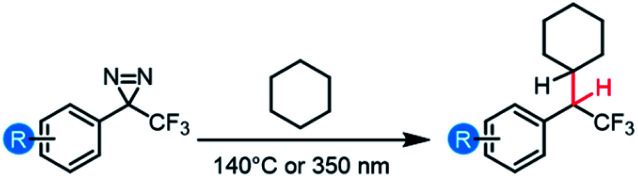			
Entry	R	Thermal C–H insertion yield^a^ (%)	Photochemical C–H insertion yield^b^ (%)
1	4-CF_3_	6	25
2	4-H	15	35
3	3-OCH_3_	18	19
4	4-OPh	75	77
5	4-OCH_3_	91	94

a Thermal C–H insertion reactions were performed using 15 mM of the desired diazirine in dry, degassed cyclohexane. Reaction mixtures were heated in a sealed tube at 140 °C for 2 h.

b Photochemical C–H insertion reactions were performed using 15 mM of the desired diazirine in dry, degassed cyclohexane. Reaction mixtures were irradiated at 350 nm for 4 h using a Rayonet reactor.

Careful examination of the crude NMR spectra for each of the reactions in Table 3 (see Fig. S70 and S71†) indicated the presence of significant quantities of ketone side products for the less-successful reactions, consistent with the hypothesis that for electron-poor or electron-neutral trifluoromethyl aryl carbenes, the triplet is the ground-state species. As further evidence for the mechanism of ketone formation, we examined the addition of triplet trifluoromethyl aryl carbenes to O_2_ computationally, and found this process to be essentially barrierless. Other side products appear to result from self-insertion pathways where one carbene molecule undergoes undesirable C–H insertion reactions with the aromatic moiety of the starting material.

**Scheme 1:**
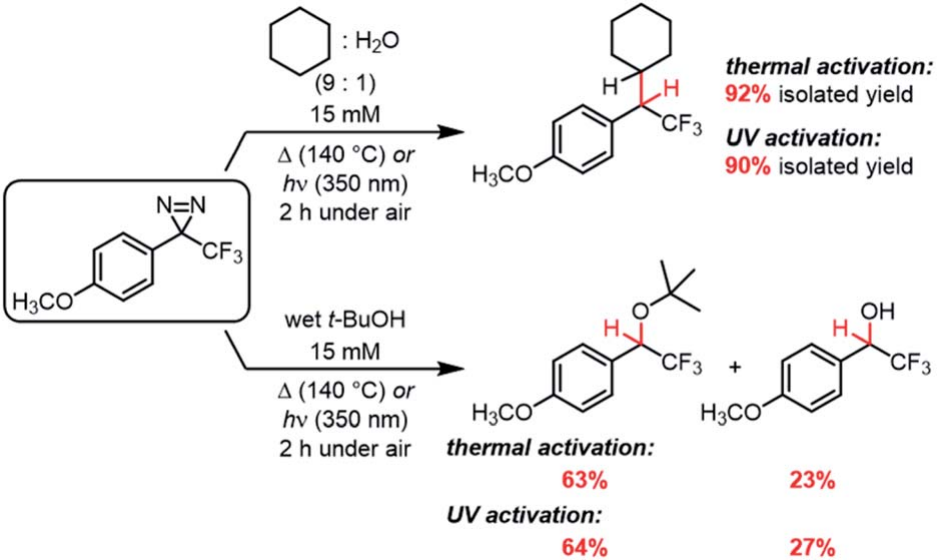
C–H and O–H insertions in the presence of air and moisture.

Whereas the data in Table 3 were collected using dry, degassed solvent, we also sought to study the reaction of the optimized substrate in less rigorous reaction conditions that might better mimic “real world” circumstances where diazirines need to be used. Thermal and photochemical reactions of *p*-methoxy trifluoromethyl phenyl diazirine were therefore repeated using 9 : 1 cyclohexane : water, and each reaction was run under air instead of inert atmosphere (Scheme 1). Remarkably, the desired C–H insertion adduct was once again the only isolable product, and could be obtained in ≥90% yield. Indeed, the crude NMR spectra for these “wet” reactions were identical to those obtained when the reaction was run using dry, deoxygenated cyclohexane (Fig. S72†).

While C–H insertions are often desired, O–H insertions can be equally useful for both protein labeling^34,53^ and polymer crosslinking.^5,12^ Raimer and Lindel had previously shown that *p*-methoxy trifluoromethyl phenyl diazirine could react with phenols,^34^ but because the proposed mechanism involved initial protonation by the acidic phenol, it was unclear what the efficiency of O–H insertion would be with alcohols. We therefore reacted our electronically optimized diazirine with *tert*-butanol under both photochemical and thermal activation conditions, once again intentionally carrying out the reaction under air and using non-anhydrous solvent (Scheme 1). Satisfyingly, the total yield of O–H insertion products was approximately 90% in both cases.

## Conclusions

Aryl diazirine reagents already find broad application in biological target identification, small molecule capture, and protein- and polymer-crosslinking, as well as in experimental adhesives for both commodity polymers and living tissue.^54^ However, in the vast majority of cases, the structure of the diazirine unit within these reagents has been chosen based on synthetic expedience rather than a consideration of what electronic properties might be optimal. In other words, all aryl diazirines have been assumed to be more or less equivalent in their ability to function as reactive “warheads”. In contrast to this view, the data presented in the current work provide compelling evidence that the properties of aryl diazirines can be readily adjusted by tuning the electronics of the aryl substituent. In particular, the observation that simply adding an alkoxy group to an aryl diazirine can improve the C–H insertion efficiency by >10-fold relative to an electronically unoptimized diazirine should stimulate the development of ever more effective reagents for the diverse array of applications discussed above.

## Data availability

Complete experimental and computational data are available in the ESI.†

## Author contributions

Synthetic work was performed by S. M. with assistance from L. B. DSC experiments were performed by L. B. Computational work was performed by Z. P., working under the supervision of G. D. The manuscript was written by J. W. and S. M. with the input of all authors.

## Conflicts of interest

There are no conflicts to declare.

## Supplementary Material

SC-012-D1SC03631A-s001

SC-012-D1SC03631A-s002

SC-012-D1SC03631A-s003

SC-012-D1SC03631A-s004

SC-012-D1SC03631A-s005

SC-012-D1SC03631A-s006

SC-012-D1SC03631A-s007

SC-012-D1SC03631A-s008

SC-012-D1SC03631A-s009

SC-012-D1SC03631A-s010

SC-012-D1SC03631A-s011

SC-012-D1SC03631A-s012

SC-012-D1SC03631A-s013

SC-012-D1SC03631A-s014

SC-012-D1SC03631A-s015

SC-012-D1SC03631A-s016

SC-012-D1SC03631A-s017

SC-012-D1SC03631A-s018

SC-012-D1SC03631A-s019

SC-012-D1SC03631A-s020

SC-012-D1SC03631A-s021

SC-012-D1SC03631A-s022

SC-012-D1SC03631A-s023

SC-012-D1SC03631A-s024

SC-012-D1SC03631A-s025

SC-012-D1SC03631A-s026

SC-012-D1SC03631A-s027

SC-012-D1SC03631A-s028

SC-012-D1SC03631A-s029

SC-012-D1SC03631A-s030

SC-012-D1SC03631A-s031

SC-012-D1SC03631A-s032

SC-012-D1SC03631A-s033

SC-012-D1SC03631A-s034

SC-012-D1SC03631A-s035

SC-012-D1SC03631A-s036

SC-012-D1SC03631A-s037

SC-012-D1SC03631A-s038

SC-012-D1SC03631A-s039

SC-012-D1SC03631A-s040

SC-012-D1SC03631A-s041

SC-012-D1SC03631A-s042

SC-012-D1SC03631A-s043

SC-012-D1SC03631A-s044

SC-012-D1SC03631A-s045

SC-012-D1SC03631A-s046

SC-012-D1SC03631A-s047

SC-012-D1SC03631A-s048

SC-012-D1SC03631A-s049

SC-012-D1SC03631A-s050

SC-012-D1SC03631A-s051

SC-012-D1SC03631A-s052

SC-012-D1SC03631A-s053

SC-012-D1SC03631A-s054

SC-012-D1SC03631A-s055

SC-012-D1SC03631A-s056

SC-012-D1SC03631A-s057

SC-012-D1SC03631A-s058

SC-012-D1SC03631A-s059

SC-012-D1SC03631A-s060

SC-012-D1SC03631A-s061

SC-012-D1SC03631A-s062

SC-012-D1SC03631A-s063

SC-012-D1SC03631A-s064

SC-012-D1SC03631A-s065

SC-012-D1SC03631A-s066

SC-012-D1SC03631A-s067

SC-012-D1SC03631A-s068

SC-012-D1SC03631A-s069

SC-012-D1SC03631A-s070

SC-012-D1SC03631A-s071

SC-012-D1SC03631A-s072

SC-012-D1SC03631A-s073

SC-012-D1SC03631A-s074

SC-012-D1SC03631A-s075

SC-012-D1SC03631A-s076

SC-012-D1SC03631A-s077

SC-012-D1SC03631A-s078

SC-012-D1SC03631A-s079

SC-012-D1SC03631A-s080

SC-012-D1SC03631A-s081

SC-012-D1SC03631A-s082

SC-012-D1SC03631A-s083

SC-012-D1SC03631A-s084

SC-012-D1SC03631A-s085

SC-012-D1SC03631A-s086

SC-012-D1SC03631A-s087

SC-012-D1SC03631A-s088

SC-012-D1SC03631A-s089

SC-012-D1SC03631A-s090

SC-012-D1SC03631A-s091

SC-012-D1SC03631A-s092

SC-012-D1SC03631A-s093

SC-012-D1SC03631A-s094

SC-012-D1SC03631A-s095

SC-012-D1SC03631A-s096

SC-012-D1SC03631A-s097

SC-012-D1SC03631A-s098

SC-012-D1SC03631A-s099

SC-012-D1SC03631A-s100

SC-012-D1SC03631A-s101

SC-012-D1SC03631A-s102

SC-012-D1SC03631A-s103

SC-012-D1SC03631A-s104

SC-012-D1SC03631A-s105

SC-012-D1SC03631A-s106

SC-012-D1SC03631A-s107

SC-012-D1SC03631A-s108

SC-012-D1SC03631A-s109

SC-012-D1SC03631A-s110

SC-012-D1SC03631A-s111

SC-012-D1SC03631A-s112

SC-012-D1SC03631A-s113

SC-012-D1SC03631A-s114

SC-012-D1SC03631A-s115

SC-012-D1SC03631A-s116

SC-012-D1SC03631A-s117

SC-012-D1SC03631A-s118

SC-012-D1SC03631A-s119

SC-012-D1SC03631A-s120

SC-012-D1SC03631A-s121

SC-012-D1SC03631A-s122

SC-012-D1SC03631A-s123

SC-012-D1SC03631A-s124

SC-012-D1SC03631A-s125

SC-012-D1SC03631A-s126

SC-012-D1SC03631A-s127

SC-012-D1SC03631A-s128

SC-012-D1SC03631A-s129

SC-012-D1SC03631A-s130

SC-012-D1SC03631A-s131

SC-012-D1SC03631A-s132

SC-012-D1SC03631A-s133

SC-012-D1SC03631A-s134

SC-012-D1SC03631A-s135

SC-012-D1SC03631A-s136

SC-012-D1SC03631A-s137

SC-012-D1SC03631A-s138

SC-012-D1SC03631A-s139

SC-012-D1SC03631A-s140

SC-012-D1SC03631A-s141

SC-012-D1SC03631A-s142

SC-012-D1SC03631A-s143

SC-012-D1SC03631A-s144

SC-012-D1SC03631A-s145

SC-012-D1SC03631A-s146

SC-012-D1SC03631A-s147

SC-012-D1SC03631A-s148

SC-012-D1SC03631A-s149

SC-012-D1SC03631A-s150

SC-012-D1SC03631A-s151

SC-012-D1SC03631A-s152

SC-012-D1SC03631A-s153

SC-012-D1SC03631A-s154

SC-012-D1SC03631A-s155

SC-012-D1SC03631A-s156

SC-012-D1SC03631A-s157

SC-012-D1SC03631A-s158

SC-012-D1SC03631A-s159

SC-012-D1SC03631A-s160

SC-012-D1SC03631A-s161

SC-012-D1SC03631A-s162

SC-012-D1SC03631A-s163

SC-012-D1SC03631A-s164

SC-012-D1SC03631A-s165

SC-012-D1SC03631A-s166

SC-012-D1SC03631A-s167

SC-012-D1SC03631A-s168

SC-012-D1SC03631A-s169

SC-012-D1SC03631A-s170

SC-012-D1SC03631A-s171

SC-012-D1SC03631A-s172

SC-012-D1SC03631A-s173

SC-012-D1SC03631A-s174

SC-012-D1SC03631A-s175

SC-012-D1SC03631A-s176

SC-012-D1SC03631A-s177

SC-012-D1SC03631A-s178

SC-012-D1SC03631A-s179

SC-012-D1SC03631A-s180

SC-012-D1SC03631A-s181

SC-012-D1SC03631A-s182

SC-012-D1SC03631A-s183

SC-012-D1SC03631A-s184

SC-012-D1SC03631A-s185

SC-012-D1SC03631A-s186

SC-012-D1SC03631A-s187

SC-012-D1SC03631A-s188

SC-012-D1SC03631A-s189

SC-012-D1SC03631A-s190

SC-012-D1SC03631A-s191

SC-012-D1SC03631A-s192

SC-012-D1SC03631A-s193

SC-012-D1SC03631A-s194

SC-012-D1SC03631A-s195

SC-012-D1SC03631A-s196

SC-012-D1SC03631A-s197

SC-012-D1SC03631A-s198

SC-012-D1SC03631A-s199

SC-012-D1SC03631A-s200

SC-012-D1SC03631A-s201

SC-012-D1SC03631A-s202

SC-012-D1SC03631A-s203

SC-012-D1SC03631A-s204

SC-012-D1SC03631A-s205

SC-012-D1SC03631A-s206

SC-012-D1SC03631A-s207

SC-012-D1SC03631A-s208

SC-012-D1SC03631A-s209

SC-012-D1SC03631A-s210

SC-012-D1SC03631A-s211

SC-012-D1SC03631A-s212

SC-012-D1SC03631A-s213

SC-012-D1SC03631A-s214

SC-012-D1SC03631A-s215

SC-012-D1SC03631A-s216

SC-012-D1SC03631A-s217

SC-012-D1SC03631A-s218

SC-012-D1SC03631A-s219

SC-012-D1SC03631A-s220

SC-012-D1SC03631A-s221

SC-012-D1SC03631A-s222

SC-012-D1SC03631A-s223

SC-012-D1SC03631A-s224

SC-012-D1SC03631A-s225

SC-012-D1SC03631A-s226

SC-012-D1SC03631A-s227

SC-012-D1SC03631A-s228

SC-012-D1SC03631A-s229

SC-012-D1SC03631A-s230

SC-012-D1SC03631A-s231

SC-012-D1SC03631A-s232

SC-012-D1SC03631A-s233

SC-012-D1SC03631A-s234

SC-012-D1SC03631A-s235

SC-012-D1SC03631A-s236

SC-012-D1SC03631A-s237

SC-012-D1SC03631A-s238

SC-012-D1SC03631A-s239

SC-012-D1SC03631A-s240

SC-012-D1SC03631A-s241

SC-012-D1SC03631A-s242

SC-012-D1SC03631A-s243

SC-012-D1SC03631A-s244

SC-012-D1SC03631A-s245

SC-012-D1SC03631A-s246

SC-012-D1SC03631A-s247

SC-012-D1SC03631A-s248

SC-012-D1SC03631A-s249

SC-012-D1SC03631A-s250

SC-012-D1SC03631A-s251

SC-012-D1SC03631A-s252

SC-012-D1SC03631A-s253

SC-012-D1SC03631A-s254

SC-012-D1SC03631A-s255

SC-012-D1SC03631A-s256

SC-012-D1SC03631A-s257

SC-012-D1SC03631A-s258

SC-012-D1SC03631A-s259

SC-012-D1SC03631A-s260

SC-012-D1SC03631A-s261

SC-012-D1SC03631A-s262

SC-012-D1SC03631A-s263

SC-012-D1SC03631A-s264

SC-012-D1SC03631A-s265

SC-012-D1SC03631A-s266

SC-012-D1SC03631A-s267

SC-012-D1SC03631A-s268

SC-012-D1SC03631A-s269

SC-012-D1SC03631A-s270

SC-012-D1SC03631A-s271

SC-012-D1SC03631A-s272

SC-012-D1SC03631A-s273

SC-012-D1SC03631A-s274

SC-012-D1SC03631A-s275

SC-012-D1SC03631A-s276

SC-012-D1SC03631A-s277

SC-012-D1SC03631A-s278

SC-012-D1SC03631A-s279

SC-012-D1SC03631A-s280

SC-012-D1SC03631A-s281

SC-012-D1SC03631A-s282

SC-012-D1SC03631A-s283

SC-012-D1SC03631A-s284

SC-012-D1SC03631A-s285

SC-012-D1SC03631A-s286

SC-012-D1SC03631A-s287

SC-012-D1SC03631A-s288

SC-012-D1SC03631A-s289

SC-012-D1SC03631A-s290

SC-012-D1SC03631A-s291

SC-012-D1SC03631A-s292

SC-012-D1SC03631A-s293

SC-012-D1SC03631A-s294

SC-012-D1SC03631A-s295

SC-012-D1SC03631A-s296

SC-012-D1SC03631A-s297

SC-012-D1SC03631A-s298

SC-012-D1SC03631A-s299

SC-012-D1SC03631A-s300

SC-012-D1SC03631A-s301

SC-012-D1SC03631A-s302

SC-012-D1SC03631A-s303

SC-012-D1SC03631A-s304

SC-012-D1SC03631A-s305

SC-012-D1SC03631A-s306

SC-012-D1SC03631A-s307

SC-012-D1SC03631A-s308

SC-012-D1SC03631A-s309

SC-012-D1SC03631A-s310

SC-012-D1SC03631A-s311

SC-012-D1SC03631A-s312

SC-012-D1SC03631A-s313

SC-012-D1SC03631A-s314

SC-012-D1SC03631A-s315

SC-012-D1SC03631A-s316

SC-012-D1SC03631A-s317
