# Supplementary material for: Structure–function relationships in aryl diazirines reveal optimal design features to maximize C–H insertion
Source: Chem Sci. 2021 Aug 10;12(36):12138–48. doi: 10.1039/d1sc03631a (PMC8457397; doi:10.1039/d1sc03631a)
Supplement: SC-012-D1SC03631A-s001 [file SC-012-D1SC03631A-s001.pdf]

Electronic Supplementary Material (ESI) for Chemical Science.
This journal is © The Royal Society of Chemistry 2021

Electronic Supplementary Information for:

Structure–function relationships in aryl diazirines reveal optimal design features to maximize C–H insertion

Stefania F. Musolino, Zhipeng Pei, Liting Bi, Gino A. DiLabio, and Jeremy E. Wulff

Table of Contents

Supplemental Figures 3
Computational Details 8
Materials and Methods 14
General considerations 14
Synthesis of aryl diazirines 14
Synthesis of 2,2,2-trifluoro-1-(4-methoxyphenyl)ethan-1-one (**S1**). 15
Synthesis of 2,2,2-trifluoro-1-(4-methoxyphenyl)ethan-1-one oxime (**S2**) (mixture of *E*-, *Z*-isomers). 15
Synthesis of 2,2,2-trifluoro-1-(4-methoxyphenyl)ethan-1-one *O*-tosyl oxime (**S3**) (mixture of *E*-, Z-isomers). 16
Synthesis of 3-(4-methoxyphenyl)-3-(trifluoromethyl)diaziridine (**S4**). 16
Synthesis of 3-(4-methoxyphenyl)-3-(trifluoromethyl)-3*H*-diazirine (**D1**). 16
Synthesis of 2,2,2-trifluoro-1-(4-phenoxyphenyl)ethan-1-one (**S5**). 18
Synthesis of 2,2,2-trifluoro-1-(4-phenoxyphenyl)ethan-1-one oxime (**S6**) (mixture of *E*-, *Z*-isomers). 18
Synthesis of 2,2,2-trifluoro-1-(4-phenoxyphenyl)ethan-1-one *O*-tosyl oxime (**S7**) (mixture of *E*-, *Z*-isomers). 19
Synthesis of 3-(4-phenoxyphenyl)-3-(trifluoromethyl)diaziridine (**S8**). 19
Synthesis of 3-(4-phenoxyphenyl)-3-(trifluoromethyl)-3*H*-diazirine (**D2**). 19
Synthesis of 1-(4-(tert-butyl)phenyl)-2,2,2-trifluoroethan-1-one (**S9**). 21
Synthesis of 1-(4-(tert-butyl)phenyl)-2,2,2-trifluoroethan-1-one oxime (**S10**) (mixture of *E*-, *Z*-isomers). 21
Synthesis of 1-(4-(tert-butyl)phenyl)-2,2,2-trifluoroethan-1-one *O*-tosyl oxime (**S11**) (mixture of *E*-, *Z*-isomers). 22
Synthesis of 3-(4-(tert-butyl)phenyl)-3-(trifluoromethyl)diaziridine (**S12**). 22
Synthesis of 3-(4-(tert-butyl)phenyl)-3-(trifluoromethyl)-3*H*-diazirine (**D3**). 22
Synthesis of 1-(4-bromophenyl)-2,2,2-trifluoroethan-1-one (**S13**). 24
Synthesis of 1-(4-bromophenyl)-2,2,2-trifluoroethan-1-one oxime (**S14**) (mixture of *E*-, *Z*-isomers). 24
Synthesis of 1-(4-bromophenyl)-2,2,2-trifluoroethan-1-one *O*-tosyl oxime (**S15**) (mixture of *E*-, *Z*-isomers). 25
Synthesis of 3-(4-bromophenyl)-3-(trifluoromethyl)diaziridine (**S16**). 25
Synthesis of 3-(4-bromophenyl)-3-(trifluoromethyl)-3*H*-diazirine (**D5**). 25
Synthesis of 2,2,2-trifluoro-1-(3-methoxyphenyl)ethan-1-one (**S17**). 27
Synthesis of 2,2,2-trifluoro-1-(3-methoxyphenyl)ethan-1-one oxime (**S18**) (mixture of *E*-, *Z*-isomers). 27
Synthesis of 2,2,2-trifluoro-1-(3-methoxyphenyl)ethan-1-one *O*-tosyl oxime (**S19**) (mixture of *E*-, *Z*-isomers). 27
Synthesis of 3-(3-methoxyphenyl)-3-(trifluoromethyl)diaziridine (**S20**). 27
Synthesis of 3-(3-methoxyphenyl)-3-(trifluoromethyl)-3*H*-diazirine (**D8**). 28
Synthesis of 3-(3-hydroxyphenyl)-3-(trifluoromethyl)-3*H*-diazirine (**D9**). 29
Synthesis of methyl 3,5-dimethoxybenzoate (**S21**). 30
Synthesis of 1-(3,5-dimethoxyphenyl)-2,2,2-trifluoroethan-1-one (**S22**). 30
Synthesis of 1-(3,5-dimethoxyphenyl)-2,2,2-trifluoroethan-1-one oxime (**S23**) (mixture of *E*-, *Z*-isomers). 31
Synthesis of 1-(3,5-dimethoxyphenyl)-2,2,2-trifluoroethan-1-one *O*-tosyl oxime (**S24**) (mixture of *E*-, *Z*-isomers). 31
Synthesis of 3-(3,5-dimethoxyphenyl)-3-(trifluoromethyl)diaziridine (**S25**). 31
Synthesis of 3-(3,5-dimethoxyphenyl)-3-(trifluoromethyl)-3*H*-diazirine (**D10**). 31

Synthesis of methyl 4-(trifluoromethyl)benzoate (**S26**). 33
Synthesis of 2,2,2-trifluoro-1-(4-(trifluoromethyl)phenyl)ethan-1-one (**S27**). 33
Synthesis of 2,2,2-trifluoro-1-(4-(trifluoromethyl)phenyl)ethan-1-one oxime (**S28**) (mixture of *E*-, *Z*-isomers). 33
Synthesis of 2,2,2-trifluoro-1-(4-(trifluoromethyl)phenyl)ethan-1-one *O*-tosyl oxime (**S29**) (mixture of *E,Z*-isomers). 34
Synthesis of 3-(trifluoromethyl)-3-(4-(trifluoromethyl)phenyl)diaziridine (**S30**). 34
Synthesis of 3-(trifluoromethyl)-3-(4-(trifluoromethyl)phenyl)-3*H*-diazirine (**D11**). 34
Synthesis of 4-[3-(Trifluoromethyl)-3*H*-diazirin-3-yl]benzaldehyde (**D12**). 36
Synthesis of 2,2,2-trifluoro-1-(4-nitrophenyl)ethan-1-one (**S31**). 37
Synthesis of 2,2,2-trifluoro-1-(4-nitrophenyl)ethan-1-one oxime (**S32**) (mixture of *E*-, *Z*-isomers). 38
Synthesis of 2,2,2-trifluoro-1-(4-nitrophenyl)ethan-1-one *O*-tosyl oxime (**S33**) (mixture of *E*-, *Z*-isomers). 38
Synthesis of 3-(4-nitrophenyl)-3-(trifluoromethyl)diaziridine (**S34**). 38
Synthesis of 3-(4-nitrophenyl)-3-(trifluoromethyl)-3*H*-diazirine (**D13**). 38
Synthesis of 3-(4-bromophenyl)-3-chloro-3*H*-diazirine (**D14**). 40
Synthesis of 3-(4-bromophenyl)-3*H*-diazirine (**D15**). 41
Synthesis of 3-(4-bromophenyl)-3-methyl-3*H*-diazirine (**D16**). 42
Synthesis of 3-(4-bromophenyl)-3-fluoro-3*H*-diazirine (**D17**). 43
General protocols for insertion reactions 45
Insertion reactions in cyclohexane 46
Preparation of 1-(1-cyclohexyl-2,2,2-trifluoroethyl)-4-(trifluoromethyl)benzene (**S35**). 46
Preparation of (1-cyclohexyl-2,2,2-trifluoroethyl)benzene (**S36**). 48
Preparation of 1-(1-cyclohexyl-2,2,2-trifluoroethyl)-3-methoxybenzene (**S37**). 51
Preparation of 1-(1-cyclohexyl-2,2,2-trifluoroethyl)-4-phenoxybenzene (**S38**). 53
Preparation of 1-(1-cyclohexyl-2,2,2-trifluoroethyl)-4-methoxybenzene (**S39**). 56
Insertion reactions in a wet environment 60
Insertion to cyclohexane:water (9:1) 60
Insertion to *tert*-Butanol 61
Monitoring of diazo-isomer formation during thermal activation of diazirines 64
Decomposition of **D14**, **D15**, **D16**, and **D5** under thermal activation. 64
Differential Scanning Calorimetry (DSC) 67
General Protocol for DSC analysis 67
DSC plots of aryl diazirines 67
References 76

Supplemental Figures

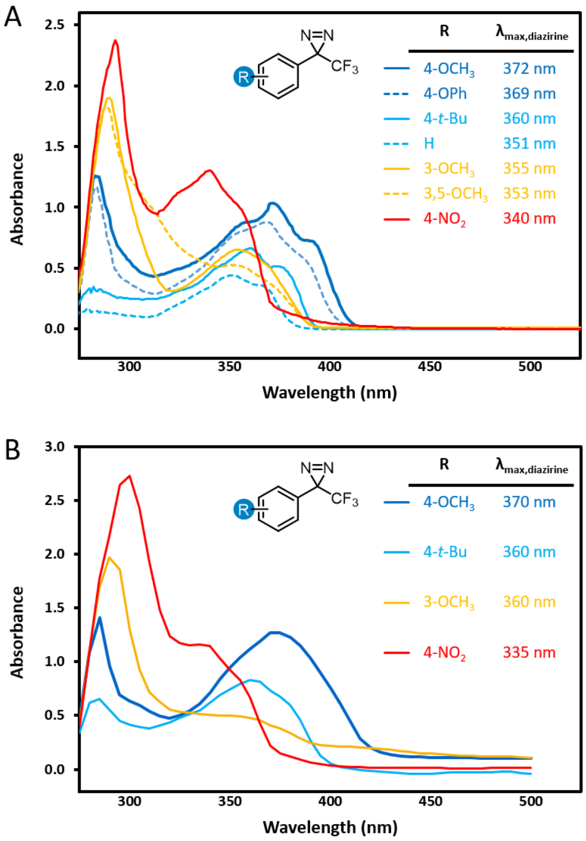

Fig. S1 Comparison of UV/Vis spectra for representative diazirines in nonpolar and polar solvents. (A) Spectra recorded in *n*-hexane, using 1 nm steps. (B) Spectra recorded in methanol, using 5 nm steps.

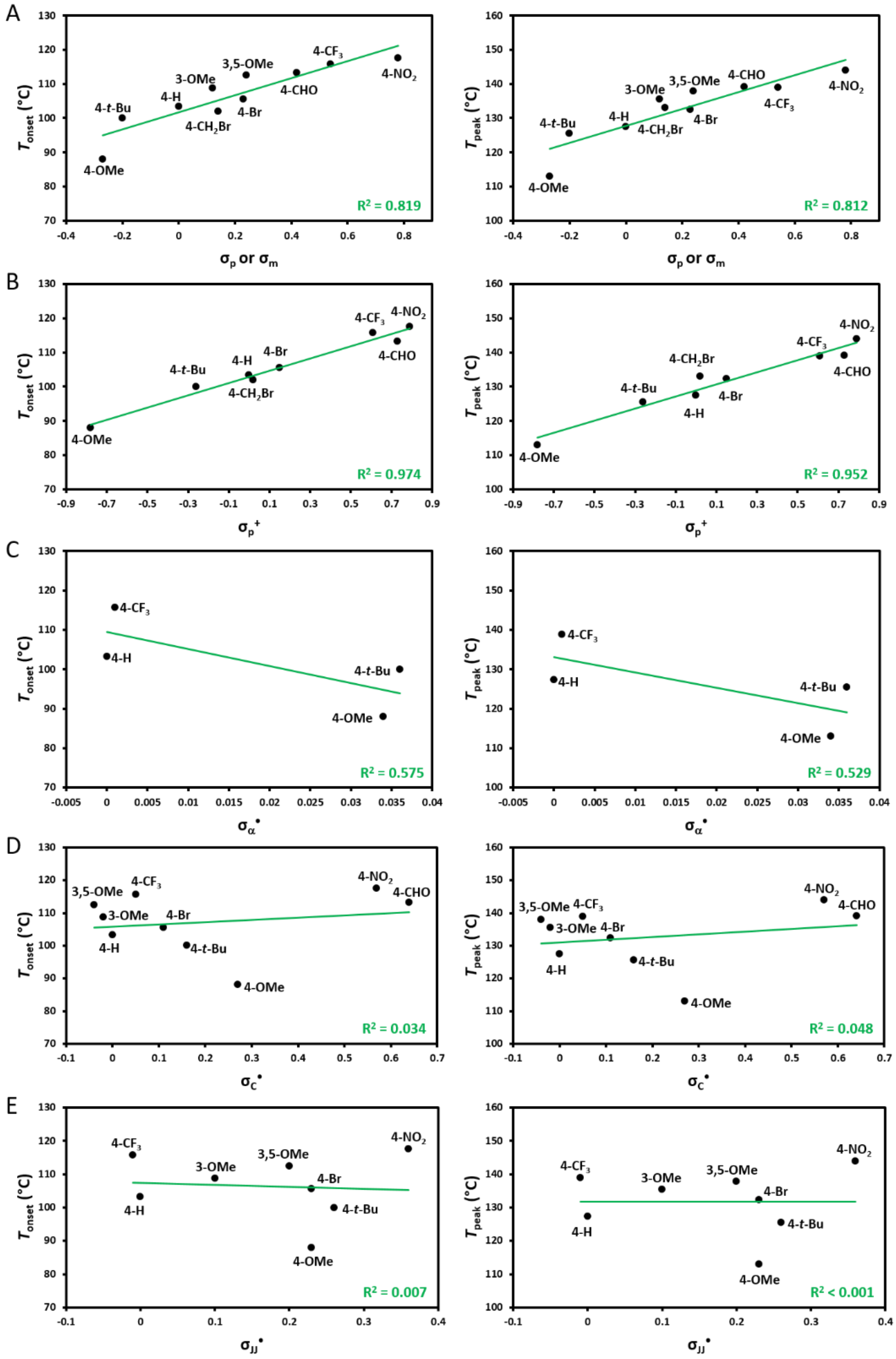

Fig. S2 Fits of experimental T_{onset} and T_{peak} data to empirically derived Hammett parameters. (A) Fits generated using Hammett's original σ parameters derived from the study of *para*-substituted benzoic acid ionization. (B) Fits generated using Brown's σ_p^+ parameters derived from observation of solvolysis of substituted *t*-cumyl chlorides. (C) Fits generated using Arnold's $\sigma_\alpha^\bullet$ parameters derived from the study of EPR hyperfine coupling of benzyl radicals. (D) Fits generated using Creary's $\sigma_C^\bullet$ parameters derived from the rearrangement of methylenecyclopropane ring systems. (E) Fits generated using Jiang and Ji's $\sigma_{jj}^\bullet$ parameters derived from the cyclodimerization of trifluorostyrenes. Excellent fits are observed using Brown's σ_p^+ parameters, while very poor fits are observed using any of the $\sigma^\bullet$ parameters. These data support some degree of carbocation character in the transition state, and are not consistent with radical character at the benzylic center during the rate-determining step.

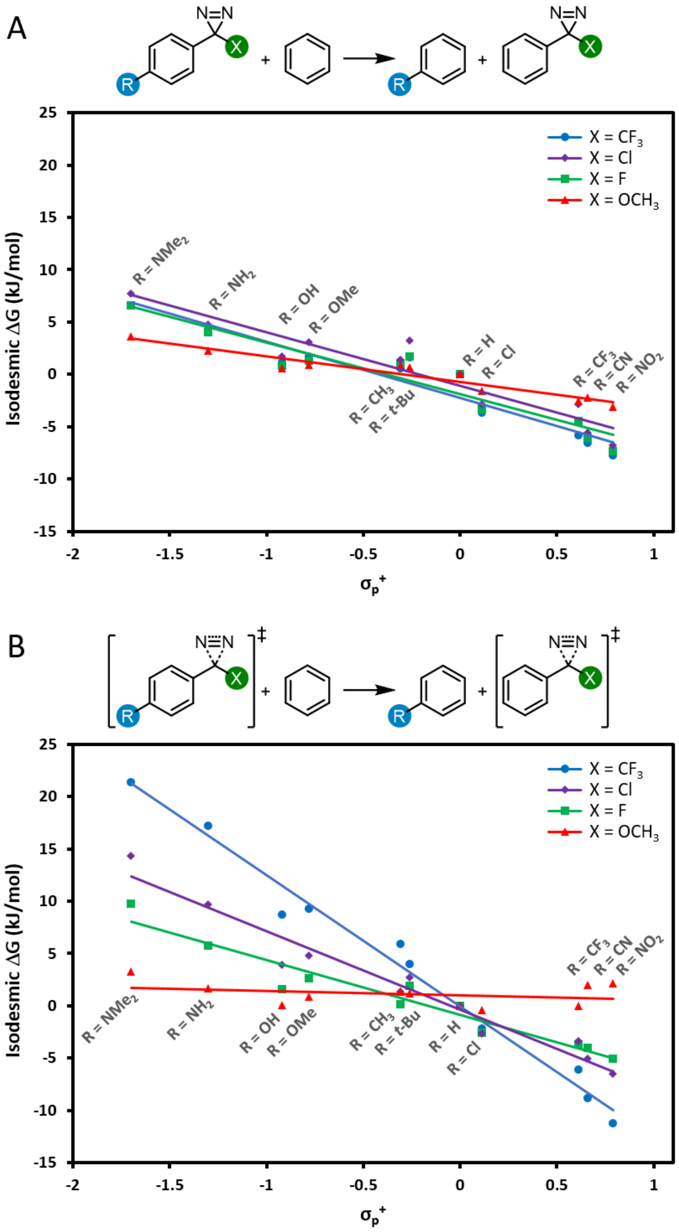

Fig. S3 Isodesmic equations used to explore the effect of aryl substituents on energetics of the diazirine ground state (panel A) and transition state (panel B). All calculations were performed using M06-2X-D3/6-31G(d,p). The data in panel A indicate that varying the electronic properties of the aryl substituent has only minor (and very consistent) effects on the energy of the ground-state diazirine; the consistent negative slope across all four series of isodesmic reactions indicates that the CN_2X function constitutes a weak electron withdrawing group. The data in panel B indicate that when X=CF_3, electron-donating groups located across the aromatic ring from the diazirine centre can help to stabilize the empty p orbital that is evolving within the transition state. However, this effect is blunted by incorporation of groups with increasing π-donating ability (X=Cl < F < OCH_3) since these groups can themselves donate electron density to help stabilize the evolving empty p orbital.

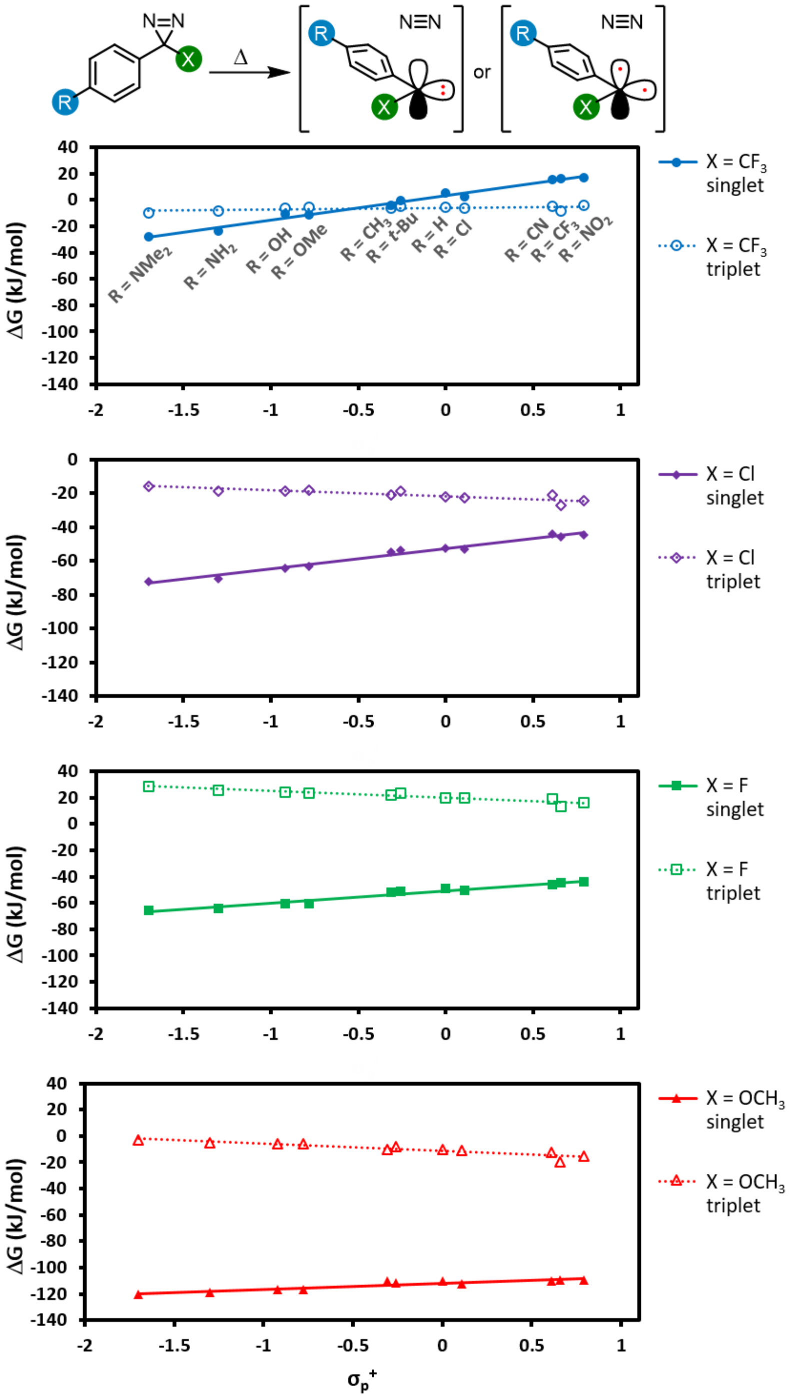

Fig. S4 Alternative presentation of the data in Fig. 6, in which the same y-axis is used for each panel.

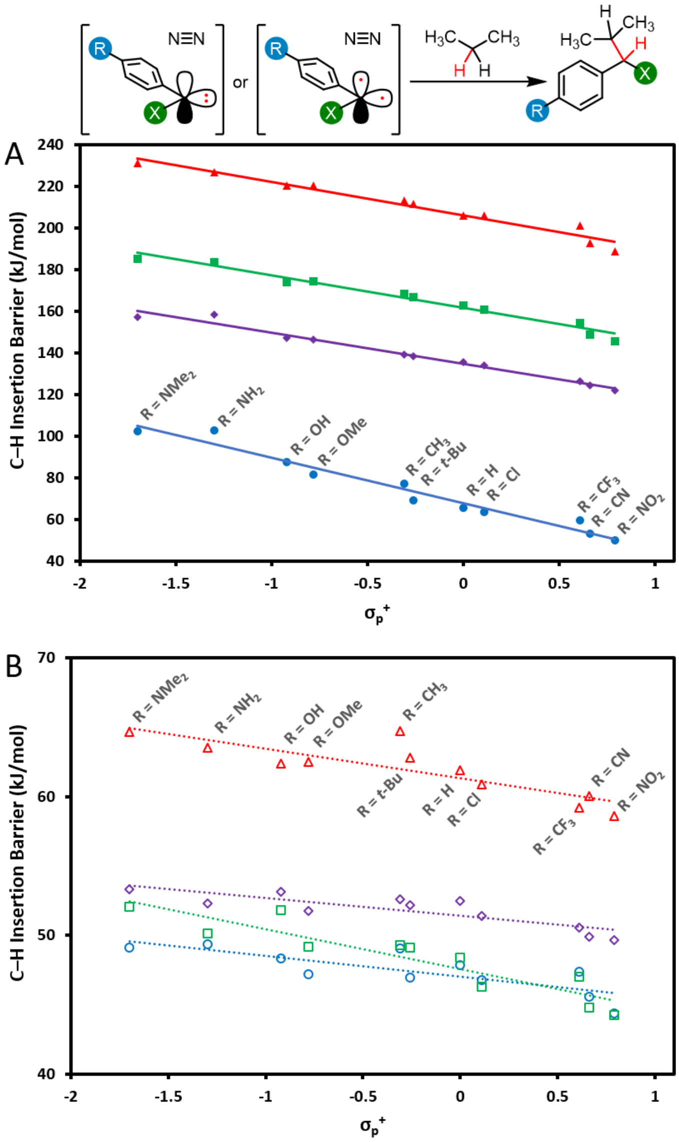

Fig. S5 Alternative presentation of the data in Fig. 7, in which the data for the singlet insertions and triplet insertions are plotted separately to improve visibility. Data and fits for singlet species (solid lines) are shown in panel A. Data and fits for triplet species (dashed lines) are shown in panel B.

Computational Details

Geometry optimization and frequency calculations were performed using M06-2X[1]-D3[2]/6-31G(d,p) at 298 K and 1 atm. A minima point was confirmed when all of the vibrations associated with a molecule's normal modes were positive, and a transition state (TS) structure was characterized by the presence of only one imaginary frequency that connects reactants to products. Additional single-point energies were calculated using the 6-31+G(d,p) basis set for data in Table 1 and Table 2. All DFT results were obtained with the Gaussian 16 package.[3]

For Fig. 6, DLPNO-CCSD(T)[4]/CBS with TightPNO option single-point energies were included, using the extrapolation scheme of cc-pVDZ and cc-pVTZ recommended in the ORCA manual.[5] All these calculations were carried out using the ORCA 4.2.1 package.[6]

The computational techniques used here were chosen on the basis of parallel work from our group,[7] wherein we show that M06-2X-D3 had some of the lowest absolute errors and lowest mean errors in computed barrier heights and energies for 449 reactions amongst the 12 common functionals tested. We also showed that complete basis set DLPNO-CCSD(T) produced barrier heights and reaction energies for a cross-section of the 449 reactions that were within 0.5 kcal/mol of canonical, complete basis set CCSD(T).

Table S1 Summary of the calculated free energy barriers (298 K, 1 atm) of carbene formations at M06-2X-D3/6-31+G(d,p)//M06-2X-D3/6-31G(d,p) for X=CF_3 diazirines. The units of single-point energies and free energy corrections are in E_h, and the barriers are in kJ/mol. These data are used in Table 1.

**X=CF_3**			**diazirine**		**carbene formation TS**		**carbene formation barrier (kJ/mol)**
T_{onset}	**#**	**R**	**single-point**	**thermal**	**single-point**	**thermal**	
88.0	1	4-OCH_3	-831.0795518	0.114832	-831.0213133	0.110526	141.6
90.2	2	4-OPh	-1022.754237	0.161346	-1022.695422	0.155958	140.3
100.0	3	4-*t*-Bu	-873.7851535	0.192149	-873.724229	0.187219	147.0
103.3	4	4-H	-716.5958686	0.085054	-716.5340457	0.080302	149.8
105.6	5	4-Br	-3287.800083	0.072023	-3287.738626	0.066862	147.8
106.8	6	4-CH_2OH	-831.0852765	0.112979	-831.0244056	0.108895	149.1
102.0	7	4-CH_2Br	-3327.092416	0.097804	-3327.030973	0.092775	148.1
108.7	8	3-OCH_3	-831.0792245	0.114743	-831.0165079	0.110092	152.5
110.5	9	3-OH	-791.7974855	0.087953	-791.7346427	0.083055	152.1
112.5	10	3,5-OCH_3	-945.5620573	0.144616	-945.5006585	0.140083	149.3
115.7	11	4-CF_3	-1053.549729	0.08415	-1053.486691	0.078515	150.7
113.2	12	4-CHO	-829.8813742	0.091449	-829.8185819	0.086989	153.2
117.6	13	4-NO_2	-921.0272248	0.084011	-920.9637568	0.079302	154.3

Table S2 Summary of the calculated free energy barriers (298 K, 1 atm) of carbene formations at M06-2X-D3/6-31+G(d,p)//M06-2X-D3/6-31G(d,p) for R=Br diazirines. The units of single-point energies and free energy corrections are in E_h, and the barriers are in kJ/mol. These data are used in Table 2.

R=Br			diazirine		carbene formation TS		carbene formation barrier (kJ/mol)
T_{onset}	#	X	single-point	thermal	single-point	thermal	
76.5	1	Cl	-3410.41549	0.059615	-3410.353335	0.055608	152.7
84.6	2	H	-2950.84887	0.07169	-2950.787209	0.066082	147.2
93.5	3	CH_3	-2990.15124	0.098135	-2990.087606	0.093075	153.8
105.6	4	CF_3	-3287.80008	0.072023	-3287.738626	0.066862	147.8
108.4	5	F	-3050.06402	0.062596	-3050.000009	0.058312	156.8
N/A	6	OCH_3	-3065.33306	0.100734	-3065.285585	0.097526	116.2

Table S3 Summary of the calculated free energy (298 K, 1 atm) S-T gaps[a] and barriers of carbene formation, singlet carbene insertion, and triplet carbene abstraction using M06-2X-D3/6-31G(d,p) for X=CF_3, Cl, F, and OMe. The central C–H bond in propane is targeted for insertion or abstraction reaction modeling. The units are in kJ/mol. These data are used in Fig. 5 and Fig. 7.

X=CF_3					
σ_p^+	R	S-T gap	formation barrier	insertion barrier	abstraction barrier
-1.7	NMe_2	10.4	138.2	102.6	49.1
-1.3	NH_2	7.3	140.0	103.0	49.3
-0.92	OH	19.7	145.4	87.6	48.3
-0.78	OMe	18.8	145.4	81.6	47.2
-0.31	CH_3	27.1	147.6	77.1	49.0
-0.26	*t*-Bu	29.9	150.6	69.2	47.0
0	H	34.1	153.0	65.6	47.9
0.11	Cl	33.9	151.5	63.5	46.7
0.61	CF_3	43.1	153.2	59.6	47.4
0.66	CN	45.6	155.3	53.0	45.6
0.79	NO_2	46.7	156.5	49.8	44.4
		slope =	***+ 7.2***		

X=Cl					
σ_p^+	R	S-T gap	formation barrier	insertion barrier	abstraction barrier
-1.7	NMe_2	-38.9	146.6	157.1	53.3
-1.3	NH_2	-39.1	148.2	158.6	52.3
-0.92	OH	-28.4	150.9	147.1	53.1
-0.78	OMe	-28.1	151.5	146.6	51.7
-0.31	CH_3	-18.8	153.2	139.2	52.6
-0.26	*t*-Bu	-18.7	153.7	138.6	52.2
0	H	-15.5	153.2	135.8	52.5
0.11	Cl	-14.6	152.9	133.9	51.4
0.61	CF_3	-6.3	153.7	126.4	50.5
0.66	CN	-2.3	152.7	124.4	49.9
0.79	NO_2	0.8	152.9	121.8	49.7
		slope =	***+ 2.4***		

X=F					
σ_p^+	R	S-T gap	formation barrier	insertion barrier	abstraction barrier
-1.7	NMe_2	-75.1	153.4	185.2	52.0
-1.3	NH_2	-73.7	154.8	183.8	50.1
-0.92	OH	-66.6	155.8	174.2	51.8
-0.78	OMe	-66.2	155.2	174.5	49.2
-0.31	CH_3	-59.0	157.5	168.5	49.3
-0.26	*t*-Bu	-57.8	156.4	166.7	49.1
0	H	-54.7	156.6	163.0	48.4
0.11	Cl	-54.4	155.8	161.0	46.3
0.61	CF_3	-48.1	155.8	154.6	47.0
0.66	CN	-42.4	154.6	149.0	44.8
0.79	NO_2	-39.6	154.3	145.5	44.2
		slope =	***+ 0.3***		

X=OMe					
σ_p^+	R	S-T gap	formation barrier	insertion barrier	abstraction barrier
-1.7	NMe_2	-97.8	118.4	231.4	64.7
-1.3	NH_2	-95.4	118.6	226.7	63.5
-0.92	OH	-92.3	118.6	220.6	62.4
-0.78	OMe	-91.3	118.0	220.4	62.5
-0.31	CH_3	-83.8	117.5	213.2	64.7
-0.26	*t*-Bu	-85.4	117.6	211.8	62.8
0	H	-84.2	118.0	206.3	61.9
0.11	Cl	-84.0	116.8	205.9	60.9
0.61	CF_3	-79.4	115.5	201.2	59.2
0.66	CN	-72.6	113.8	192.8	60.0
0.79	NO_2	-70.7	112.8	188.8	58.6
		slope =	***– 2.0***		

[a] A positive S-T gap indicates that the ground state is in a triplet state.

Table S4 Summary of the calculated (298 K, 1 atm) isodesmic free energies (of the parent reaction or TS reaction) using M06-2X-D3/6-31G(d,p) for X=CF_3, Cl, F, and OMe. The units are in kJ/mol. These data are used in Fig. S3.

**X=CF_3**			
σ_p^+	**R**	**isodesmic free energy (parent)**	**isodesmic free energy (TS)**
-1.7	NMe_2	6.6	21.4
-1.3	NH_2	4.3	17.2
-0.92	OH	1.1	8.7
-0.78	OMe	1.7	9.3
-0.31	CH_3	0.6	6.0
-0.26	*t*-Bu	1.6	4.0
0	H	0.0	0.0
0.11	Cl	-3.6	-2.2
0.61	CF_3	-5.8	-6.1
0.66	CN	-6.5	-8.8
0.79	NO_2	-7.7	-11.2
	slope =	***– 5.4***	**– 12.5**

**X=Cl**			
σ_p^+	**R**	**isodesmic free energy (parent)**	**isodesmic free energy (TS)**
-1.7	NMe_2	7.8	14.3
-1.3	NH_2	4.7	9.7
-0.92	OH	1.7	3.9
-0.78	OMe	3.0	4.8
-0.31	CH_3	1.4	1.4
-0.26	*t*-Bu	3.2	2.7
0	H	0.0	0.0
0.11	Cl	-2.9	-2.7
0.61	CF_3	-2.9	-3.3
0.66	CN	-5.6	-5.1
0.79	NO_2	-6.8	-6.5
	slope =	***– 5.1***	**– 7.5**

**X group=F**			
σ_p^+	**R**	**isodesmic free energy (parent)**	**isodesmic free energy (TS)**
-1.7	NMe_2	6.6	9.8
-1.3	NH_2	4.0	5.8
-0.92	OH	0.9	1.6
-0.78	OMe	1.3	2.6
-0.31	CH_3	1.1	0.2
-0.26	*t*-Bu	1.7	1.9
0	H	0.0	0.0
0.11	Cl	-3.4	-2.6
0.61	CF_3	-4.4	-3.7
0.66	CN	-6.0	-4.0
0.79	NO_2	-7.3	-5.1
	slope =	***– 4.9***	**– 5.3**

X=OMe			
σ_p^+	R	isodesmic free energy (parent)	isodesmic free energy (TS)
-1.7	NMe_2	3.7	3.3
-1.3	NH_2	2.3	1.7
-0.92	OH	0.6	0.1
-0.78	OMe	0.9	0.9
-0.31	CH_3	0.8	1.3
-0.26	*t*-Bu	0.7	1.2
0	H	0.0	0.0
0.11	Cl	-1.6	-0.4
0.61	CF_3	-2.6	0.0
0.66	CN	-2.2	2.0
0.79	NO_2	-3.1	2.1
	slope =	***– 2.5***	**– 0.4**

Table S5 Summary of the calculated free energy of carbene formations ΔG (298 K, 1 atm) at DLPNO-CCSD(T)/CBS//M06-2X-D3/6-31G(d,p) using cc-pV(D-T)Z for X=CF_3, Cl, F, and OMe. The high-level corrected free energy of N_2 is -109.435558328013 E_h. The units of final Gibbs free energies are in E_h, and ΔG are in kJ/mol. These data are used in Fig. 6.

X=CF_3						
σ_p^+	R	diazirine	singlet carbene	triplet carbene	Δ*G* (singlet)	Δ*G* (triplet)
-1.7	NMe_2	-849.7679547	-740.3430655	-740.3361249	-28.0	-9.8
-1.3	NH_2	-771.3194645	-661.8928763	-661.88708	-23.6	-8.3
-0.92	OH	-791.1941893	-681.7627094	-681.7609716	-10.7	-6.1
-0.78	OMe	-830.4123156	-720.9812062	-720.9789479	-11.7	-5.8
-0.31	CH_3	-755.267888	-645.8340032	-645.8347959	-4.4	-6.5
-0.26	*t*-Bu	-872.9716236	-763.5363741	-763.53786	-0.8	-4.7
0	H	-716.0275523	-606.5900275	-606.5940309	5.2	-5.3
0.11	Cl	-1175.212266	-1065.775821	-1065.779023	2.3	-6.1
0.61	CF_3	-1052.856771	-943.4153379	-943.4230487	15.4	-4.8
0.66	CN	-808.163276	-698.7216225	-698.7308207	16.0	-8.1
0.79	NO_2	-920.3539023	-810.9120569	-810.9198491	16.5	-4.0

X=Cl						
σ_p^+	R	diazirine	singlet carbene	triplet carbene	Δ*G* (singlet)	Δ*G* (triplet)
-1.7	NMe_2	-972.1260473	-862.7179391	-862.6964872	-72.1	-15.7
-1.3	NH_2	-893.677363	-784.2686337	-784.2488785	-70.4	-18.6
-0.92	OH	-913.5521539	-804.1411027	-804.1237454	-64.3	-18.8
-0.78	OMe	-952.7705419	-843.3590024	-843.3420274	-63.1	-18.5
-0.31	CH_3	-877.6259946	-768.2113494	-768.1985676	-54.9	-21.3
-0.26	*t*-Bu	-995.3299626	-885.9148193	-885.9015305	-53.6	-18.7
0	H	-838.3853105	-728.9697445	-728.9583101	-52.5	-22.5
0.11	Cl	-1297.570446	-1188.155095	-1188.14348	-53.1	-22.6
0.61	CF_3	-1175.215881	-1065.797208	-1065.788261	-44.3	-20.8
0.66	CN	-930.5217074	-821.1036191	-821.0966442	-45.9	-27.6
0.79	NO_2	-1042.71229	-933.2937615	-933.2860351	-44.7	-24.4

X=F						
σ_p^+	R	diazirine	singlet carbene	triplet carbene	Δ*G* (singlet)	Δ*G* (triplet)
-1.7	NMe_2	-612.1358394	-502.7252733	-502.6894244	-65.6	28.5
-1.3	NH_2	-533.6874531	-424.2763436	-424.2420508	-64.2	25.8
-0.92	OH	-553.5618957	-444.1493504	-444.1170748	-60.4	24.3
-0.78	OMe	-592.7799314	-483.3675455	-483.3354003	-60.8	23.6
-0.31	CH_3	-517.6359311	-408.2202432	-408.1919048	-52.2	22.2
-0.26	*t*-Bu	-635.3394467	-525.9235265	-525.8950691	-51.6	23.2
0	H	-478.3953286	-368.9785601	-368.9521205	-49.3	20.1
0.11	Cl	-937.5801908	-828.1639494	-828.1371375	-50.7	19.7
0.61	CF_3	-815.2250991	-705.8070995	-705.7822079	-46.1	19.3
0.66	CN	-570.5312352	-461.1127491	-461.090625	-44.8	13.3
0.79	NO_2	-682.7217083	-573.3029851	-573.2799927	-44.2	16.2

X=OMe						
σ_p^+	R	diazirine	singlet carbene	triplet carbene	Δ*G* (singlet)	Δ*G* (triplet)
-1.7	NMe_2	-627.3266876	-517.937063	-517.8923537	-120.6	-3.2
-1.3	NH_2	-548.878565	-439.4882194	-439.44495	-118.7	-5.1
-0.92	OH	-568.753901	-459.3627794	-459.3204945	-116.7	-5.6
-0.78	OMe	-607.9718126	-498.5807972	-498.5386439	-116.9	-6.3
-0.31	CH_3	-532.8278622	-423.4343948	-423.3961121	-110.5	-10.0
-0.26	*t*-Bu	-650.5310368	-541.1379472	-541.0985569	-111.5	-8.1
0	H	-493.5873668	-384.1937892	-384.1556017	-110.2	-10.0
0.11	Cl	-952.7727654	-843.3798918	-843.3415037	-112.1	-11.3
0.61	CF_3	-830.4179481	-721.024299	-720.9870833	-110.0	-12.3
0.66	CN	-585.7248119	-476.3309406	-476.2967454	-109.4	-19.7
0.79	NO_2	-697.9154032	-588.5216064	-588.4858059	-109.6	-15.7

Table S6 Summary of the calculated free energy S–T gaps[a] (298 K, 1 atm) of X=CF_3, Cl, F, and OMe carbenes using DLPNO-CCSD(T)/CBS//M06-2X-D3/6-31G(d,p). (D-T)CBS stands for cc-pV(D-T)Z, and (T-Q) stands for cc-pV(T-Q)Z. The units are in kJ/mol. Highlighted cells indicate the calculated singlet–triplet gap (at the two levels of theory noted above) for *para*-methoxyphenyl trifluoromethyl diazirine. Good agreement is found between the two methods, and in each case we find the singlet to be the lower-energy carbene when R=4-OMe and X=CF_3.

σ_p^+	R	X=CF_3		X=Cl	X=F	X=OMe
		(D-T)CBS	(T-Q)CBS	(D-T)CBS	(D-T)CBS	(D-T)CBS
-1.7	NMe_2	-18.2	-19.5	-56.3	-94.1	-117.4
-1.3	NH_2	-15.2	-16.8	-51.9	-90.0	-113.6
-0.92	OH	-4.6	-5.2	-45.6	-84.7	-111.0
-0.78	OMe	-5.9	-6.9	-44.6	-84.4	-110.7
-0.31	CH_3	2.1	1.1	-33.6	-74.4	-100.5
-0.26	*t*-Bu	3.9	2.8	-34.9	-74.7	-103.4
0	H	10.5	9.7	-30.0	-69.4	-100.3
0.11	Cl	8.4	7.8	-30.5	-70.4	-100.8
0.61	CF_3	20.2	19.7	-23.5	-65.4	-97.7
0.66	CN	24.1	22.9	-18.3	-58.1	-89.8
0.79	NO_2	20.5	19.6	-20.3	-60.4	-94.0

[a] A positive S-T gap indicates that the ground state is in a triplet state.

Materials and Methods

General considerations

All commercial materials were used as received. THF was freshly dried over Na/benzophenone. Dichloromethane (DCM) was freshly dried by passage over alumina in a commercial solvent purification system. Anhydrous cyclohexane was used in crosslinking experiments. Spectranalyzed™ pentane was used for purification of diazirines. Unless otherwise stated, all reactions were performed in flame-dried glassware equipped with rubber septa under a positive pressure of argon. NMR spectra were acquired on either a Bruker AVANCE 300 (300.27 MHz for ^{1}H, 282.54 MHz for ^{19}F, 75.5 MHz for ^{13}C) or a Bruker AVANCE Neo 500 (500.27 MHz for ^{1}H, 470.72 MHz for ^{19}F, 125 MHz for ^{13}C) spectrometer. Chemical shifts were reported in parts per million (ppm) and were calibrated to the central peak of residual NMR solvent (central peak of chloroform-d: ^{1}H NMR δ = 7.26 ppm, ^{13}C NMR δ = 77.16 ppm; dichloromethane-d_2: ^{1}H NMR δ = 5.32 ppm, ^{13}C NMR δ = 53.84 ppm). ^{13}C spectra and ^{19}F spectra were ^{1}H decoupled. Data is reported as follows: chemical shift (multiplicity [s = singlet, d = doublet, t = triplet, q = quartet, qd = quartet of doublet, p = pentet, dt = doublet of triplet, tt = triplet of triplet, td = triplet of doublet, br-s = broad singlet, m = multiplet], coupling constant in Hz, integration). Chemical shifts in ^{19}F spectra are reported in ppm and reported as obtained. IR spectra were recorded using a Perkin-Elmer ATR spectrometer. IR wave numbers (*v*) are reported in cm^{-1}. Rayonet UV chamber equipped with eight 350 nm UV lamps and an operating fan was used for photochemical C–H insertion experiments. High resolution electrospray ionization mass spectrometry (HRMS) data were acquired using a Thermo Scientific Orbitrap Exactive Plus spectrometer using electrospray ionization experiments. GC-MS experiments were conducted using a Finnigan Trace GC Ultra and DSQ mass spectrometer system coupled with an AI 3000 autosampler (Thermo, MA). Samples were dissolved in Optima™ Acetonitrile (Fisher Chemical, NH) and then 2 µL was injected into the GC with a carrier flow of He gas set at 1 mL/min. The column used was a RTX-5MS capillary column with dimensions of 15 m x 0.25 mm x 0.5 µm (Restek, PA). The inlet temperature was set to 220°C with a split flow of 10 mL/min. The oven temperature was held at 50°C for 1 min and then raised to 250°C using a ramp rate of 20°C/min. The oven then was held for three minutes at 250°C resulting in a total run time of 9 min. The DSQ mass spectrometer was scanned continuously in positive mode in the range of m/z = 50 to 650 Da. The source temperature was set to 150°C and electron impact energy 70 eV. Differential scanning calorimetry analysis was performed using a DSC 25 TA instrument.

All diazirine-forming reactions were performed in the dark. Removal of solvent was done at 25°C, avoiding the use of excessive vacuum.

Diazirines are numbered in order of appearance in the manuscript **D1**–**D17**.

Synthesis of aryl diazirines

Diazirine **D4** was purchased from Amadis Chemical and diazirines **D6** and **D7** were purchased from TCI America.

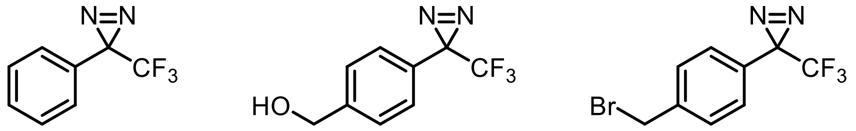

D4 **D6** **D7**

Synthesis of 2,2,2-trifluoro-1-(4-methoxyphenyl)ethan-1-one (S1).

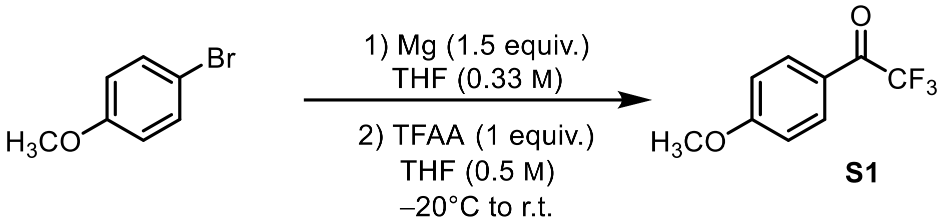

To a suspension of magnesium turnings (182 mg, 7.5 mmol, 1.5 equiv.) in anhydrous THF (7.5 mL) stirred under argon was added drop wise a solution of 4-bromoanisole (0.6 mL, 5 mmol, 1 equiv.) in anhydrous THF (7.5 mL). The reaction was left for 2 h stirring and the product, (4-methoxyphenyl)magnesium bromide (14 mL of 0.33 M solution in THF fresh-made, 4.62 mmol) was added drop wise to a solution of trifluoroacetic anhydride (TFAA, 0.64 mL, 4.2 mmol, 1 equiv.) in anhydrous THF at –20 °C. The reaction was stirred at –20 °C for 2 h, then it was quenched with NH_4Cl and it was extracted with diethyl ether (× 3) and the combined organic layers were washed with brine and dried over magnesium sulfate. The crude compound **S1** was purified by silica gel column chromatography using petroleum ether: EtOAc (9:1, R_f = 0.35) as eluent to afford the desired ketone **S1** (713 mg, 3.49 mmol, 83%) as a yellow solid with spectroscopic data in accordance with the literature.[8] ^{1}H NMR (300 MHz, $CDCl_3$) δ 8.06 (dd, *J* = 9.1, 1.0 Hz, 2H), 7.01 (d, *J* = 9.1 Hz, 2H), 3.92 (s, 3H). ^{19}F NMR (283 MHz, $CDCl_3$) δ –71.01.

Synthesis of 2,2,2-trifluoro-1-(4-methoxyphenyl)ethan-1-one oxime (S2) (mixture of *E*-, *Z*-isomers).

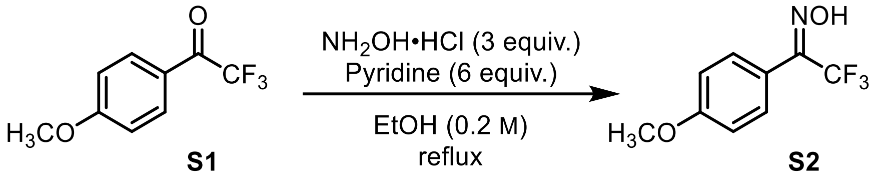

To a stirred solution of compound **S1** (713 mg, 3.49 mmol, 1 equiv.) in ethanol (0.2 M), hydroxylamine hydrochloride (728 mg, 10.5 mmol, 3 equiv.) and pyridine (1.7 mL, 20.9 mmol, 6 equiv.) were added and the reaction mixture was heated to reflux for 21 h. The mixture was then cooled to room temperature and the mixture was treated with 2 M HCl and extracted with diethyl ether (3 times). The combined organic layers were washed with distilled water until the pH of the washing layer became neutral, and then dried with sodium sulfate, filtered, and concentrated. The residue was dried under high vacuum for a prolonged time to afford the desired crude oxime **S2** (as a mixture of geometric isomers) as a white solid, with spectroscopic data in accordance with the literature.[8] The compound was submitted to the next step without further purification. ^{1}H NMR (300 MHz, $CDCl_3$) δ 8.36 (br-s, 1H, OH), 8.12 – 8.01 (m, 0.3H, OH, minor isomer), 7.53 (d, *J* = 8.7 Hz, 2H), 7.44 (dd, *J* = 9.0, 1.0 Hz, 0.75H, minor isomer), 6.98 (d, *J* = 9.0 Hz, 2H), 6.95 – 6.90 (m, 0.75H, minor isomer), 3.85 (s, 3H), 3.84 (s, 1.3H, minor isomer). ^{19}F NMR (283 MHz, $CDCl_3$) δ –62.36 (minor isomer), –66.31.

Synthesis of 2,2,2-trifluoro-1-(4-methoxyphenyl)ethan-1-one *O*-tosyl oxime (S3) (mixture of *E*-, Z-isomers).

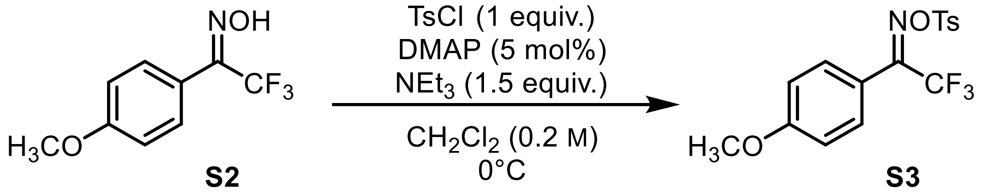

Compound **S2** (764 mg, 3.49 mmol, 1 equiv.) was dissolved in CH_2Cl_2 (0.2 M), and triethylamine (0.7 mL, 5.2 mmol, 1.5 equiv.), DMAP (21 mg, 0.17 mmol, 5 mol%) and tosyl chloride (665 mg, 3.49 mmol, 1 equiv.) were successively added at 0°C. The ice bath was removed after 5 min, and the reaction mixture was stirred at room temperature for 1 h. The mixture was then treated with sat. aq. NH_4Cl and extracted with CH_2Cl_2. The combined organic extracts were dried with sodium sulfate, filtered, and concentrated to afford the desired crude tosyl oxime **S3** as a white solid, with spectroscopic data in accordance with the literature.[8] The compound was submitted to the next step without further purification. ^1H NMR (300 MHz, $CDCl_3$) δ 7.89 (d, *J* = 8.3 Hz, 2H), 7.45 (d, *J* = 8.9 Hz, 2H), 7.38 (d, *J* = 8.2 Hz, 2H), 6.96 (d, *J* = 9.0 Hz, 2H), 3.86 (s, 3H), 2.48 (s, 3H). ^{19}F NMR (283 MHz, $CDCl_3$) δ –61.42 (minor isomer), –66.03.

Synthesis of 3-(4-methoxyphenyl)-3-(trifluoromethyl)diaziridine (S4).

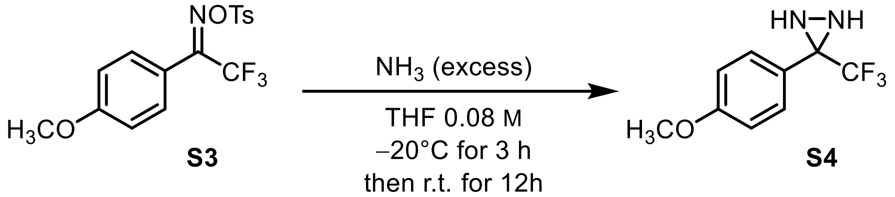

Tosyl oxime **S3** (856 mg, 2.29 mmol, 1 equiv.) was transferred to a flame-dried 3-neck flask under argon in anhydrous THF (0.08 M) and cooled to –20°C. Anhydrous gaseous ammonia was bubbled into the stirred solution for 3 h. Then, the reaction was left stirring for 12 h, during which time it was allowed to warm from –20°C to room temperature. The mixture was quenched with sat. aq. NH_4Cl and extracted with diethyl ether (3 times). The combined organic layers were washed with brine and then dried with sodium sulfate, filtered, and concentrated to afford the desired crude diaziridine **S4** as a colourless solid, which was submitted to the next step without further purification. The analytical data were in accordance with literature.[8] ^1H NMR (300 MHz, $CDCl_3$) δ 7.53 (d, *J* = 8.7 Hz, 2H), 6.93 (d, *J* = 8.7 Hz, 2H), 3.83 (s, 3H), 2.75 (d, *J* = 8.6 Hz, 1H, NH), 2.16 (d, *J* = 8.6 Hz, 1H, NH). ^{19}F NMR (283 MHz, $CDCl_3$) δ –75.81.

Synthesis of 3-(4-methoxyphenyl)-3-(trifluoromethyl)-3*H*-diazirine (D1).

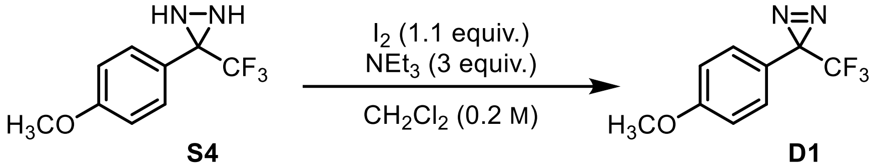

To a solution of the crude diaziridine **S4** (298 mg, 1.37 mmol, 1 equiv.) in CH_2Cl_2 (0.2 M) at 0 °C were added successively triethylamine (0.57 mL, 4.11 mmol, 3 equiv.) and iodine (382 mg, 1.5 mmol, 1.1 equiv.). The colored mixture was stirred at 0°C for 1 h. The mixture was diluted with CH_2Cl_2 and washed with sat. aq. sodium thiosulfate. The aqueous layer was re-extracted with CH_2Cl_2 (3 times). Then the combined organic extracts were washed with brine and dried with sodium sulfate, filtered, and concentrated. The residue was purified by silica gel column chromatography using pentane: diethyl ether (9:1, R_f= 0.33) as eluent to afford the desired diazirine **D1** (291 mg, 1.34 mmol, 98%) as a yellow oil, with spectroscopic data in accordance with the literature.[8] ^1H NMR (500 MHz, $CDCl_3$) δ 7.15 (dd, *J* = 9.1, 0.7 Hz, 2H), 6.91 (d, *J* = 9.0 Hz, 2H), 3.81 (s, 3H).^{13}C NMR (126 MHz, $CDCl_3$) δ 160.78, 128.30, 122.41 (q, *J* = 274.6 Hz), 121.09, 114.51, 55.51. ^{19}F NMR (283 MHz, $CDCl_3$) δ –65.63. UV (*n*-hexane): $\lambda_{max,\ diazirine}$ = 372 nm. IR (diamond-ATR) ν: 2937, 2844, 1606, 1514, 1297, 1261, 1176, 1133, 1032, 969, 835.

Fig. S6 ^{1}H NMR spectrum of **D1** in $CDCl_3$.

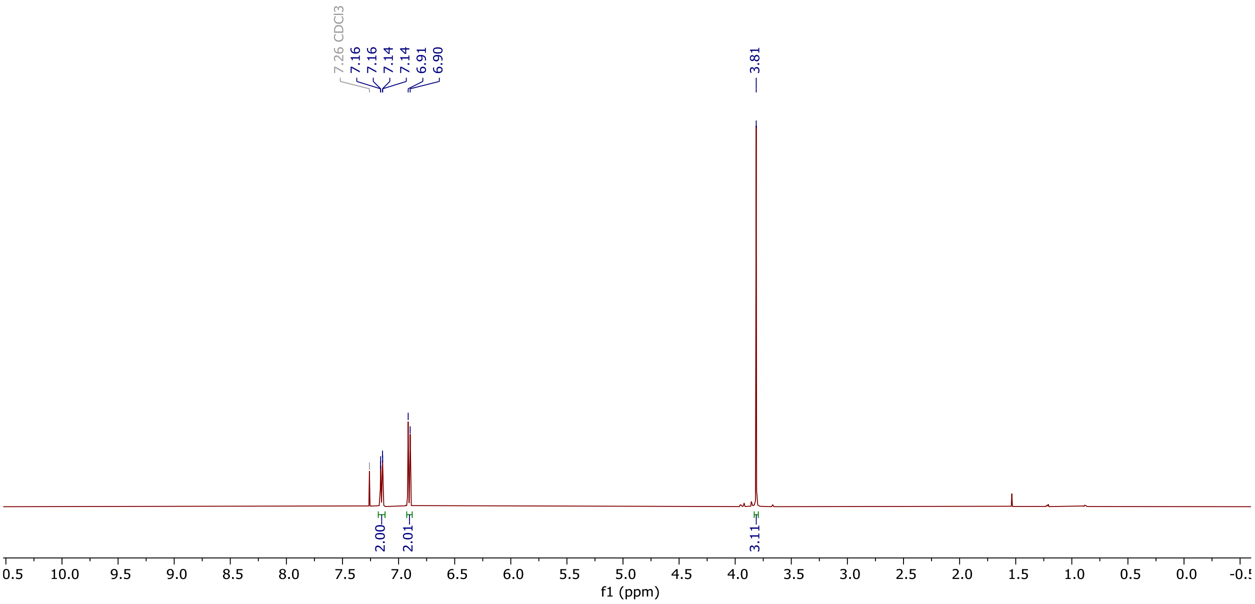

Fig. S7 ^{13}C NMR spectrum of **D1** in $CDCl_3$.

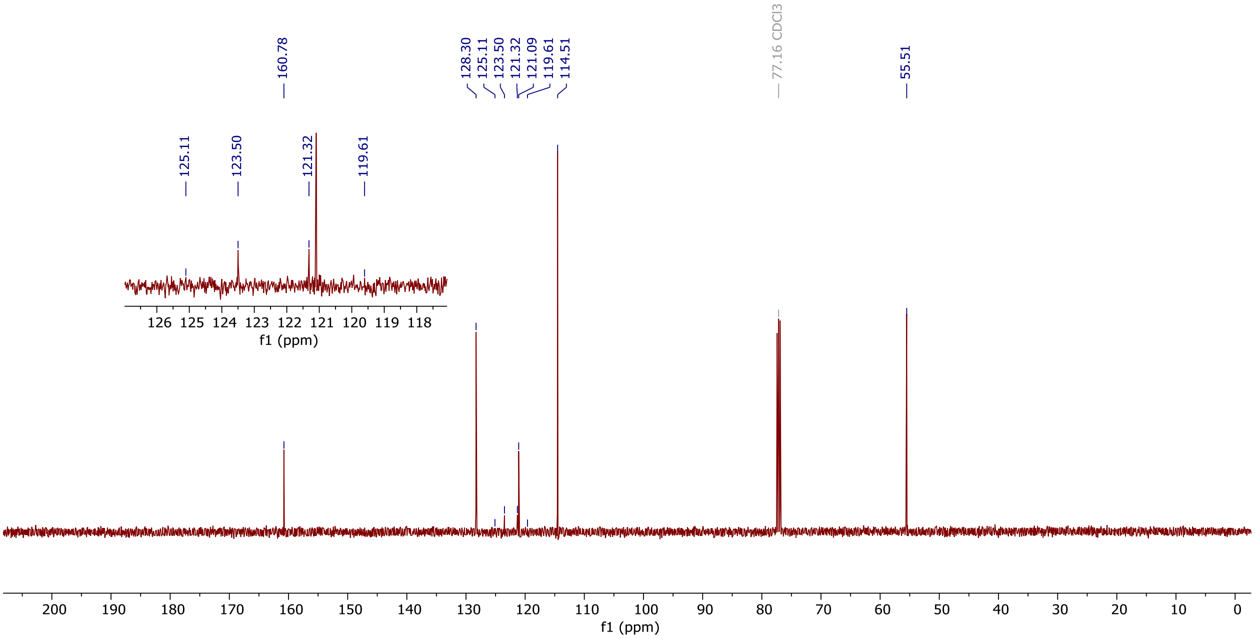

Fig. S8 ^{19}F NMR spectrum of **D1** in $CDCl_3$.

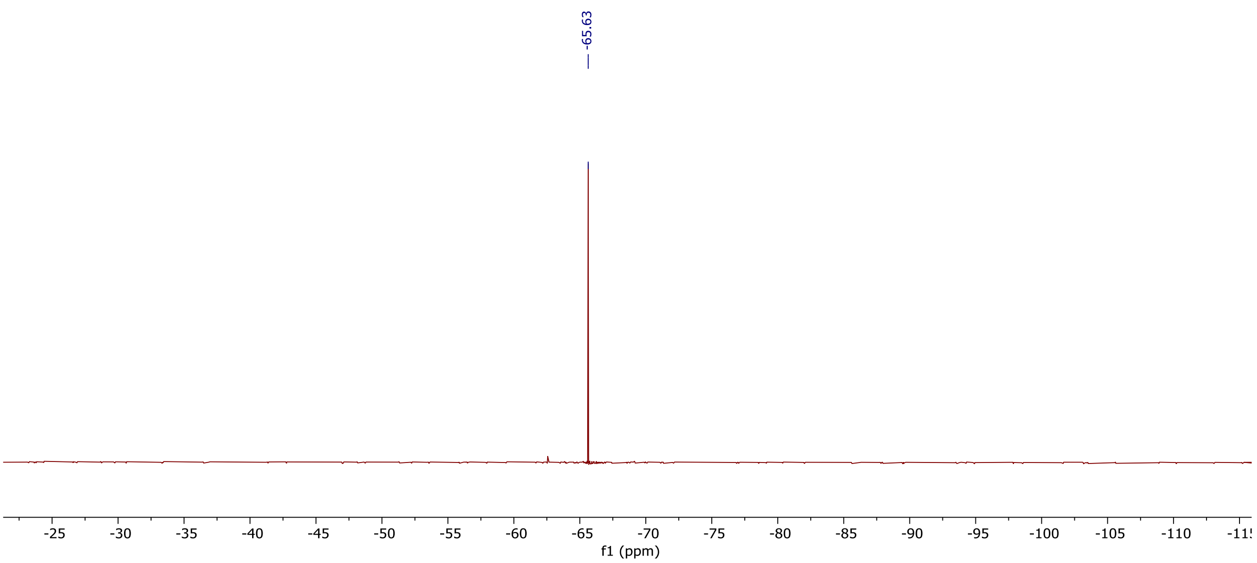

Synthesis of 2,2,2-trifluoro-1-(4-phenoxyphenyl)ethan-1-one (S5).

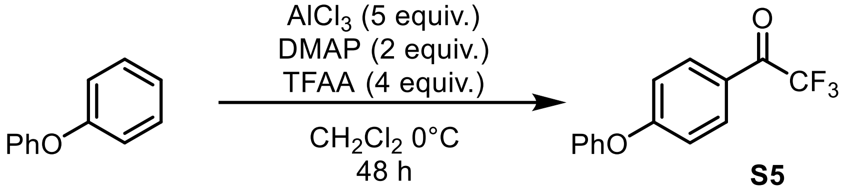

To a solution of diphenyl ether (1 g, 5.87 mmol, 1 equiv.) and 4-dimethylaminopyridine (DMAP, 1.4 g, 11.7 mmol, 2 equiv.) in dry dichloromethane (10 mL), trifluoroacetic anhydride (TFAA, 3.2 mL, 23.4 mmol, 4 equiv.) was injected dropwise at 0 °C under argon atmosphere. Then, anhydrous aluminum chloride (3.9 g, 29.35 mmol, 5 equiv.) was added to the mixture. The reaction was warmed to room temperature and stirred for 48 h. The reaction mixture was poured into ice water and extracted with dichloromethane. The organic layer was washed with brine and dried over sodium sulfate. After evaporation of the solvent the reaction afforded the desired pure product **S5** as a colorless oil (1.25 g, 4.69 mmol, 80%) in agreement with literature data.[9] ^{1}H NMR (300 MHz, $CDCl_3$) δ 8.05 (d, *J* = 8.2 Hz, 2H), 7.44 (t, *J* = 7.9 Hz, 2H), 7.29 – 7.21 (m, 1H), 7.10 (d, *J* = 7.6 Hz, 2H), 7.04 (d, *J* = 9.0 Hz, 2H). ^{19}F NMR (283 MHz, $CDCl_3$) δ –71.15.

Synthesis of 2,2,2-trifluoro-1-(4-phenoxyphenyl)ethan-1-one oxime (S6) (mixture of *E*-, *Z*-isomers).

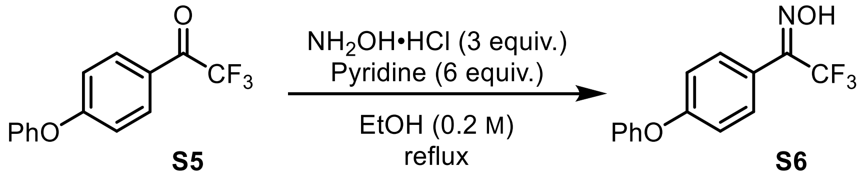

To a stirred solution of compound **S5** (443 mg, 1.66 mmol, 1 equiv,) in ethanol (0.2 M), hydroxylamine hydrochloride (1.15 g, 16.5 mmol, 3 equiv.) and pyridine (2.6 mL, 33 mmol, 6 equiv.) were added and the reaction mixture was heated to reflux for 21 h. The mixture was then cooled to room temperature and the mixture was treated with 2 M HCl and extracted with diethyl ether (3 times). The combined organic layers were washed with distilled water until the pH of the washing layer became neutral, and then dried with sodium sulfate, filtered, and concentrated. The residue was dried under high vacuum for a prolonged time to afford the desired crude oxime **S6** (as a mixture of geometric isomers) as a yellow oil (464 mg). The compound was submitted to the next step without further purification. ^{1}H NMR (300 MHz, $CDCl_3$) δ 8.30 (d, *J* = 8.9 Hz, 2H), 8.25 (d, *J* = 9.0 Hz, 1.6H, minor isomer), 7.68 (d, *J* = 8.6 Hz, 3.6H, major and minor isomers). ^{19}F NMR (283 MHz, $CDCl_3$) δ –62.28, –66.06.

Synthesis of 2,2,2-trifluoro-1-(4-phenoxyphenyl)ethan-1-one *O*-tosyl oxime (S7) (mixture of *E*-, *Z*-isomers).

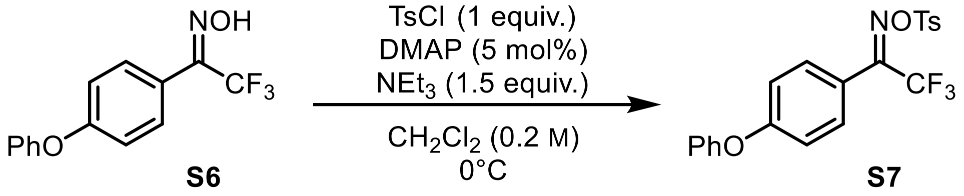

Compound **S6** (464 mg, 1.65 mmol, 1 equiv.) was dissolved in CH_2Cl_2 (0.2 M), and triethylamine (0.35 mL, 2.47 mmol, 1.5 equiv.), DMAP (10 mg, 0.082 mmol, 5 mol%) and tosyl chloride (315 mg, 1.65 mmol, 1 equiv.) were successively added at 0°C. The ice bath was removed after 5 min, and the reaction mixture was stirred at room temperature for 1 h. The mixture was then treated with sat. aq. NH_4Cl and extracted with CH_2Cl_2. The combined organic extracts were dried with sodium sulfate, filtered, and concentrated to afford the desired crude tosyl oxime **S7** as a yellow solid, which was submitted to the next step without further purification. ^{1}H NMR (300 MHz, $CDCl_3$) δ 7.89 (d, *J* = 8.3 Hz, 2.90H, major and minor isomers), 7.48 – 7.33 (m, 9H, major and minor isomers), 7.24 – 7.16 (m, 1.55H, minor isomer), 7.14 – 6.92 (m, 6H, major and minor isomers), 2.48 (s, 3H), 2.46 (s, 1.39H, minor isomer). ^{19}F NMR (283 MHz, $CDCl_3$) δ –61.45 (minor isomer), –66.26.

Synthesis of 3-(4-phenoxyphenyl)-3-(trifluoromethyl)diaziridine (S8).

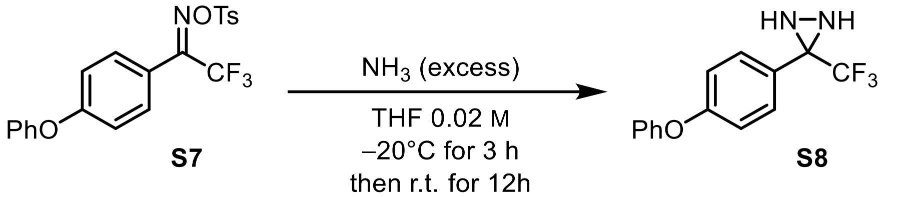

Tosyl oxime **S7** (714 mg, 1.60 mmol, 1 equiv.) was transferred to a flame-dried 3-neck flask under argon in anhydrous THF (0.02 M) and cooled to –20°C. Anhydrous gaseous ammonia was bubbled into the stirred solution for 3 h. Then, the reaction was left stirring for 12 h, during which time it was allowed to warm from –20°C to room temperature. The mixture was quenched with sat. aq. NH_4Cl and extracted with diethyl ether (3 times). The combined organic layers were washed with brine and then dried with sodium sulfate, filtered and concentrated to afford the desired crude diaziridine **S8**, which was submitted to the next step without further purification.^{19}F NMR (283 MHz, $CDCl_3$) δ –75.69.

Synthesis of 3-(4-phenoxyphenyl)-3-(trifluoromethyl)-3*H*-diazirine (D2).

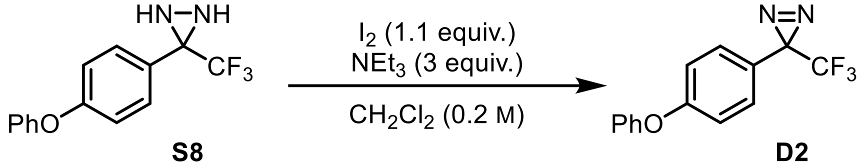

To a solution of the crude diaziridine **S8** in CH_2Cl_2 (0.2 M) at 0 °C were added successively triethylamine (3 equiv.) and iodine (1.1 equiv.). The colored mixture was stirred at 0°C for 1 h. The mixture was diluted with CH_2Cl_2 and washed with sat. aq. sodium thiosulfate. The aqueous layer was re-extracted with CH_2Cl_2 (3 times). Then the combined organic extracts were washed with brine and dried with sodium sulfate, filtered, and concentrated. The residue was purified by silica gel column chromatography using pentane as eluent (R_f = 0.44) to afford the desired diazirine **D2** (216 mg, 0.77 mmol, 48% over two steps) as a pale-yellow oil. ^{1}H NMR (500 MHz, $CDCl_3$) δ 7.40 – 7.33 (m, 2H), 7.19 – 7.12 (m, 3H), 7.04 – 7.01 (m, 2H), 7.01 – 6.97 (m, 2H). ^{13}C NMR (126 MHz, $CDCl_3$) δ 159.09, 156.11, 130.14, 128.45, 124.40, 123.34, 119.83, 118.56, 29.86. ^{19}F NMR (471 MHz, $CDCl_3$) δ –65.52. UV (*n*-hexane): $\lambda_{max,\ diazirine}$ = 369 nm. IR (diamond-ATR) ν: 3044, 2927, 2856, 1614, 1589, 1512, 1489, 1344, 1232, 1186, 1148, 937, 691. GC–MS *m/z* (% relative intensity) [ion]: 291 (70) $[M - N_2 + CH_3CN]^+$, 250 (40) $[M - N_2]^+$, 157 (100) $[C_8H_4F_3]^+$, 137 (10) $[C_8H_3F_2]^+$, 76 (45) $[C_6H_4]^+$. [M = $C_{14}H_9F_3N_2O$ (278.06)].

Fig. S9 ^{1}H NMR spectrum of **D2** in $CDCl_3$.

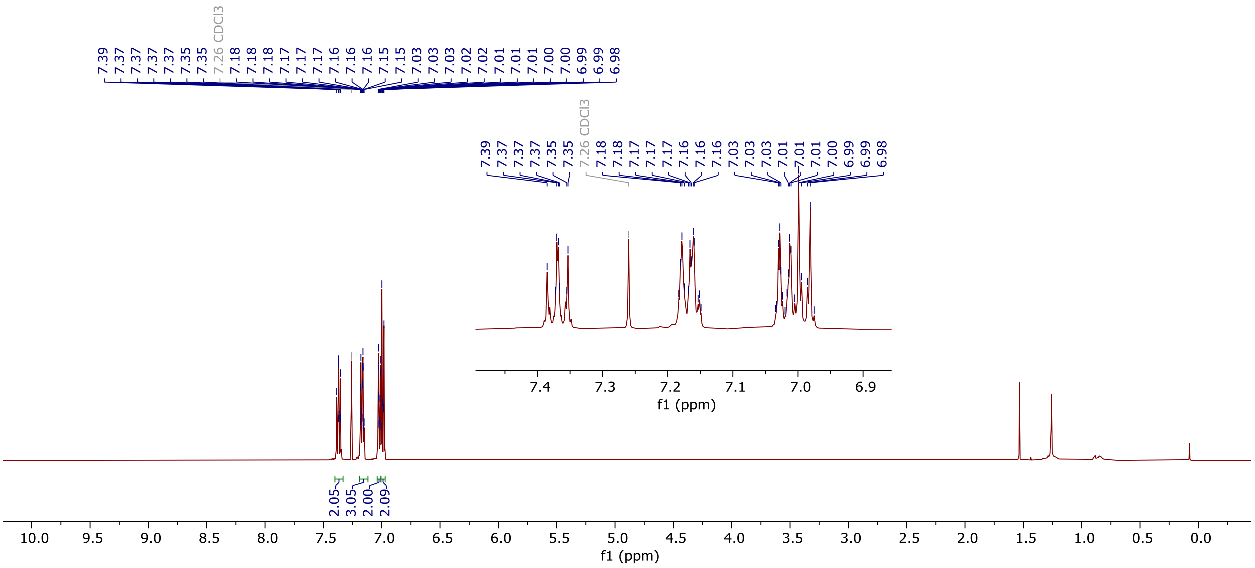

Fig. S10 ^{13}C NMR spectrum of **D2** in $CDCl_3$.

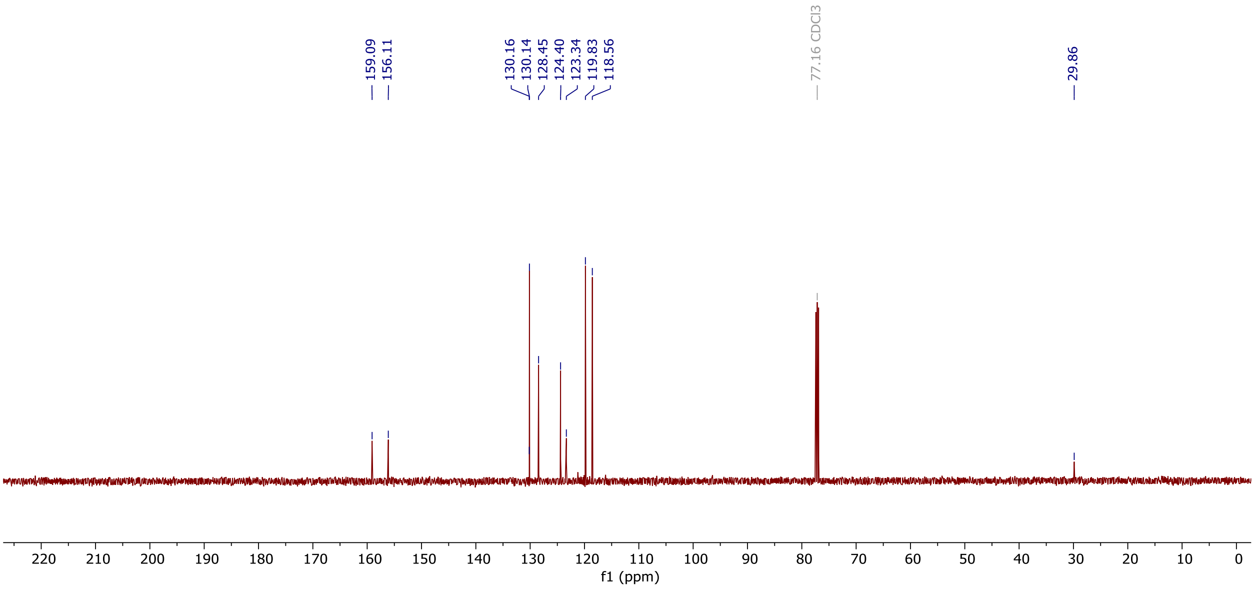

Fig. S11 ^{19}F NMR spectrum of **D2** in $CDCl_3$.

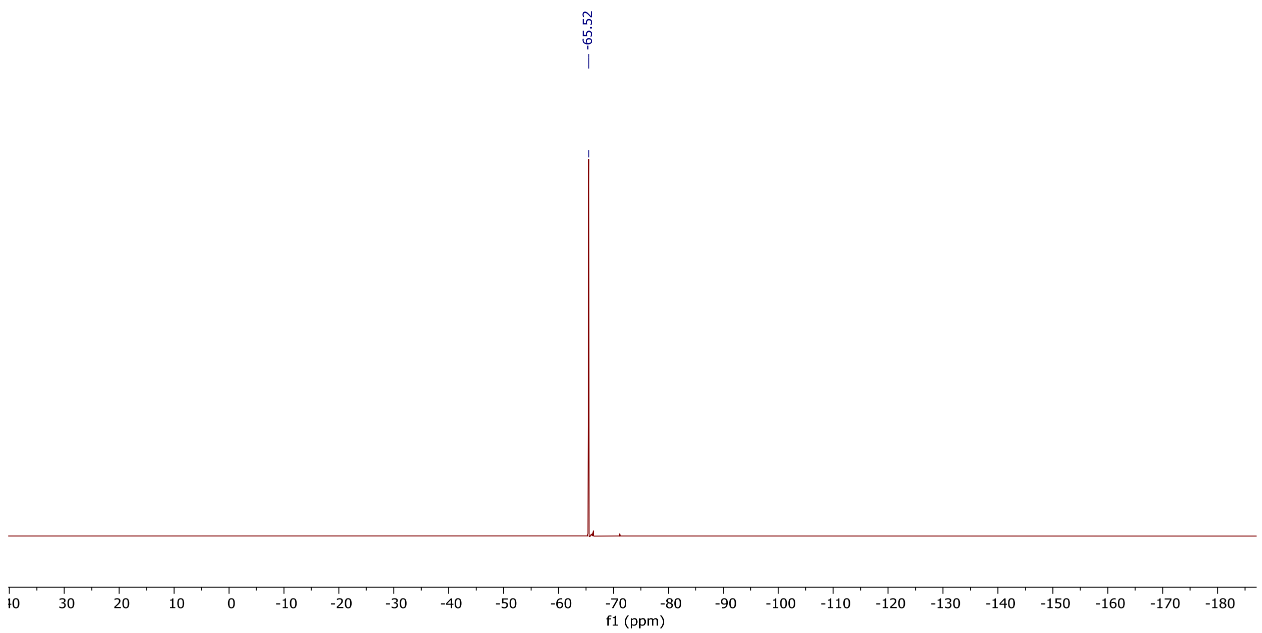

Synthesis of 1-(4-(tert-butyl)phenyl)-2,2,2-trifluoroethan-1-one (S9).

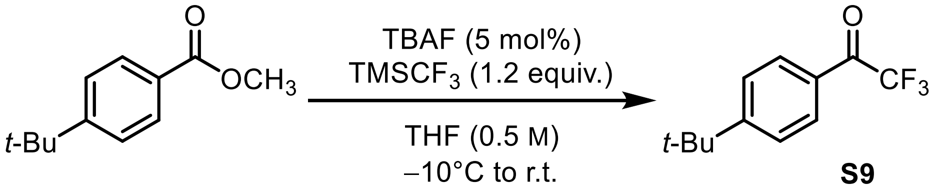

In a flame-dried flask, to a stirring solution of commercially available methyl 4-(tert-butyl)benzoate (1.0 mL, 5.2 mmol, 1 equiv.) in distilled THF (8.2 mL) under argon atmosphere at –10 °C, $TMSCF_3$ (0.92 mL, 6.24 mmol, 1.2 equiv.) and then TBAF (0.26 mL, 1 M in THF, 5 mol%) were slowly added dropwise. The resulting solution was allowed to warm to room temperature with continued stirring for an hour and the colour changed from transparent to orange. The reaction was left stirring for 16 h and then quenched with NH_4Cl, extracted with diethyl ether (2 times) and the combined organic extracts were washed with brine and dried with sodium sulfate. The filtered and concentrated orange oil was purified by silica gel column chromatography using pentane: chloroform (6:4, R_f = 0.62) as eluent to afford the desired ketone **S9** (446 mg, 1.94 mmol, 37%) as a yellow oil, in accordance with literature data.[10] ^1H NMR (300 MHz, $CDCl_3$) δ 8.02 (d, *J* = 8.8 Hz, 2H), 7.56 (d, *J* = 8.8 Hz, 2H), 1.36 (s, 9H). ^{19}F NMR (283 MHz, $CDCl_3$) δ –71.34.

Synthesis of 1-(4-(tert-butyl)phenyl)-2,2,2-trifluoroethan-1-one oxime (S10) (mixture of *E*-, *Z*-isomers).

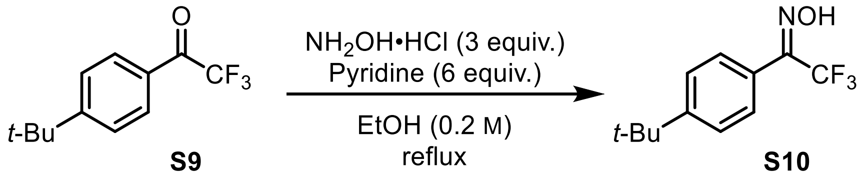

To a stirred solution of compound **S9** (400 mg, 1.74 mmol, 1 equiv,) in ethanol (0.2 M), hydroxylamine hydrochloride (363 mg, 5.22 mmol, 3 equiv.) and pyridine (0.9 mL, 10.42 mmol, 6 equiv.) were added and the reaction mixture was heated to reflux for 21 h. The mixture was then cooled to room temperature and the mixture was treated with 2 M HCl and extracted with diethyl ether (3 times). The combined organic layers were washed with distilled water until the pH of the washing layer became neutral, and then dried with sodium sulfate, filtered, and concentrated. The residue was dried under high vacuum for a prolonged time to afford the desired crude oxime **S10** (as a mixture of geometric isomers) as a white solid (410 mg, 96%). The compound was submitted to the next step without further purification. ^1H NMR (300 MHz, $CDCl_3$) δ7.47 (d, *J* = 2.3 Hz, 2H), 7.43 (s, 2H), 1.33 (s, 9H). ^{19}F NMR (283 MHz, $CDCl_3$) δ –62.36 (minor isomer), –66.44.

Synthesis of 1-(4-(tert-butyl)phenyl)-2,2,2-trifluoroethan-1-one *O*-tosyl oxime (S11) (mixture of *E*-, *Z*-isomers).

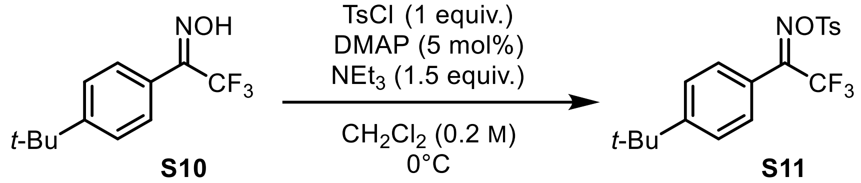

Compound **S10** (410 mg, 1.67 mmol, 1 equiv.) was dissolved in CH_2Cl_2 (0.8 mL), and triethylamine (0.35 mL, 2.5 mmol, 1.5 equiv.), DMAP (10 mg, 0.083 mmol, 5 mol%) and tosyl chloride (319 mg, 1.67 mmol, 1 equiv.) were successively added at 0°C. The ice bath was removed after 5 min, and the reaction mixture was stirred at room temperature for 1 h. The mixture was then treated with sat. aq. NH_4Cl and extracted with CH_2Cl_2. The combined organic extracts were dried with magnesium sulfate, filtered, and concentrated to afford the desired crude tosyl oxime **S11** (587 mg, 91%) as a yellow oil, which was submitted to the next step without further purification. ^{1}H NMR (300 MHz, $CDCl_3$) δ 7.95 – 7.84 (m, 2H), 7.80 (d, *J* = 8.3 Hz, 2H), 7.48 (d, *J* = 8.8 Hz, 2H), 7.42 – 7.29 (m, 2H), 2.45 (s, 3H), 1.34 (s, 9H). ^{19}F NMR (283 MHz, $CDCl_3$) δ –61.49 (minor isomer), –66.46.

Synthesis of 3-(4-(tert-butyl)phenyl)-3-(trifluoromethyl)diaziridine (S12).

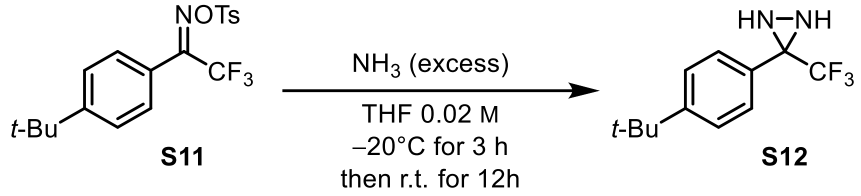

Tosyl oxime **S11** (533 mg, 1.3 mmol, 1 equiv.) was transferred to a flame-dried 3-neck flask under argon in anhydrous THF (65 mL) and cooled to –20°C. Anhydrous gaseous ammonia was bubbled into the stirred solution for 3 h. Then, the reaction was left stirring for 12 h, during which time it was allowed to warm from –20°C to room temperature. The mixture was quenched with sat. aq. NH_4Cl and extracted with CH_2Cl_2 (3 times). The combined organic layers were washed with brine and then dried with sodium sulfate, filtered and concentrated to afford the desired crude diaziridine **S12**, which was submitted to the next step without further purification. ^{1}H NMR (300 MHz, $CDCl_3$) δ 7.54 (d, *J* = 8.3 Hz, 2H), 7.43 (d, *J* = 8.7 Hz, 2H), 2.76 (d, *J* = 9.1 Hz, 1H), 2.19 (d, *J* = 9.1 Hz, 1H), 1.32 (s, 9H). ^{19}F NMR (283 MHz, $CDCl_3$) δ –75.59.

Synthesis of 3-(4-(tert-butyl)phenyl)-3-(trifluoromethyl)-3*H*-diazirine (D3).

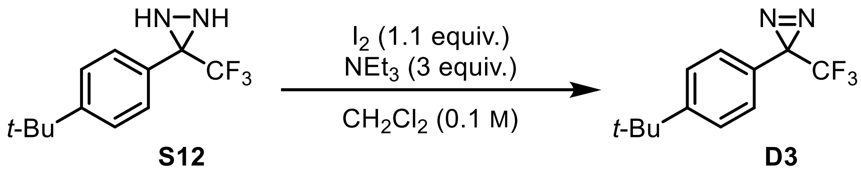

To a solution of the crude diaziridine **S12** (318 mg, 1.3 mmol, 1 equiv.) in CH_2Cl_2 (10 mL) at 0 °C were added successively triethylamine (0.54 mL, 3.9 mmol, 3 equiv.) and iodine (363 mg, 1.43 mmol, 1.1 equiv.). The colored mixture was stirred at 0°C for 1 h. The mixture was diluted with CH_2Cl_2 and washed with sat. aq. sodium thiosulfate. The aqueous layer was re-extracted with CH_2Cl_2 (3 times). Then the combined organic extracts were washed with brine and dried with sodium sulfate, filtered, and concentrated. The residue was purified by silica gel column chromatography using pentane as eluent to afford the desired diazirine **D3** (282 mg, 1.16 mmol, 80%) as a pale-yellow oil. ^{1}H NMR (500 MHz, $CDCl_3$) δ 7.41 (d, *J* = 8.6 Hz, 2H), 7.13 (d, *J* = 8.1 Hz, 2H), 1.31 (s, 9H). ^{13}C NMR (126 MHz, $CDCl_3$) δ 153.08, 126.36, 126.32, 125.96, 122.38 (q, *J* = 274.7, 274.3 Hz), 34.88, 31.27, 29.87. ^{19}F NMR (283 MHz, $CDCl_3$) δ –65.30. UV (*n*-hexane): $\lambda_{max,\,diazirine}$ = 360 nm. IR (diamond-ATR) ν: 2960, 2925, 2855, 1616, 1514, 1464, 1344, 1238, 1188, 1158, 941, 703. HRMS (ESI-) m/z [M – N_2 + HO^-] calculated for $[C_{12}H_{14}F_3O]^-$: 231.1002, found: 231.1006.

Fig. S12 ^{1}H NMR spectrum of **D3** in $CDCl_3$.

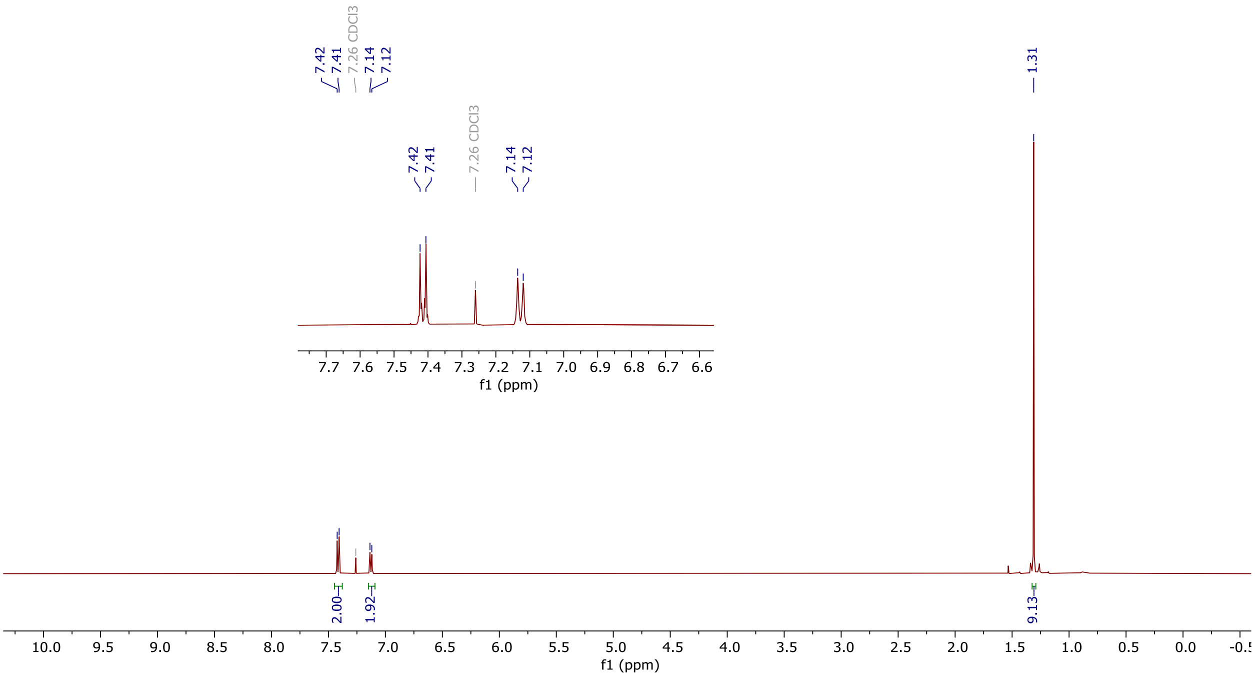

Fig. S13 ^{13}C NMR spectrum of **D3** in $CDCl_3$.

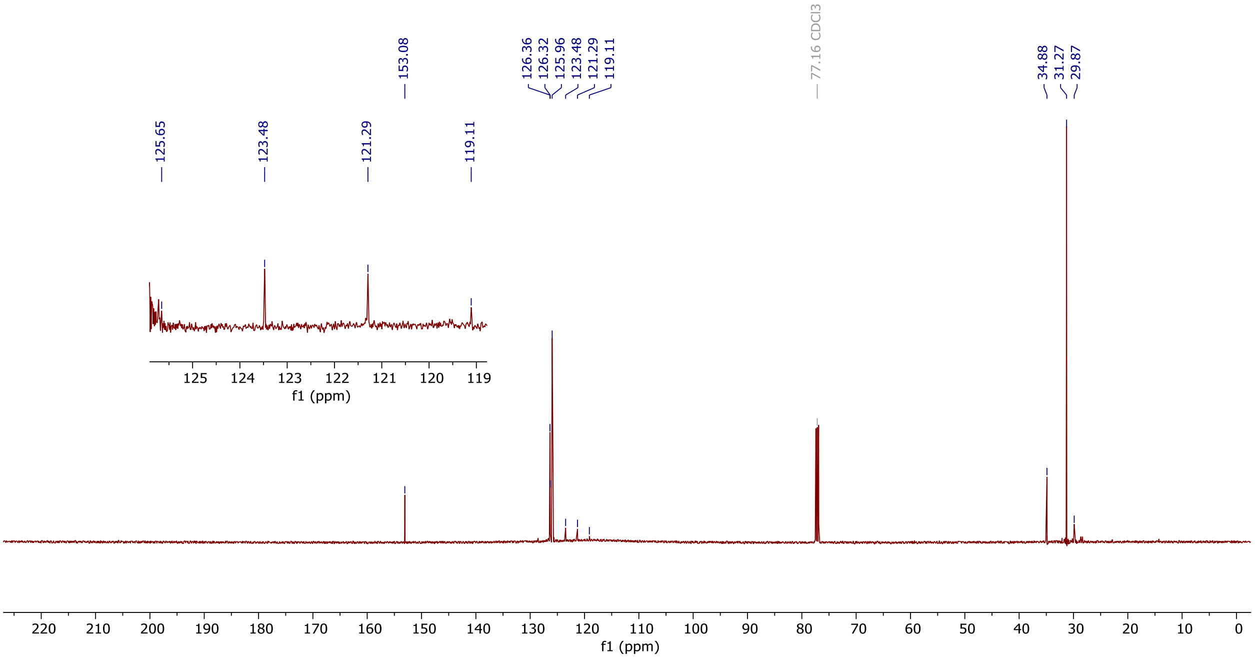

Fig. S14 ^{19}F NMR spectrum of **D3** in $CDCl_3$.

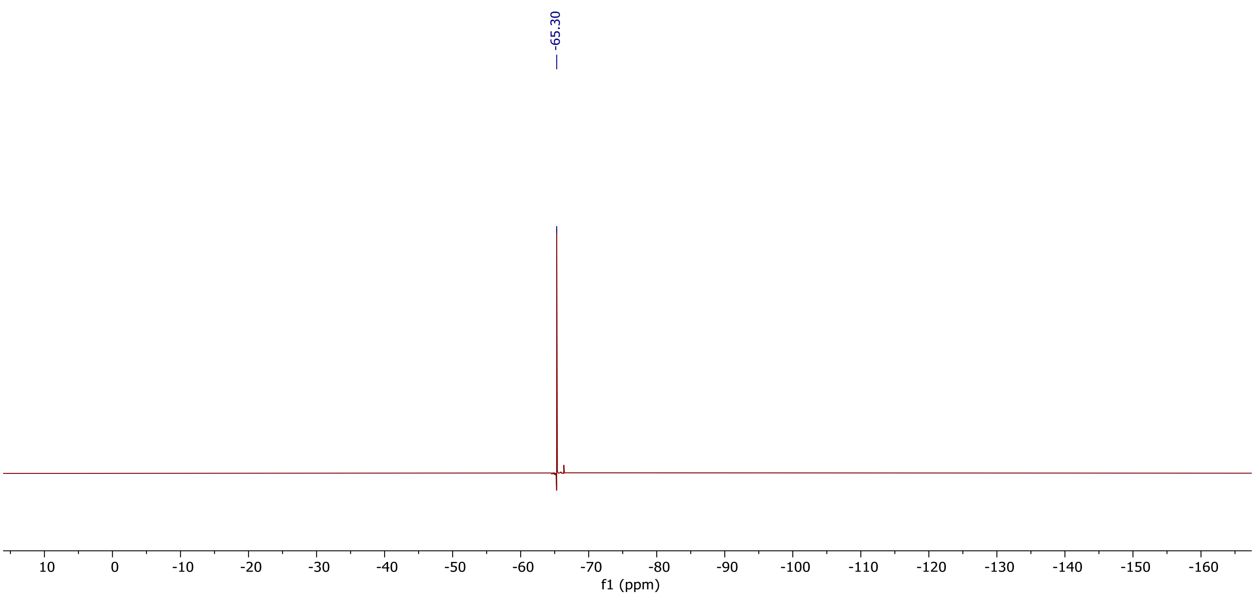

Synthesis of 1-(4-bromophenyl)-2,2,2-trifluoroethan-1-one (S13).

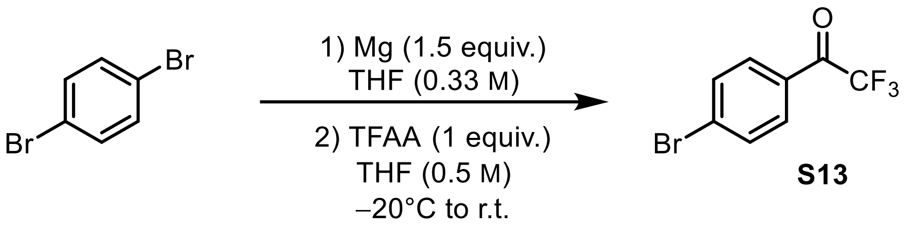

To a suspension of magnesium turnings (250 mg, 10.4 mmol, 1.5 equiv.) in anhydrous THF (11 mL) stirred under argon was added drop wise a solution of 1,4-dibromobenzene (1.63 g, 6.9 mmol, 1 equiv.) in anhydrous THF (9 mL). The reaction was left for 2 h stirring and the product, (4-bromophenyl)magnesium bromide (16 mL of 0.33 M solution in THF fresh-made, 5.4 mmol) was added drop wise to a solution of trifluoroacetic anhydride (TFAA, 0.83 mL, 5 mmol, 1 equiv.) in anhydrous THF at –20 °C. The reaction was stirred at –20 °C for 2 h, then it was quenched with NH_4Cl and it was extracted with diethyl ether (× 3) and the combined organic layers were washed with brine and dried over magnesium sulfate. The crude compound **S13** was purified by silica gel column chromatography using petroleum ether: EtOAc (9:1, R_f = 0.40) as eluent to afford the desired ketone **S13** (898 mg, 3.55 mmol, 71%) as a pale-yellow oil with spectroscopic data in accordance with the literature.[11] ^{1}H NMR (300 MHz, $CDCl_3$) δ 7.93 (d, J = 8.7 Hz, 2H), 7.71 (d, J = 8.7 Hz, 2H). ^{19}F NMR (283 MHz, $CDCl_3$) δ –71.54.

Synthesis of 1-(4-bromophenyl)-2,2,2-trifluoroethan-1-one oxime (S14) (mixture of *E*-, *Z*-isomers).

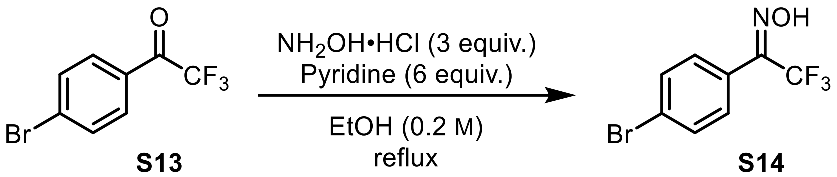

To a stirred solution of compound **S13** (815 mg, 3.22 mmol, 1 equiv.) in ethanol (0.2 M), hydroxylamine hydrochloride (672 mg, 9.66 mmol, 3 equiv.) and pyridine (1.5 mL, 19.3 mmol, 6 equiv.) were added and the reaction mixture was heated to reflux for 21 h. The mixture was then cooled to room temperature and the mixture was treated with 2 M HCl and extracted with diethyl ether (3 times). The combined organic layers were washed with distilled water until the pH of the washing layer became neutral, and then dried with sodium sulfate, filtered, and concentrated. The residue was dried under high vacuum for a prolonged time to afford the desired crude oxime **S14** (as a mixture of geometric isomers) as a yellow oil, with spectroscopic data in accordance with the literature.[12] The compound was submitted to the next step without further purification. ^{1}H NMR (300 MHz, $CDCl_3$) δ 8.61 (s, OH), 8.34 (s, OH, minor isomer), 7.60–7.53 (m, 2H), 7.40–7.37 (m, 2H). ^{19}F NMR (283 MHz, $CDCl_3$) δ –62.32, –66.60 (minor isomer).

Synthesis of 1-(4-bromophenyl)-2,2,2-trifluoroethan-1-one *O*-tosyl oxime (S15) (mixture of *E*-, *Z*-isomers).

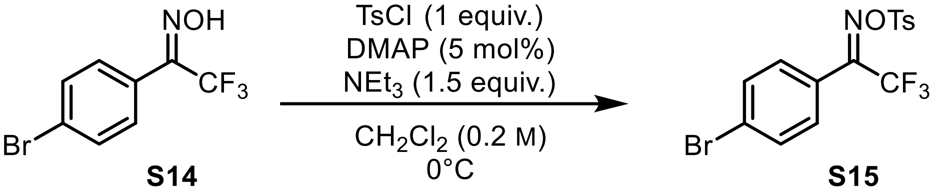

Compound **S14** (787 mg, 2.94 mmol, 1 equiv.) was dissolved in CH_2Cl_2 (0.2 M), and triethylamine (0.20 mL, 4.41 mmol, 1.5 equiv.), DMAP (18 mg, 0.14 mmol, 5 mol%) and tosyl chloride (560 mg, 2.94 mmol, 1 equiv.) were successively added at 0°C. The ice bath was removed after 5 min, and the reaction mixture was stirred at room temperature for 1 h. The mixture was then treated with sat. aq. NH_4Cl and extracted with CH_2Cl_2. The combined organic extracts were dried with sodium sulfate, filtered, and concentrated to afford the desired crude tosyl oxime **S15** as a white solid, with spectroscopic data in accordance with the literature. [12] The compound was submitted to the next step without further purification. ^{1}H NMR (300 MHz, $CDCl_3$) δ 7.87 (d, *J* = 8.3 Hz, 2H), 7.58 (d, *J* = 8.6 Hz, 2H), 7.37 – 7.42 (m, 2H), 7.27 (d, *J* = 8.6 Hz, 2H), 2.46 (s, 3H). ^{19}F NMR (283 MHz, $CDCl_3$) δ –61.53 (minor isomer), –66.70.

Synthesis of 3-(4-bromophenyl)-3-(trifluoromethyl)diaziridine (S16).

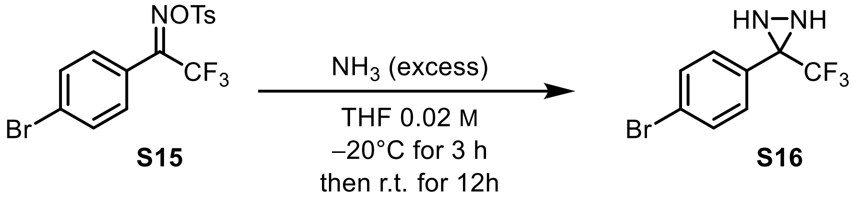

Tosyl oxime **S15** (1.22 g, 2.89 mmol, 1 equiv.) was transferred to a flame-dried 3-neck flask under argon in anhydrous THF (0.02 M) and cooled to –20°C. Anhydrous gaseous ammonia was bubbled into the stirred solution for 3 h. Then, the reaction was left stirring for 12 h, during which time it was allowed to warm from –20°C to room temperature. The mixture was quenched with sat. aq. NH_4Cl and extracted with diethyl ether (3 times). The combined organic layers were washed with brine and then dried with sodium sulfate, filtered and concentrated to afford the desired crude diaziridine **S16** as a colourless solid, which was submitted to the next step without further purification. The analytical data were in accordance with literature.[12] ^{1}H NMR (300 MHz, $CDCl_3$) δ 7.56 (d, *J* = 8.6 Hz, 2H), 7.49 (d, *J* = 8.6 Hz, 2H), 2.21 (d, *J* = 8.4 Hz, 1H), 2.81 (d, *J* = 8.4 Hz, 1H). ^{19}F NMR (283 MHz, $CDCl_3$) δ –75.48.

Synthesis of 3-(4-bromophenyl)-3-(trifluoromethyl)-3*H*-diazirine (D5).

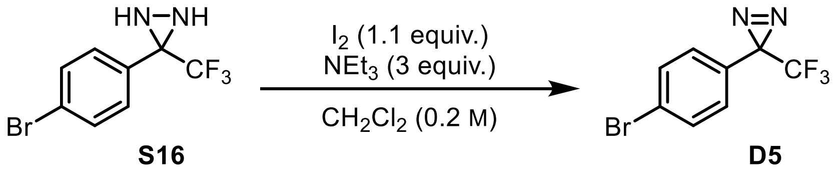

To a solution of the crude diaziridine **S16** (774 mg, 2.89 mmol, 1 equiv.) in CH_2Cl_2 (0.2 M) at 0 °C were added successively triethylamine (1.21 mL, 8.69 mmol, 3 equiv.) and iodine (809 mg, 3.18 mmol, 1.1 equiv.). The colored mixture was stirred at 0°C for 1 h. The mixture was diluted with CH_2Cl_2 and washed with sat. aq. sodium thiosulfate. The aqueous layer was re-extracted with CH_2Cl_2 (3 times). Then the combined organic extracts were washed with brine and dried with sodium sulfate, filtered, and concentrated. The residue was purified by silica gel column chromatography using pentane as eluent (R_f = 0.75) to afford the desired diazirine **D5** (742 mg, 2.80 mmol, 96%) as a pale-yellow oil, with spectroscopic data in accordance with the literature.[12] ^{1}H NMR (500 MHz, $CDCl_3$) δ7.54 (d, *J* = 8.7 Hz, 2H), 7.07 (d, *J* = 8.1 Hz, 2H).^{13}C NMR (126 MHz, $CDCl_3$) δ 132.13, 124.28, 121.92 (q, *J* = 274.8 Hz), 28.22 (q, *J* = 43.2, 41.9 Hz). ^{19}F NMR (283 MHz, $CDCl_3$) δ –65.33. IR (diamond-ATR) ν: 2928, 1614, 1497, 1339, 1232, 1185, 1151, 936, 814, 747, 532.

Fig. S15 ^{1}H NMR spectrum of **D5** in $CDCl_3$.

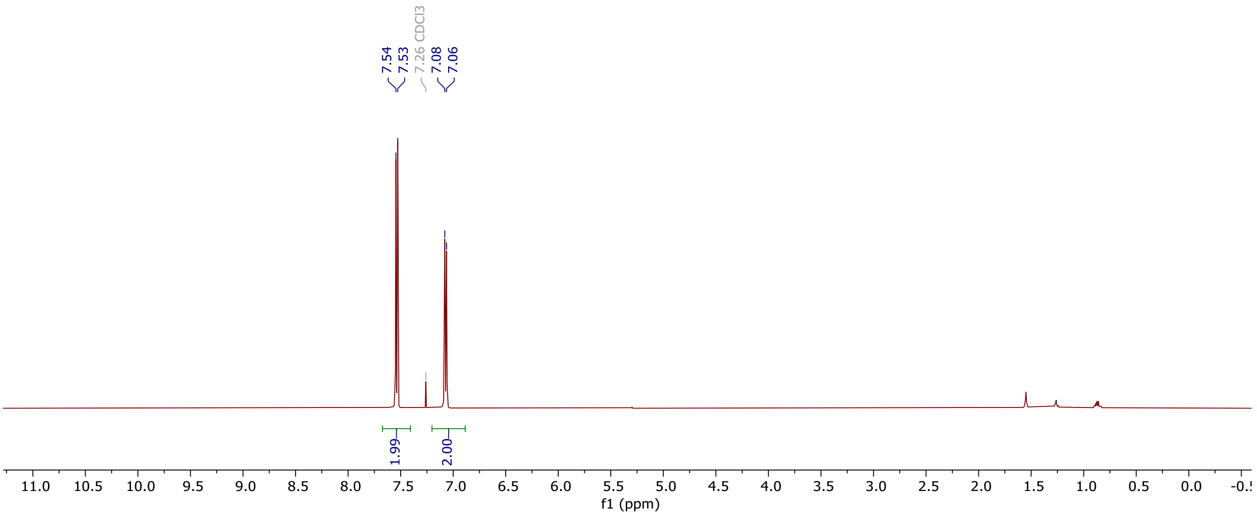

Fig. S16 ^{13}C NMR spectrum of **D5** in $CDCl_3$.

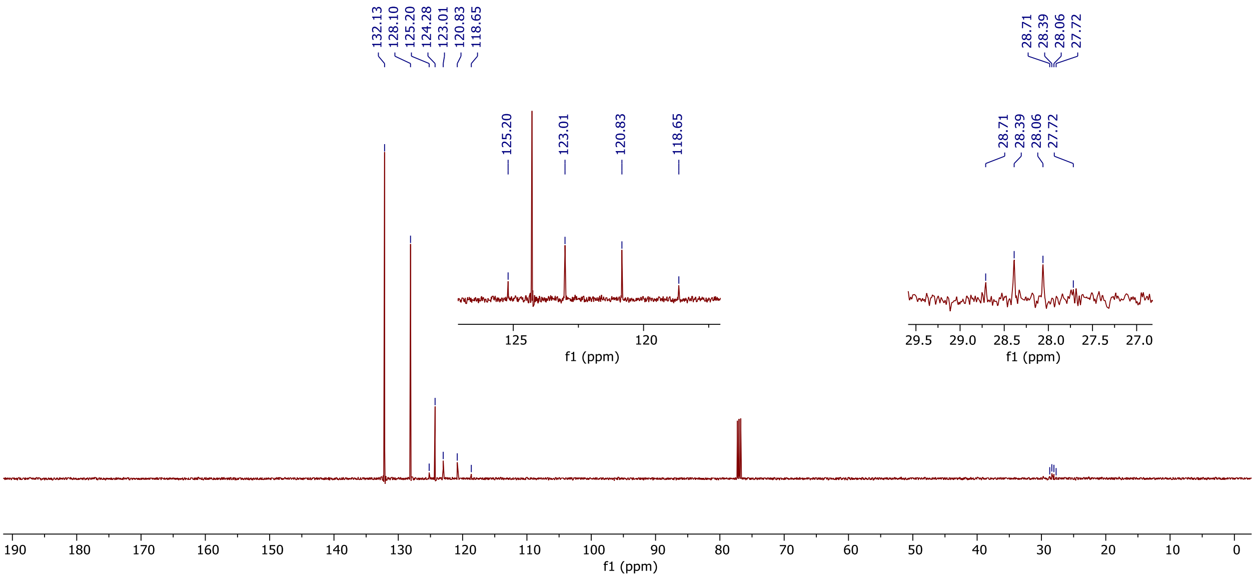

Fig. S17 ^{19}F NMR spectrum of **D5** in $CDCl_3$.

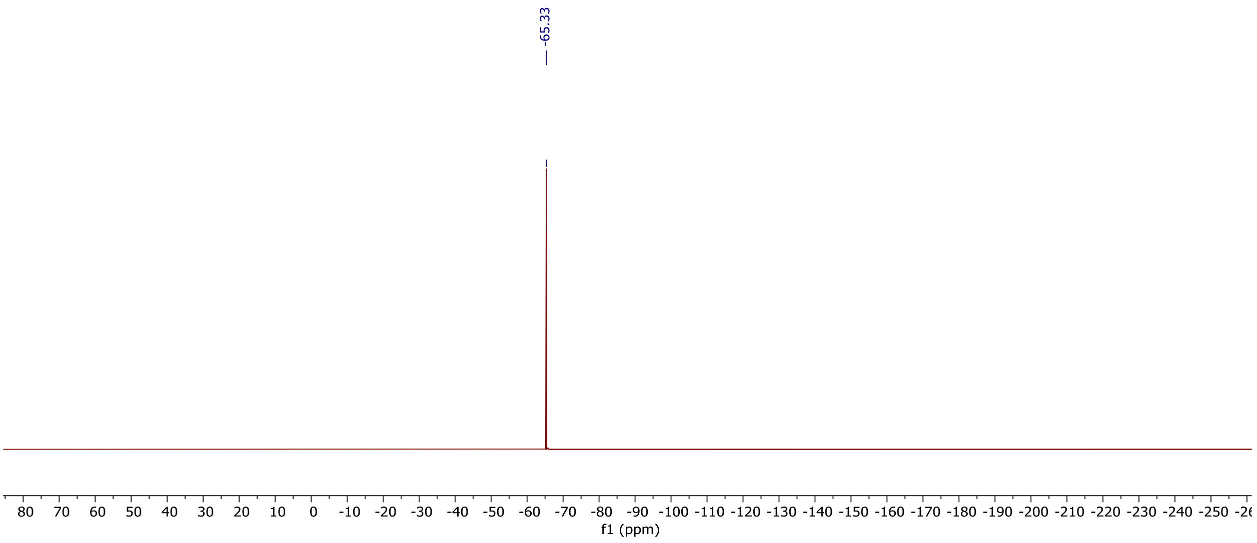

Synthesis of 2,2,2-trifluoro-1-(3-methoxyphenyl)ethan-1-one (S17).

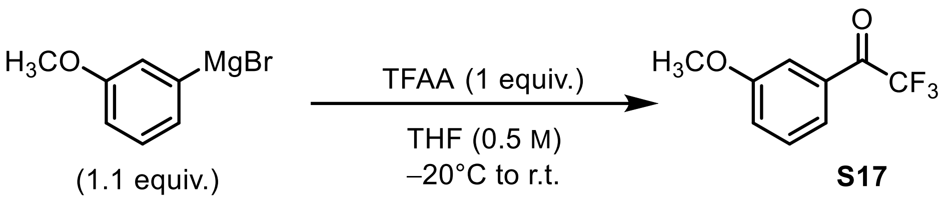

In a round bottom flask equipped with a magnetic stir bar and a condenser, to a stirring mixture of trifluoroacetic anhydride (0.66 mL, 4.76 mmol, 1 equiv.) in distilled THF (0.5 M) at –20°C, 3-methoxyphenyl magnesium Grignard (5.2 mL, 1 M in THF, 1.1 equiv.) was added. The mixture was stirred for 4 h and then quenched with NH4Cl and extracted with EtOAc (3 times). Then the combined organic extracts were washed with brine and dried with magnesium sulfate, filtered, and concentrated. The crude compound **S17** was purified by silica gel column chromatography using petroleum ether: EtOAc (8:2, R_f= 0.6) as eluent to afford the desired ketone **S17** (766 mg, 3.75 mmol, 79%) as a yellow oil. ^{1}H NMR (300 MHz, $CDCl_3$) δ 7.66 (d, *J* = 7.7 Hz, 1H), 7.57 (s, 1H), 7.46 (t, *J* = 8.0 Hz, 1H), 7.03 – 6.88 (m, 1H), 3.88 (s, 3H). ^{19}F NMR (283 MHz, $CDCl_3$) δ –71.22.

Synthesis of 2,2,2-trifluoro-1-(3-methoxyphenyl)ethan-1-one oxime (S18) (mixture of *E*-, *Z*-isomers).

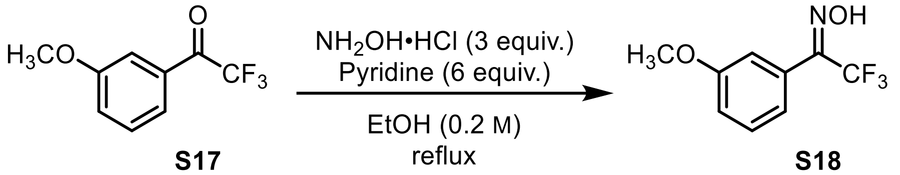

To a stirred solution of compound **S17** (673 mg, 3.3 mmol, 1 equiv,) in ethanol (0.2 M), hydroxylamine hydrochloride (688 mg, 9.9 mmol, 3 equiv.) and pyridine (1.6 mL, 19.8 mmol, 6 equiv.) were added and the reaction mixture was heated to reflux for 1 h. The mixture was then cooled to room temperature and the mixture was treated with 2 M HCl and extracted with diethyl ether (3 times). The combined organic layers were washed with distilled water until the pH of the washing layer became neutral, and then dried with sodium sulfate, filtered, and concentrated. The residue was dried under high vacuum for a prolonged time to afford the desired crude oxime **S18** (as a mixture of geometric isomers) as a white solid (720 mg). The compound was submitted to the next step without further purification. The analytical data were in accordance with literature.[13] ^{1}H NMR (300 MHz, $CDCl_3$) δ 9.64 (br-s, 1H), 9.33 (s, 0.3H, minor isomer), 7.47 – 7.28 (m, 1H and 0.3H minor isomer), 7.14 – 6.80 (m, 3H and 0.9H minor isomer), 3.83 (s, 3H), 3.78 (s, 1H, minor isomer).^{19}F NMR (283 MHz, $CDCl_3$) δ –62.37, –66.67.

Synthesis of 2,2,2-trifluoro-1-(3-methoxyphenyl)ethan-1-one *O*-tosyl oxime (S19) (mixture of *E*-, *Z*-isomers).

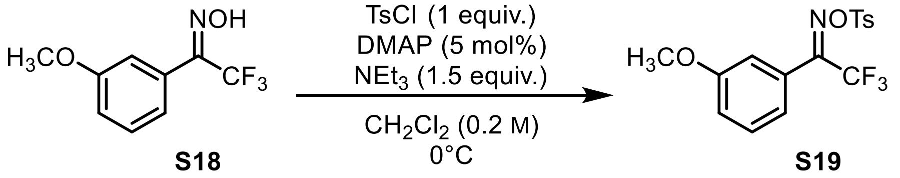

Compound **S18** (720 mg, 3.3 mmol, 1 equiv.) was dissolved in CH_2Cl_2 (0.2 M), and triethylamine (0.69 mL, 4.95 mmol, 1.5 equiv.), DMAP (20 mg, 0.165 mmol, 5 mol%) and tosyl chloride (629 mg, 3.3 mmol, 1 equiv.) were successively added at 0°C. The ice bath was removed after 5 min, and the reaction mixture was stirred at room temperature for 1 h. The mixture was then treated with sat. aq. NH_4Cl and extracted with CH_2Cl_2. The combined organic extracts were dried with sodium sulfate, filtered, and concentrated to afford the desired crude tosyl oxime **S19** (1.05 g) as a yellow oil, which was submitted to the next step without further purification. The analytical data were in accordance with literature.[13] ^{1}H NMR (300 MHz, $CDCl_3$) δ 7.92 – 7.85 (m, 2H), 7.41 – 7.36 (m, 2H), 7.34 – 7.29 (m, 1H), 7.08 – 7.02 (m, 1H), 6.97 – 6.91 (m, 1H), 6.90 – 6.84 (m, 1H), 3.82 (s, 3H), 2.48 (s, 3H). ^{19}F NMR (283 MHz, $CDCl_3$) δ –61.51 (minor isomer), –66.93.

Synthesis of 3-(3-methoxyphenyl)-3-(trifluoromethyl)diaziridine (S20).

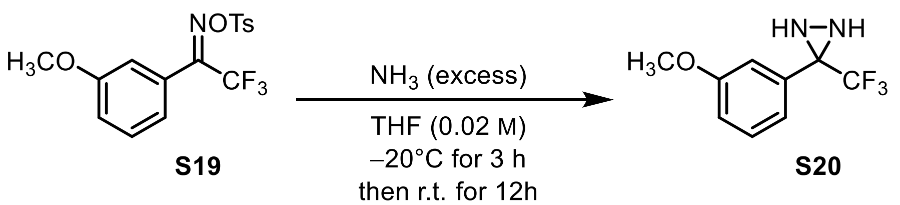

Tosyl oxime **S19** (1 g, 2.68 mmol, 1 equiv.) was transferred to a flame-dried 3-neck flask under argon in anhydrous THF (30 mL) and cooled to –20°C. Anhydrous gaseous ammonia was bubbled into the stirred solution for 3 h. Then, the reaction was left stirring for 12 h, during which time it was allowed to warm from –20°C to room temperature. The mixture was quenched with sat. aq. NH_4Cl and extracted with CH_2Cl_2 (3 times). The combined organic layers were washed with brine and then dried with sodium sulfate, filtered and concentrated to afford the desired crude diaziridine **S20**, which was submitted to the next step without further purification. The analytical data were in accordance with literature.[13] ^{1}H NMR (300 MHz, $CDCl_3$) δ 7.34 (t, *J* = 8.0 Hz, 1H), 7.20 (d, *J* = 7.7 Hz, 1H), 7.15 (s, 1H), 7.02 – 6.93 (m, 1H), 3.83 (s, 3H).^{19}F NMR (283 MHz, $CDCl_3$) δ –75.49.

Synthesis of 3-(3-methoxyphenyl)-3-(trifluoromethyl)-3*H*-diazirine (D8).

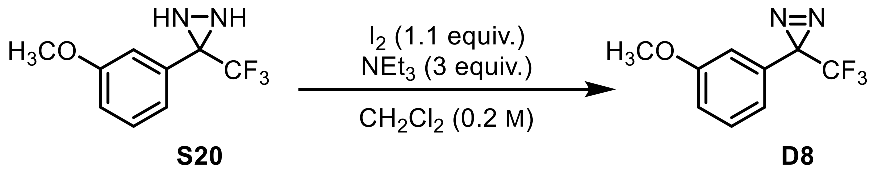

To a solution of the crude diaziridine **S20** (585 mg, 2.68 mmol, 1 equiv.) in CH_2Cl_2 (0.2 M) at 0 °C were added successively triethylamine (1.1 mL, 8.04 mmol, 3 equiv.) and iodine (748 mg, 2.94 mmol, 1.1 equiv.). The colored mixture was stirred at 0°C for 1 h. The mixture was diluted with CH_2Cl_2 and washed with sat. aq. sodium thiosulfate. The aqueous layer was re-extracted with CH_2Cl_2 (3 times). Then the combined organic extracts were washed with brine and dried with sodium sulfate, filtered, and concentrated. The residue was purified by silica gel column chromatography using pentane: EtOAc (9:1) as eluent to afford the desired diazirine **D8** (241 mg, 1.1 mmol, 42%) as a pale-yellow oil, in accordance with literature data.[13] ^{1}H NMR (500 MHz, $CDCl_3$) δ 7.31 (t, *J* = 8.0 Hz, 1H), 6.95 (ddd, *J* = 8.3, 2.6, 0.9 Hz, 1H), 6.78 (ddq, *J* = 7.8, 1.7, 0.9 Hz, 1H), 6.69 (t, *J* = 2.3 Hz, 1H), 3.81 (s, 3H). ^{13}C NMR (126 MHz, $CDCl_3$) δ 159.94, 130.70, 130.17, 122.25 (d, *J* = 274.7 Hz), 118.89, 115.35, 112.39, 55.48, 29.86. ^{19}F NMR (283 MHz, $CDCl_3$) δ –65.16. UV (*n*-hexane): $\lambda_{max,\ diazirine}$ = 355 nm. IR (diamond-ATR) ν: 2959, 2929, 2874, 2860, 1606, 1462, 1265, 1247, 1115, 1100, 1019, 729.

Fig. S18 ^{1}H NMR spectrum of **D8** in $CDCl_3$.

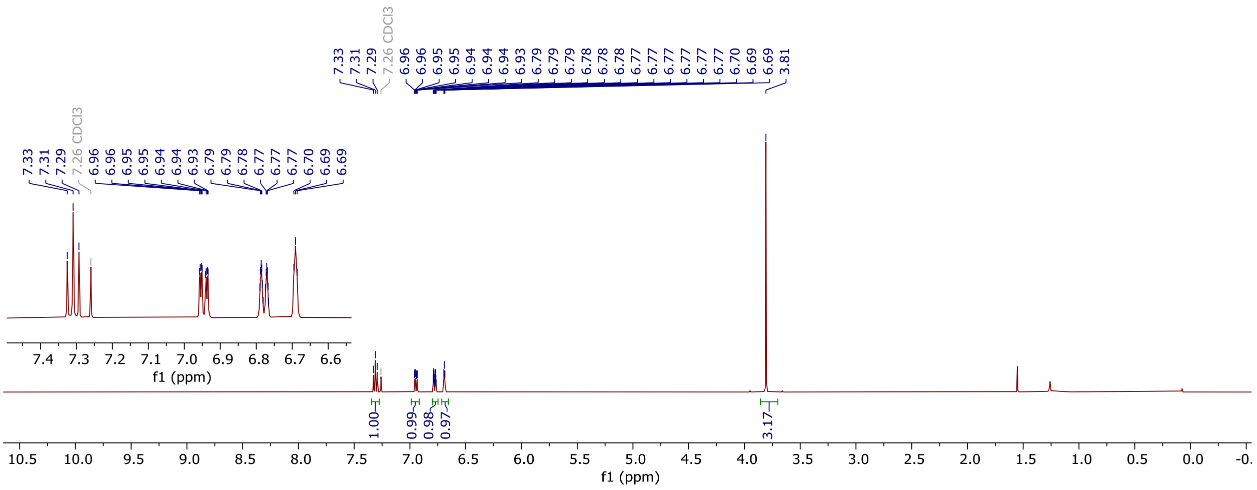

Fig. S19 ^{13}C NMR spectrum of **D8** in $CDCl_3$.

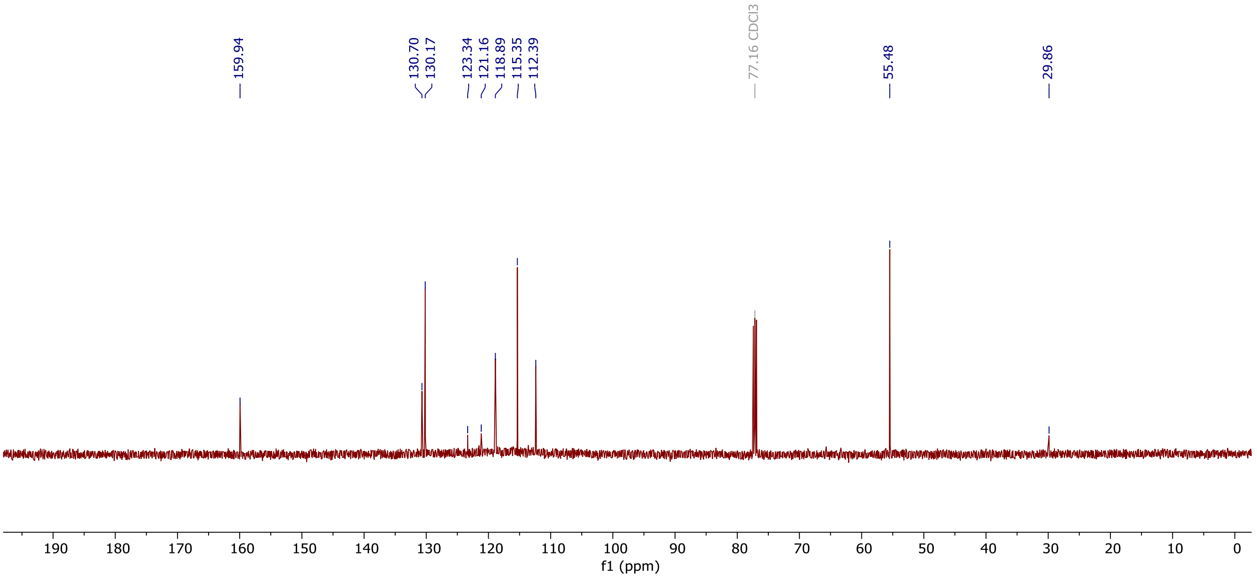

Fig. S20 ^{19}F NMR spectrum of **D8** in $CDCl_3$.

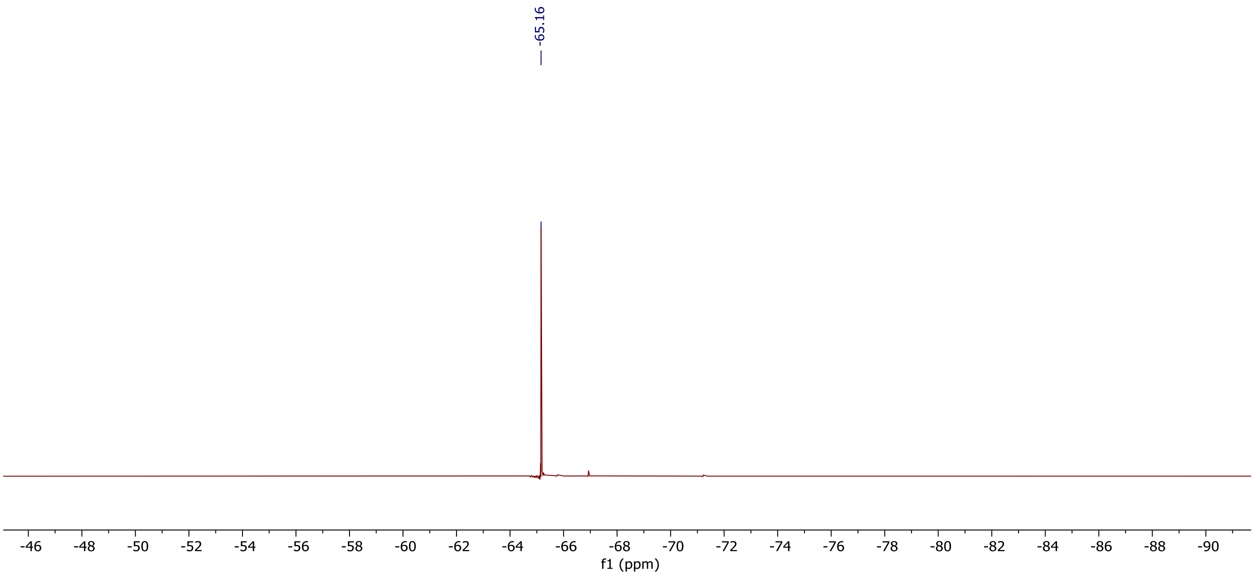

Synthesis of 3-(3-hydroxyphenyl)-3-(trifluoromethyl)-3*H*-diazirine (D9).

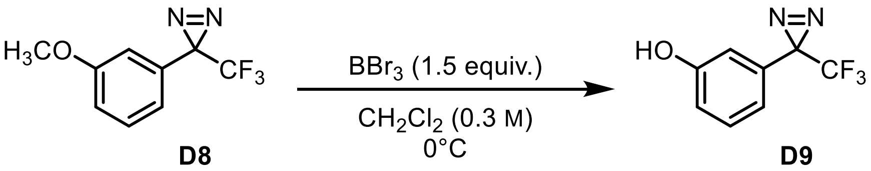

The reaction was carried out in dim light: to a solution of compound **D8** (400 mg, 1.85 mmol, 1 equiv.) in CH_2Cl_2 (0.3 M) at 0 °C were added successively a solution of 1 M boron tribromide in CH_2Cl_2 (2.8 mL, 2.78 mmol, 1.5 equiv.). The colored mixture was stirred at 0°C and slowly warm up to room temperature for 4 h. Water (5 mL) was added to the reaction mixture followed by CH_2Cl_2. The aqueous layer was re-extracted with CH_2Cl_2 (2 times). Then the combined organic extracts were dried with sodium sulfate, filtered, and concentrated. The residue was characterized directly without further purification, affording the desired diazirine **D9** (365 mg, 1.81 mmol, 98%) as a brown-yellow oil. The analytical data were in accordance with literature.[13] ^{1}H NMR (500 MHz, CD_2Cl_2) δ 7.28 (t, *J* = 8.0 Hz, 1H), 6.90 (dd, *J* = 8.2, 2.4 Hz, 1H), 6.74 (d, *J* = 7.8 Hz, 1H), 6.68 (s, 1H), 5.42 (s, 1H). ^{13}C NMR (126 MHz, CD_2Cl_2) δ 156.69, 131.20, 130.86, 122.67 (q, *J* = 274.8 Hz), 119.22, 117.46, 114.02, 30.28. ^{19}F NMR (283 MHz, CD_2Cl_2) δ –65.60. IR (diamond-ATR) ν: 3343.8, 2954, 2924, 2854, 1588, 1456, 1158.

Fig. S21 ^{1}H NMR spectrum of **D9** in CD_2Cl_2.

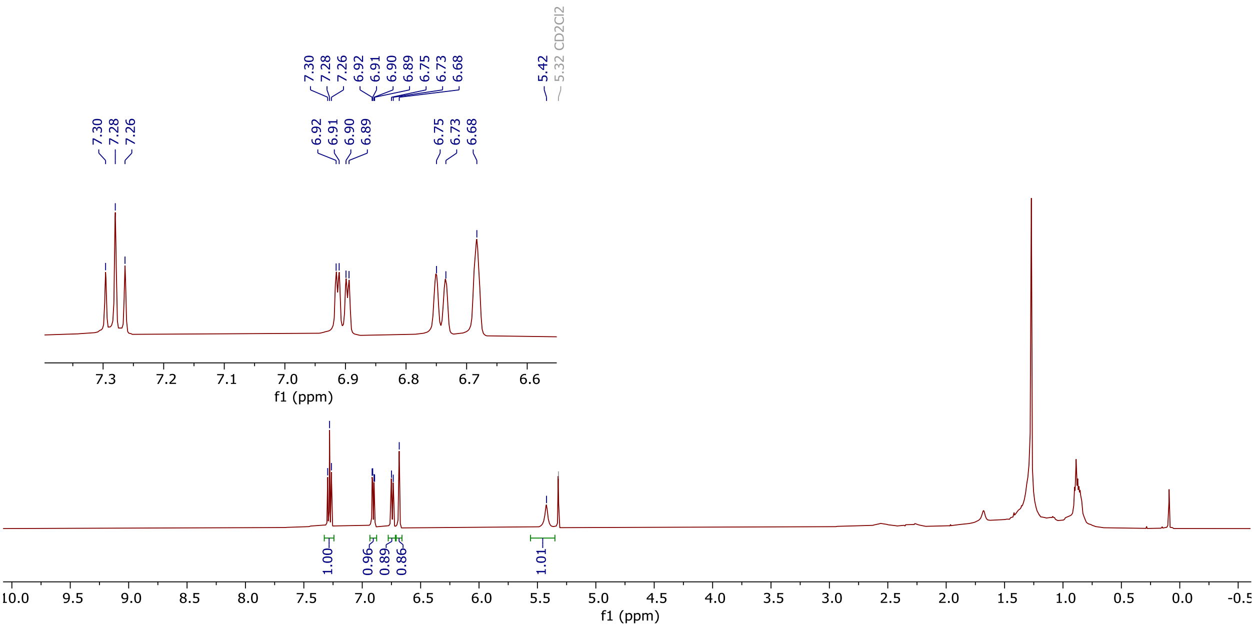

Fig. S22 ^{13}C NMR spectrum of **D9** in CD_2Cl_2.

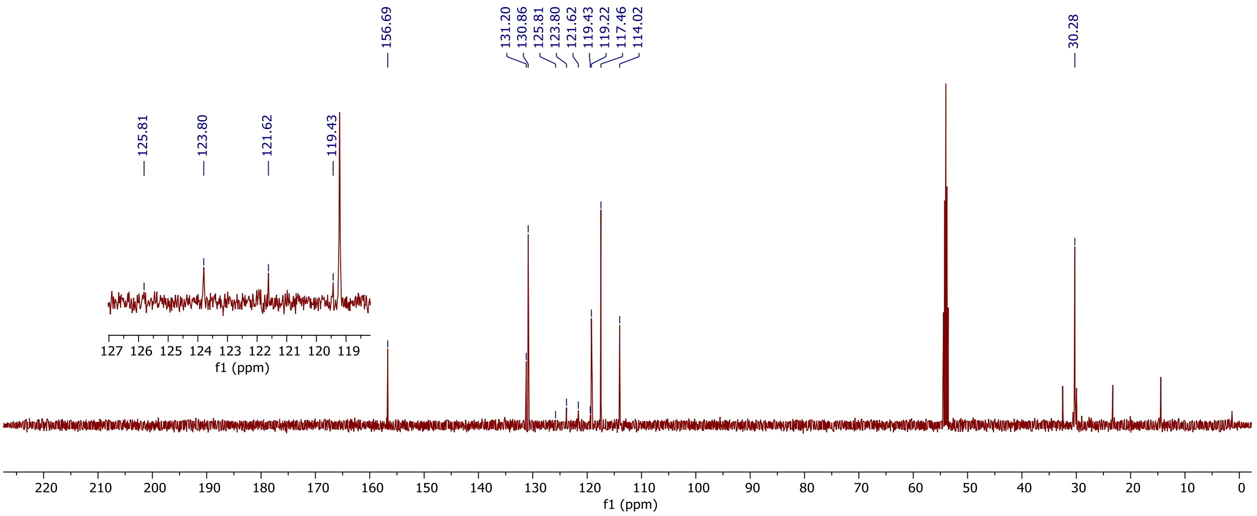

Fig. S23 ^{19}F NMR spectrum of **D9** in CD_2Cl_2.

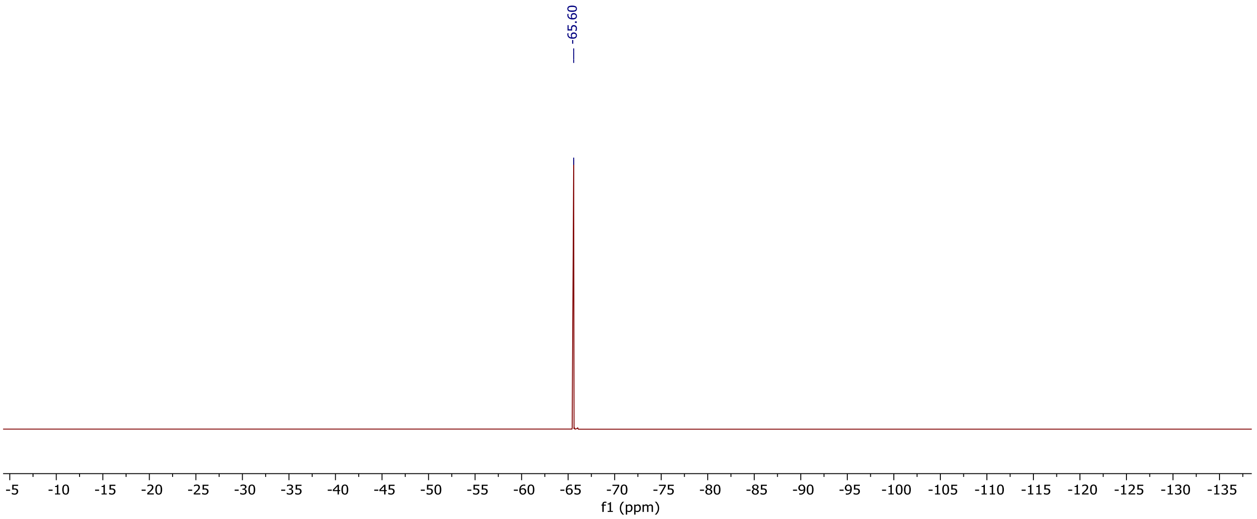

Synthesis of methyl 3,5-dimethoxybenzoate (S21).

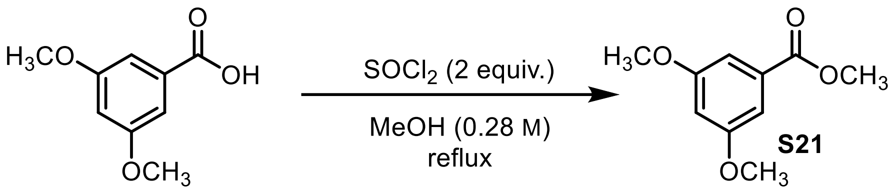

To a stirring solution of commercially available 3,5-dimethoxybenzoic acid (1.06 g, 5.8 mmol, 1 equiv.) in MeOH (40 mL), thionyl chloride (0.84 mL, 11.6 mmol, 2 equiv.) was slowly added dropwise. The suspension was heated to reflux for 16 h. The mixture was then cooled to room temperature and it was treated with $NaHCO_3$ until pH is around 7-8. The organic layer was extracted with diethyl ether (3 times) and washed with sat. NaHCO3 and brine, and then dried over anhydrous sodium sulfate. Solvent was removed to afford the desired ester **S21** as a white solid (1.07 g, 5.49 mmol, 95%), in accordance with literature data.[14] ^1H NMR (300 MHz, $CDCl_3$) δ 7.19 (d, J = 2.4 Hz, 2H), 6.65 (t, J = 2.4 Hz, 1H), 3.91 (s, 3H), 3.83 (s, 6H).

Synthesis of 1-(3,5-dimethoxyphenyl)-2,2,2-trifluoroethan-1-one (S22).

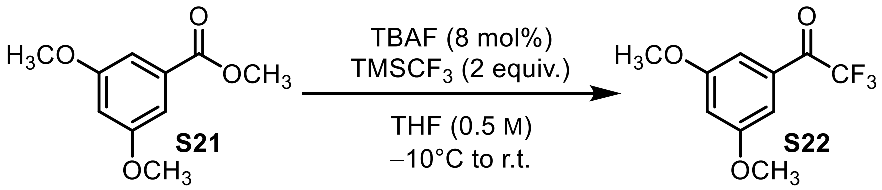

In a flame-dried flask, to a stirring solution of **S21** (1 g, 5.48 mmol, 1 equiv.) in distilled THF (1 M) under argon atmosphere at –10 °C, $TMSCF_3$ (1.2 mL, 8.2 mmol, 2 equiv.) and then TBAF (0.43 mL, 1 M in THF, 8 mol%) were slowly added dropwise. The resulting solution was allowed to warm

to room temperature with continued stirring for an hour. The reaction was left stirring for 1 h and then quenched with NH_4Cl, extracted with diethyl ether (2 times) and the combined organic extracts were washed with brine and dried with sodium sulfate to afford the desired ketone **S22**, which was submitted to the next step without further purification. The analytical data were in accordance with literature.[15] ^1H NMR (300 MHz, $CDCl_3$) δ 6.71 (d, J = 2.2 Hz, 2H), 6.45 (t, J = 2.1 Hz, 1H), 3.83 ppm (s, 6H). ^{19}F NMR (283 MHz, $CDCl_3$) δ –71.19.

Synthesis of 1-(3,5-dimethoxyphenyl)-2,2,2-trifluoroethan-1-one oxime (S23) (mixture of *E*-, *Z*-isomers).

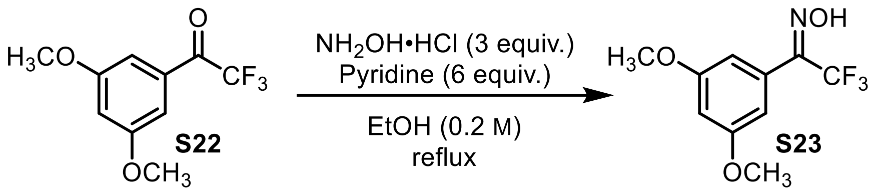

To a stirred solution of compound **S22** in ethanol (0.2 M), hydroxylamine hydrochloride (1.14 g, 16.5 mmol, 3 equiv.) and pyridine (2.6 mL, 33 mmol, 6 equiv.) were added and the reaction mixture was heated to reflux for 21 h. The mixture was then cooled to room temperature and the mixture was treated with 2 M HCl and extracted with diethyl ether (3 times). The combined organic layers were washed with distilled water until the pH of the washing layer became neutral, and then dried with sodium sulfate, filtered, and concentrated. The residue was dried under high vacuum for a prolonged time to afford the desired crude oxime **S23** (as a mixture of geometric isomers) as a yellow oil. The compound was submitted to the next step without further purification. The analytical data were in accordance with literature. [15] ^1H NMR (300 MHz, $CDCl_3$) δ 7.18 (d, *J* = 2.4 Hz, 2H), 6.64 (t, *J* = 2.4 Hz, 1H), 3.82 (s, 6H). ^{19}F NMR (283 MHz, $CDCl_3$) δ –62.35, –66.61.

Synthesis of 1-(3,5-dimethoxyphenyl)-2,2,2-trifluoroethan-1-one *O*-tosyl oxime (S24) (mixture of *E*-, *Z*-isomers).

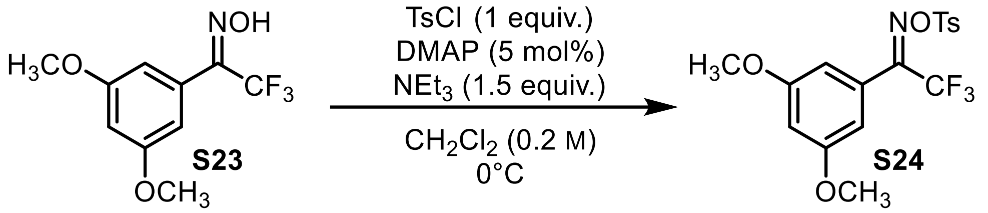

Compound **S23** (1.3 g, 5.49 mmol, 1 equiv.) was dissolved in CH_2Cl_2 (0.2 M), and triethylamine (1.15 mL, 8.2 mmol, 1.5 equiv.), DMAP (34 mg, 0.27 mmol, 5 mol%) and tosyl chloride (1.05 g, 5.49 mmol, 1 equiv.) were successively added at 0°C. The ice bath was removed after 5 min, and the reaction mixture was stirred at room temperature for 1 h. The mixture was then treated with sat. aq. NH_4Cl and extracted with CH_2Cl_2. The combined organic extracts were dried with sodium sulfate, filtered, and concentrated to afford the desired crude tosyl oxime **S24** (714 mg) as a colourless solid, which was submitted to the next step without further purification. The analytical data were in accordance with literature.[15] ^1H NMR (300 MHz, $CDCl_3$) δ 7.89 (d, *J* = 8.1 Hz, 2H), 7.38 (d, J = 8.2 Hz, 2H), 6.56 (t, J = 2.0 Hz, 1H), 6.44 (d, J = 2.1 Hz, 2H), 3.81 (s, 6H), 2.46 ppm (s, 3H). ^{19}F NMR (283 MHz, $CDCl_3$) δ –61.55, –67.11 (minor isomer).

Synthesis of 3-(3,5-dimethoxyphenyl)-3-(trifluoromethyl)diaziridine (S25).

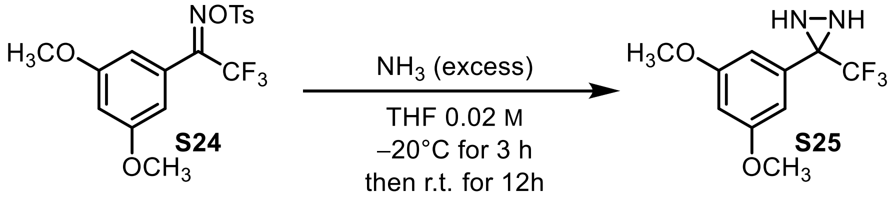

Tosyl oxime **S24** (1 equiv.) was transferred to a flame-dried 3-neck flask under argon in anhydrous THF (0.02 M) and cooled to –20°C. Anhydrous gaseous ammonia was bubbled into the stirred solution for 3 h. Then, the reaction was left stirring for 12 h, during which time it was allowed to warm from –20°C to room temperature. The mixture was quenched with sat. aq. NH_4Cl and extracted with diethyl ether (3 times). The combined organic layers were washed with brine and then dried with sodium sulfate, filtered and concentrated to afford the desired crude diaziridine **S25**, which was submitted to the next step without further purification. The analytical data were in accordance with literature.[15] ^1H NMR (300 MHz, $CDCl_3$) δ 6.77 (d, J = 2.0 Hz, 2H), 6.51 (d, J = 2.0 Hz, 1H), 3.81 (s, 6H), 2.74 (d, J = 9.0 Hz, 1H), 2.21 ppm (d, J = 9.0 Hz, 1H). ^{19}F NMR (283 MHz, $CDCl_3$) δ –75.41.

Synthesis of 3-(3,5-dimethoxyphenyl)-3-(trifluoromethyl)-3*H*-diazirine (D10).

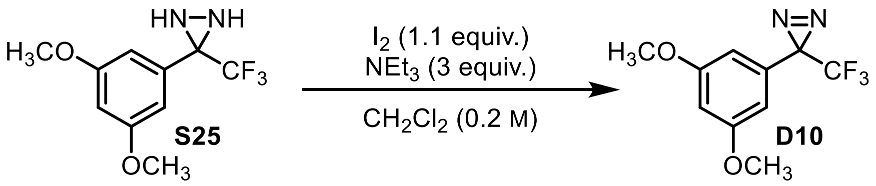

To a solution of the crude diaziridine **S25** (1.3 g, 5.48 mmol, 1 equiv.) in CH_2Cl_2 (0.2 M) at 0 °C were added successively triethylamine (2.2 mL, 16.4 mmol, 3 equiv.) and iodine (1.5 g, 6.04 mmol, 1.1 equiv.). The colored mixture was stirred at 0°C for 1 h. The mixture was diluted with CH_2Cl_2 and washed with sat. aq. sodium thiosulfate. The aqueous layer was re-extracted with CH_2Cl_2 (3 times). Then the combined organic extracts were washed with brine and dried with sodium sulfate, filtered, and concentrated. The residue was purified by silica gel column chromatography using pentane: diethyl ether (8:2, R_f= 0.76), as eluent mixture to afford the desired diazirine **D10** (1.16 g, 4.72 mmol, 86%) as a pale-yellow oil, in

accordance with literature data.[15] ^{1}H NMR (500 MHz, $CDCl_3$) δ 6.48 (t, *J* = 2.2 Hz, 1H), 6.32 – 6.27 (m, 2H), 3.78 (s, 6H). ^{13}C NMR (126 MHz, $CDCl_3$) δ 161.25, 131.32, 104.90, 122.20 (q, *J* = 274.3 Hz), 101.72, 55.59, 28.67 (d, *J* = 40.1 Hz). ^{19}F NMR (283 MHz, $CDCl_3$) δ –65.07. UV (*n*-hexane): $\lambda_{max, diazirine}$ = 353 nm. IR (diamond-ATR) ν: 2963, 2942, 2846, 1595, 1460, 1429, 1288, 1207, 1151, 999, 704.

Fig. S24 ^{1}H NMR spectrum of **D10** in $CDCl_3$.

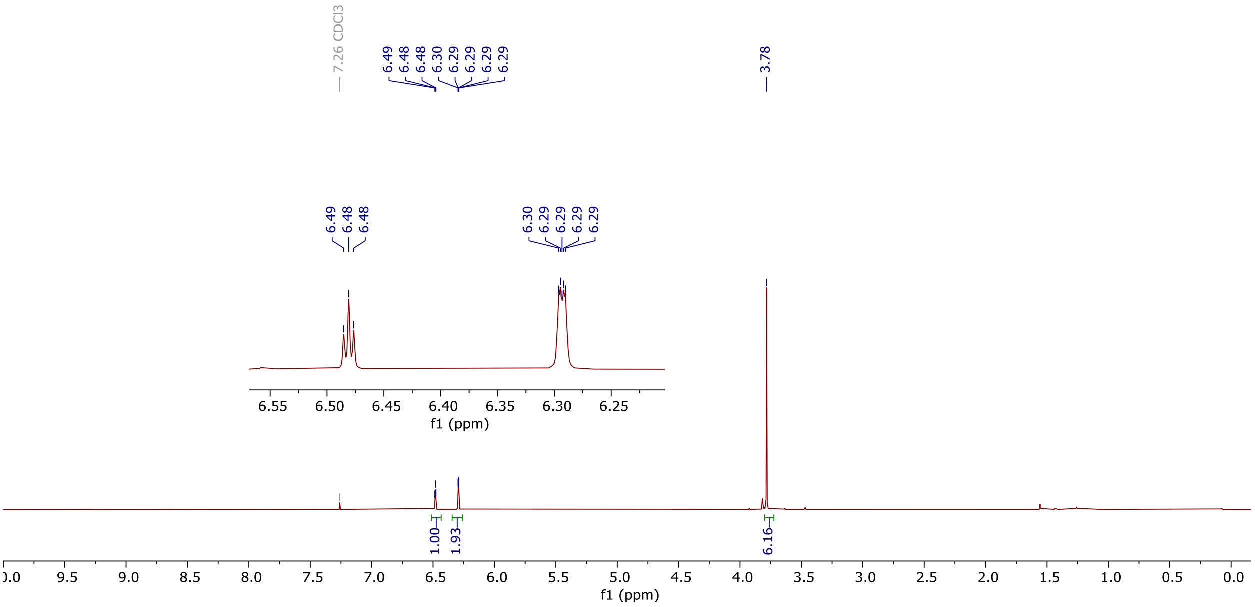

Fig. S25 ^{13}C NMR spectrum of **D10** in $CDCl_3$.

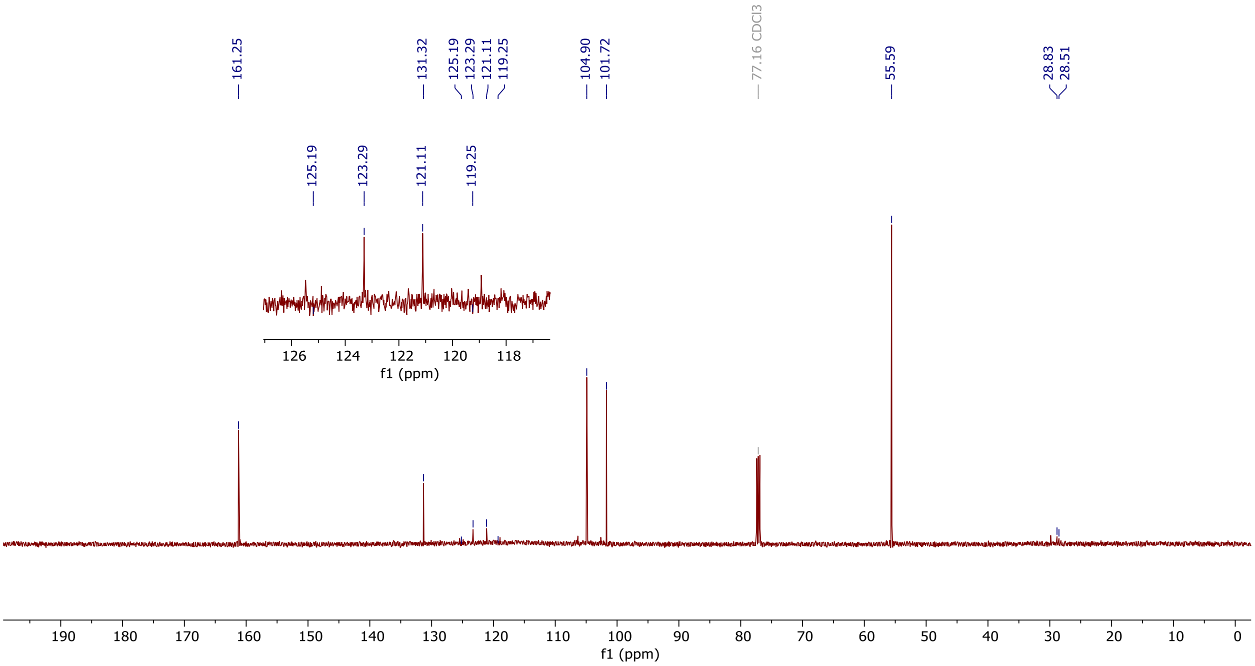

Fig. S26 ^{19}F NMR spectrum of **D10** in $CDCl_3$.

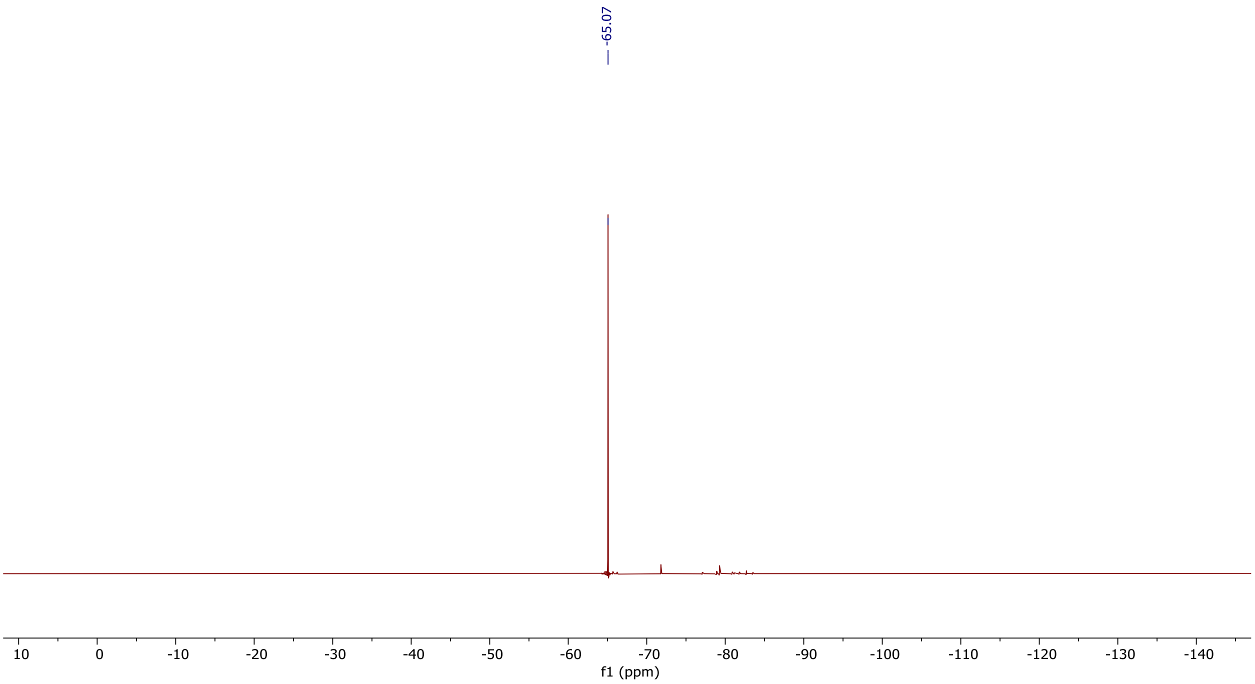

Synthesis of methyl 4-(trifluoromethyl)benzoate (S26).

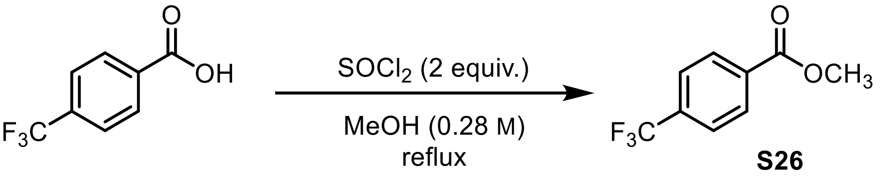

To a stirring solution of commercially available 4-(trifluoromethyl)benzoic acid (1 g, 5.26 mmol, 1 equiv.) in MeOH (20 mL), thionyl chloride (0.76 mL, 10.5 mmol, 2 equiv.) was slowly added dropwise. The suspension was heated to reflux for 2 h. The mixture was then cooled to room temperature and it was treated with $NaHCO_3$ until pH is around 7-8. The organic layer was extracted with diethyl ether (3 times) and washed with sat. NaHCO3 and brine, and then dried over anhydrous magnesium sulfate. Solvent was removed to afford the desired ester **S26** as pale-yellow oil (1.2 g, 5 mmol, 95%), in accordance with literature data.[16] ^{1}H NMR (300 MHz, $CDCl_3$) δ 8.16 (d, *J* = 8.1 Hz, 2H), 7.71 (d, *J* = 8.2 Hz, 2H), 3.96 (s, 3H). ^{19}F NMR (471 MHz, $CDCl_3$) δ –63.13.

Synthesis of 2,2,2-trifluoro-1-(4-(trifluoromethyl)phenyl)ethan-1-one (S27).

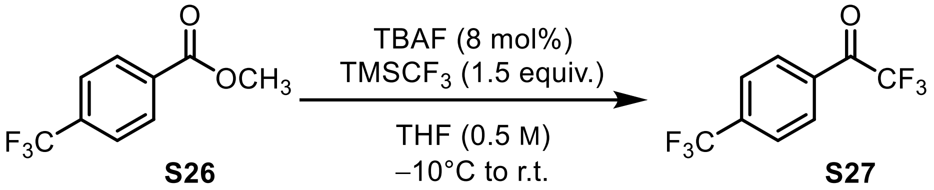

In a flame-dried flask, to a stirring solution of compound **S26** (1.1 g, 5.5 mmol, 1 equiv.) in distilled THF (11 mL) under argon atmosphere at –10 °C, $TMSCF_3$ (1.2 mL, 8.22 mmol, 1.5 equiv.) and then TBAF (0.5 mL, 1 M in THF, 8 mol%) were slowly added dropwise. The resulting solution was allowed to warm to room temperature with continued stirring for 2 h. The reaction was quenched with NH_4Cl, extracted with diethyl ether (2 times) and the combined organic extracts were washed with brine and dried with sodium sulfate. The filtered and concentrated oil was purified by silica gel column chromatography using pentane: ethyl acetate (9:1, R_f = 0.42) as eluent to afford the desired ketone **S27** (892 mg, 3.68 mmol, 67%) as a colourless oil, in accordance with literature data.[17] ^{1}H NMR (300 MHz, $CDCl_3$) δ 8.14 (d, *J* = 8.4 Hz, 2H), 7.77 (d, *J* = 8.4 Hz, 2H). ^{19}F NMR (283 MHz, $CDCl_3$) δ –63.63 (*p*-CF_3), –71.83.

Synthesis of 2,2,2-trifluoro-1-(4-(trifluoromethyl)phenyl)ethan-1-one oxime (S28) (mixture of *E*-, *Z*-isomers).

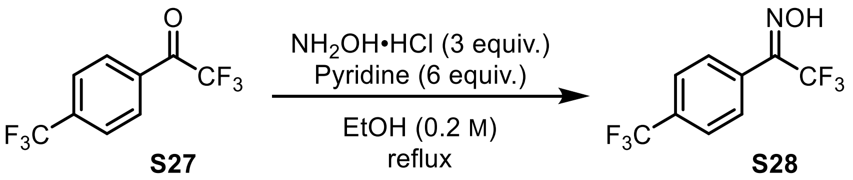

To a stirred solution of compound **S27** (892 mg, 3.68 mmol, 1 equiv.) in ethanol (0.2 M), hydroxylamine hydrochloride (767 mg, 11.4 mmol, 3 equiv.) and pyridine (1.8 mL, 22 mmol, 6 equiv.) were added and the reaction mixture was heated to reflux for 21 h. The mixture was then cooled

to room temperature and the mixture was treated with 2 M HCl and extracted with diethyl ether (3 times). The combined organic layers were washed with distilled water until the pH of the washing layer became neutral, and then dried with sodium sulfate, filtered, and concentrated. The residue was dried under high vacuum for a prolonged time to afford the desired crude oxime **S28** (as a mixture of geometric isomers) as a colorless oil. The compound was submitted to the next step without further purification. ^{1}H NMR (300 MHz, $CDCl_3$) δ 8.62 (d, *J* = 1.8 Hz, 1H, OH), 8.60 (d, *J* = 1.6 Hz, 1H, OH, minor isomer), 7.74 (d, *J* = 7.9 Hz, 2H), 7.39 – 7.32 (m, 2H). ^{19}F NMR (283 MHz, $CDCl_3$) δ –62.36 (minor isomer), –62.96 (minor isomer, *p*-CF_3), –63.13 (*p*-CF_3), –66.24.

Synthesis of 2,2,2-trifluoro-1-(4-(trifluoromethyl)phenyl)ethan-1-one *O*-tosyl oxime (S29) (mixture of *E,Z*-isomers).

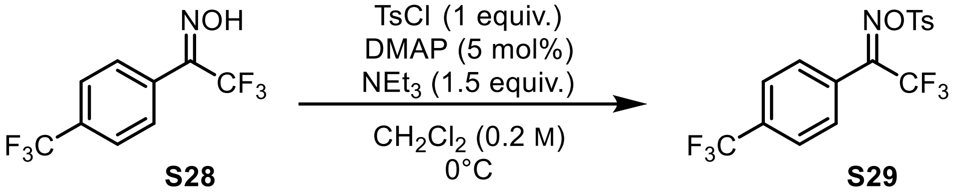

Compound **S28** (789 mg, 3.07 mmol, 1 equiv.) was dissolved in CH_2Cl_2 (0.2 M), and triethylamine (0.65 mL, 4.6 mmol, 1.5 equiv.), DMAP (19 mg, 0.15 mmol, 5 mol%) and tosyl chloride (585 mg, 3.07 mmol, 1 equiv.) were successively added at 0°C. The ice bath was removed after 5 min, and the reaction mixture was stirred at room temperature for 1 h. The mixture was then treated with sat. aq. NH_4Cl and extracted with CH_2Cl_2. The combined organic extracts were dried with sodium sulfate, filtered, and concentrated to afford the desired crude tosyl oxime **S29** as a yellow oil. The compound was submitted to the next step without further purification. ^{1}H NMR (300 MHz, $CDCl_3$) δ 7.90 (d, *J* = 8.5 Hz, 2H, minor isomer), 7.89 (d, *J* = 8.4 Hz, 2H), 7.76 (d, *J* = 8.2 Hz, 2H), 7.70 (d, *J* = 8.3 Hz, 2H, minor isomer), 7.58 (d, *J* = 8.7 Hz, 2H, minor isomer), 7.51 (d, *J* = 8.4 Hz, 1H), 7.44 – 7.33 (m, 4H, both isomers), 2.49 (s, 3H), 2.47 (s, 3H, minor isomer). ^{19}F NMR (283 MHz, $CDCl_3$) δ –61.53 (minor isomer), –63.26 (minor isomer, *p*-CF_3), –63.36 (*p*-CF_3), –66.93.

Synthesis of 3-(trifluoromethyl)-3-(4-(trifluoromethyl)phenyl)diaziridine (S30).

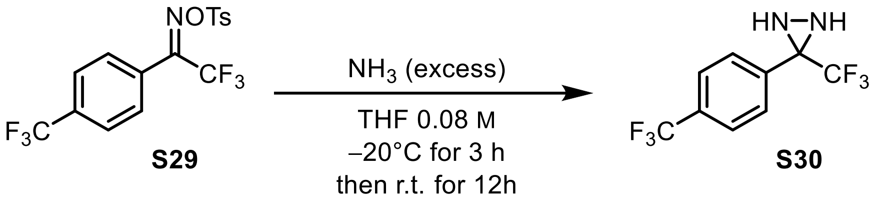

Tosyl oxime **S29** (1.2 g, 3 mmol, 1 equiv.) was transferred to a flame-dried 3-neck flask under argon in anhydrous THF (0.08 M) and cooled to –20°C. Anhydrous gaseous ammonia was bubbled into the stirred solution for 3 h. Then, the reaction was left stirring for 12 h, during which time it was allowed to warm from –20°C to room temperature. The mixture was quenched with sat. aq. NH_4Cl and extracted with diethyl ether (3 times). The combined organic layers were washed with brine and then dried with sodium sulfate, filtered, and concentrated to afford the desired crude diaziridine **S30** as a colourless oil, which was submitted to the next step without further purification. ^{1}H NMR (300 MHz, $CDCl_3$) δ 7.78 (d, *J* = 8.5 Hz, 2H), 7.70 (d, *J* = 8.4 Hz, 2H), 2.88 (d, *J* = 8.8 Hz, 1H, NH), 2.26 (d, *J* = 8.8 Hz, 1H, NH). ^{19}F NMR (283 MHz, $CDCl_3$) δ –63.03 (*p*-CF_3), –75.23.

Synthesis of 3-(trifluoromethyl)-3-(4-(trifluoromethyl)phenyl)-3*H*-diazirine (D11).

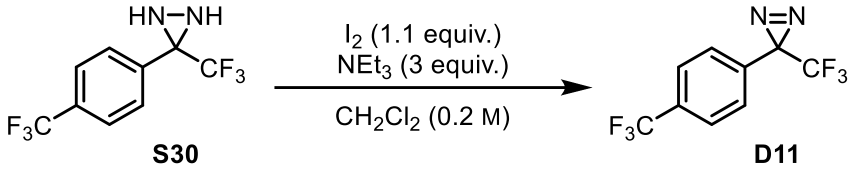

To a solution of the crude diaziridine **S30** (644 mg, 2.5 mmol, 1 equiv.) in CH_2Cl_2 (0.2 M) at 0 °C were added successively triethylamine (1.1 mL, 7.5 mmol, 3 equiv.) and iodine (702 mg, 2.8 mmol, 1.1 equiv.). The colored mixture was stirred at 0°C for 1 h. The mixture was diluted with CH_2Cl_2 and washed with sat. aq. sodium thiosulfate. The aqueous layer was re-extracted with CH_2Cl_2 (3 times). Then the combined organic extracts were washed with brine and dried with sodium sulfate, filtered, and concentrated (the title compound is very volatile and decomposes very easily). The residue was purified by silica gel column chromatography using pentane (R_f = 0.86) as eluent to afford the desired diazirine **D11** (400 mg, 1.57 mmol, 63%) as a colourless oil. ^{1}H NMR (300 MHz, $CDCl_3$) δ 7.67 (d, *J* = 8.2 Hz, 1H), 7.32 (dd, *J* = 8.3, 0.7 Hz, 1H).^{13}C NMR (126 MHz, $CDCl_3$) δ 133.13, 132.00 (d, *J* = 33.0 Hz), 127.05(d, *J* = 1.5 Hz), 126.00 (q, *J* = 3.7 Hz), 124.74, 123.07, 122.58, 120.89, 34.29, 29.86. ^{19}F NMR (471 MHz, $CDCl_3$) δ –63.09 (*p*-CF_3), –65.09. IR (diamond-ATR) ν: 2959, 2928, 2860, 1265, 1247, 1115, 1101, 1019, 729. GC–MS *m/z* (% relative intensity) [ion]: 254 (2) $[M]^+$, 226 (100) $[M - N_2]^+$, 207 (40) $[C_9H_4F_5]^+$, 187 (20) $[C_9H_3F_4]^+$, 176 (60) $[C_8H_4F_4]^+$, 157 (60) $[C_8H_4F_3]^+$, 138 (18) $[C_8H_4F_2]^+$, 137 (32) $[C_8H_3F_2]^+$, 126 (18) $[C_7H_4F_2]^+$, 107 (26) $[C_7H_4F_1]^+$, 87 (20) $[C_7H_3]^+$, 81 (10) $[C_2F_3]^+$, 69 (12) $[CF_3]^+$. [M = $C_9H_4F_6N_2$ (254.03)].

Fig. S27 ^{1}H NMR spectrum of **D11** in $CDCl_3$.

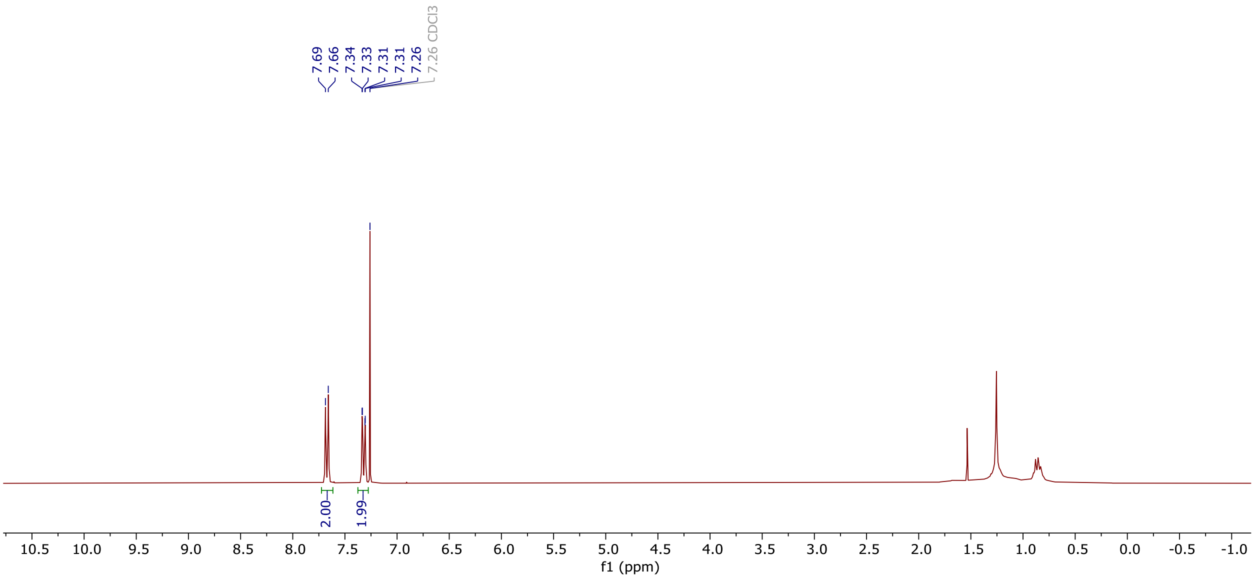

Fig. S28 ^{13}C NMR spectrum of **D11** in $CDCl_3$.

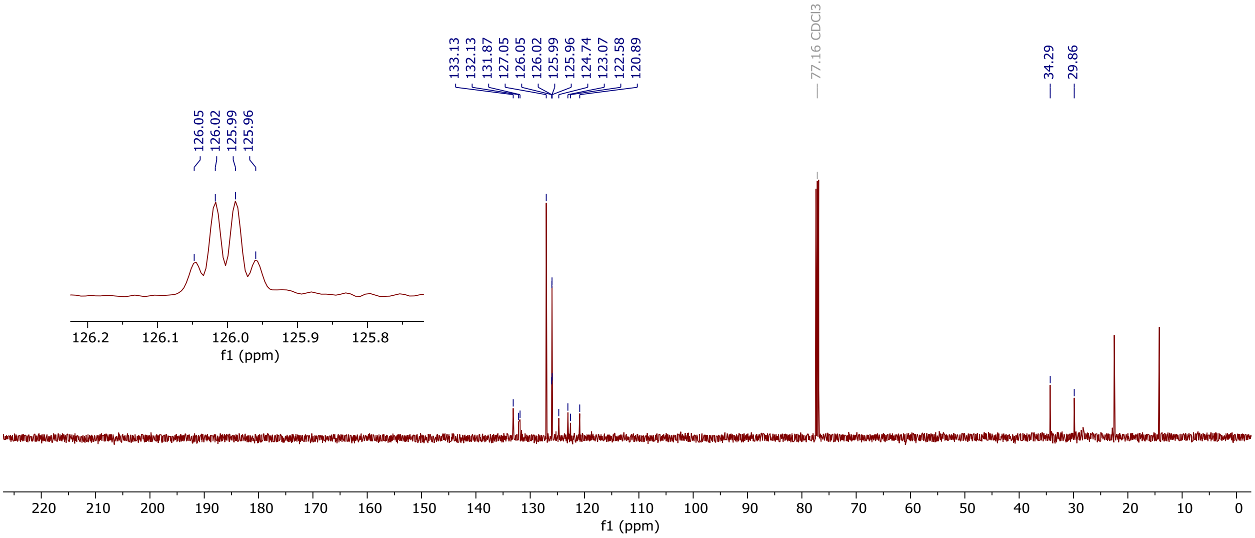

Fig. S29 ^{19}F NMR spectrum of **D11** in $CDCl_3$.

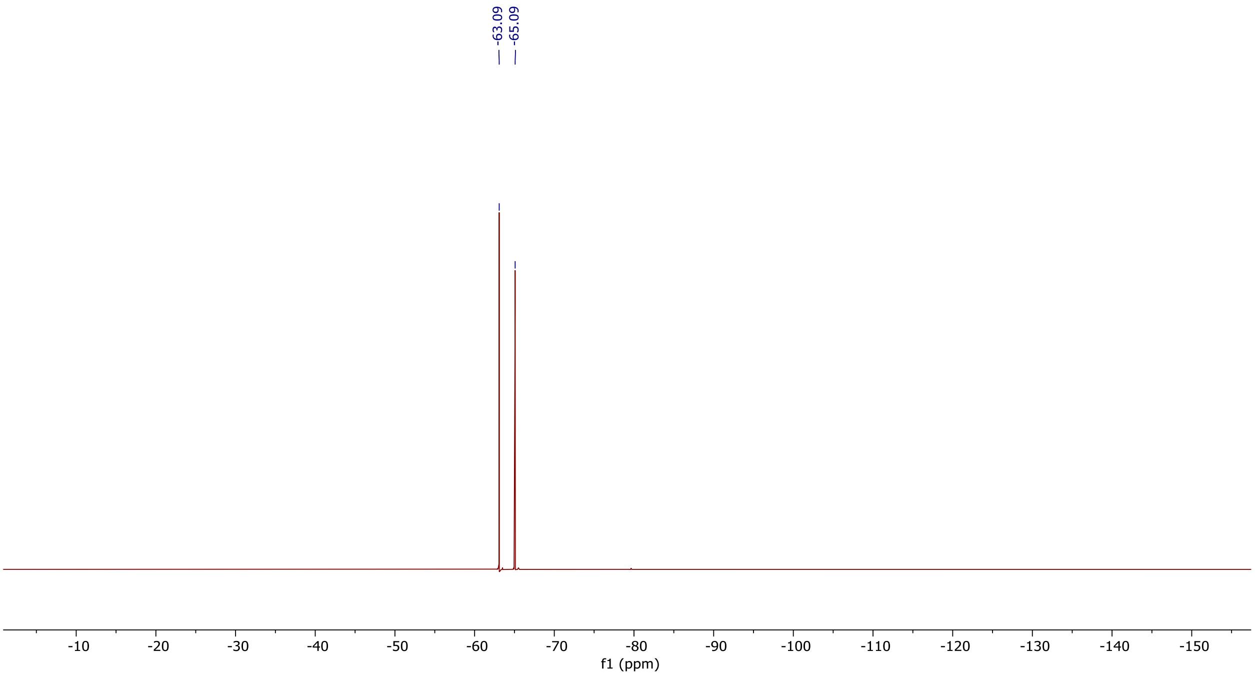

Synthesis of 4-[3-(Trifluoromethyl)-3*H*-diazirin-3-yl]benzaldehyde (D12).

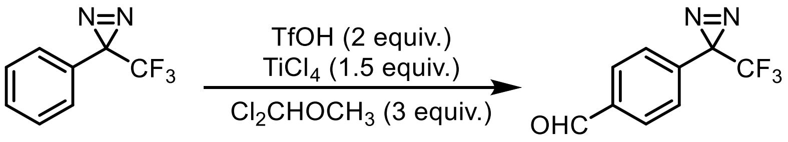

Following the procedure reported by Hatanaka,[18] in a flame dried two-neck flask under argon a yellow bright solid was prepared mixing TfOH (1.8 mL, 20 mmol, 2 equiv.) and $TiCl_4$ (1.6 mL, 15 mmol, 1.5 equiv.). The fuming solid was added portion wise to a second flame dried flask containing a mixture of 3-phenyl-3-(trifluoromethyl)-3*H*-diazirine **D4** (1.86 g, 10 mmol, 1 equiv.) and dichloromethyl methyl ether (2.7 mL, 30 mmol, 3 equiv.) (*Warning: acute toxicity, carcinogens*). The colour changed from pale yellow to dark brown and the reaction mixture started bubbling. The reaction was allowed to proceed for 1 h and quenched with a cold water at –20°C. The resulting mixture was neutralized with Na_2CO_3, left stirring for 1 hour, and extracted with pentane. The organic phase was washed with brine and dried over magnesium sulfate. After removal of solvent, the residue was purified by chromatography using pentane: diethyl ether (gradient 10:0 to 8:2, R_f = 0.62 (8:2)) to afford compound **D12** as a pale-yellow oil (899 mg, 4.2 mmol, 42%). The analytical data were in accordance with literature.[19] ^1H NMR (500 MHz, $CDCl_3$) δ 10.04 (s, 1H), 7.92 (d, *J* = 8.6 Hz, 2H), 7.35 (d, *J* = 8.1 Hz, 2H). ^{13}C NMR (126 MHz, $CDCl_3$) δ 191.19, 136.95, 135.41, 130.00, 127.14, 121.95 (d, *J* = 274.9 Hz), 22.49. ^{19}F NMR (283 MHz, $CDCl_3$) δ –64.82. IR (diamond-ATR) ν: 2833, 2744, 1707, 1609, 1342, 1187, 1153, 939, 737.

Fig. S30 ^1H NMR spectrum of **D12** in $CDCl_3$.

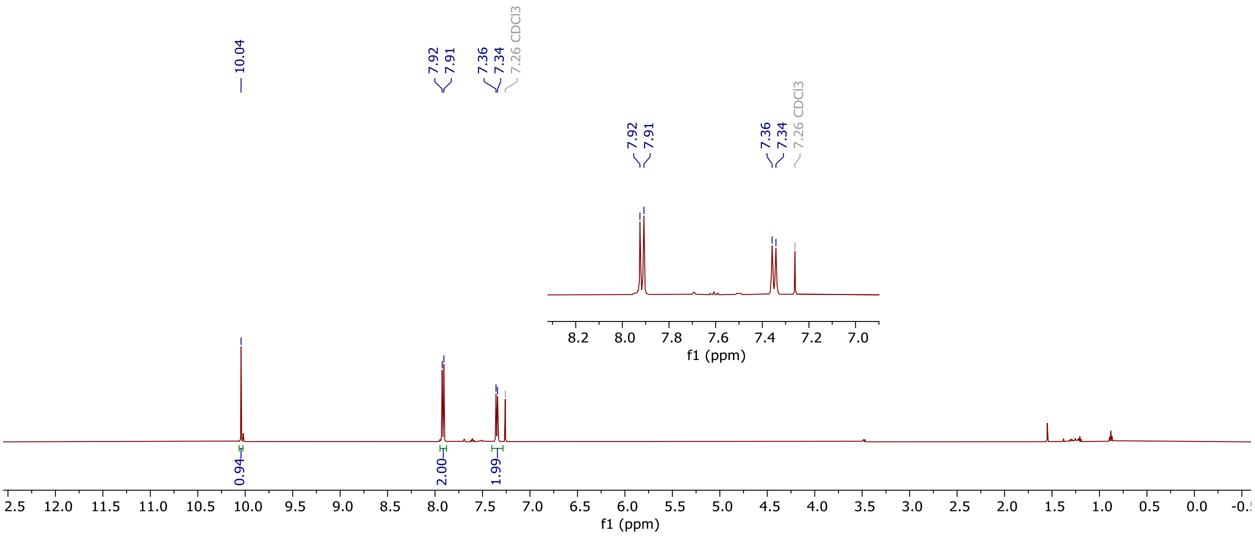

Fig. S31 ^{13}C NMR spectrum of **D12** in $CDCl_3$.

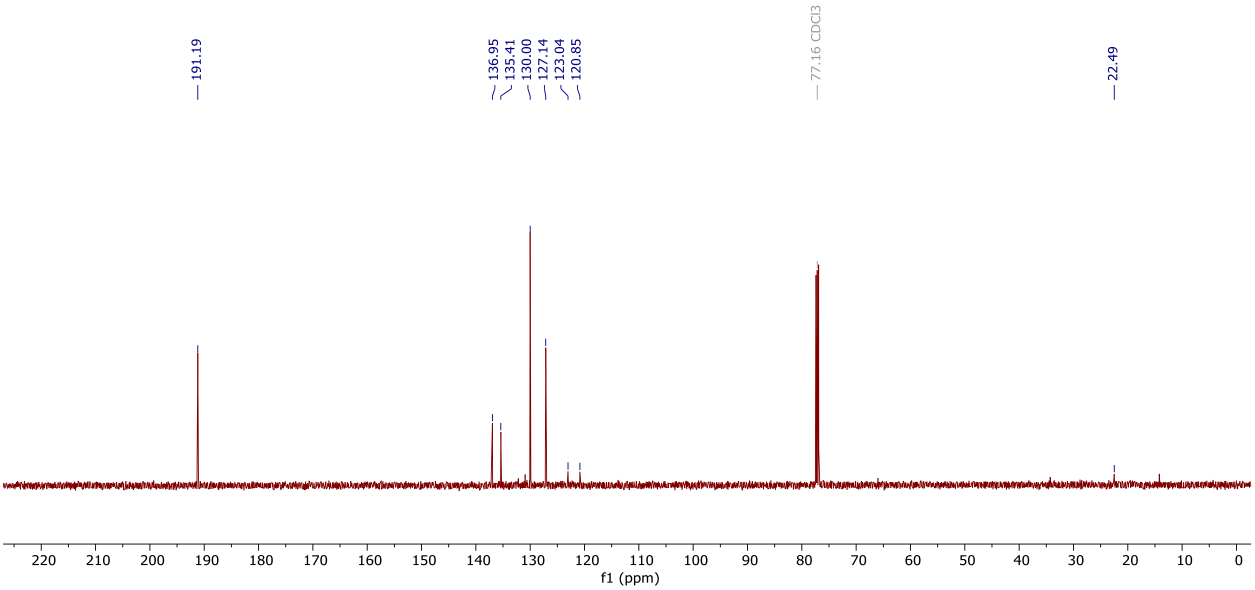

Fig. S32 ^{19}F NMR spectrum of **D12** in $CDCl_3$.

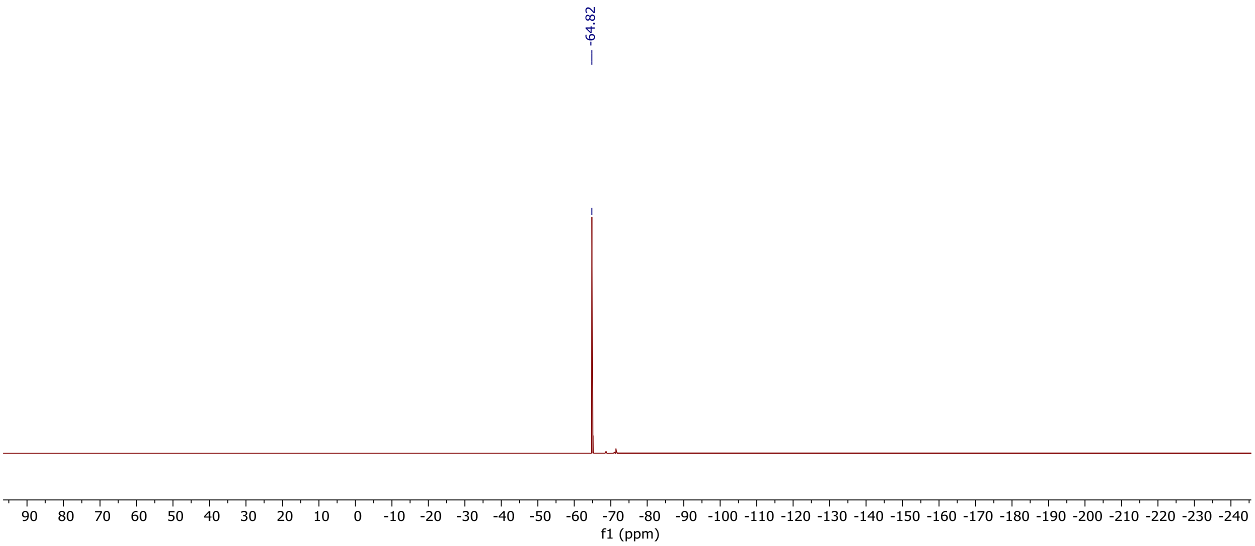

Synthesis of 2,2,2-trifluoro-1-(4-nitrophenyl)ethan-1-one (S31).

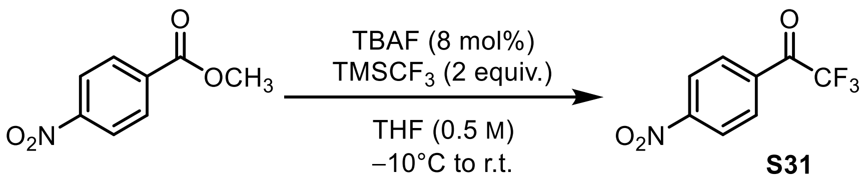

In a flame-dried flask, to a stirring solution of commercially available methyl 4-nitrobenzoate (1 g, 5.52 mmol, 1 equiv.) in distilled THF (11 mL) under argon atmosphere at –10 °C, $TMSCF_3$ (1.6 mL, 11 mmol, 2 equiv.) and then TBAF (0.44 mL, 1 M in THF, 8 mol%) were slowly added dropwise. The resulting solution was allowed to warm to room temperature with continued stirring for an hour and the colour changed from transparent to orange. The reaction was left stirring for 16 h and then quenched with NH_4Cl., extracted with diethyl ether (2 times) and the combined organic extracts were washed with brine and dried with sodium sulfate to afford the desired ketone **S31**, which was submitted to the next step without further purification. The analytical data are in accordance with the literature.[20] ^1H NMR (300 MHz, $CDCl_3$) 8.39 (d, *J* = 8.9 Hz, 1H), 8.23 (d, *J* = 9.0 Hz, 2H). ^{19}F NMR (283 MHz, $CDCl_3$) δ –71.92.

Synthesis of 2,2,2-trifluoro-1-(4-nitrophenyl)ethan-1-one oxime (S32) (mixture of *E*-, *Z*-isomers).

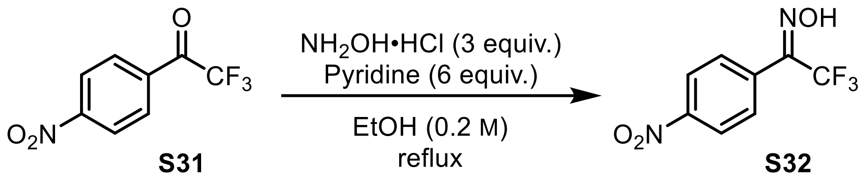

To a stirred solution of compound **S31** (1.2 g, 5.5 mmol, 1 equiv,) in ethanol (0.2 M), hydroxylamine hydrochloride (1.15 g, 16.5 mmol, 3 equiv.) and pyridine (2.6 mL, 33 mmol, 6 equiv.) were added and the reaction mixture was heated to reflux for 21 h. The mixture was then cooled to room temperature and the mixture was treated with 2 M HCl and extracted with diethyl ether (3 times). The combined organic layers were washed with distilled water until the pH of the washing layer became neutral, and then dried with sodium sulfate, filtered, and concentrated. The residue was dried under high vacuum for a prolonged time to afford the desired crude oxime **S32** (as a mixture of geometric isomers) as a yellow oil (1.3 g). The compound was submitted to the next step without further purification. ^{1}H NMR (300 MHz, $CDCl_3$) δ 8.30 (d, *J* = 8.9 Hz, 2H), 8.25 (d, *J* = 9.0 Hz, 1.6H, minor isomer), 7.68 (d, *J* = 8.6 Hz, 3.6H, major and minor isomers). ^{19}F NMR (283 MHz, $CDCl_3$) δ –62.28, –66.06.

Synthesis of 2,2,2-trifluoro-1-(4-nitrophenyl)ethan-1-one *O*-tosyl oxime (S33) (mixture of *E*-, *Z*-isomers).

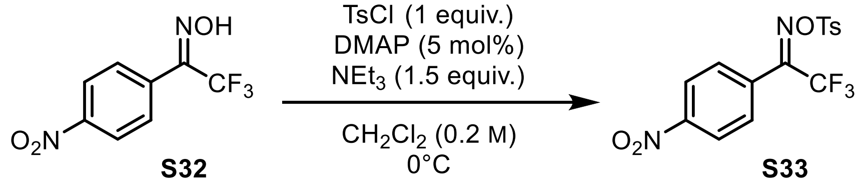

Compound **S32** (1.2 g, 5.5 mmol, 1 equiv.) was dissolved in CH_2Cl_2 (28 mL), and triethylamine (1.15 mL, 8.25 mmol, 1.5 equiv.), DMAP (34 mg, 0.275 mmol, 5 mol%) and tosyl chloride (1.05 g, 5.5 mmol, 1 equiv.) were successively added at 0°C. The ice bath was removed after 5 min, and the reaction mixture was stirred at room temperature for 1 h. The mixture was then treated with sat. aq. NH_4Cl and extracted with CH_2Cl_2. The combined organic extracts were dried with magnesium sulfate, filtered, and concentrated to afford the desired crude tosyl oxime **S33** (1.5 g), which was submitted to the next step without further purification. ^{1}H NMR (300 MHz, $CDCl_3$) δ 8.36 (d, *J* = 8.9 Hz, 2H), 8.30 (d, *J* = 8.9 Hz, 1.7H, minor isomer), 7.91 (d, *J* = 8.3 Hz, 1.6H, minor isomer), 7.89 (d, *J* = 8.3 Hz, 2H), 7.66 (d, *J* = 8.6 Hz, 1.8H, minor isomer), 7.59 (d, *J* = 8.8 Hz, 2H), 7.42 (d, *J* = 8.2 Hz, 2H), 7.40 (d, *J* = 8.3 Hz, 1.8H, minor isomer), 2.50 (s, 3H), 2.48 (s, 2.6H, minor isomer). ^{19}F NMR (283 MHz, $CDCl_3$) δ –61.43 (minor isomer), –66.82.

Synthesis of 3-(4-nitrophenyl)-3-(trifluoromethyl)diaziridine (S34).

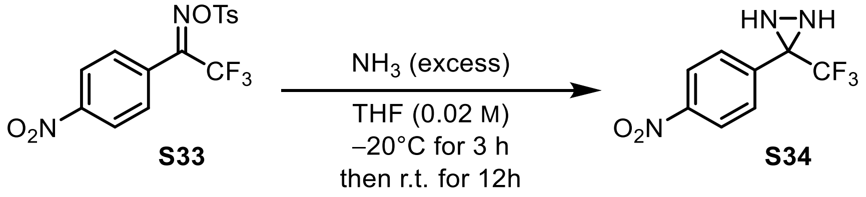

Tosyl oxime **S33** (1.5 g, 4.4 mmol, 1 equiv.) was transferred to a flame-dried 3-neck flask under argon in anhydrous THF (200 mL) and cooled to –20°C. Anhydrous gaseous ammonia was bubbled into the stirred solution for 3 h. Then, the reaction was left stirring for 12 h, during which time it was allowed to warm from –20°C to room temperature. The mixture was quenched with sat. aq. NH_4Cl and extracted with CH_2Cl_2 (3 times). The combined organic layers were washed with brine and then dried with sodium sulfate, filtered and concentrated to afford the desired crude diaziridine **S34** (930 mg), which was submitted to the next step without further purification. ^{1}H NMR (300 MHz, $CDCl_3$) δ 8.29 (d, *J* = 8.9 Hz, 2H), 7.84 (d, *J* = 8.6 Hz, 2H), 2.95 (d, *J* = 8.6 Hz, 1H), 2.33 (d, *J* = 9.0 Hz, 1H).^{19}F NMR (283 MHz, $CDCl_3$) δ –74.92.

Synthesis of 3-(4-nitrophenyl)-3-(trifluoromethyl)-3*H*-diazirine (D13).

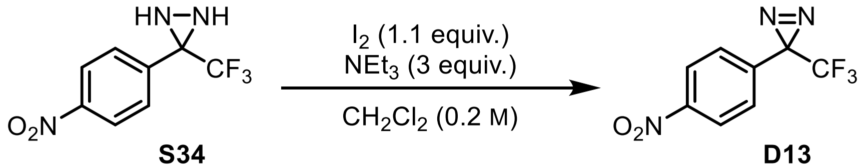

To a solution of the crude diaziridine **S34** (930 mg, 4 mmol, 1 equiv.) in CH_2Cl_2 (20 mL) at 0 °C were added successively triethylamine (1.7 mL, 12 mmol, 3 equiv.) and iodine (1.12 g, 4.4 mmol, 1.1 equiv.). The colored mixture was stirred at 0°C for 1 h. The mixture was diluted with CH_2Cl_2 and washed with sat. aq. sodium thiosulfate. The aqueous layer was re-extracted with CH_2Cl_2 (3 times). Then the combined organic extracts were washed with brine and dried with sodium sulfate, filtered, and concentrated. The residue was purified by silica gel column chromatography using pentane: EtOAc (0 to 20%) as eluent to afford the desired diazirine **D13** (632 mg, 2.7 mmol, 68%) as a pale-yellow oil, in accordance with literature data.[21] ^{1}H NMR (300 MHz, $CDCl_3$) δ 8.27 (d, *J* = 8.8 Hz, 2H), 7.37 (d, *J* = 8.6 Hz, 2H). ^{13}C NMR (126 MHz, $CDCl_3$) δ 148.57, 135.95, 127.58, 124.12, 120.59 (q, *J* = 274.8 Hz), 28.45 (q, *J* = 41.0 Hz). ^{19}F NMR (283 MHz, $CDCl_3$) δ –64.81. UV (*n*-hexane): $\lambda_{max,\ diazirine}$ = 340 nm. IR (diamond-ATR) ν: 3089, 1604, 1525, 1352, 1338, 1225, 1185, 1151, 1046. HRMS (ESI-) m/z [M – N_2 + HO^-] calculated for $[C_8H_5F_3NO_3]^-$: 220.0227, found: 220.0228.

Fig. S33 ^{1}H NMR spectrum of **D13** in $CDCl_3$.

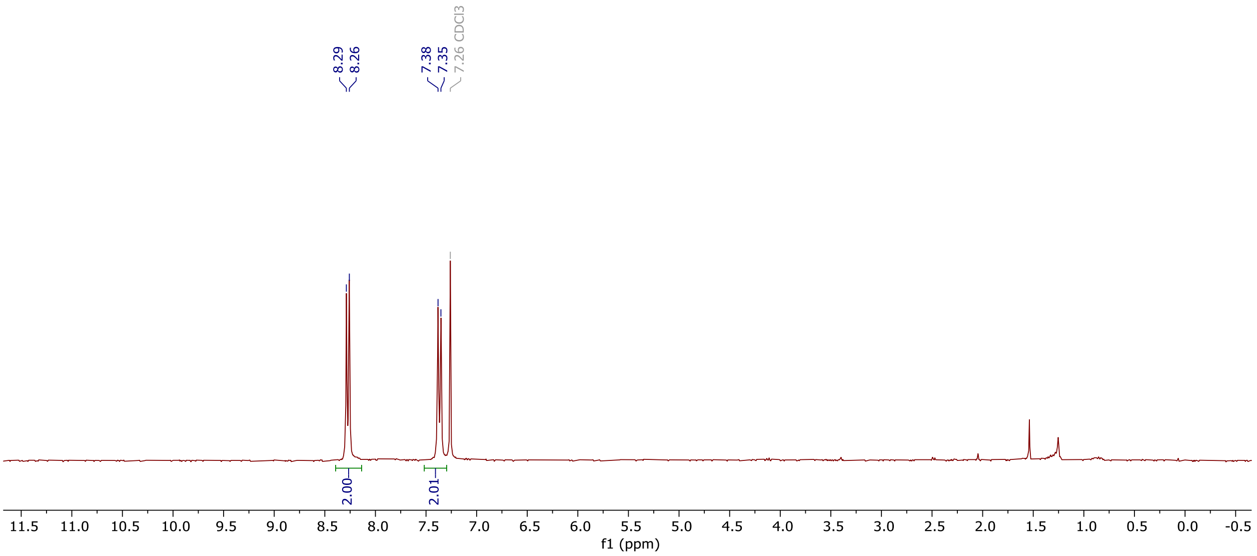

Fig. S34 ^{13}C NMR spectrum of **D13** in $CDCl_3$.

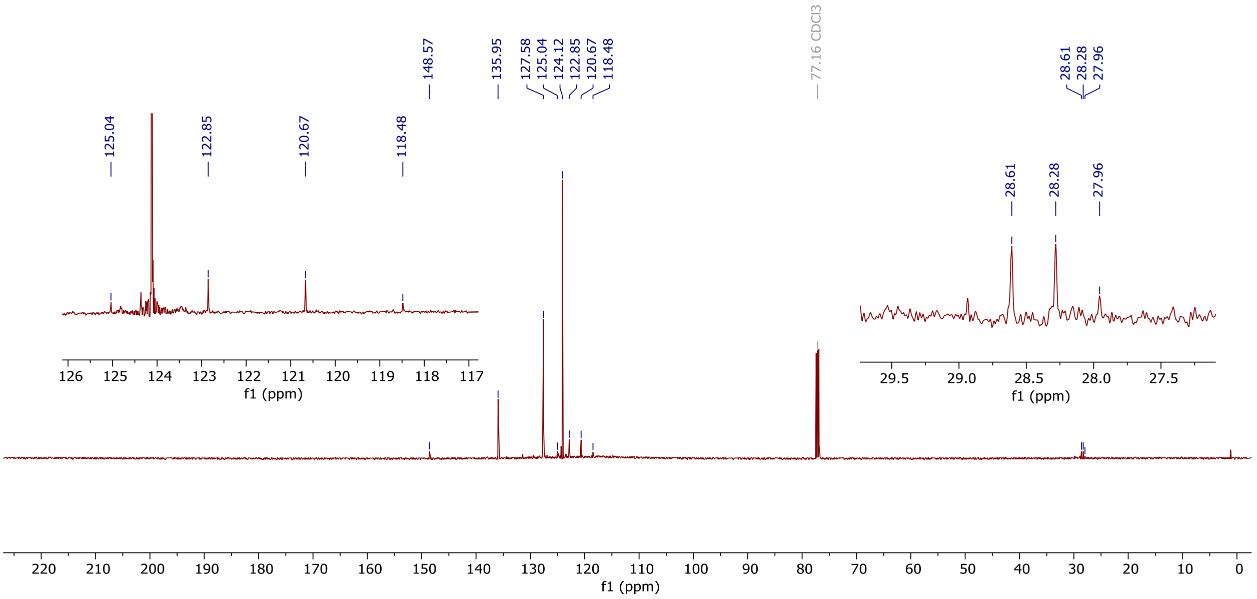

Fig. S35 ^{19}F NMR spectrum of **D13** in $CDCl_3$.

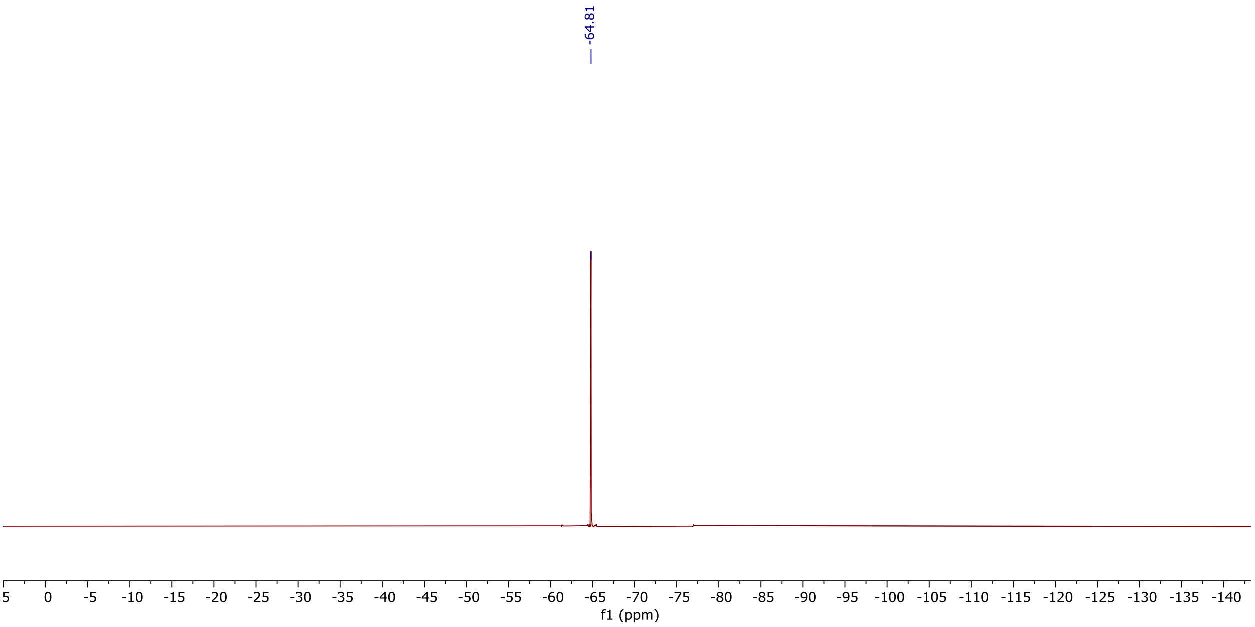

Synthesis of 3-(4-bromophenyl)-3-chloro-3*H*-diazirine (D14).

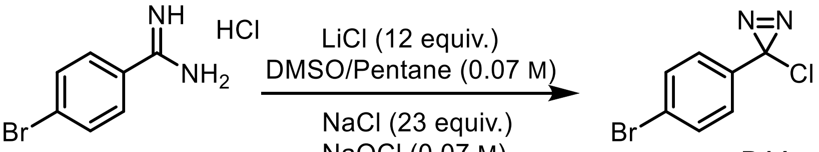

In a three-neck flask fitted with a dropping funnel and thermometer, to a mixture of lithium chloride (1 g, 25.4 mmol, 12 equiv.) and DMSO (15 mL), commercially available 4-bromobenzamidine hydrochloride (500 mg, 2.21 mmol, 1 equiv.) and pentane (15 mL) were added and the reaction was cooled to 0 °C. Aqueous NaOCl solution (30 mL, 8.25% hypochlorite) saturated with sodium chloride (2.8 g, 48.7 mmol, 23 equiv.) was added slowly, maintaining the temperature between 35 and 40 °C with an ice bath. After 5 min, 15 mL of DMSO, 15 mL of pentane, and 40 mL of aqueous NaOCl were added, and the mixture was allowed to stir for 1 h at room temperature. The reaction solution was poured into a separatory funnel containing ice water, and the pentane layer was separated, washed with brine (4 times), and dried over $CaCl_2$. The crude was purified by silica gel column chromatography using pentane (R_f = 0.72), as eluent mixture to afford the desired diazirine **D14** (351 mg, 1.51 mmol, 71%) as a colourless oil, in accordance with literature data.[22] ^{1}H NMR (300 MHz, $CDCl_3$) δ 7.53 (d, *J* = 8.9 Hz, 2H), 6.98 (d, *J* = 8.9 Hz, 2H). ^{13}C NMR (126 MHz, $CDCl_3$) δ 134.92, 131.87, 127.72, 124.00, 29.86. IR (diamond-ATR) ν: 2925, 2853, 1594, 1578, 1491, 1398, 1080, 1020, 905, 814, 536. GC–MS *m/z* (% relative intensity) [ion]: 243/245/247 (42) $[M - N_2 + CH_3CN]^+$, 208/210 (52) $[C_7H_4Br + CH_3CN]^+$, 202/204/206 (68) $[C_7H_4BrCl]^+$, 167/169 (20) $[C_7H_4Br]^+$, 164/166 (48) $[C_7H_4Cl + CH_3CN]^+$, 155/157 (18) $[C_6H_4Br]^+$, 123/125 (100) $[C_7H_4Cl]^+$, 88 (50) $[C_7H_4]^+$, 76 (44) $[C_6H_4]^+$. [M = $C_7H_4BrClN_2$ (229.92)].

Fig. S36 ^{1}H NMR spectrum of **D14** in $CDCl_3$.

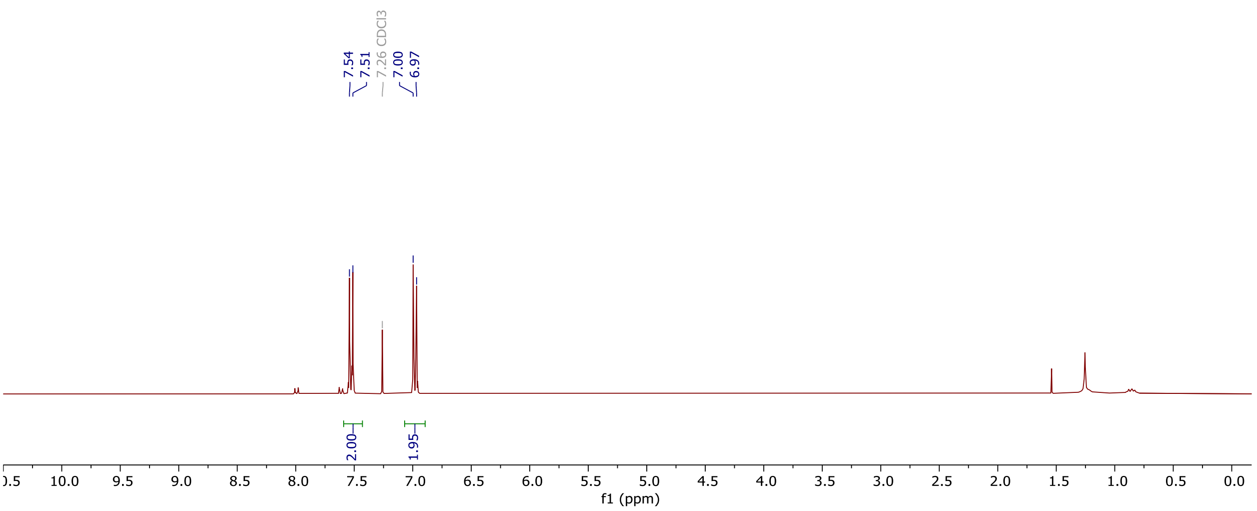

Fig. S37 ^{13}C NMR spectrum of **D14** in $CDCl_3$.

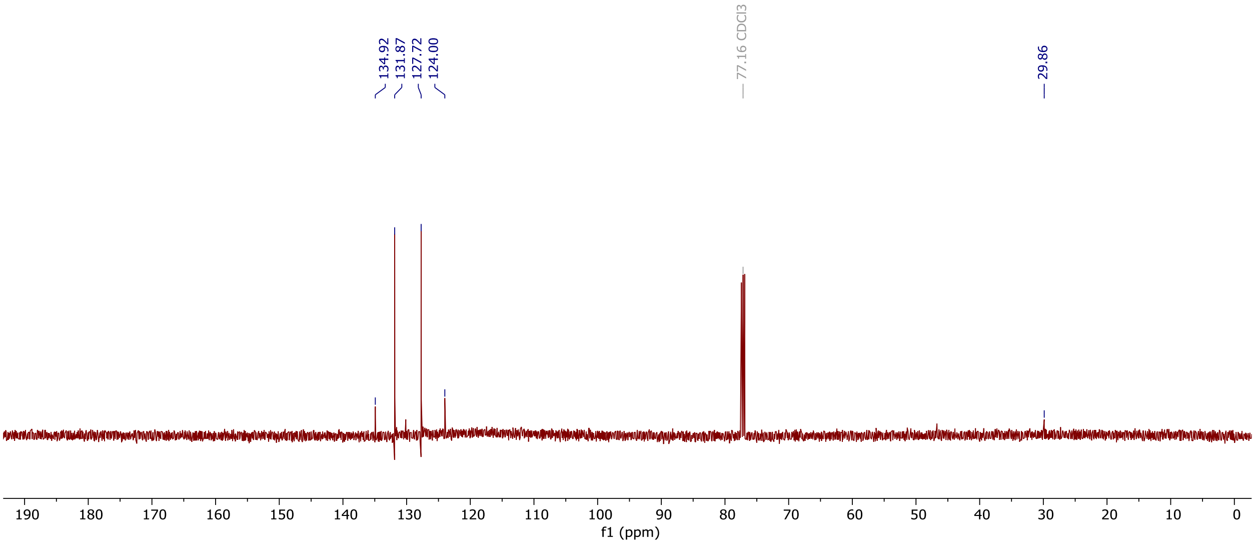

Synthesis of 3-(4-bromophenyl)-3*H*-diazirine (D15).

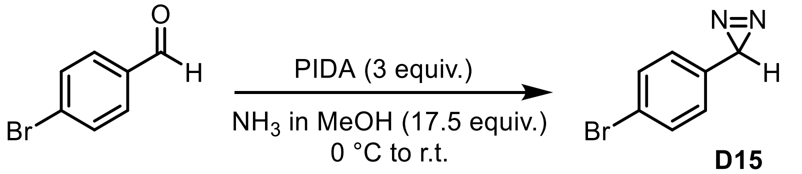

D15

(Diacetoxyiodo)benzene (PIDA, 7.5 mmol, 3 equiv.) was added in one portion to a stirred solution of 4-bromobenzaldehyde (2.5 mmol, 1 equiv.) in NH_3 in MeOH 7 M (6.3 mL, 17.5 equiv.) at 0°C under argon. After 30 minutes at 0°C, the batch was allowed to reach room temperature and was left stirred for 2 h. After completion (monitored by TLC and ^{1}H NMR), the batch was concentrated under reduced pressure and the crude was purified by flash chromatography on silica gel. Pentane was employed to afford diazirine **D15** (122 mg, 0.618 mmol, 25% yield) as a pale-yellow oil. ^{1}H NMR (500 MHz, $CDCl_3$) δ 7.44 (d, *J* = 8.5 Hz, 2H), 6.78 (d, *J* = 8.5 Hz, 2H), 2.03 (s, 1H). ^{13}C NMR (126 MHz, $CDCl_3$) δ 135.55, 131.63, 126.86, 122.23, 23.17. IR (diamond-ATR) ν: 3054, 1622, 1608, 1490, 1013, 831, 818, 782, 535. GC–MS *m/z* (% relative intensity) [ion]: 209/211 (25) $[M - N_2 + CH_3CN]^+$, 208/210 (25) $[C_7H_4Br + CH_3CN]^+$, 168/170 (10) $[C_7H_5Br]^+$, 130 (64) $[C_7H_5 + CH_3CN]^+$, 89 (100) $[C_7H_5]^+$. [M = $C_7H_5BrN_2$ (195.96)].

Fig. S38 ^1H NMR spectrum of **D15** in $CDCl_3$.

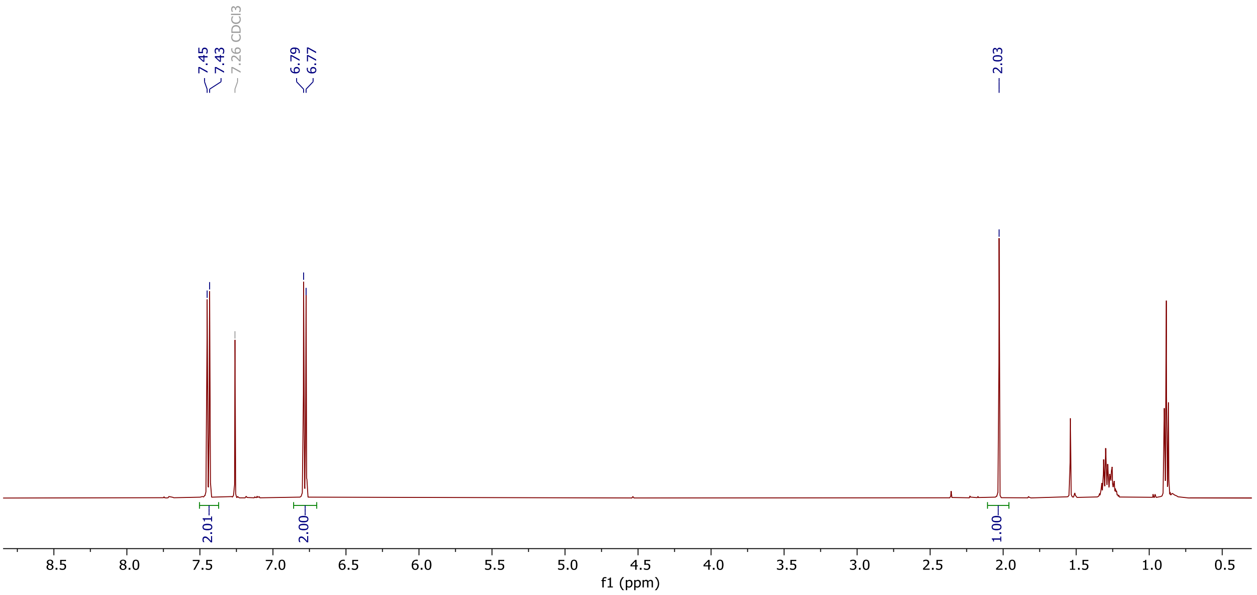

Fig. S39 ^{13}C NMR spectrum of **D15** in $CDCl_3$.

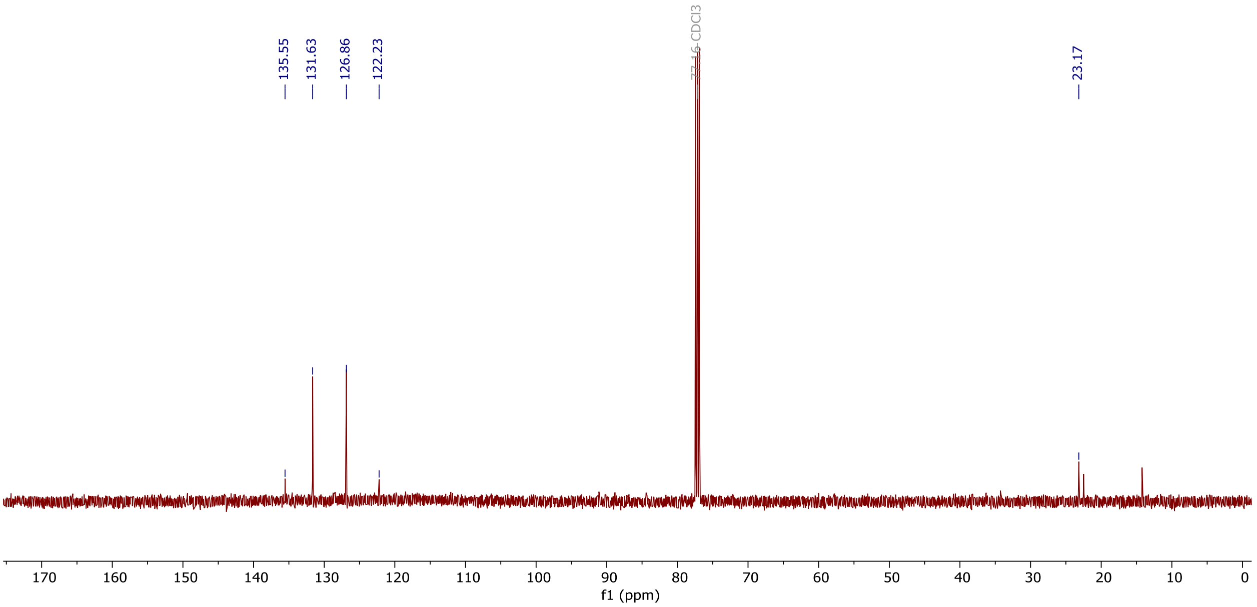

Synthesis of 3-(4-bromophenyl)-3-methyl-3*H*-diazirine (D16).

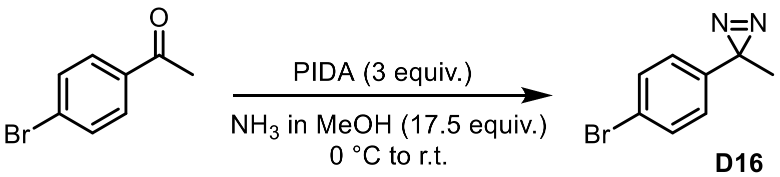

D16

(Diacetoxyiodo)benzene (PIDA, 10.5 mmol, 3 equiv.) was added in one portion to a stirred solution of 4'-bromoacetophenone (3.5 mmol, 1 equiv.) in NH_3 in MeOH 7M (8.8 mL, 17.5 equiv.) at 0°C under argon. After 30 minutes at 0°C, the batch was allowed to reach room temperature and was left stirred for 2h. After completion (monitored by TLC and ^1H NMR), the batch was concentrated under reduced pressure and the crude was purified by flash chromatography on silica gel. Pentane was employed to afford diazirine **D16** (150 mg, 0.711 mmol, 20% yield) as a pale-yellow oil. ^1H NMR (500 MHz, Chloroform-*d*) δ 7.51 – 7.42 (m, 2H), 6.85 – 6.75 (m, 2H), 1.50 (s, 3H). ^{13}C NMR (126 MHz, $CDCl_3$) δ 138.96, 131.48, 127.24, 121.82, 26.10, 17.68. IR (diamond-ATR) ν: 2964, 2930, 1607, 1591, 1494, 1404, 1082, 1008, 829, 728, 540. HRMS (ESI+) m/z [M+H] calculated for $[C_8H_8BrN_2]^+$: 210.9865, found: 210.9864.

Fig. S40 ^{1}H NMR spectrum of **D16** in $CDCl_3$.

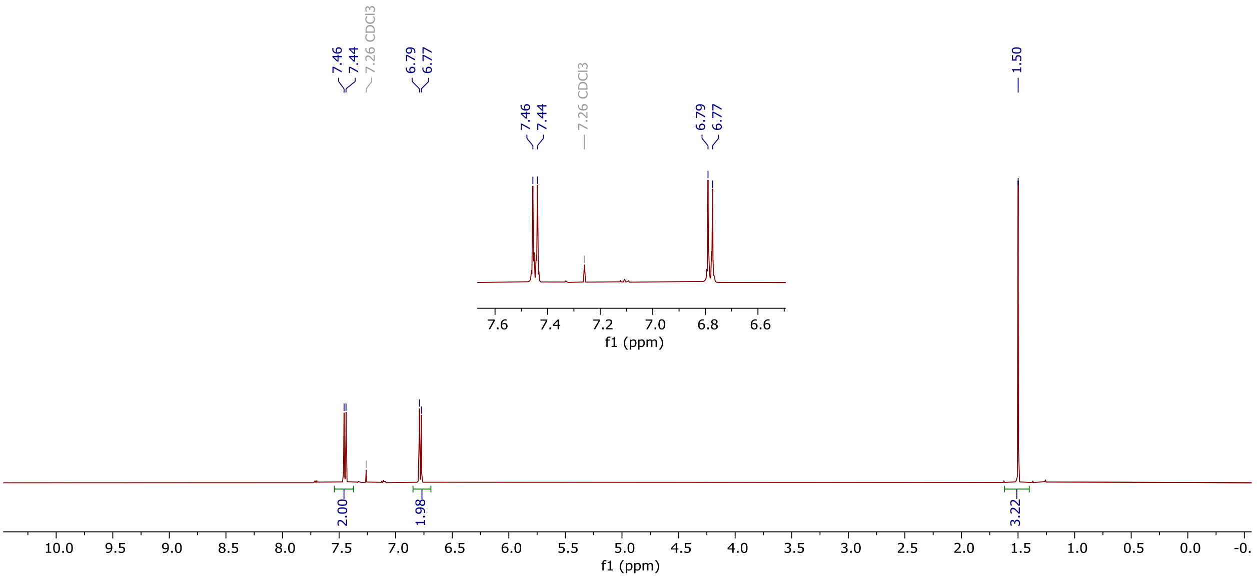

Fig. S41 ^{13}C NMR spectrum of **D16** in $CDCl_3$.

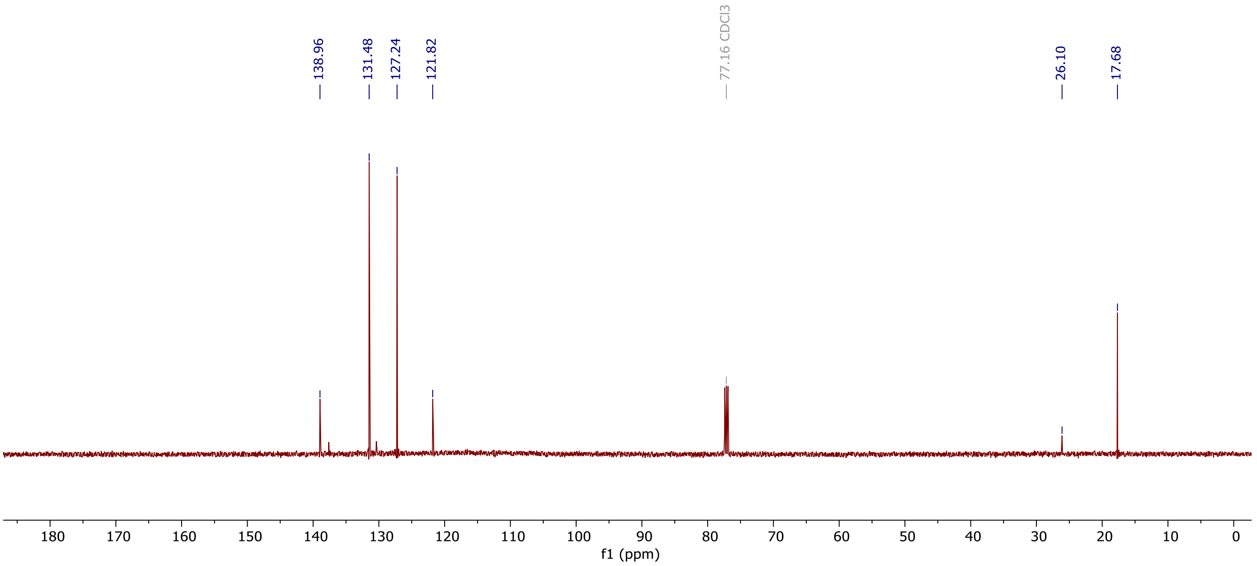

Synthesis of 3-(4-bromophenyl)-3-fluoro-3*H*-diazirine (D17).

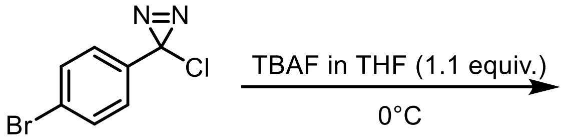

D14 **D17**

To a stirred solution of TBAF (4.4 mL, 1 M in THF, 1.1 equiv.) under argon at 0°C, 3-(4-bromophenyl)-3-chloro-3H-diazirine **D14** (107 mg, 0.4 mmol, 1 equiv.) was added dropwise. The reaction mixture was stirred for 2 h and then quenched with NH_4Cl. The resulting mixture was extracted with pentane (3 times). The combined organic layers were dried over magnesium sulfate. The desired diazirine **D17** was afforded as a clean colourless oil (86 mg, 0.39 mmol, 99%), in accordance with literature data.[23] ^{1}H NMR (500 MHz, $CDCl_3$) δ 7.56 (d, *J* = 7.9 Hz, 2H), 6.90 (d, *J* = 8.6 Hz, 2H). ^{13}C NMR (126 MHz, $CDCl_3$) δ 129.39 (q, *J* = 258.0 Hz). ^{13}C NMR (126 MHz, $CDCl_3$) δ 132.66, 132.11, 129.39 (q, *J* = 258.0 Hz), 126.37 (q, *J* = 4.1 Hz), 124.53, 29.85. ^{19}F NMR (283 MHz, $CDCl_3$) δ –154.09. IR (diamond-ATR) ν: 2957, 2925, 2854, 1601, 1491, 1400, 1021, 817, 552, 537. GC–MS *m/z* (% relative intensity) [ion]: 227/229 (80) [M – N_2 + CH_3CN]$^+$, 212/214 (10) [M – N_2 + CN]$^+$, 186/188 (10) [M – N_2]$^+$, 148 (100) [C_7H_4F + CH_3CN]$^+$, 107 (95) [C_7H_4F]$^+$, 76 (50) [C_6H_4]$^+$. [M = $C_7H_4BrFN_2$ (213.95)].

Fig. S42 ^{1}H NMR spectrum of **D17** in $CDCl_3$.

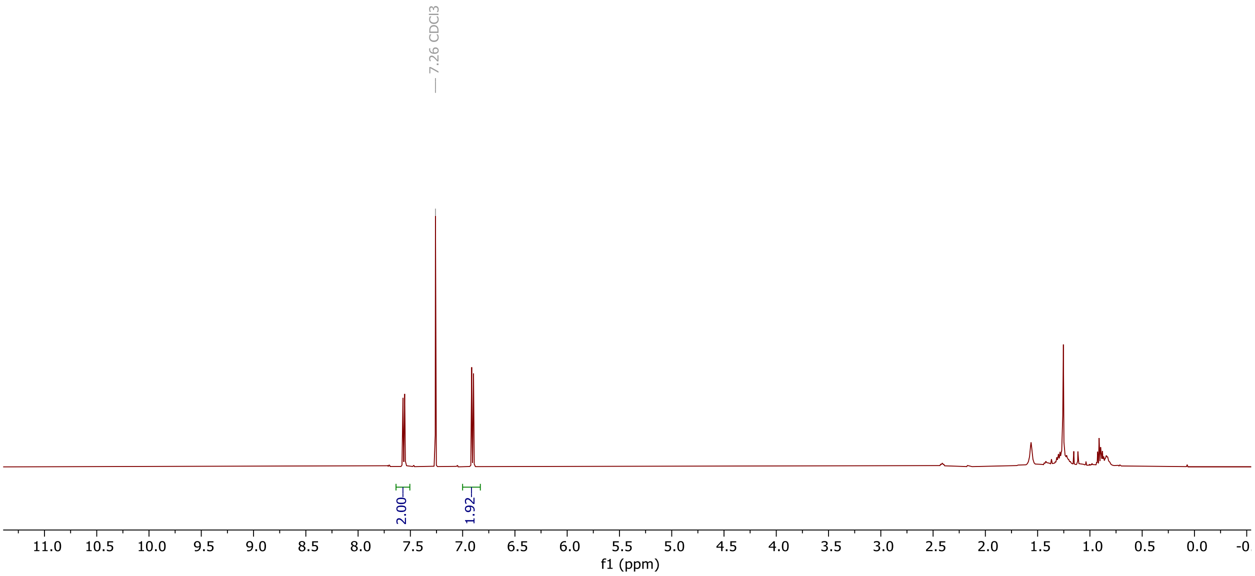

Fig. S43 ^{13}C NMR spectrum of **D17** in $CDCl_3$.

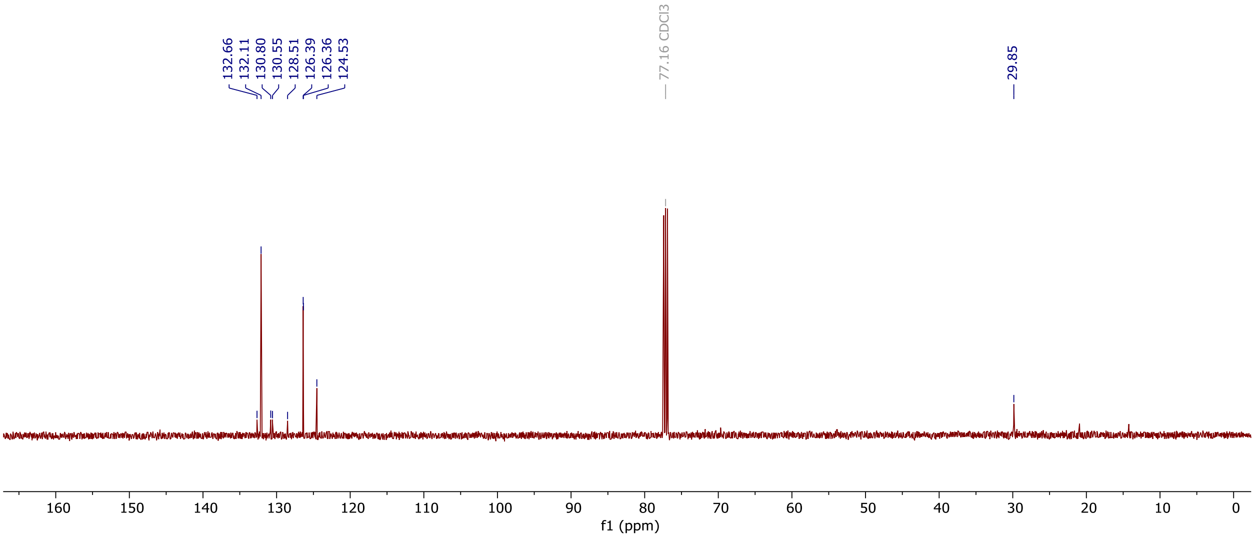

Fig. S44 ^{19}F NMR spectrum of **D17** in $CDCl_3$.

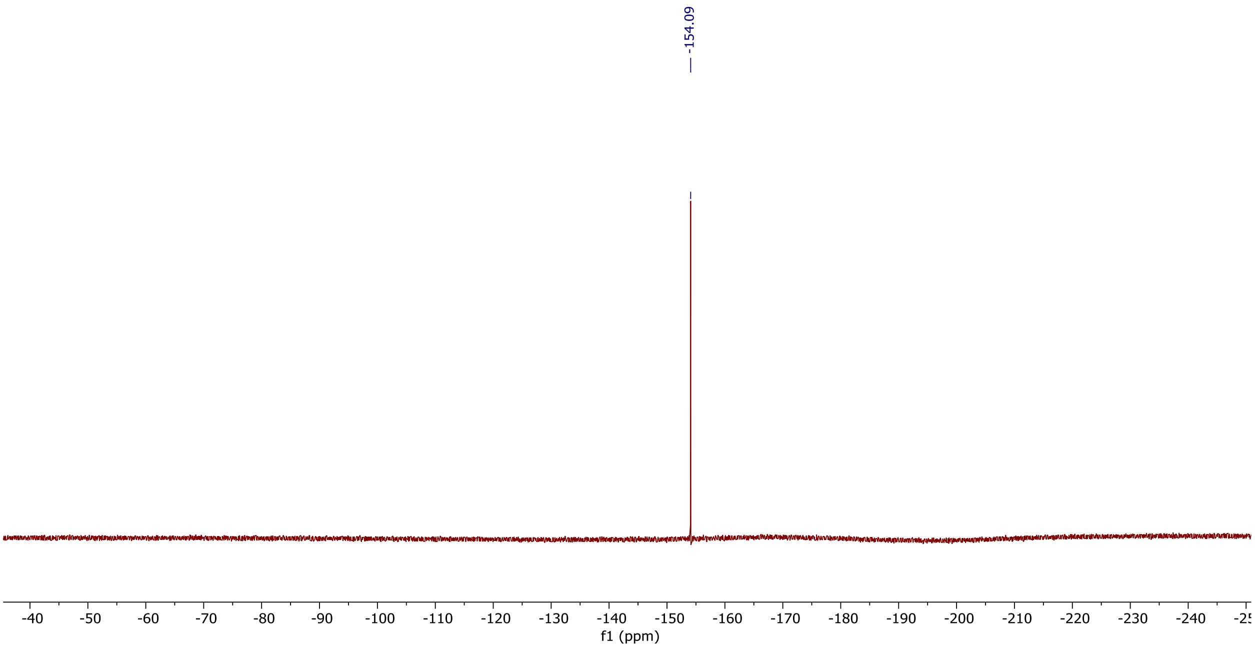

General protocols for insertion reactions

A) **Thermal C–H insertion reaction**: a 15 mM solution of the desired diazirine in anhydrous, degassed cyclohexane was prepared in a heavy wall pressure vessel with PTFE bushing as pressure seal. Glassware was previously flame dried and kept under argon pressure. After sealing the vessel, the reaction was immerged in an oil bath at 140 °C and stirred at that temperature for 2 h. After cooling the mixture to room temperature, the reaction was transferred into a round bottom flask and concentrated in vacuo to provide crude product.

B) **Photochemical C–H insertion reaction**: a 15 mM solution of the desired diazirine in anhydrous, degassed cyclohexane was prepared in a long neck round bottom flask. Glassware was previously flame dried and kept under argon pressure. The flask was sealed with a septum and placed under a balloon of argon to maintain an inert atmosphere. The flask was suspended into a Rayonet UV chamber that was equipped with eight 350 nm UV lamps and an operating fan. The reaction contents were irradiated for 4 h. The reaction was then concentrated in vacuo to provide crude product.

C) **Thermal insertion reaction in a "wet environment"**: a 15 mM solution of the desired diazirine in a specific, moisture-containing solvent was prepared in a heavy wall pressure vessel with PTFE bushing as pressure seal. The reaction was prepared under air. After sealing the vessel, the reaction was immerged in an oil bath at 140 °C and stirred at that temperature for 2 h. After cooling the mixture to room temperature, the reaction was transferred into a round bottom flask and concentrated in vacuo to provide crude product.

D) **Photochemical insertion reaction in a "wet environment"**: a 15 mM solution of the desired diazirine in a specific, moisture-containing solvent was prepared in a long neck round bottom flask. The reaction was prepared under air. The flask was sealed with a septum and suspended into a Rayonet UV chamber that was equipped with eight 350 nm UV lamps and an operating fan. The reaction contents were irradiated for 2 h. The reaction was then concentrated in vacuo to provide crude product.

Crude ^{19}F NMR ($CDCl_3$) spectra were collected and compared to show the effect of aryl substituent electronics on the type of insertion products. The crude materials were purified by chromatography using petroleum ether as eluent, unless otherwise specified. Several 2–4 mL fractions were collected in 12 x 75 mm test tubes. Fractions that contained the desired product were combined and concentrated.

Insertion reactions in cyclohexane

Preparation of 1-(1-cyclohexyl-2,2,2-trifluoroethyl)-4-(trifluoromethyl)benzene (S35).

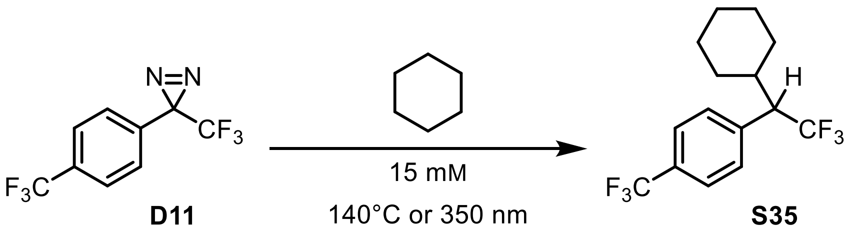

Following the above general protocols (**A** and **B**) for insertion experiments, the reaction was performed using: **A) Thermal insertion:** diazirine **D11** (55.5 mg, 0.218 mmol) dissolved in cyclohexane (14 mL). The desired product **S35** (4 mg, 0.013 mmol, 6%) was isolated following chromatography. **B) Photochemical insertion**: diazirine **D11** (29 mg, 0.114 mmol) dissolved in cyclohexane (7.6 mL). The desired product **S35** (9.2 mg, 0.029 mmol, 25%) was isolated following chromatography. ^{1}H NMR (500 MHz, $CDCl_3$) δ 7.60 (d, *J* = 8.1 Hz, 2H), 7.36 (d, *J* = 8.0 Hz, 2H), 3.12 (qd, *J* = 9.6, 8.5 Hz, 1H), 2.05 – 1.93 (m, 2H), 1.82 – 1.74 (m, 1H), 1.68 – 1.58 (m, 2H), 1.49 – 1.41 (m, 1H), 1.36 – 1.30 (m, 1H), 1.15 – 1.03 (m, 3H), 0.82 – 0.75 (m, 1H). ^{13}C NMR (126 MHz, $CDCl_3$) δ 129.71, 125.59 (q, *J* = 3.8 Hz), 56.21 (q, *J* = 26.0 Hz), 38.65, 31.54, 30.82, 29.85, 26.21, 26.12, 26.02. ^{19}F NMR (471 MHz, $CDCl_3$) δ –62.65 (*p*-CF_3), –63.23. IR (diamond-ATR) ν: 2954, 2920, 2851, 1464, 1267, 1168, 1131, 1116, 730. GC–MS *m/z* (% relative intensity) [ion]: 310 (10) $[M]^+$, 291 (8) $[C_{15}H_{16}F_5]^+$, 177 (14) $[C_8H_5F_4]^+$, 159 (8) $[C_8H_6F_3]^+$, 83 (100) $[C_6H_{11}]^+$, 67 (15) $[C_5H_7]^+$, 55 (96) $[C_4H_7]^+$. [M = $C_{15}H_{16}F_6$ (310.12)].

Fig. S45 ^{1}H NMR spectrum of cyclohexane adduct **S35** in $CDCl_3$.

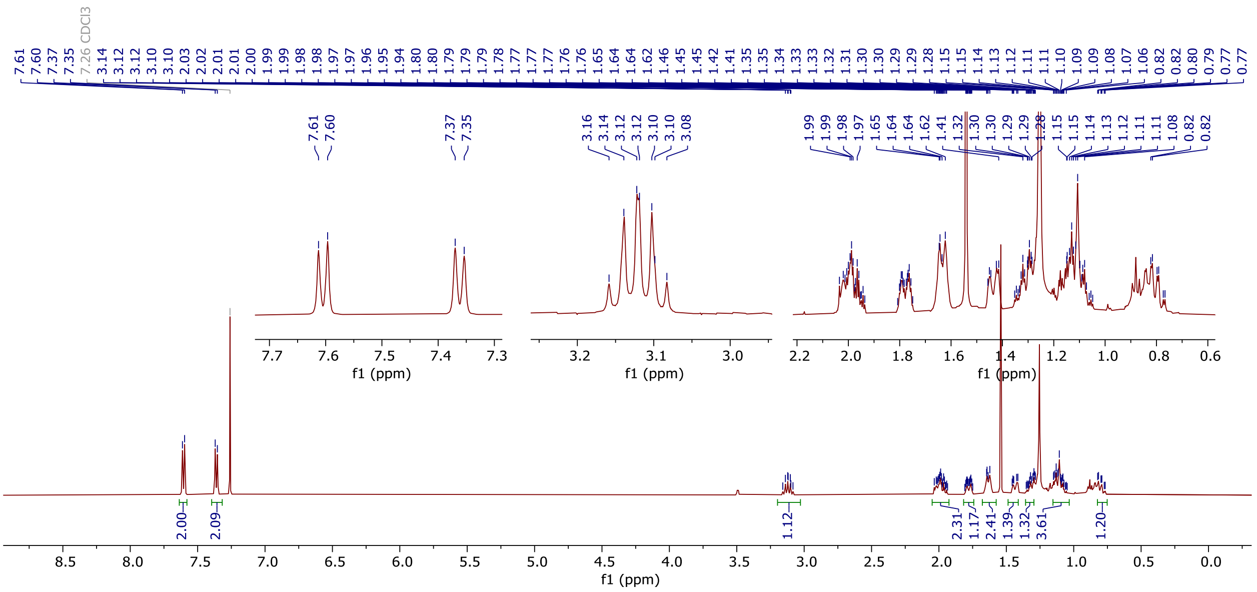

Fig. S46 ^{13}C NMR spectrum of cyclohexane adduct **S35** in $CDCl_3$.

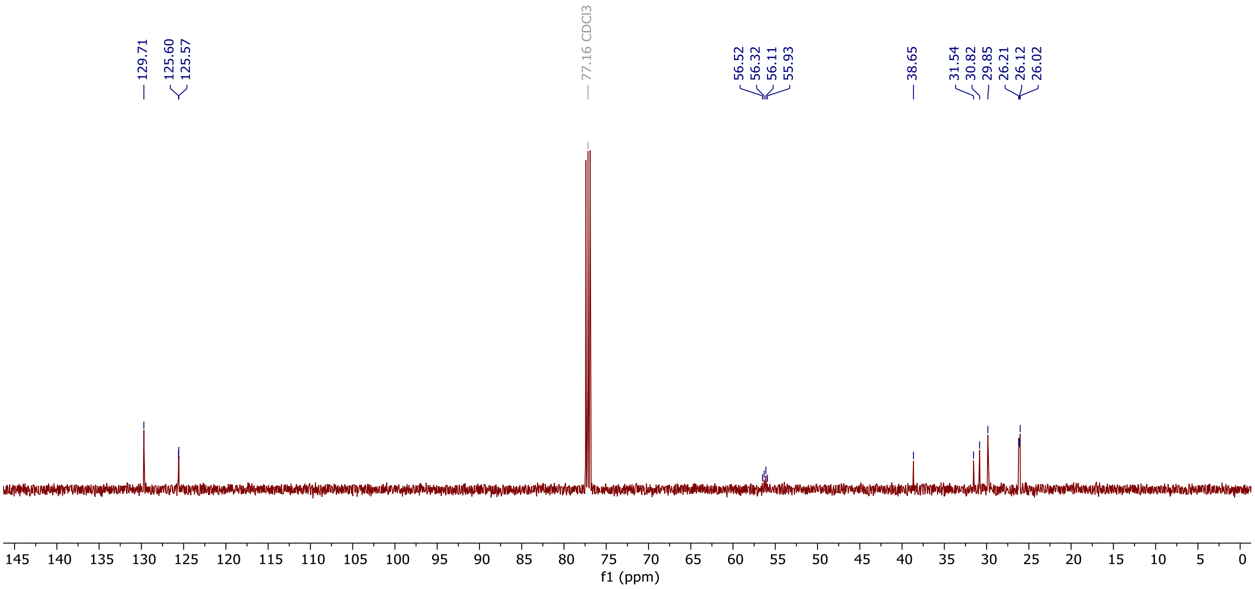

Fig. S47 ^{19}F NMR spectrum of cyclohexane adduct **S35** in $CDCl_3$.

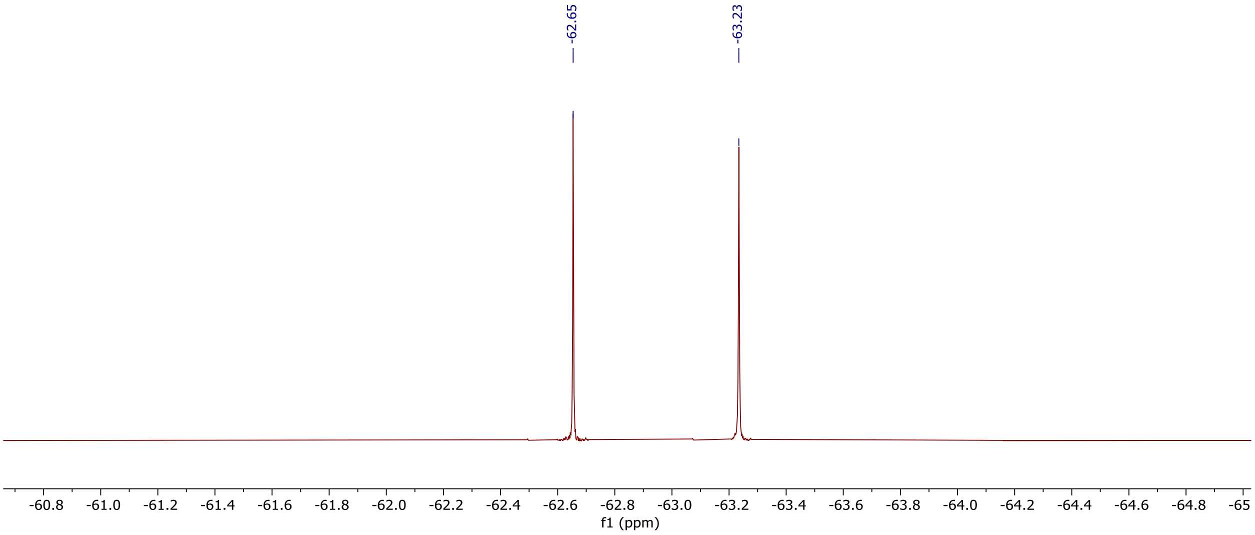

Fig. S48 Crude ^{19}F NMR spectrum under thermal insertion conditions.

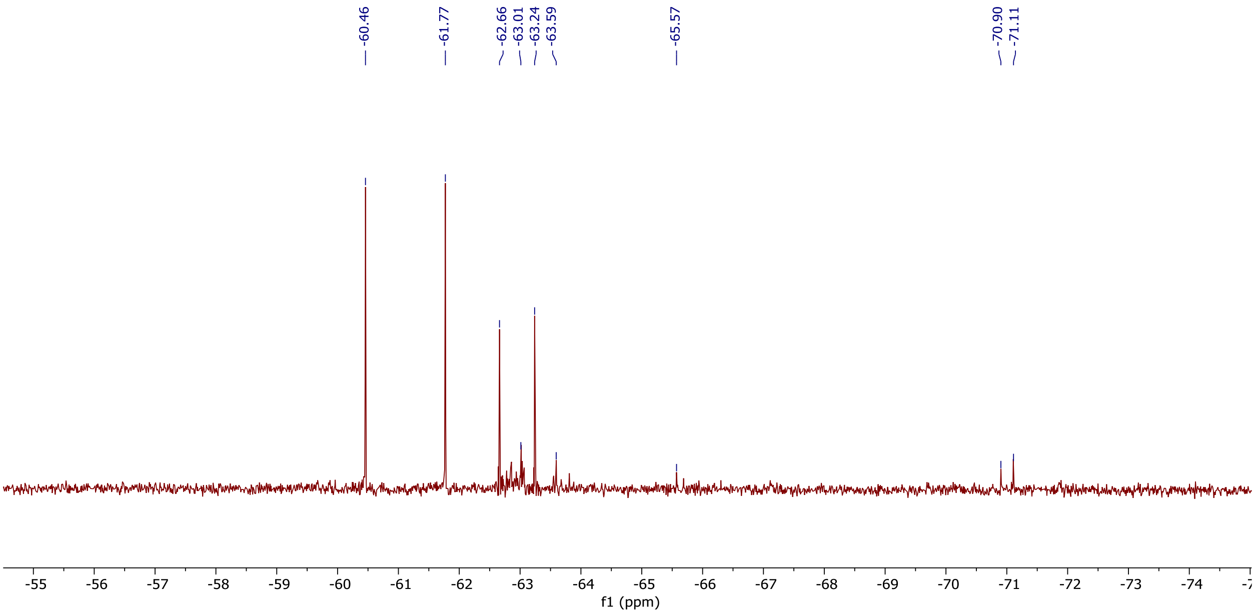

Fig. S49 Crude ^{19}F NMR spectrum under photochemical insertion conditions.

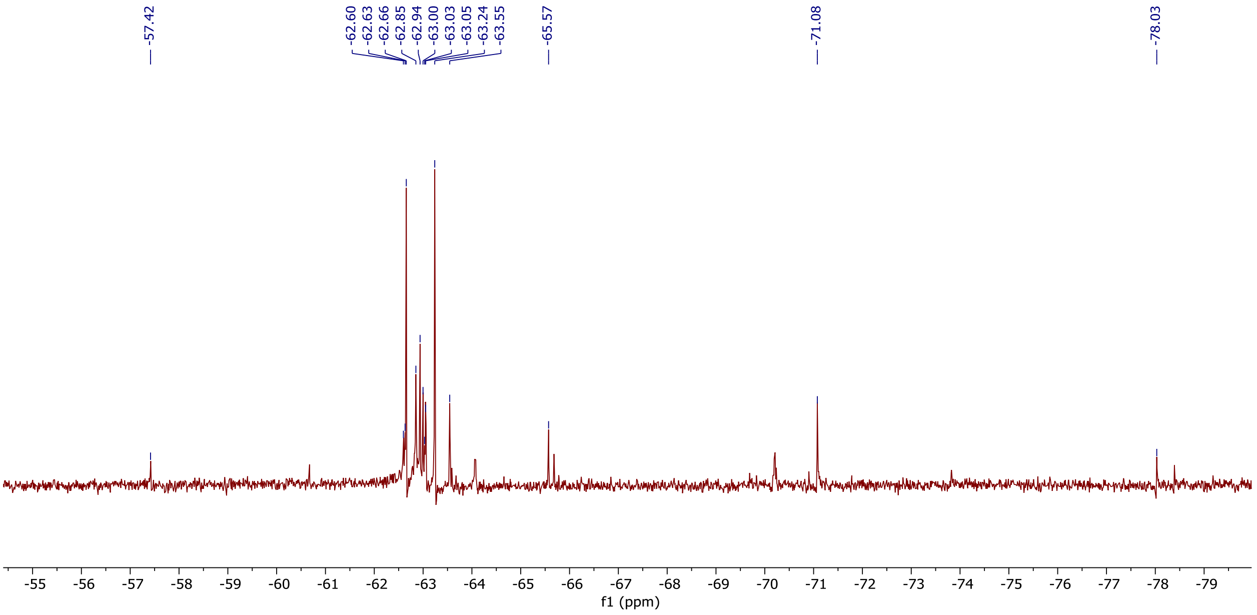

Preparation of (1-cyclohexyl-2,2,2-trifluoroethyl)benzene (S36).

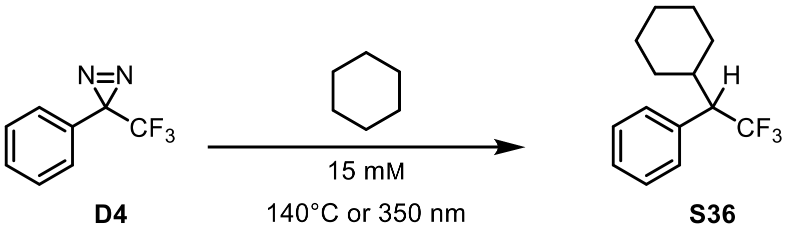

Following the above general protocols (**A** and **B**) for insertion experiments, the reaction was performed using: **A) Thermal insertion:** diazirine **D4** (66 mg, 0.35 mmol) dissolved in cyclohexane (24 mL). The desired product **S36** (12.7 mg, 0.053 mmol, 15%) was isolated following chromatography. **B) Photochemical insertion**: diazirine **D4** (66 mg, 0.35 mmol) dissolved in cyclohexane (24 mL). The desired product **S36** (30.1 mg, 0.124 mmol, 35%) was isolated following chromatography. The analytical data were in accordance with literature.[24] ^{1}H NMR (300 MHz, $CDCl_3$) δ 7.39 – 7.28 (m, 3H), 7.26 – 7.20 (m, 2H), 3.03 (qd, J = 10.2, 8.0 Hz, 1H), 2.06 – 1.98 (m, 1H), 1.98 – 1.89 (m, 1H), 1.84 – 1.71 (m, 1H), 1.70

– 1.57 (m, 2H), 1.52 – 1.41 (m, 1H), 1.39 – 1.27 (m, 1H), 1.21 – 1.03 (m, 3H), 0.85 – 0.72 (m, 1H). ^{13}C NMR (126 MHz, $CDCl_3$) δ 129.34, 128.58, 127.84, 38.69, 30.90, 29.86, 26.31, 26.23, 26.15. ^{19}F NMR (283 MHz, $CDCl_3$) δ –63.31. IR (diamond-ATR) ν: 2931, 2857, 1169, 1104, 700.

Fig. S50 ^1H NMR spectrum of cyclohexane adduct **S36** in $CDCl_3$.

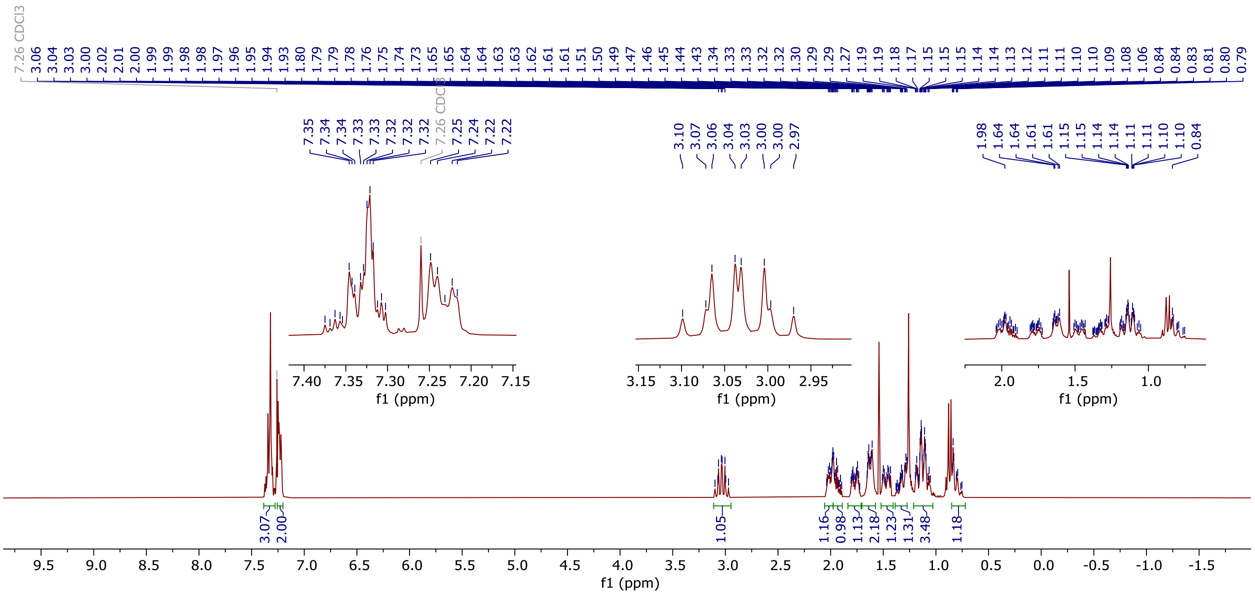

Fig. S51 ^{13}C NMR spectrum of cyclohexane adduct **S36** in $CDCl_3$.

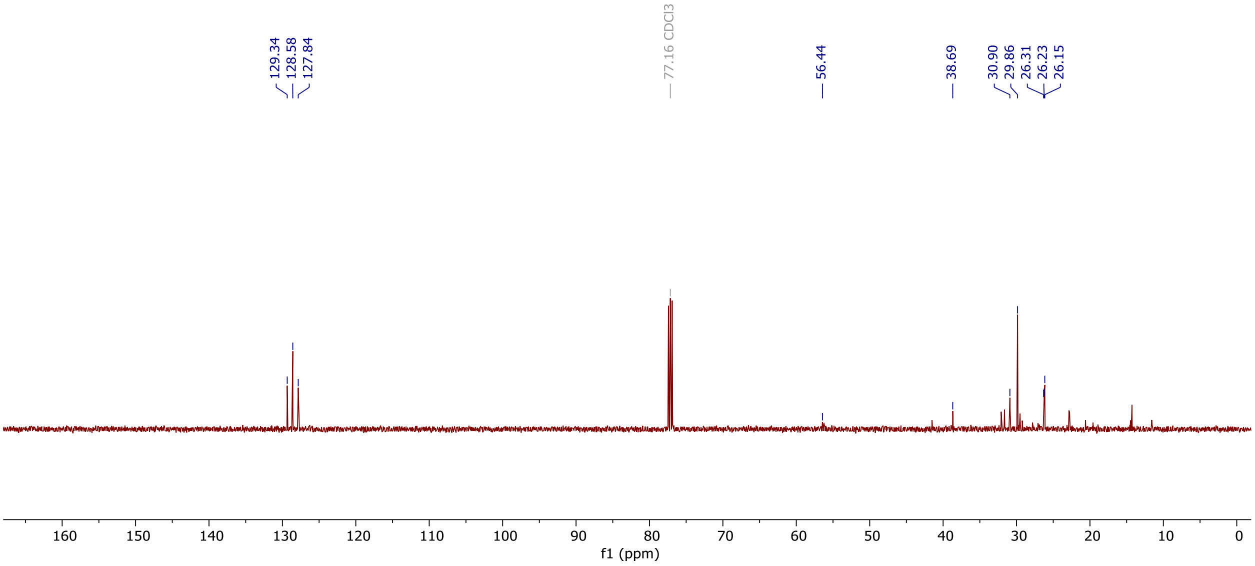

Fig. S52 ^{19}F NMR spectrum of **S36** in $CDCl_3$.

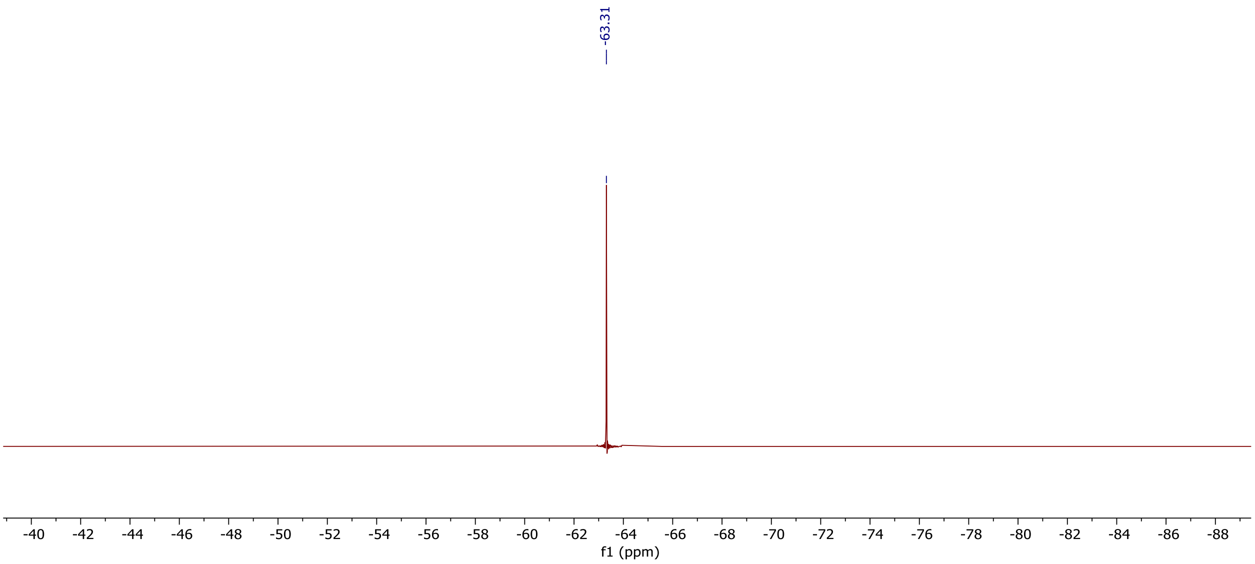

Fig. S53 Crude ^{19}F NMR spectrum under thermal insertion conditions.

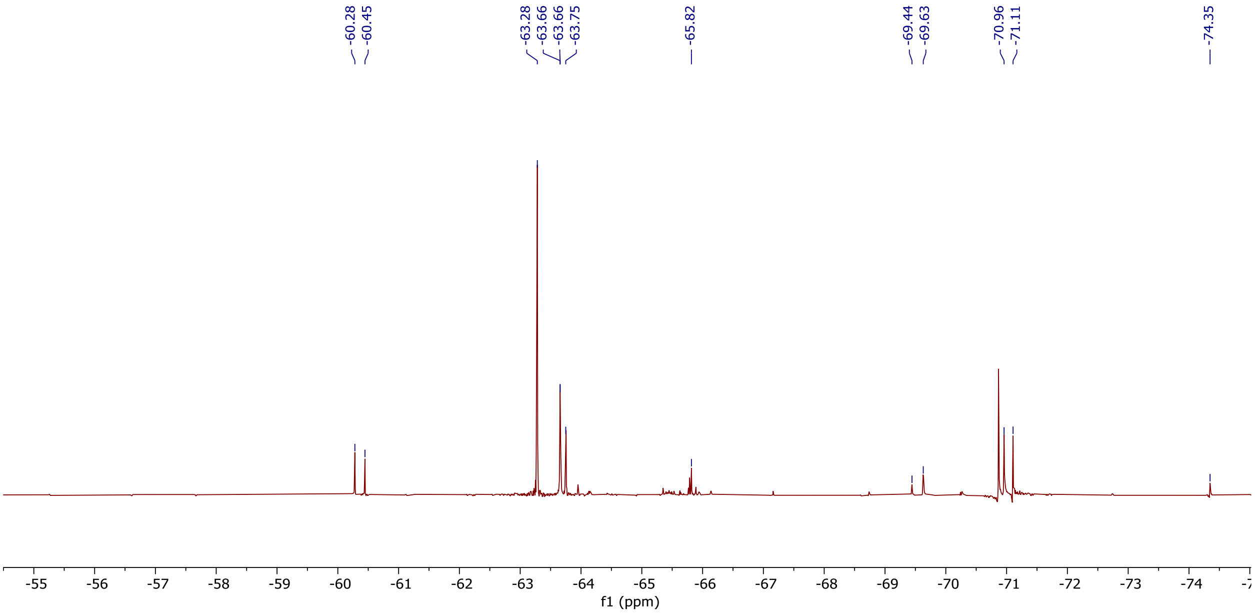

Fig. S54 Crude ^{19}F NMR spectrum under photochemical insertion conditions.

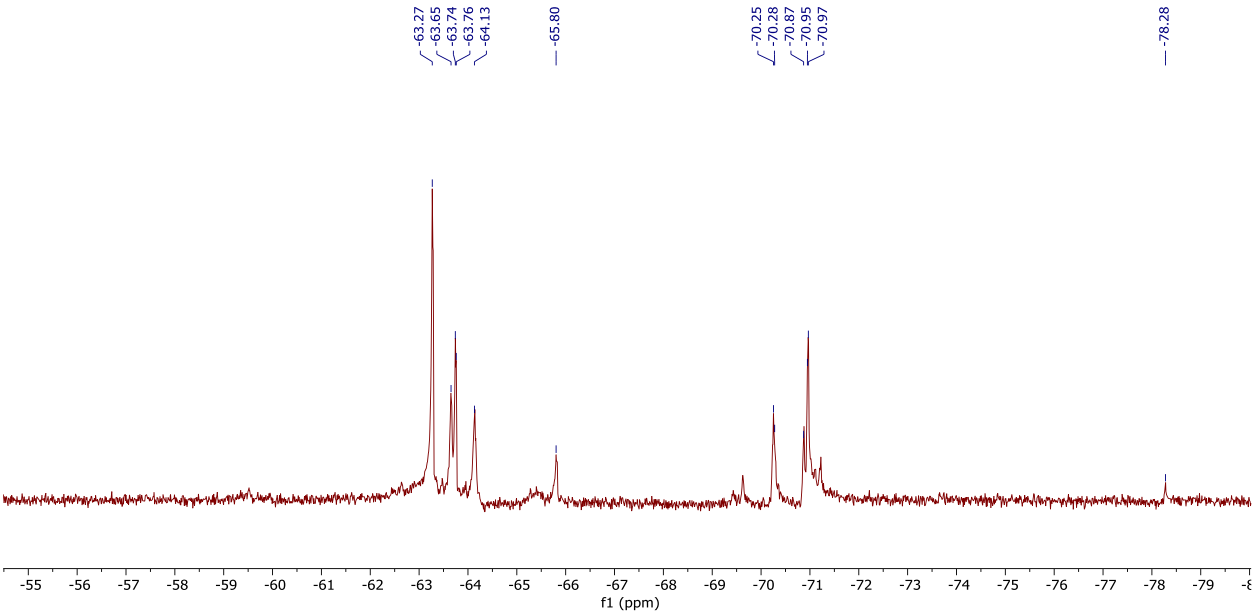

Preparation of 1-(1-cyclohexyl-2,2,2-trifluoroethyl)-3-methoxybenzene (S37).

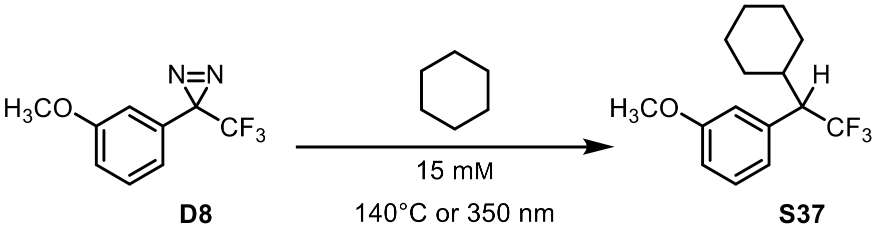

Following the above general protocols (**A** and **B**) for insertion experiments, the reaction was performed using: **A) Thermal insertion:** diazirine **D8** (64.3 mg, 0.3 mmol) dissolved in cyclohexane (20 mL). The desired product **S37** (14.7 mg, 0.054 mmol, 18%) was isolated following chromatography. **B) Photochemical insertion**: diazirine **D8** (45.1 mg, 0.208 mmol) dissolved in cyclohexane (14 mL). The desired product **S37** (11 mg, 0.04 mmol, 19%) was isolated following chromatography. ^{1}H NMR (500 MHz, $CDCl_3$) δ 7.25 (t, *J* = 7.9 Hz, 1H), 6.85 (ddd, *J* = 8.3, 2.6, 1.0 Hz, 1H), 6.82 (d, *J* = 7.7 Hz, 1H), 6.78 (t, *J* = 2.1 Hz, 1H), 3.81 (s, 3H), 3.00 (qd, *J* = 10.1, 8.3 Hz, 1H), 2.03 – 1.96 (m, 1H), 1.96 – 1.88 (m, 1H), 1.81 – 1.72 (m, 1H), 1.68 – 1.58 (m, 2H), 1.48 (dt, *J* = 14.0, 3.4 Hz, 1H), 1.32 – 1.24 (m, 1H), 1.20 – 1.07 (m, 3H), 0.83 (qd, *J* = 12.1, 3.3 Hz, 1H). ^{13}C NMR (126 MHz, $CDCl_3$) δ 159.68, 129.52, 121.79, 115.43, 112.76, 56.31 (q, *J* = 25.3 Hz), 55.34, 38.69, 31.61, 30.93, 26.30, 26.24, 26.15. ^{19}F NMR (283 MHz, $CDCl_3$) δ –63.28. IR (diamond-ATR) ν: 2928, 2855, 1603, 1586, 1251, 1156, 1132, 712.

Fig. S55 ^{1}H NMR spectrum of cyclohexane adduct **S37** in $CDCl_3$.

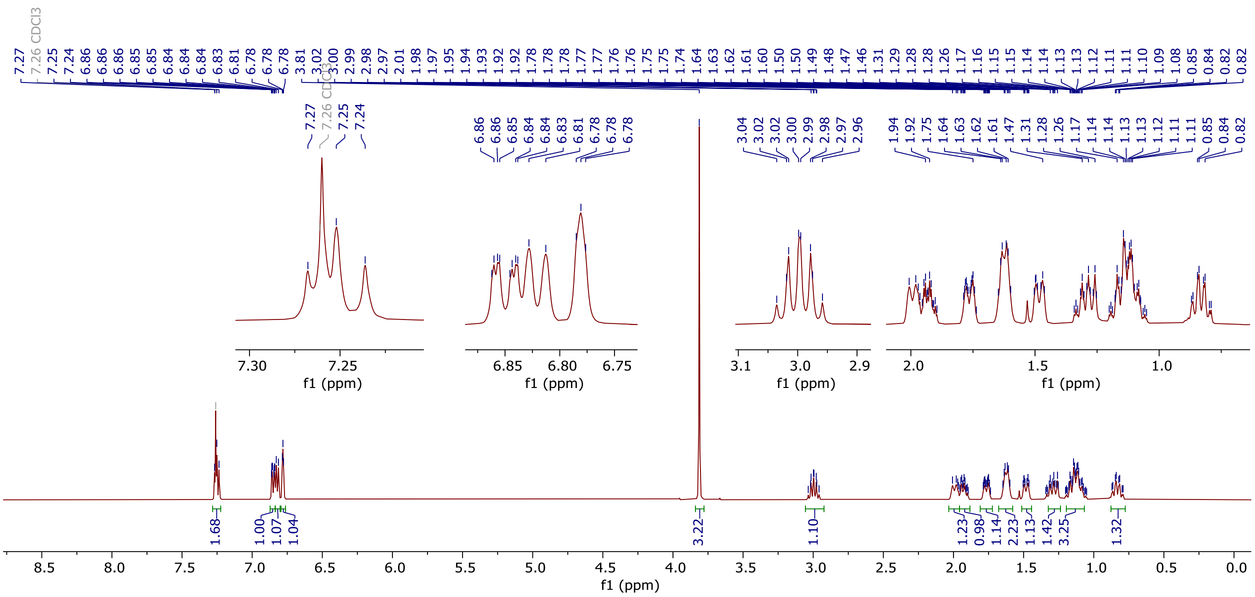

Fig. S56 ^{13}C NMR spectrum of cyclohexane adduct **S37** in $CDCl_3$.

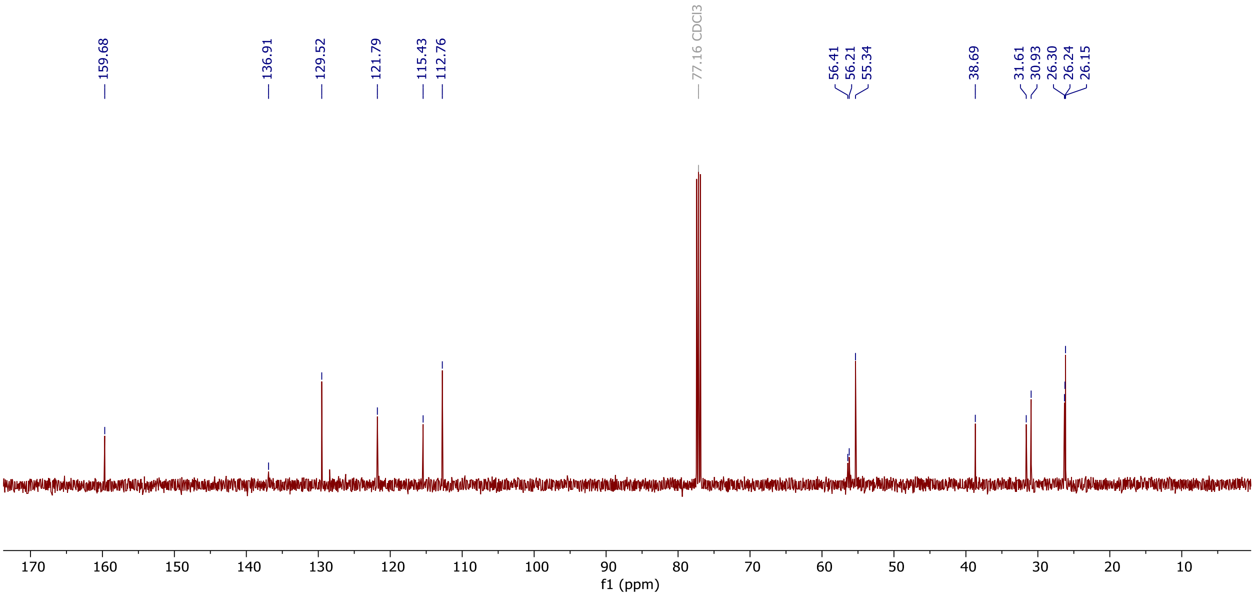

Fig. S57 ^{19}F NMR spectrum of **S37** in $CDCl_3$.

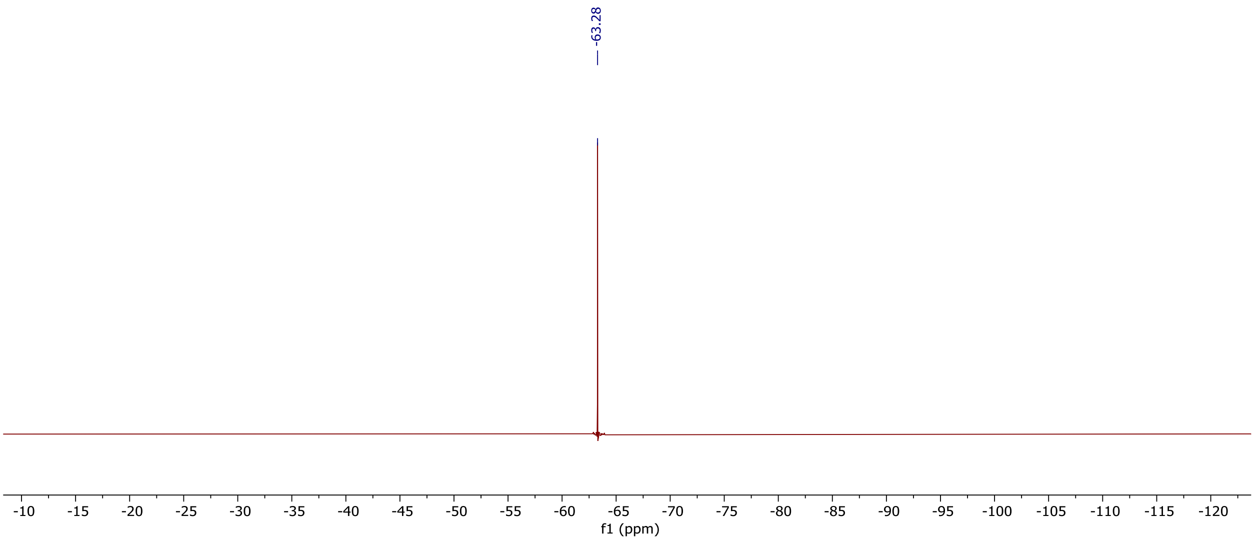

Fig. S58 Crude ^{19}F NMR spectrum under thermal insertion conditions.

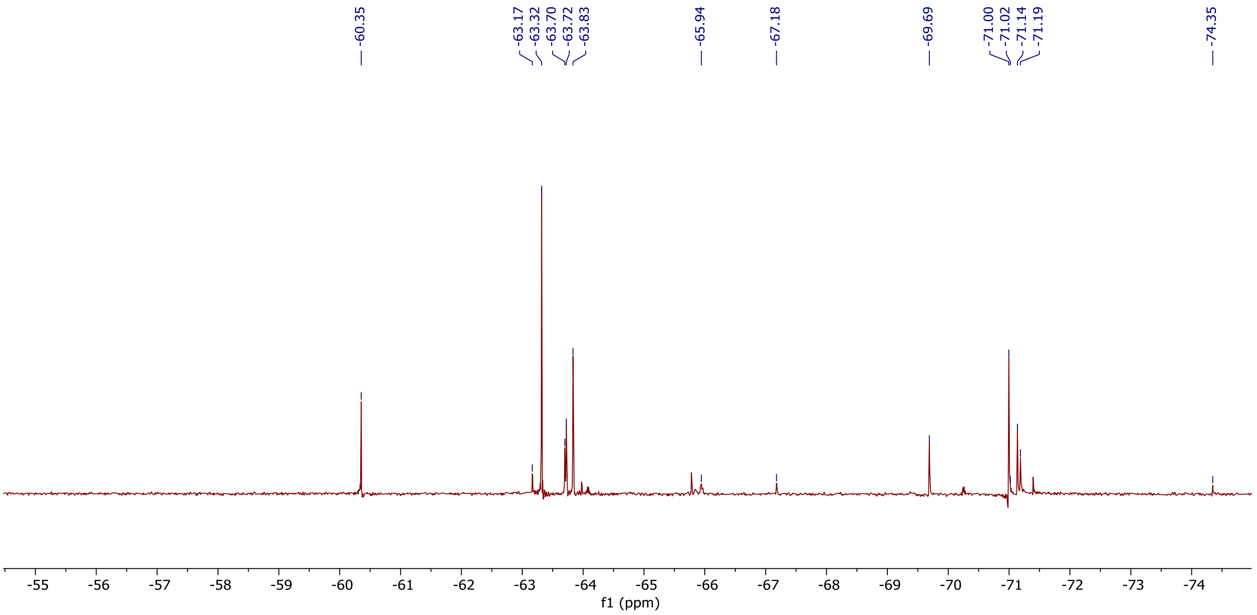

Fig. S59 Crude ^{19}F NMR spectrum under photochemical insertion conditions.

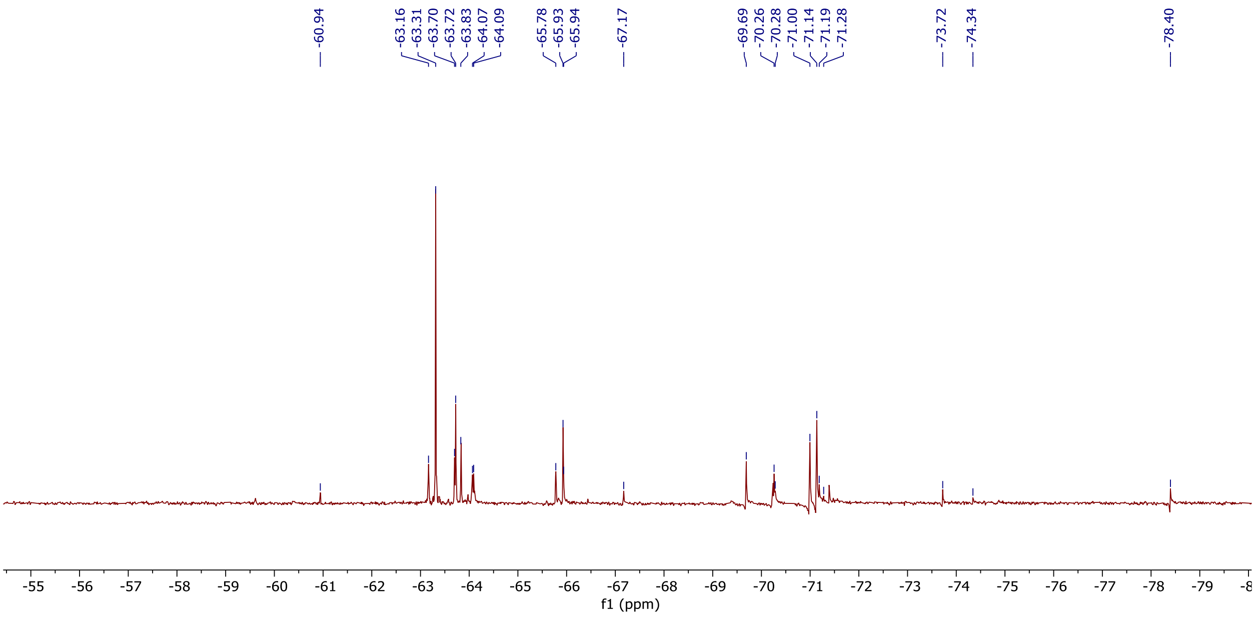

Preparation of 1-(1-cyclohexyl-2,2,2-trifluoroethyl)-4-phenoxybenzene (S38).

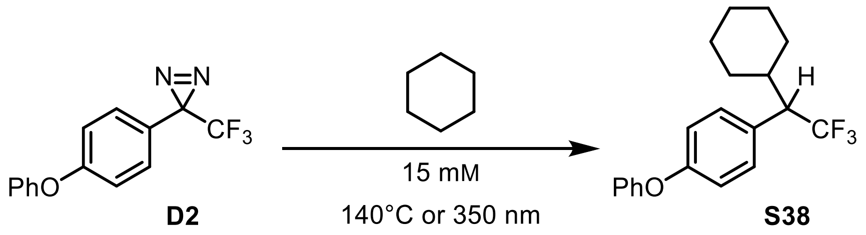

Following the above general protocols (**A** and **B**) for insertion experiments, the reaction was performed using: **A) Thermal insertion:** diazirine **D2** (51 mg, 0.183 mmol) dissolved in cyclohexane (12 mL). The desired product **S38** (45.9 mg, 0.137 mmol, 75%) was isolated following chromatography. **B) Photochemical insertion**: diazirine **D2** (22.9 mg, 0.082 mmol) dissolved in cyclohexane (5.5 mL). The desired product **S38** (21.2 mg, 0.063 mmol, 77%) was isolated following chromatography. ^{1}H NMR (500 MHz, $CDCl_3$) δ 7.40 – 7.31 (m, 2H), 7.18 (d, *J* = 8.5 Hz, 2H), 7.12 (t, *J* = 7.4 Hz, 1H), 7.03 (d, *J* = 7.6 Hz, 2H), 6.96 (d, *J* = 8.7 Hz, 2H), 3.02 (qd, *J* = 9.9, 8.5 Hz, 1H), 1.98 (d, *J* = 13.9 Hz, 1H), 1.95 – 1.88 (m, 1H), 1.83 – 1.72 (m, 1H), 1.70 – 1.59 (m, 2H), 1.50 (s, 1H), 1.37 – 1.28 (m, 1H), 1.20 – 1.04 (m, 3H), 0.83 – 0.76 (m, 1H). ^{13}C NMR (126 MHz, $CDCl_3$) δ 157.11, 156.94, 130.62, 129.94, 123.68, 119.39, 118.53, 38.70, 31.63, 30.86, 26.31, 26.24, 26.15. ^{19}F NMR (283 MHz, $CDCl_3$) δ –63.60. IR (diamond-ATR) ν:

2925, 2854, 1508, 1489, 1238, 1154, 1099. GC–MS *m/z* (% relative intensity) [ion]: 334 (90) $[M]^+$, 315 (2) $[C_{20}H_{21}F_2O]^+$, 252 (76) $[C_{14}H_{11}F_3O]^+$, 251 (72) $[C_{14}H_{10}F_3O]^+$, 183 (32) $[C_{13}H_{11}O]^+$, 159 (16) $[C_8H_6F_3]^+$, 83 (100) $[C_6H_{11}]^+$, 77 (46) $[C_6H_5]^+$, 55 (96) $[C_4H_7]^+$. [M = $C_{20}H_{21}F_3O$ (334.15)].

Fig. S60 ^{1}H NMR spectrum of cyclohexane adduct **S38** in $CDCl_3$.

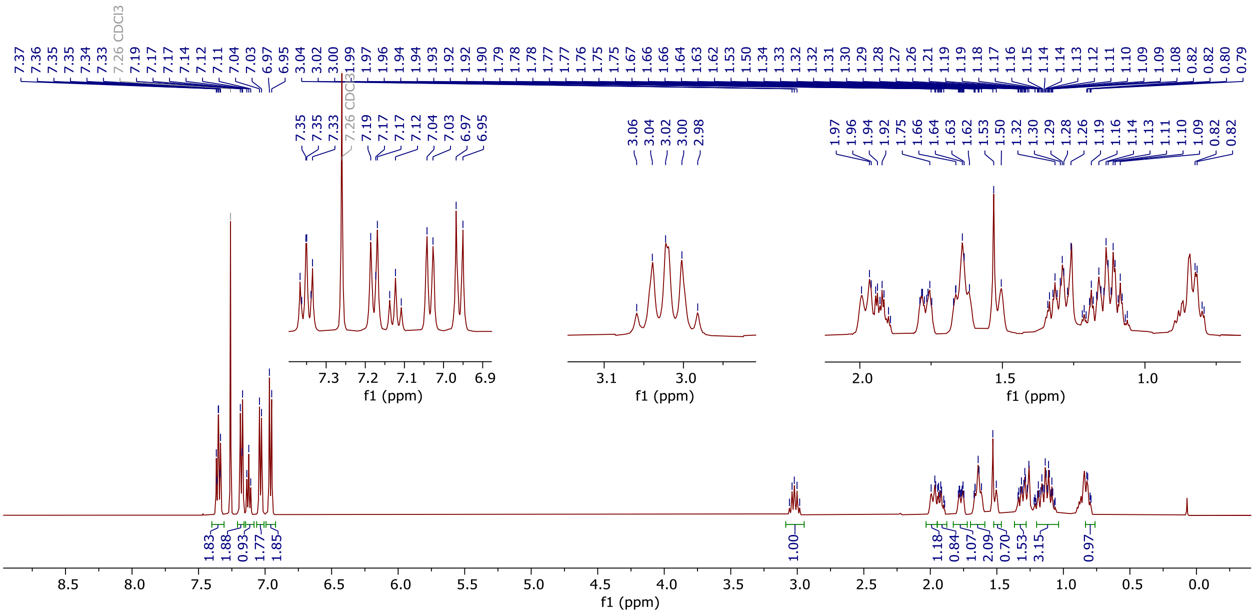

Fig. S61 ^{13}C NMR spectrum of cyclohexane adduct **S38** in $CDCl_3$.

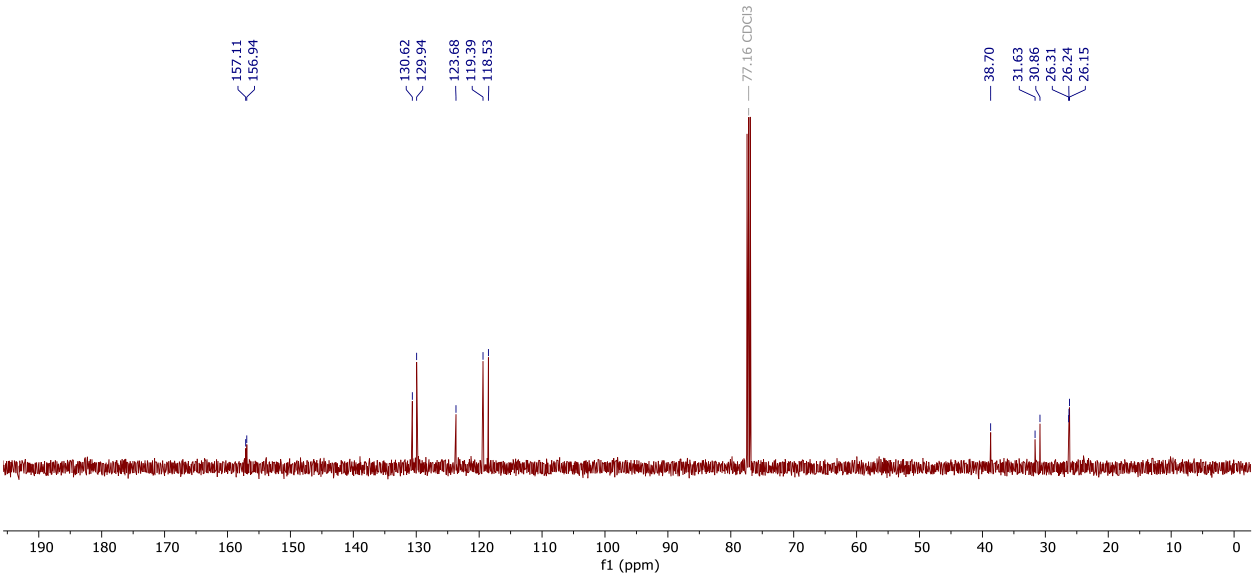

Fig. S62 ^{19}F NMR spectrum of **S38** in $CDCl_3$.

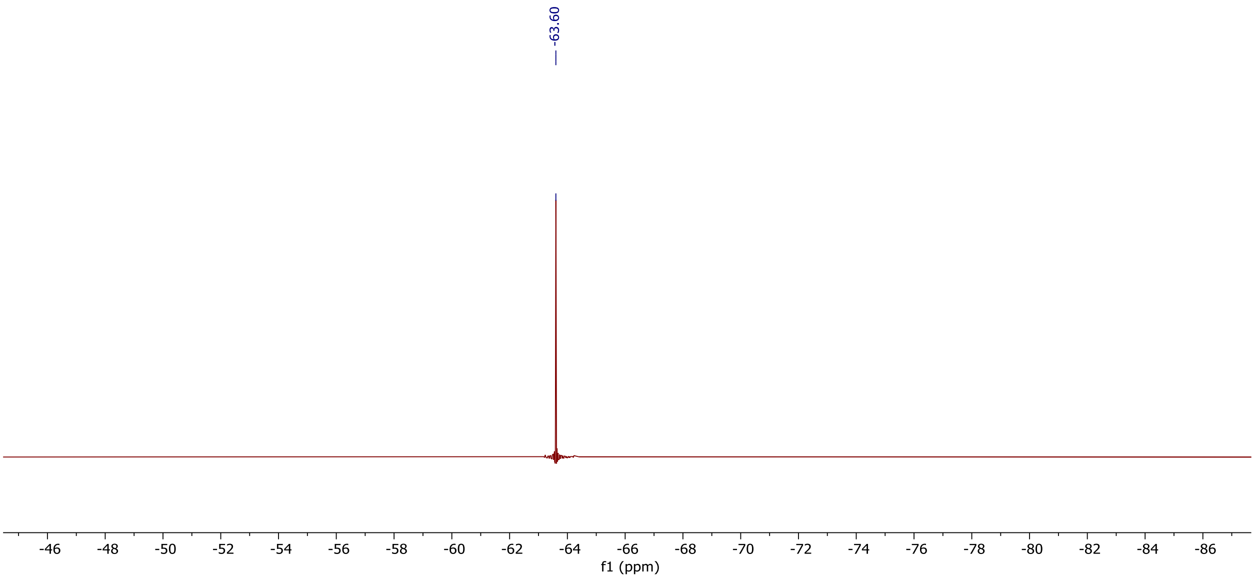

Fig. S63 Crude ^{19}F NMR spectrum under thermal insertion conditions.

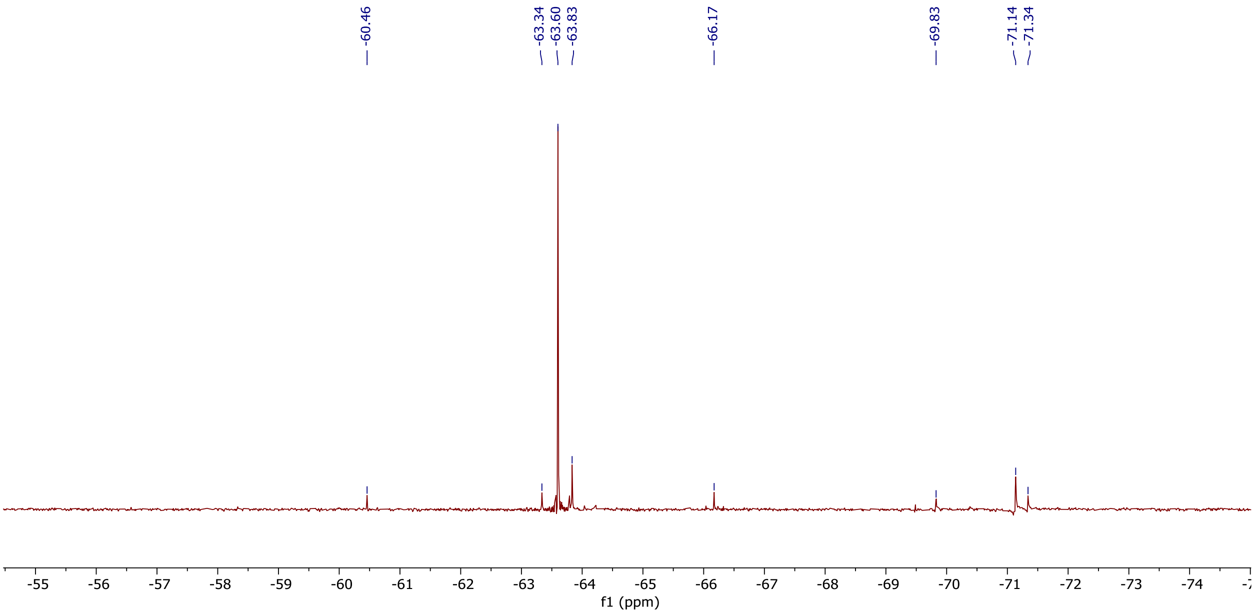

Fig. S64 Crude ^{19}F NMR spectrum under photochemical insertion conditions.

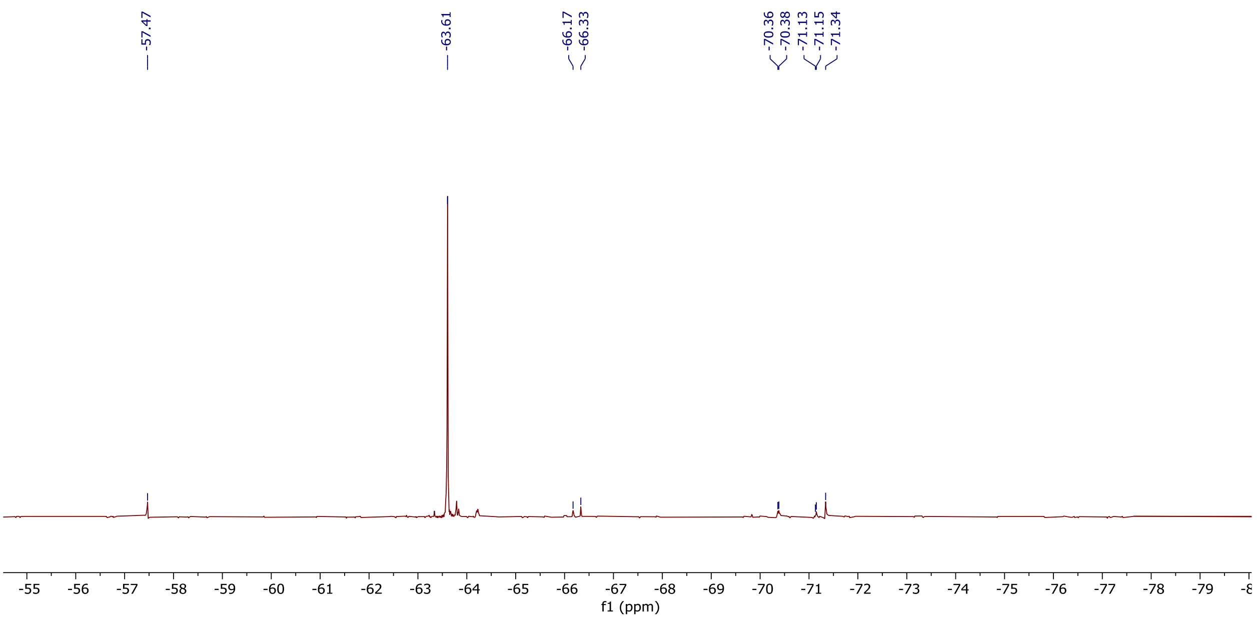

Preparation of 1-(1-cyclohexyl-2,2,2-trifluoroethyl)-4-methoxybenzene (S39).

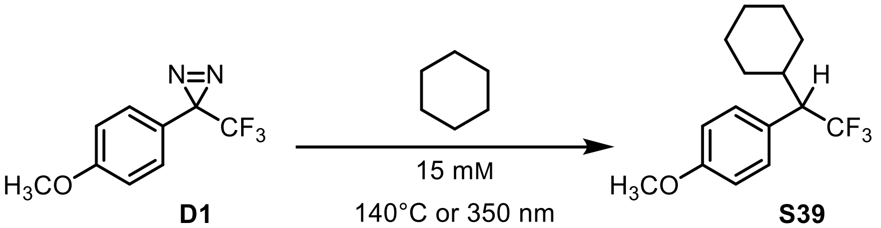

Following the above general protocols (**A** and **B**) for insertion experiments, the reaction was performed using: **A) Thermal insertion:** diazirine **D1** (108 mg, 0.5 mmol) dissolved in cyclohexane (35 mL). The desired product **S39** (124 mg, 0.455 mmol, 91%) was isolated following chromatography. **B) Photochemical insertion**: diazirine **D1** (56.1 mg, 0.259 mmol) dissolved in cyclohexane (17 mL). The desired product **S39** (66.4 mg, 0.244 mmol, 94%) was isolated following chromatography. Spectroscopic data are in accordance with the literature.[8] ^{1}H NMR (500 MHz, $CDCl_3$) δ 7.15 (d, *J* = 8.6 Hz, 2H), 6.87 (d, *J* = 8.7 Hz, 2H), 3.81 (s, 3H), 3.01 (qd, *J* = 10.0, 8.4 Hz, 1H), 1.96 (d, *J* = 14.0 Hz, 1H), 1.90 (ddt, *J* = 11.4, 6.8, 3.3 Hz, 1H), 1.81 – 1.72 (m, 1H), 1.67 – 1.57 (m, 2H), 1.50 (d, *J* = 13.6 Hz, 1H), 1.36 – 1.23 (m, 1H), 1.22 – 1.02 (m, 3H), 0.87 – 0.75 (m, 1H). ^{13}C NMR (126 MHz, $CDCl_3$) δ 159.20, 130.38, 113.96, 55.35, 38.68, 31.66, 30.82, 26.33, 26.26, 26.17. ^{19}F NMR (283 MHz, $CDCl_3$) δ –63.74. IR (diamond-ATR) ν: 2927, 2855, 1515, 1247, 1153, 1134, 1099, 831, 820.

Fig. S65 ^{1}H NMR spectrum of cyclohexane adduct **S39** in $CDCl_3$.

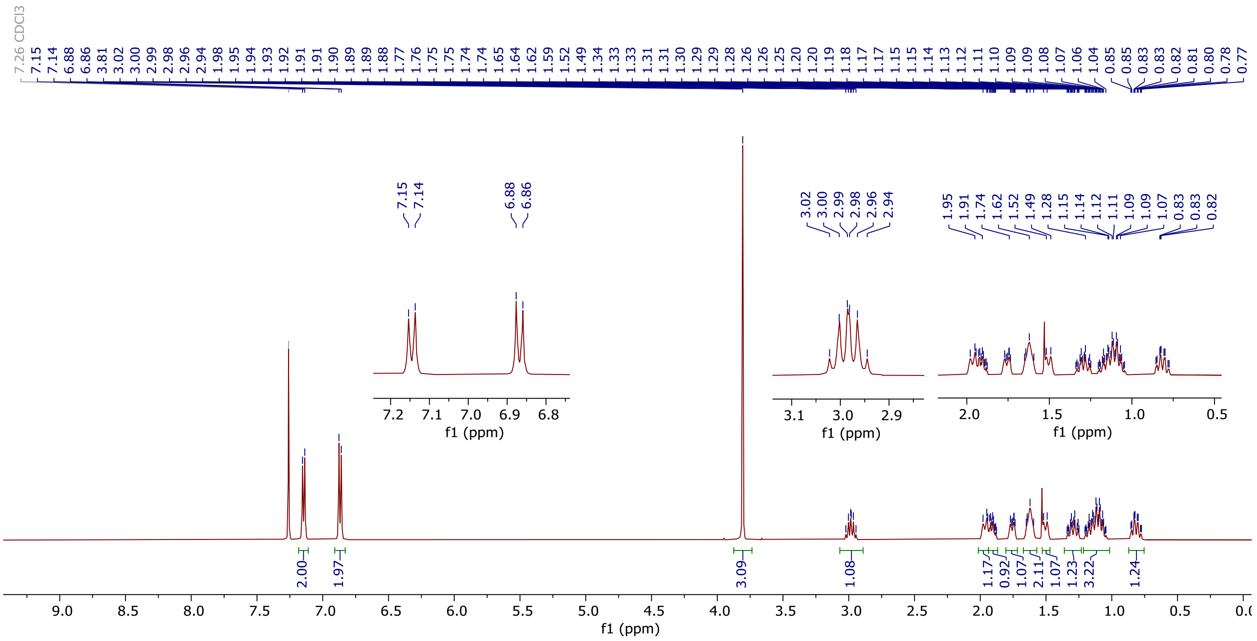

Fig. 66 ^{13}C NMR spectrum of cyclohexane adduct **S39** in $CDCl_3$.

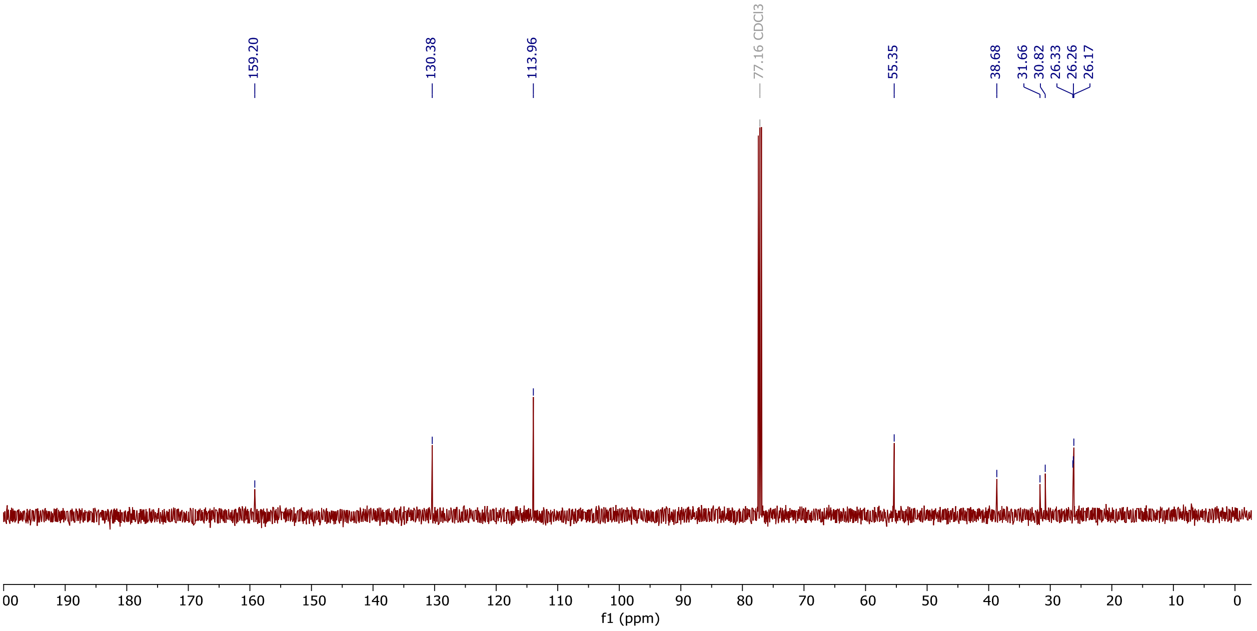

Fig. S67 ^{19}F NMR spectrum of **S39** in $CDCl_3$.

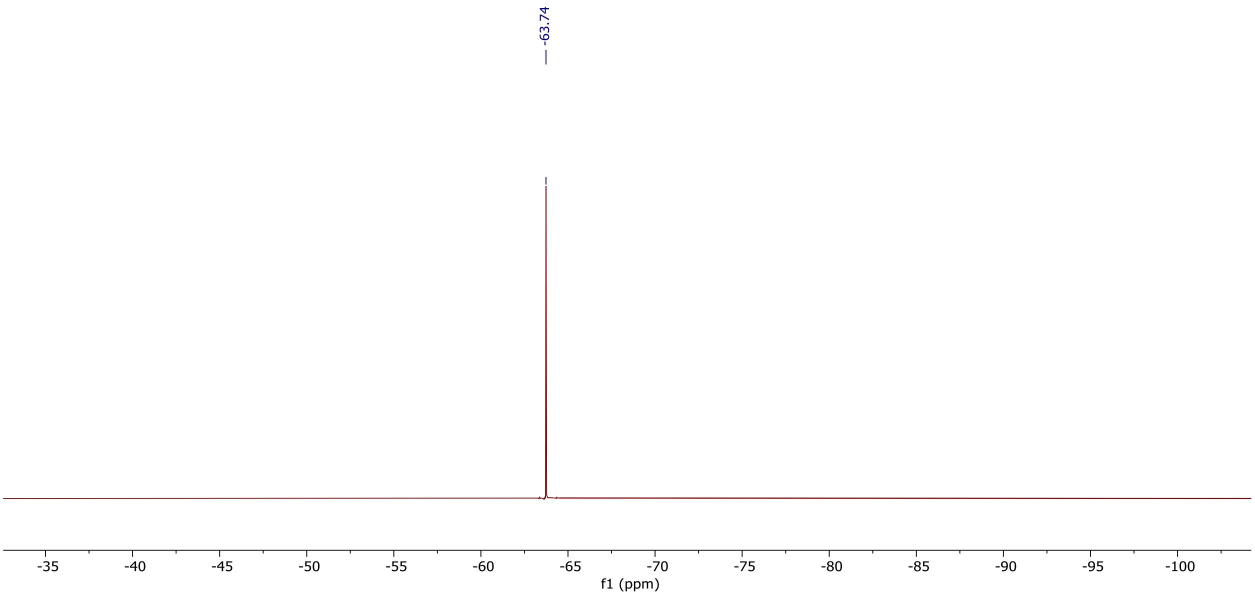

Fig. S68 Crude ^{19}F NMR spectrum under thermal insertion conditions.

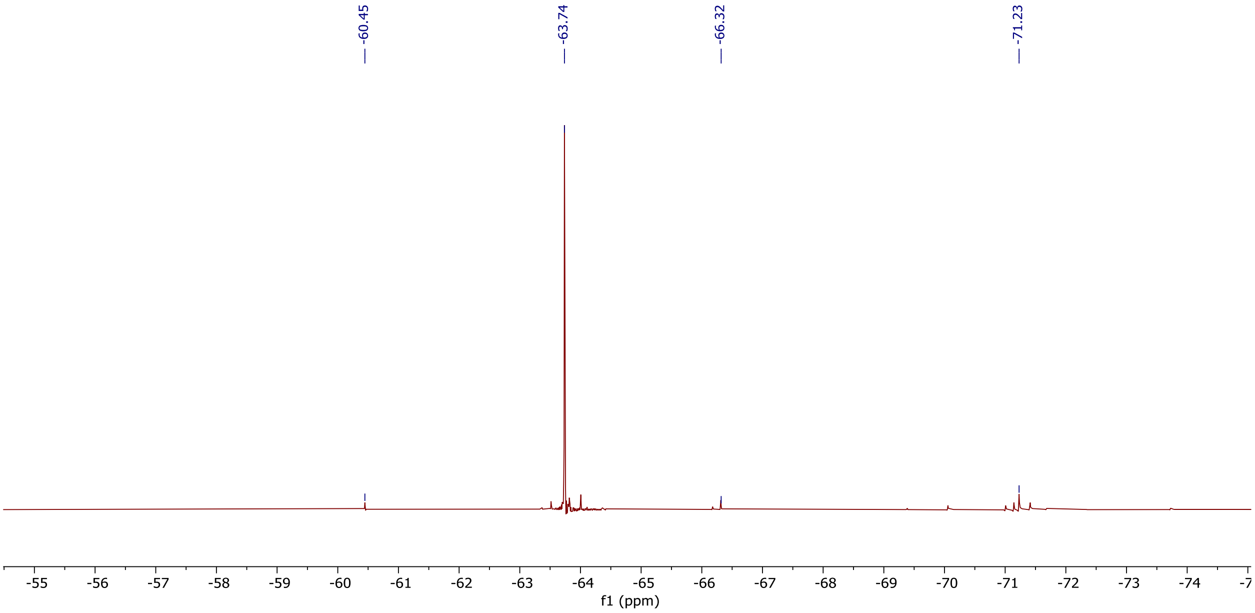

Fig. S69 Crude ^{19}F NMR spectrum under photochemical insertion conditions.

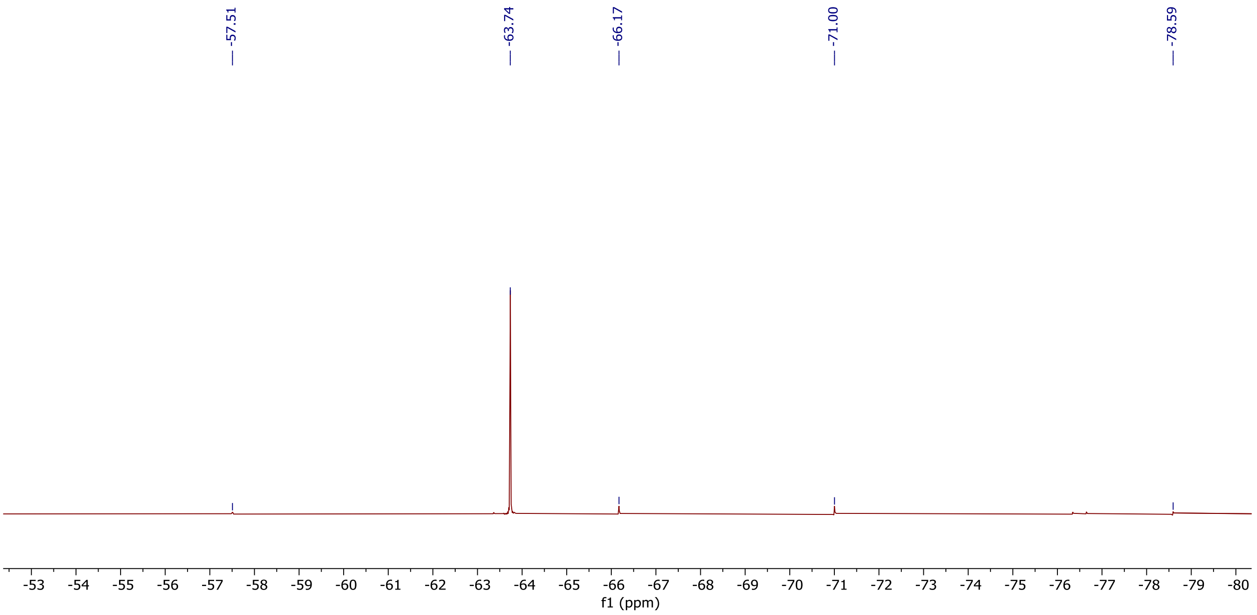

Fig. S70 Comparison of crude ^{19}F NMR spectra under thermal C–H insertion experiments.

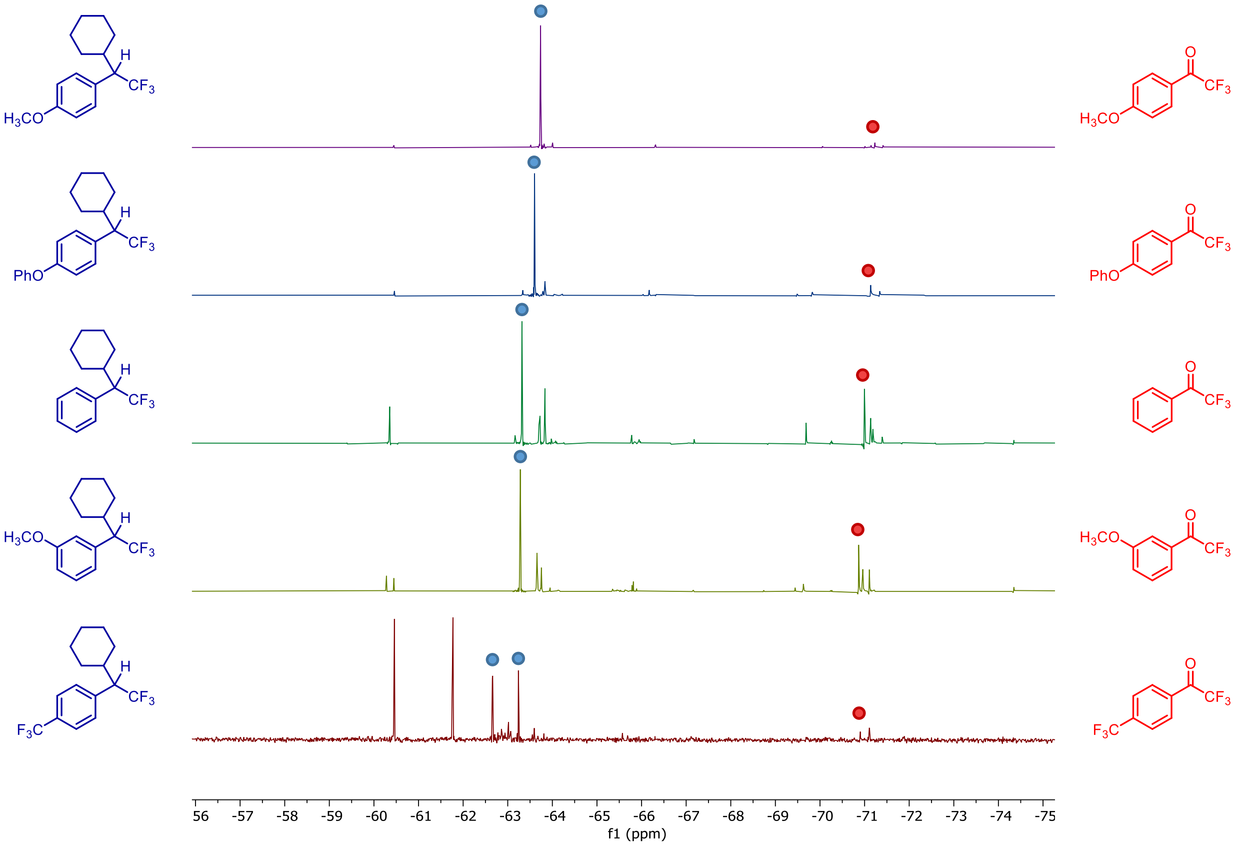

Fig. S71 Comparison of crude ^{19}F NMR spectra under photochemical C–H insertion experiments.

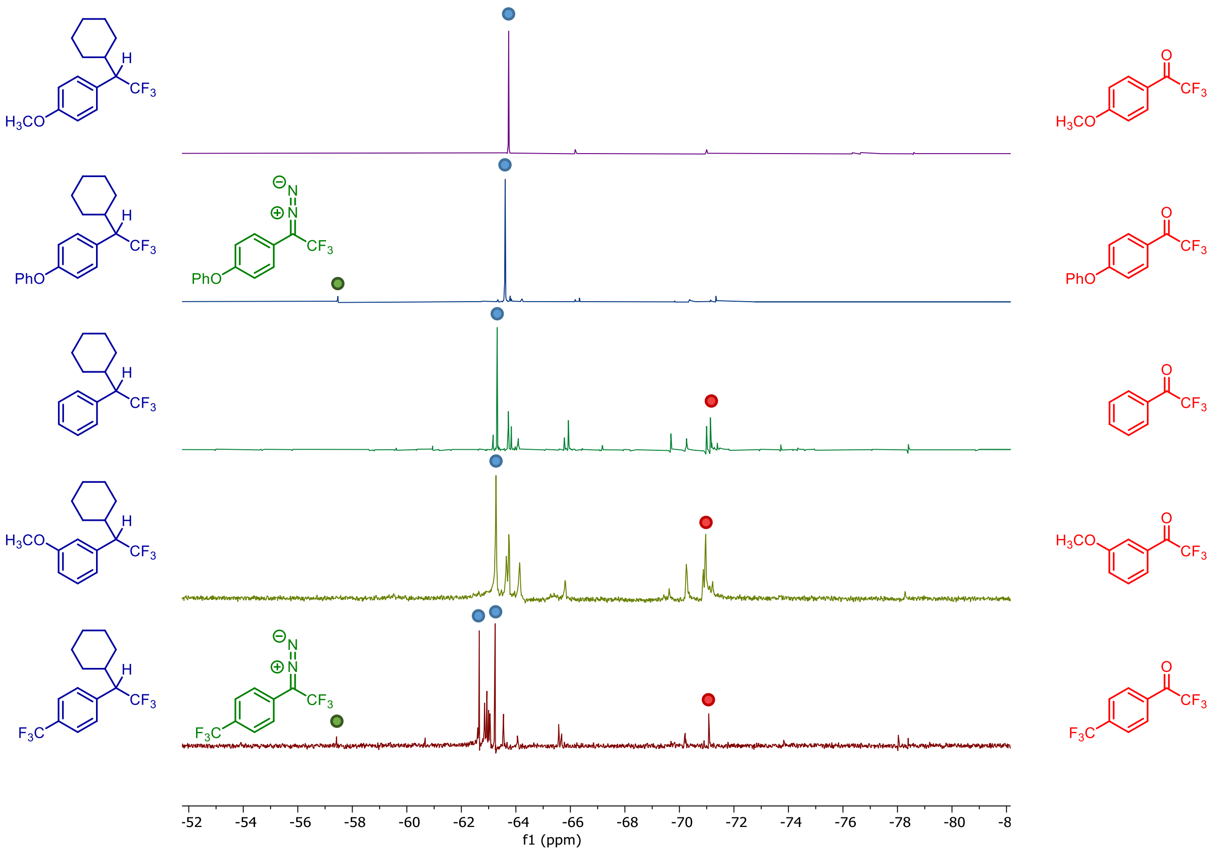

Insertion reactions in a wet environment

Insertion to cyclohexane:water (9:1)

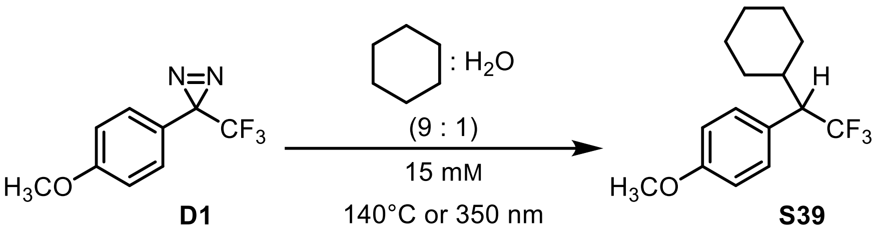

Following the above general protocols (**C** and **D**) for insertion experiments, the reaction was performed using: **C) Thermal insertion:** diazirine **D1** (8.5 mg, 0.039 mmol) dissolved in cyclohexane:water (9:1, 2.6 mL). The desired product **S39** (9.8 mg, 0.0359 mmol, 92%) was isolated following chromatography. **D) Photochemical insertion**: diazirine **D1** (10 mg, 0.046 mmol) dissolved in cyclohexane:water (9:1, 3 mL). The desired product **S39** (11.2 mg, 0.041 mmol, 90%) was isolated following chromatography. Spectroscopic data are consistent with the ones reported above for compound **S39**.

Fig. S72 Crude ^{1}H NMR spectra of insertion reaction in cyclohexane:water 9:1 (top: thermal; bottom: photochemical).

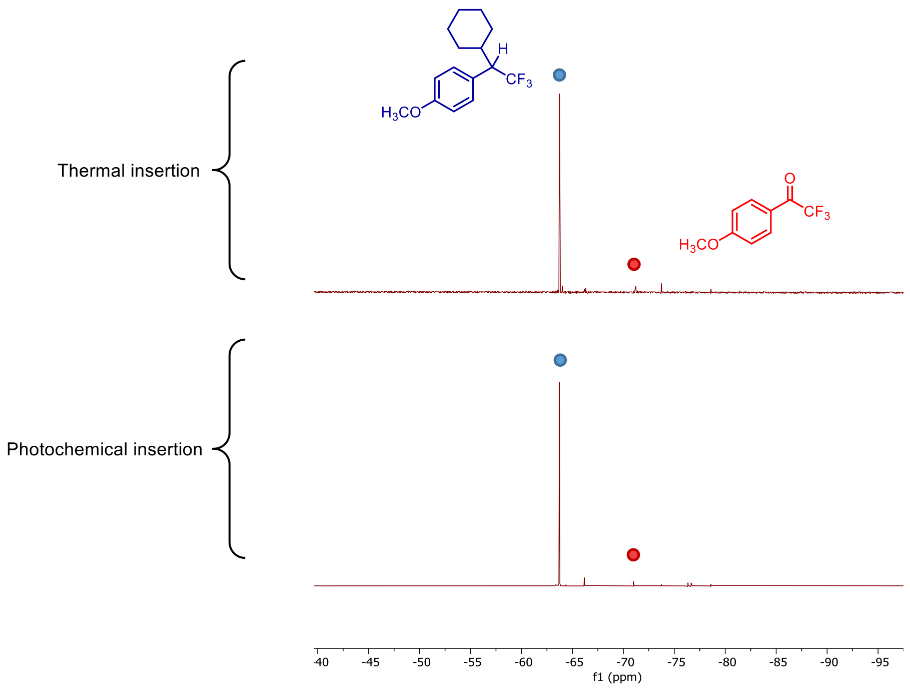

Insertion to *tert*-Butanol

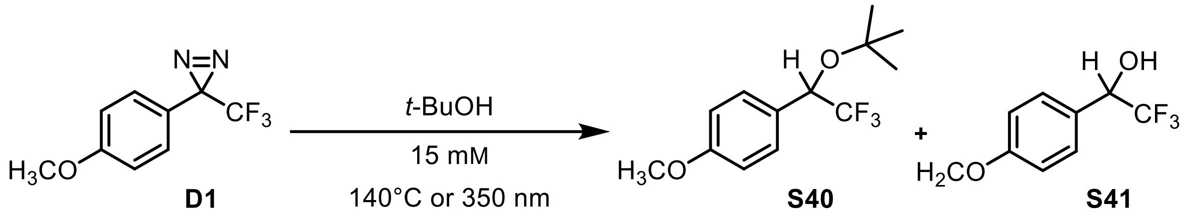

Following the above general protocols (**C** and **D**) for insertion experiments, the reaction was performed using: **C) Thermal insertion:** diazirine **D1** (10.7 mg, 0.049 mmol) dissolved in *tert*-butanol (3.2 mL). Compound **S40** (8.1 mg, 0.031 mmol, 63%) and compound **S41** (2.3 mg, 0.017 mmol, 23%) were isolated following chromatography using petroleum ether: diethyl ether (9:1, R_f = 0.72 (**S40**) and R_f = 0.25 (**S41**)). **D) Photochemical insertion**: diazirine **D1** (16.2 mg, 0.075 mmol) dissolved in *tert*-butanol (5 mL). Compound **S40** (12.5 mg, 0.048 mmol, 64%) and compound **S41** (4.2 mg, 0.020 mmol, 27%) were isolated following chromatography.

1-(1-(*tert*-Butoxy)-2,2,2-trifluoroethyl)-4-methoxybenzene (S40): ^{1}H NMR (500 MHz, $CDCl_3$) δ 7.38 (d, *J* = 8.7 Hz, 2H), 6.89 (d, *J* = 8.8 Hz, 2H), 4.73 (q, *J* = 7.0 Hz, 1H), 3.81 (s, 3H), 1.18 (s, 9H). ^{13}C NMR (126 MHz, $CDCl_3$) δ 160.07, 129.15, 113.81, 72.29 (q, *J* = 31.1 Hz), 55.38, 29.85, 28.29. ^{19}F NMR (283 MHz, $CDCl_3$) δ –77.22. IR (diamond-ATR) ν: 2960, 2928, 2858, 1613, 1514, 1269, 1249, 1129. GC–MS *m/z* (% relative intensity) [ion]: 262 (10) $[M]^+$, 193 (24) $[C_{12}H_{17}O_2]^+$, 189 (30) $[C_9H_8F_3O]^+$, 137 (100) $[C_8H_9O_2]^+$, 107 (5) $[C_7H_7O]^+$, 77 (10) $[C_6H_5]^+$, 57 (30) $[C_4H_9]^+$. [M = $C_{13}H_{17}F_3O_2$ (262.12)].

Fig. S73 ^{1}H NMR spectrum of **S40** in $CDCl_3$.

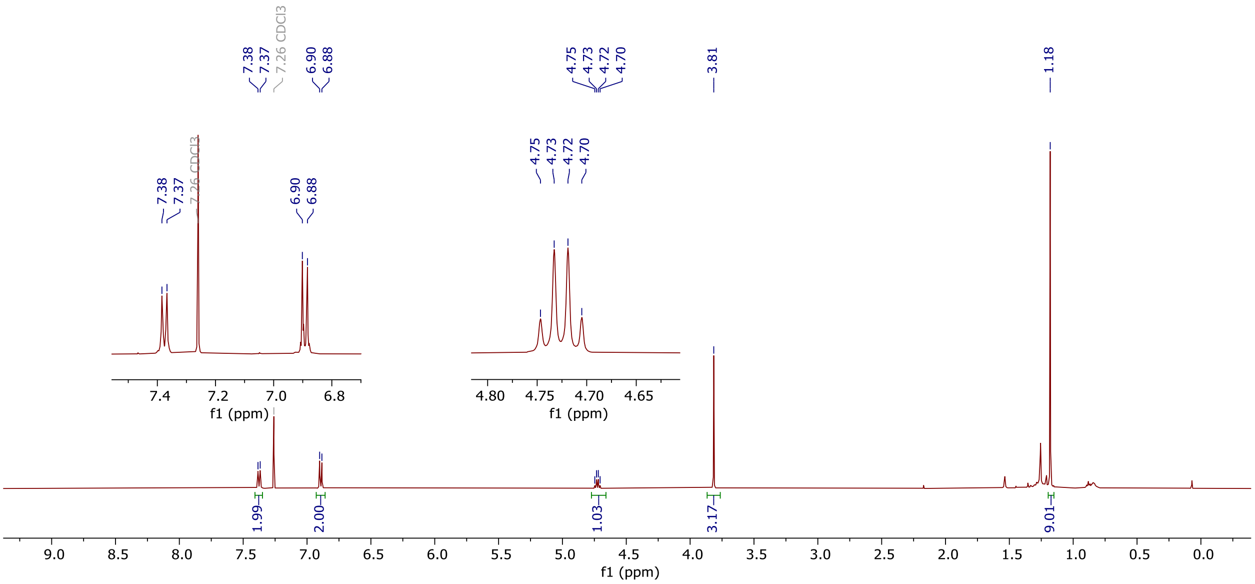

Fig. S74 ^{13}C NMR spectrum of **S40** in $CDCl_3$.

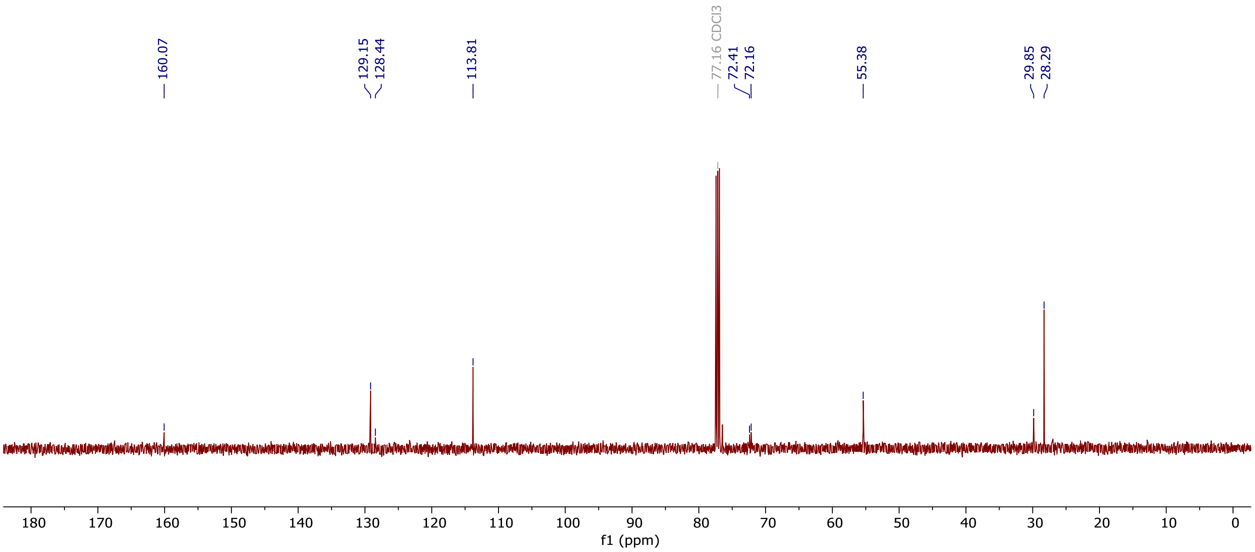

Fig. S75 ^{19}F NMR spectrum of **S40** in $CDCl_3$.

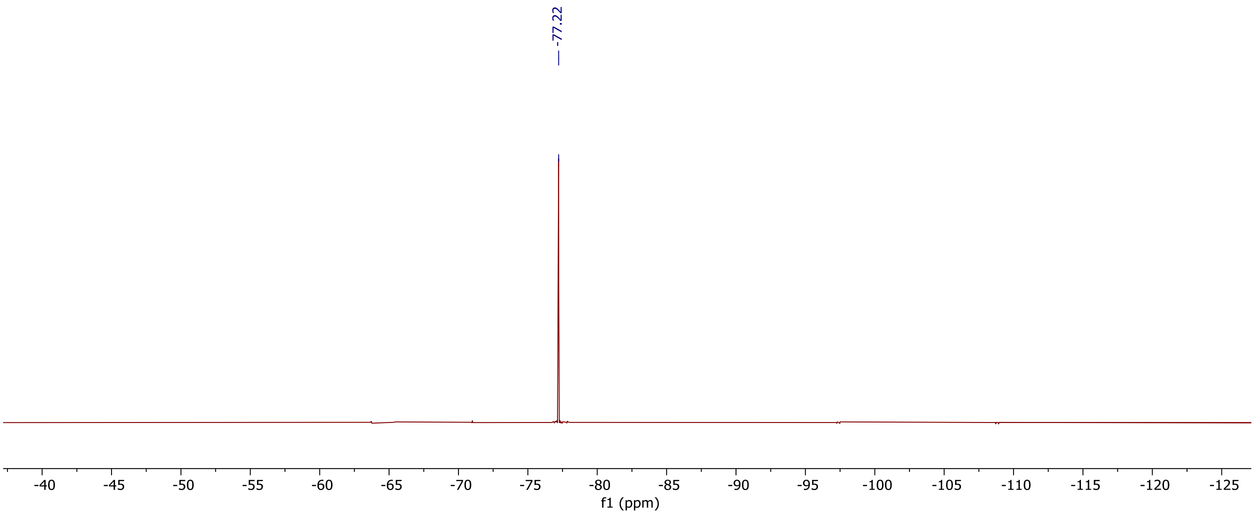

(4-(2,2,2-Trifluoro-1-hydroxyethyl)phenoxy)methylium (S41): ^{1}H NMR (500 MHz, $CDCl_3$) δ 7.48 – 7.33 (m, 2H), 6.94 (d, *J* = 8.8 Hz, 2H), 4.98 (q, *J* = 6.7 Hz, 1H), 3.83 (s, 3H), 2.08 (d, *J* = 5.0 Hz, 1H, OH). ^{19}F NMR (283 MHz, $CDCl_3$) δ – 78.59. Spectroscopic data in accordance with the literature.[8]

Fig. S76 ^{1}H NMR spectrum of **S41** in $CDCl_3$.

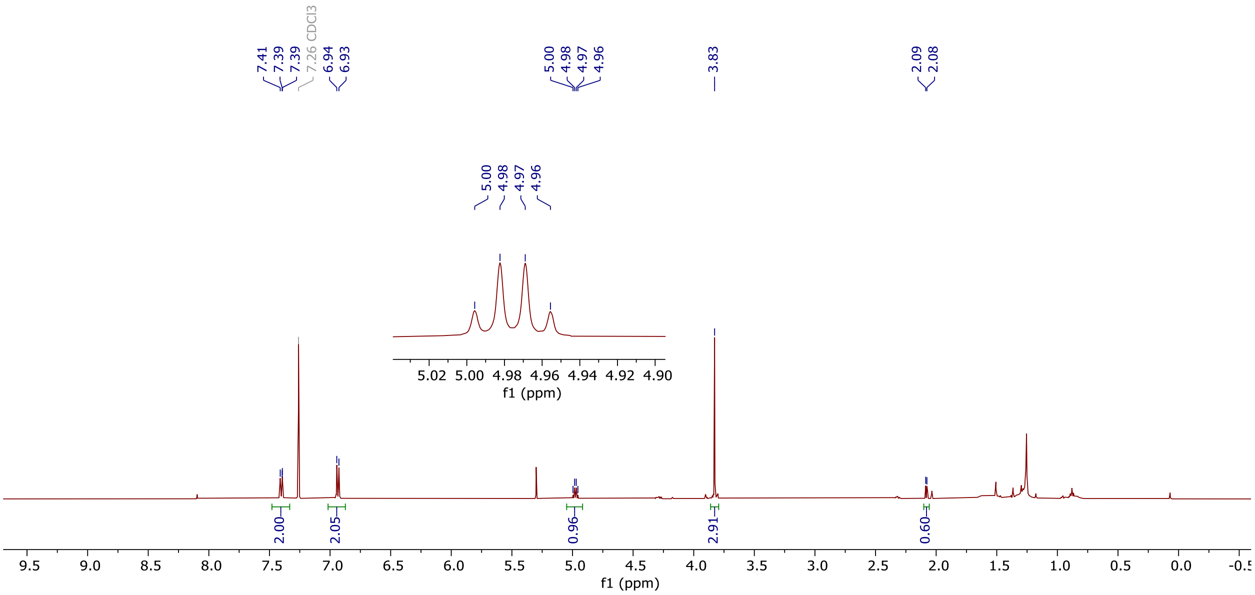

Fig. S77 ^{19}F NMR spectrum of **S41** in $CDCl_3$.

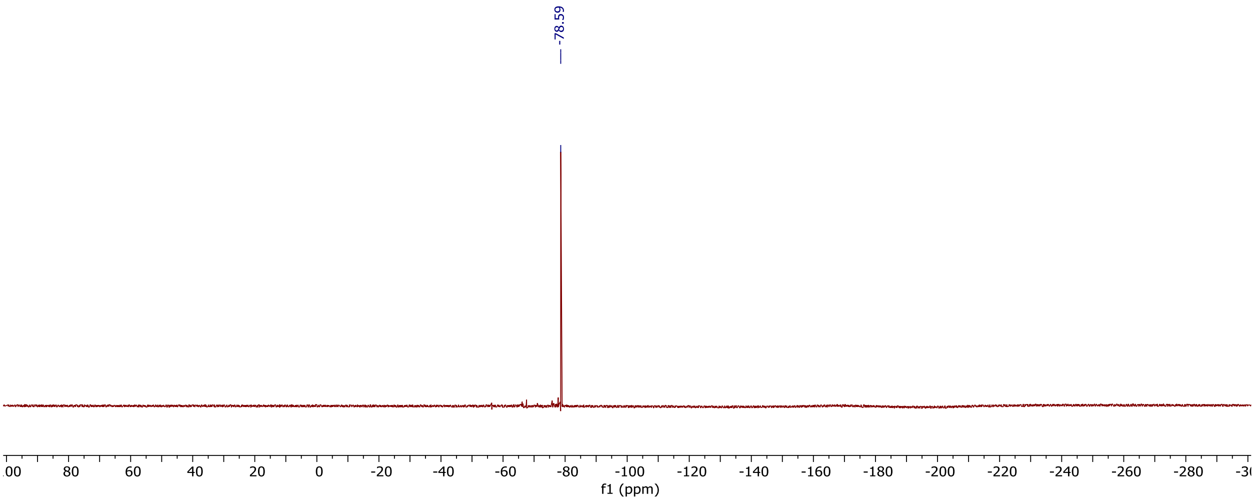

Fig. S78 Crude ^{1}H NMR spectra of insertion reaction in tert-butanol (top: thermal; bottom: photochemical).

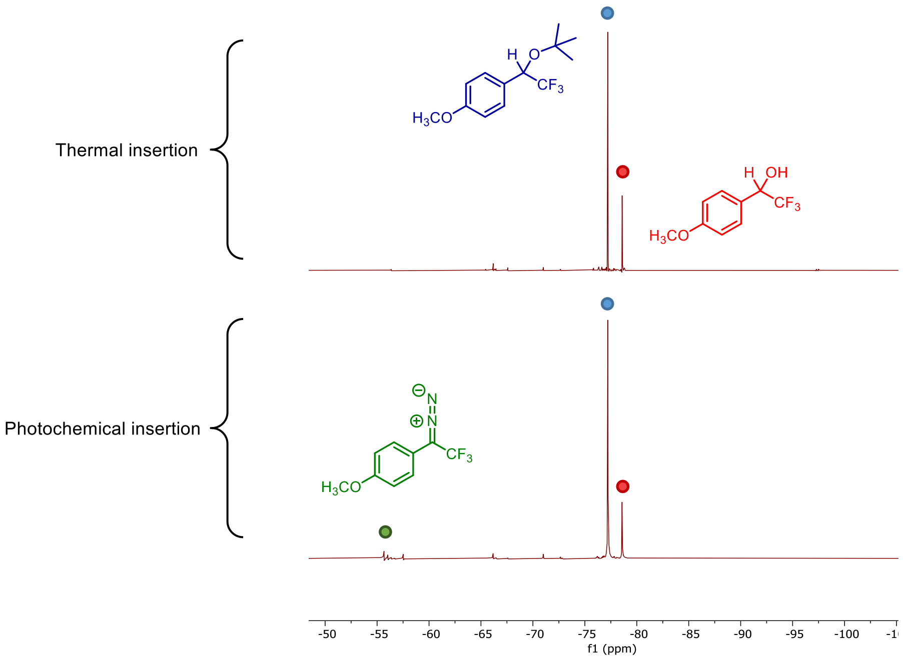

Monitoring of diazo-isomer formation during thermal activation of diazirines

Diazirines are known to undergo partial rearrangement to linear diazo species upon excitation, although this isomerization is known to be less significant for α-trifluoromethyl diazirines than for other diazirine species,[25] and is also known to be less significant under conditions of thermal activation compared with photochemical activation.[26] The weak correlation between experimentally determined activation temperature and calculated activation energy observed in Table 2 led us to question whether the α-H, -CH_3 and -Cl substrates may have followed a different reaction pathway (possibly involving the diazo-isomer) in the DSC experiment, compared to the α-CF_3 substrate. Therefore, we carried out thermal activation experiments on four representative α-substituted diazirines while monitoring the progress of the reaction by UV/Vis spectroscopy, in order to determine if the formation of the diazo-isomer during thermal activation could be detected.

Traces of diazo-isomer formation (indicated by a signal in the characteristic diazo absorption region of 450–550 nm) were observed only with the α-CH_3 sample. We observed a gradual consumption of the α-CF_3 diazirine over the course of 20 mins, whereas the other three α-substituted diazirines were consumed in less than 6 mins.

Decomposition of D14, D15, D16, and D5 under thermal activation.

A SpectraMax M5 multi-channel platereader was used for all spectral measurements. The diazirine solution in cyclohexane was prepared as described above for the cyclohexane C–H insertion experiment. For each substrate, thermal activation was performed in ten high-pressure glass tubes, under an atmosphere of argon, at 140 °C. Each tube was withdrawn from the heating bath at the appropriate time, and the UV/Vis spectrum was recorded.

Fig. S79 UV absorbance spectra of **D14** showing consumption of starting material over time.

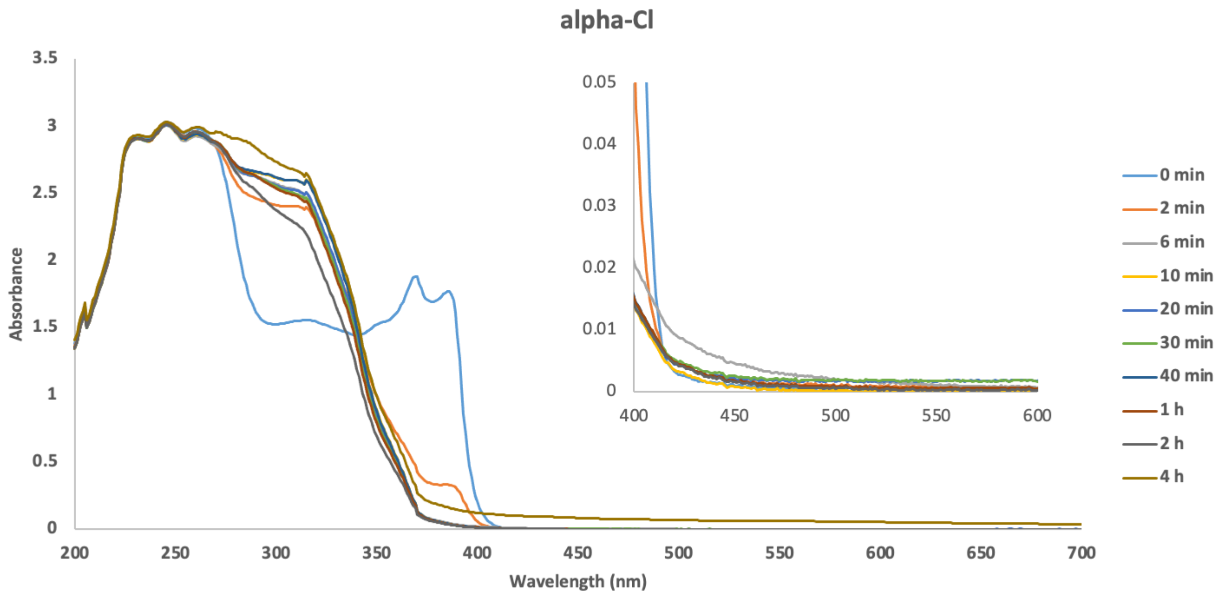

The diazirine was consumed over the first 6 mins, and examination of the spectrum between 400–600 nm (inset) indicated the absence of the diazo-isomer.

Fig. S80 UV absorbance spectra of **D15** showing consumption of starting material over time.

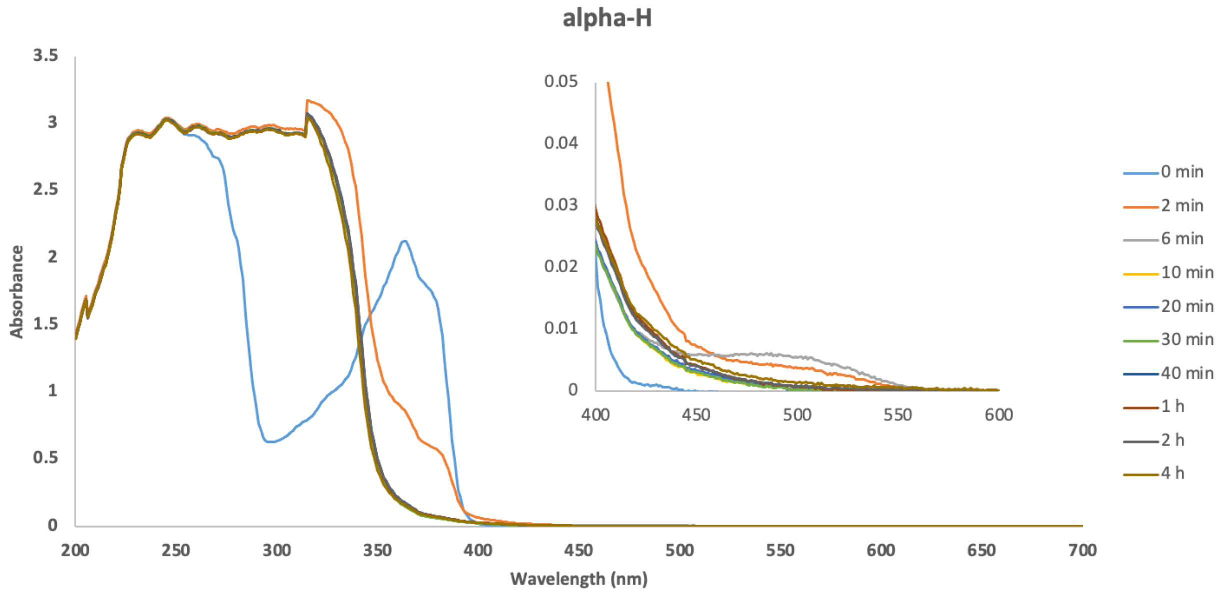

The diazirine was consumed over the first 6 mins, and examination of the spectrum between 400–600 nm (inset) indicated only trace amounts of the diazo-isomer.

Fig. S81 UV absorbance spectra of **D16** showing consumption of starting material over time.

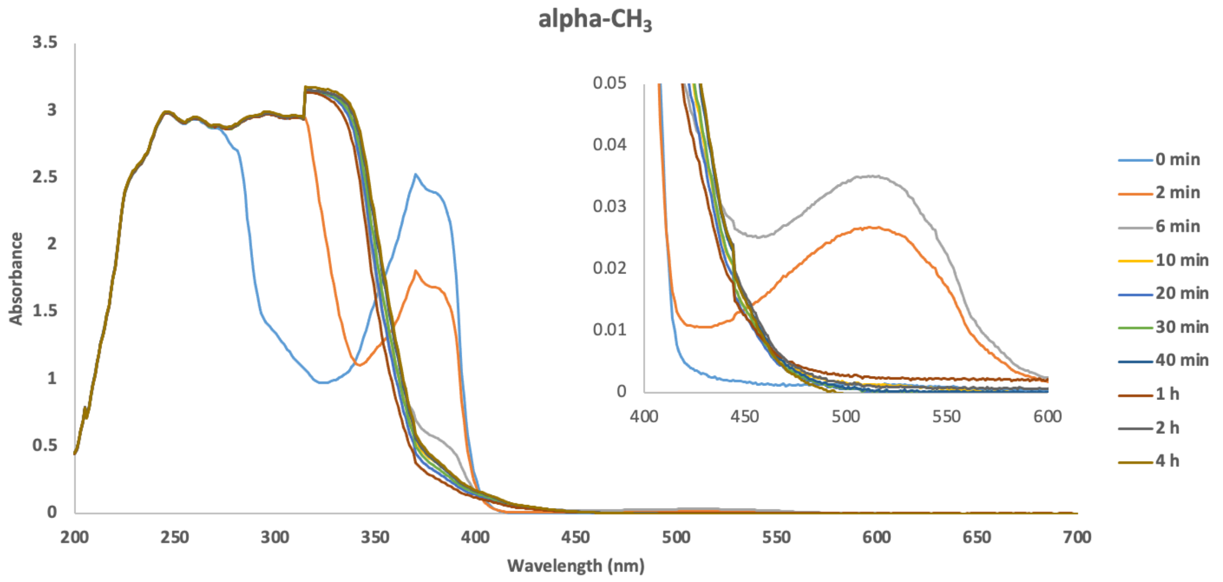

The diazirine was consumed over the first 6 mins, and examination of the spectrum between 400–600 nm (inset) indicated the diazo-isomer as a transient isomerization product.

Fig. S82 UV absorbance spectra of **D5** showing consumption of starting material over time.

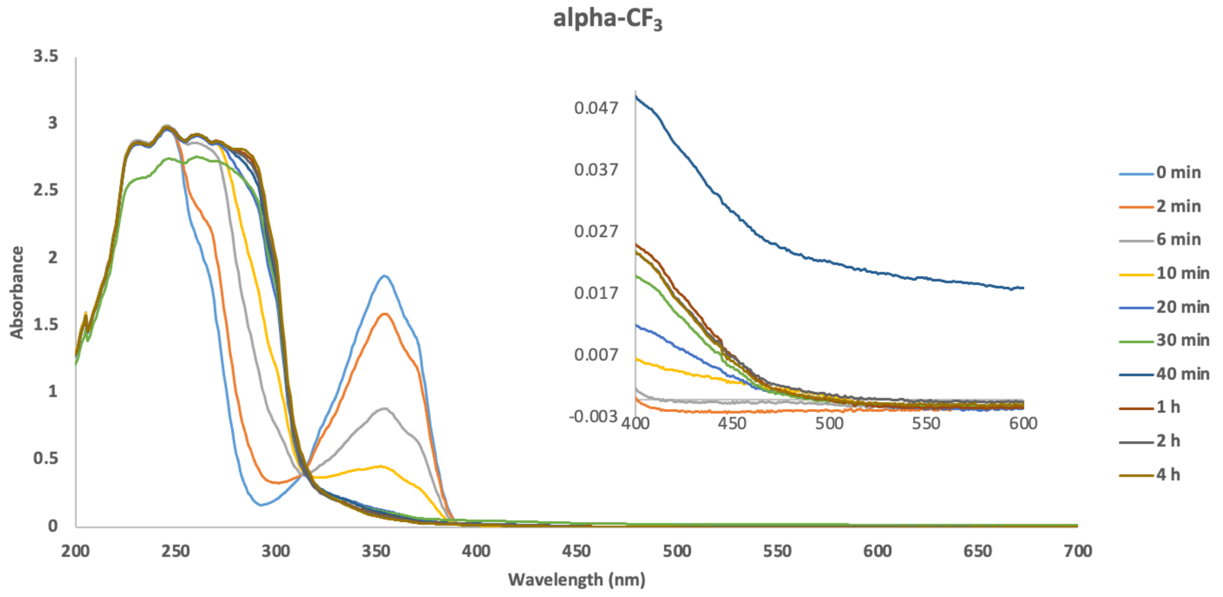

The diazirine was consumed over the first 20 mins, and examination of the spectrum between 400–600 nm (inset) indicated the absence of the diazo-isomer.

Differential Scanning Calorimetry (DSC)

General Protocol for DSC analysis

A sample of the substance to be analyzed (typically 2 to 7 mg) was placed in a Tzero aluminum hermetic pan and sealed by a matching lid. The pan was pierced with a small pinhole to allow evolution of nitrogen gas. The pan was placed in the oven of a DSC25 device (TA instruments) and heated from 40°C to 200°C at a rate of 5°C/min, with an identical empty pan as a reference. The oven was constantly flushed by a 50 mL/min flow of nitrogen. The device recorded the difference in heat flow between the reference and the studied sample, allowing the assignment of T_{onset} and T_{peak}. No significant differences were observed in the T_{onset} and T_{peak} values when the DSC experiment was repeated using stainless steel high-pressure capsules sealed with gold-plated copper sealing disks.

DSC plots of aryl diazirines

DSC analysis for each aryl diazirine substrate was conducted in triplicate. A representative plot for each diazirine is reported below.

Fig. S83 Representative DSC data for **D1**, illustrating the positions of T_{init}, T_{onset} and T_{peak}.

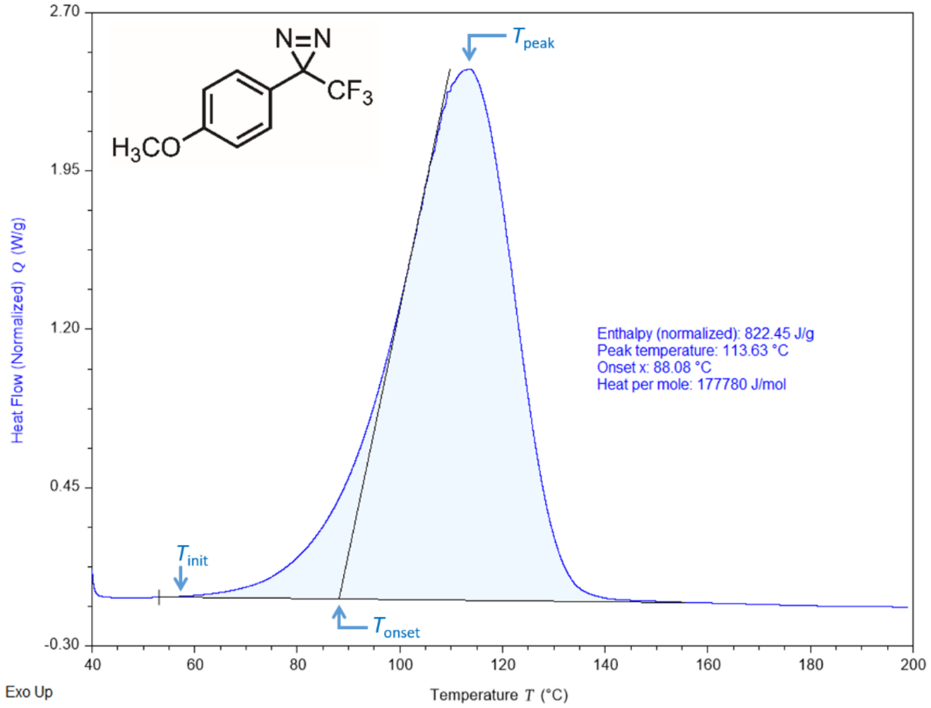

Fig. S84 Representative DSC data for **D2**.

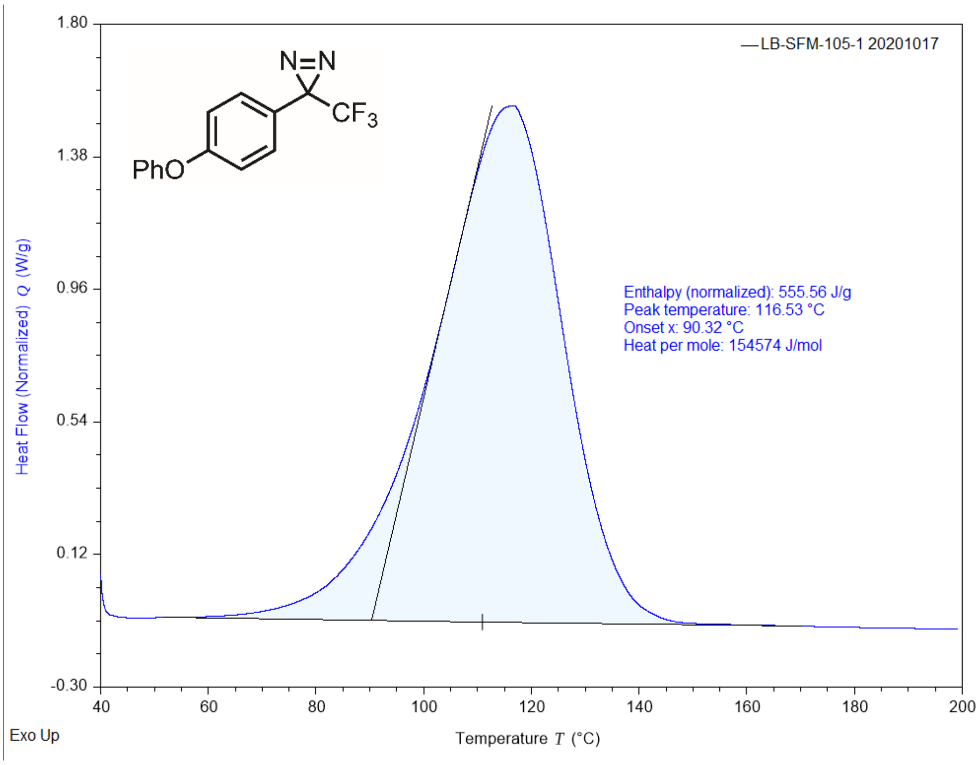

Fig. S85 Representative DSC data for **D3**.

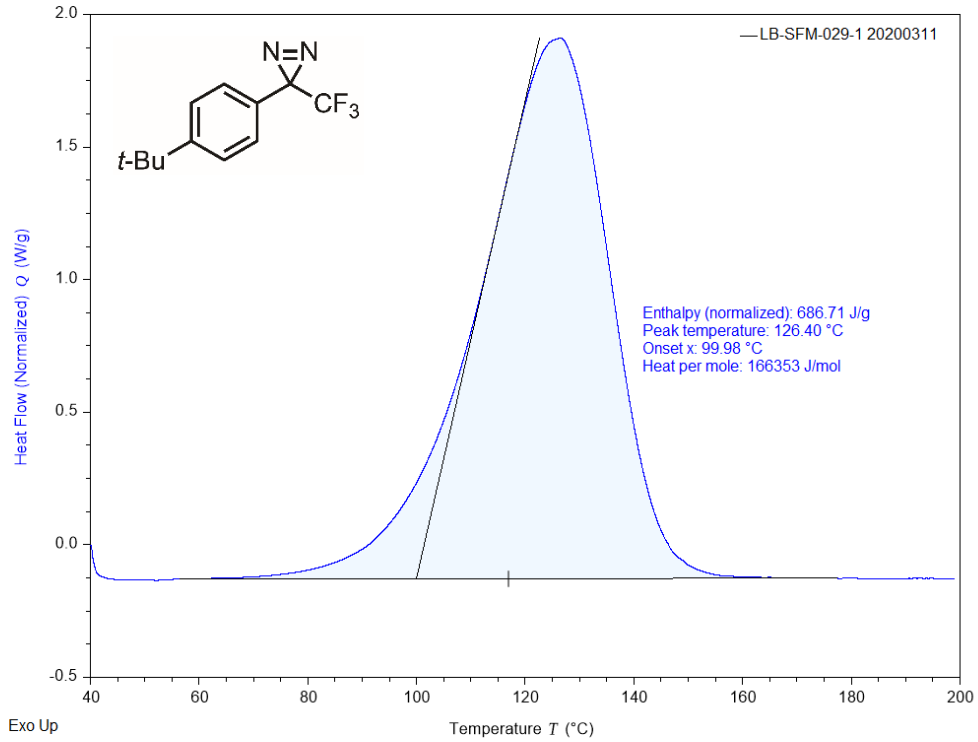

Fig. S86 Representative DSC data for **D4**.

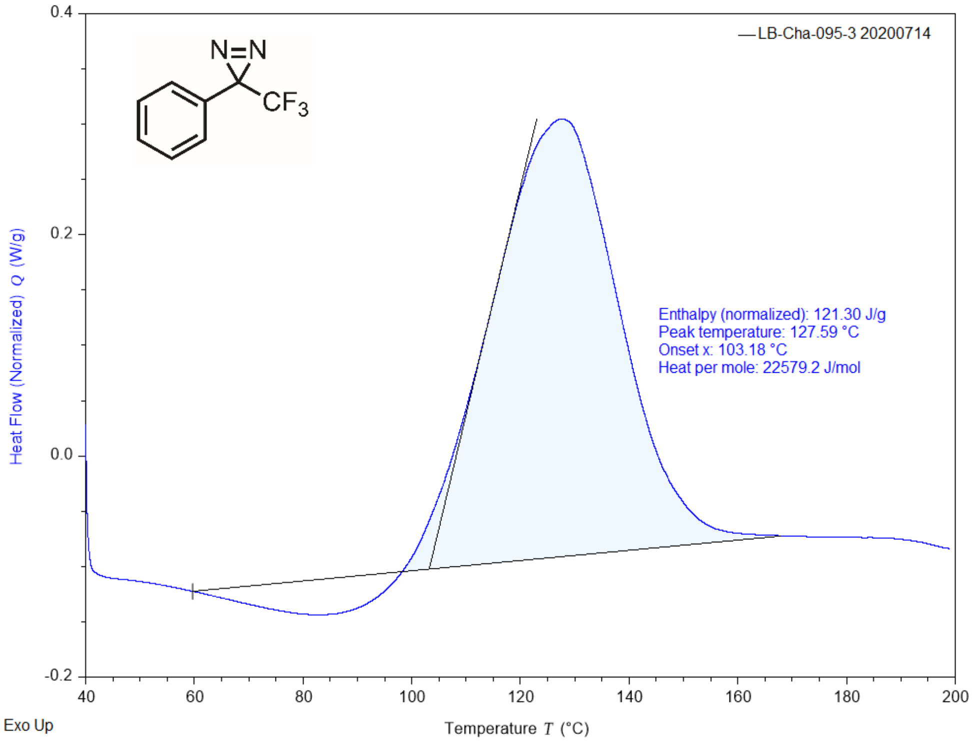

Fig. S87 Representative DSC data for **D5**.

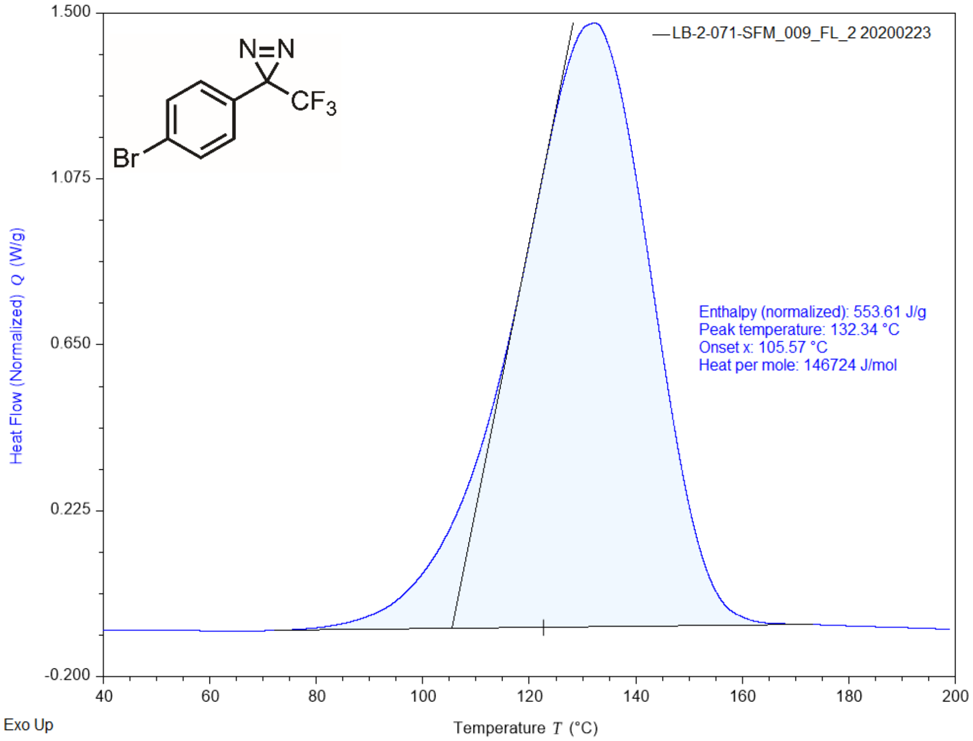

Fig. S88 Representative DSC data for **D6**.

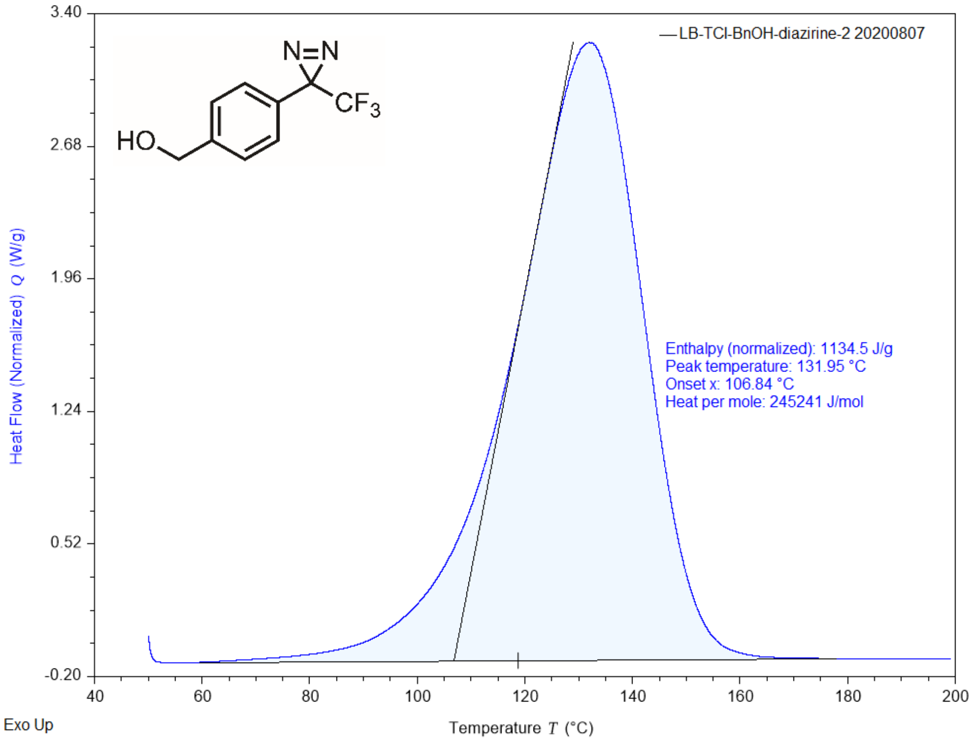

Fig. S89 Representative DSC data for **D7**.

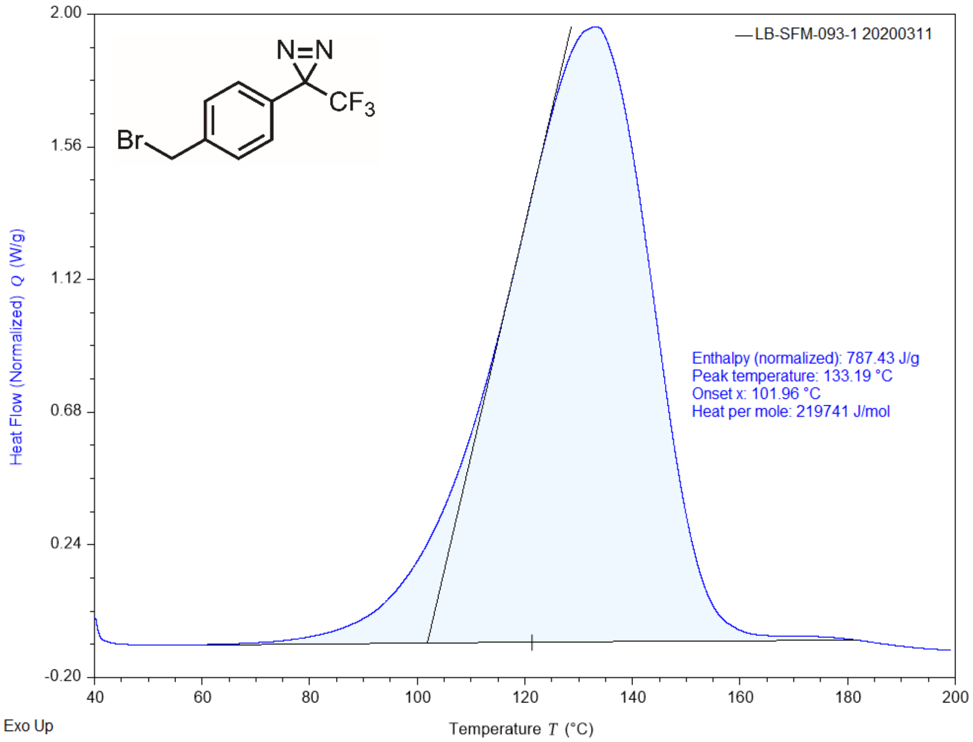

Fig. S90 Representative DSC data for **D8**.

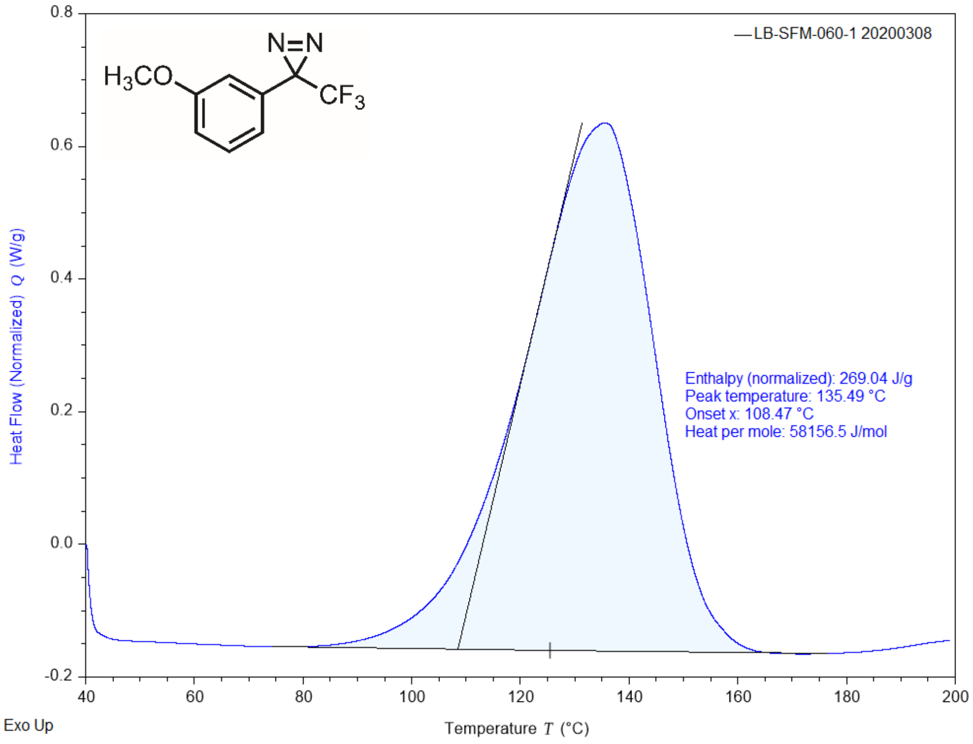

Fig. S91 Representative DSC data for **D9**.

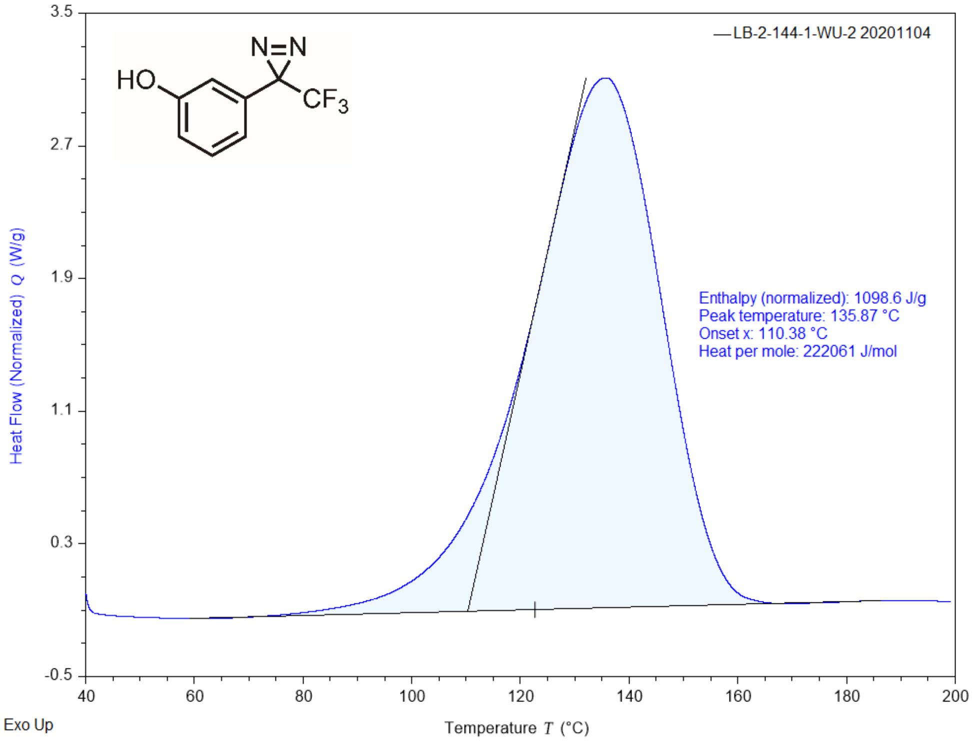

Fig. S92 Representative DSC data for **D10**.

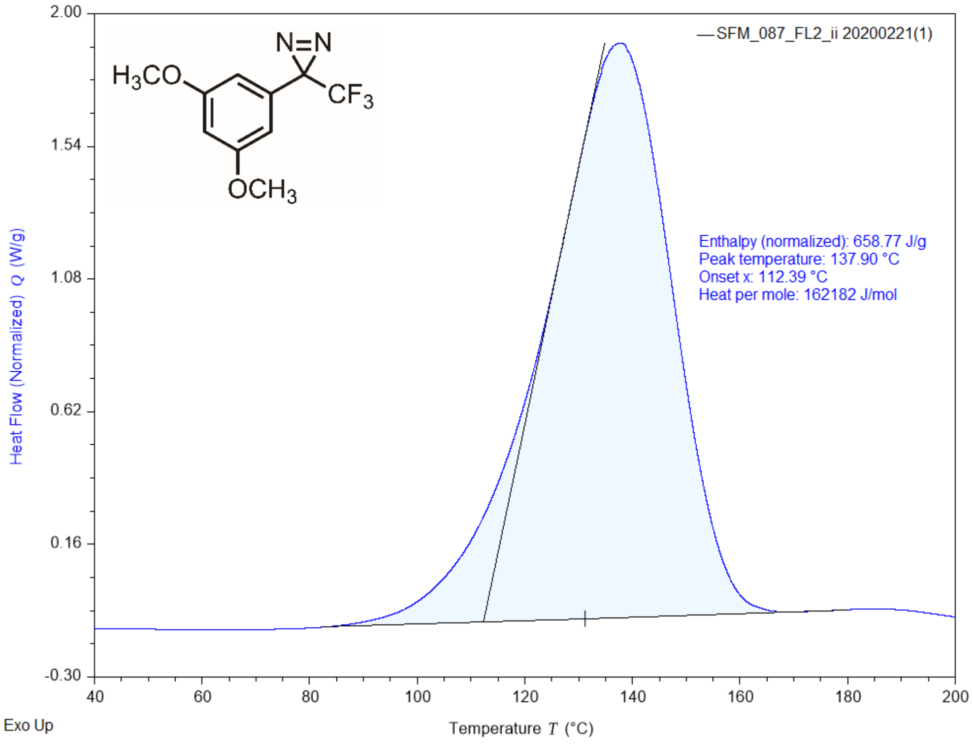

Fig. S93 Representative DSC data for **D11**.

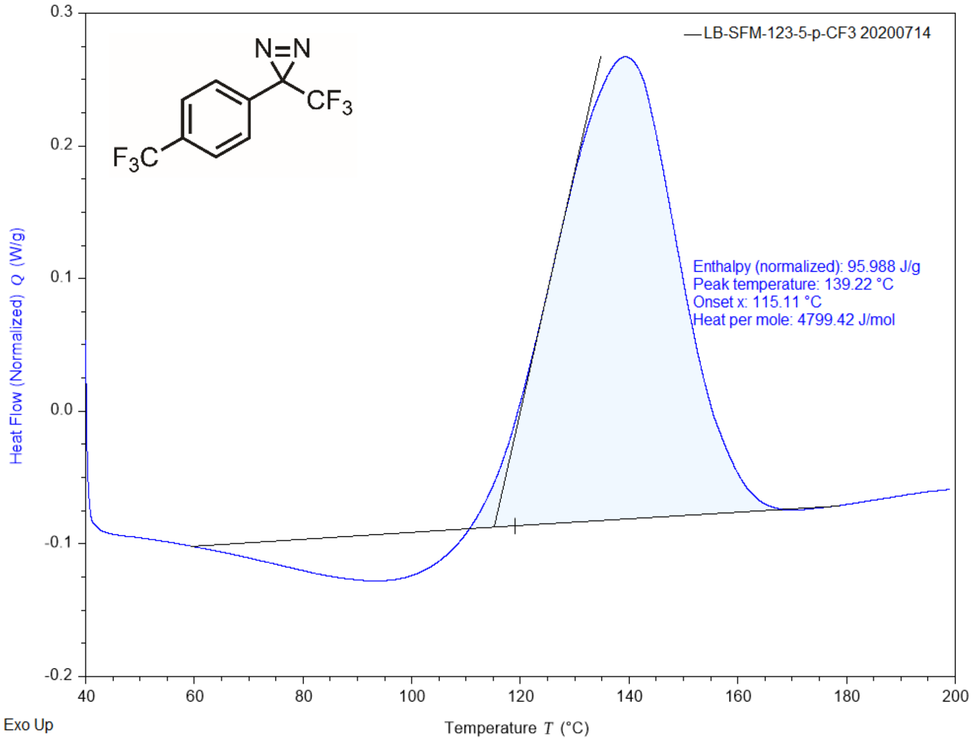

Fig. S94 Representative DSC data for **D12**.

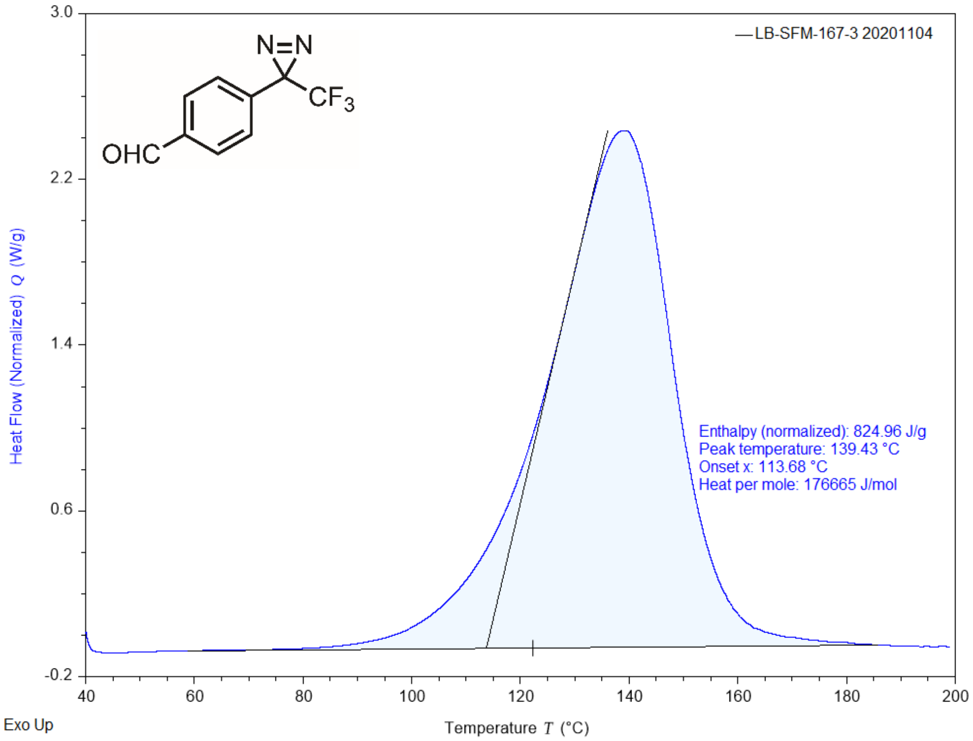

Fig. S95 Representative DSC data for **D13**.

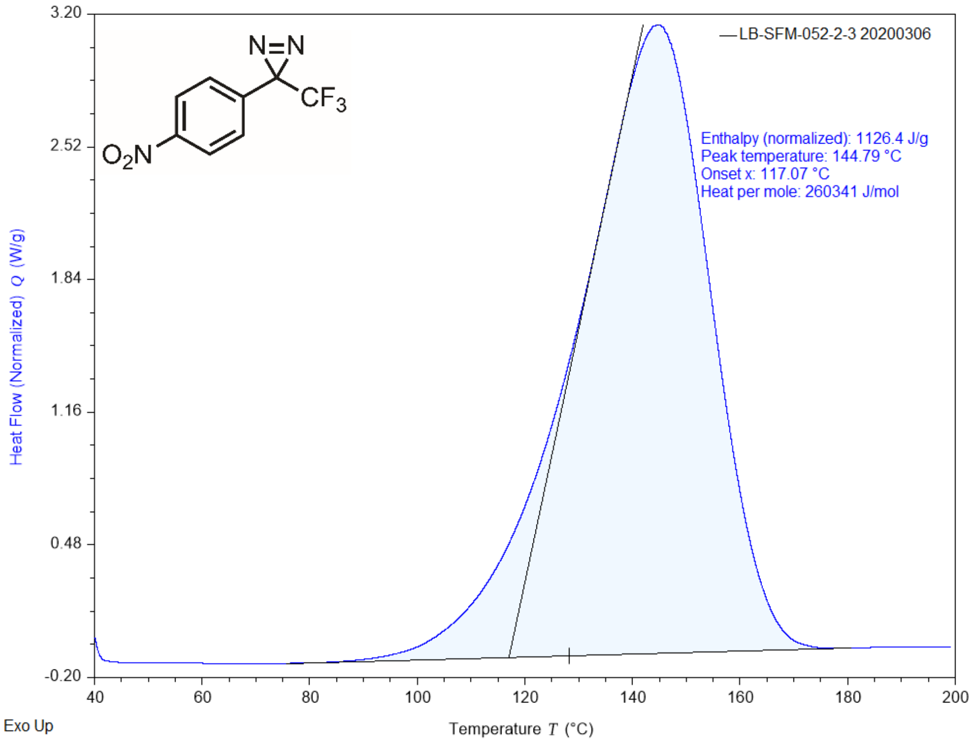

Fig. S96 Representative DSC data for **D14**.

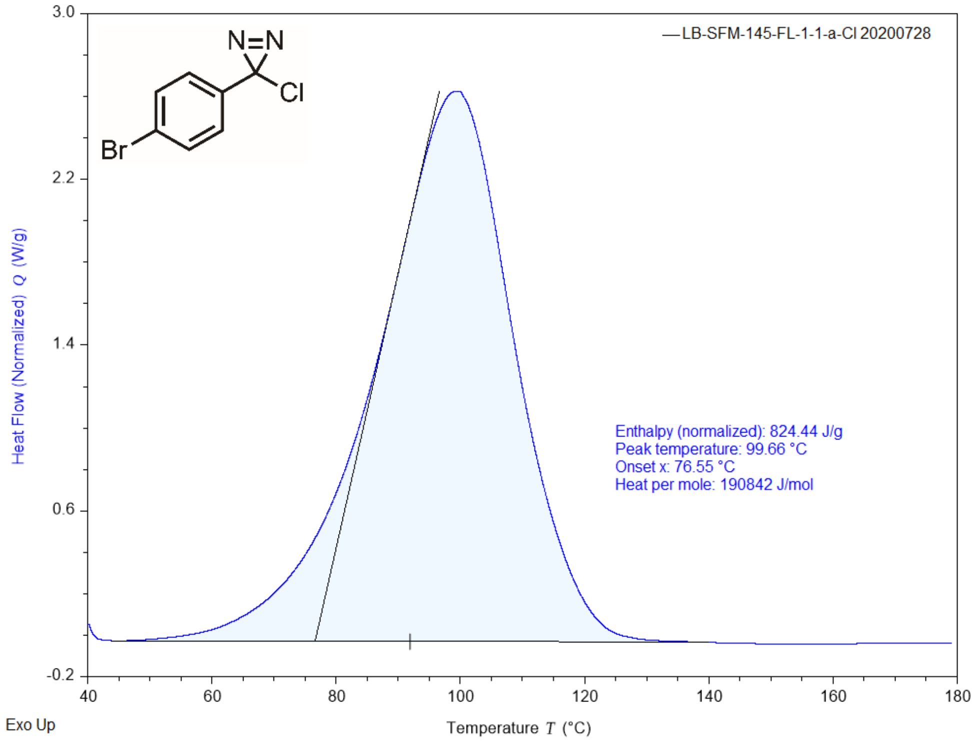

Fig. S97 Representative DSC data for **D15**.

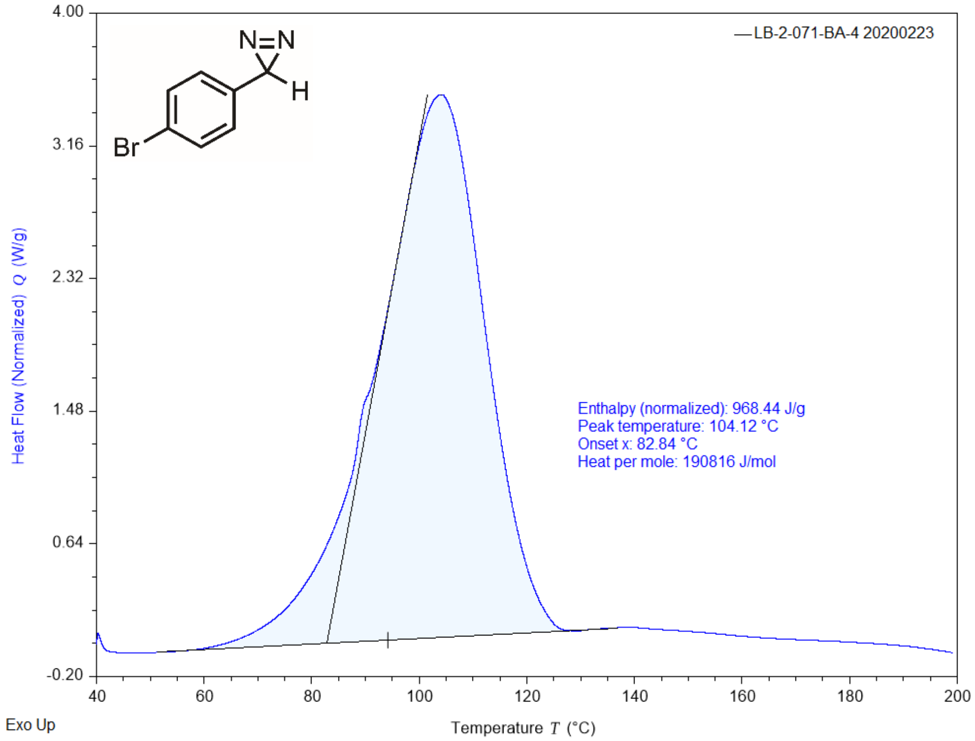

Fig. S98 Representative DSC data for **D16**.

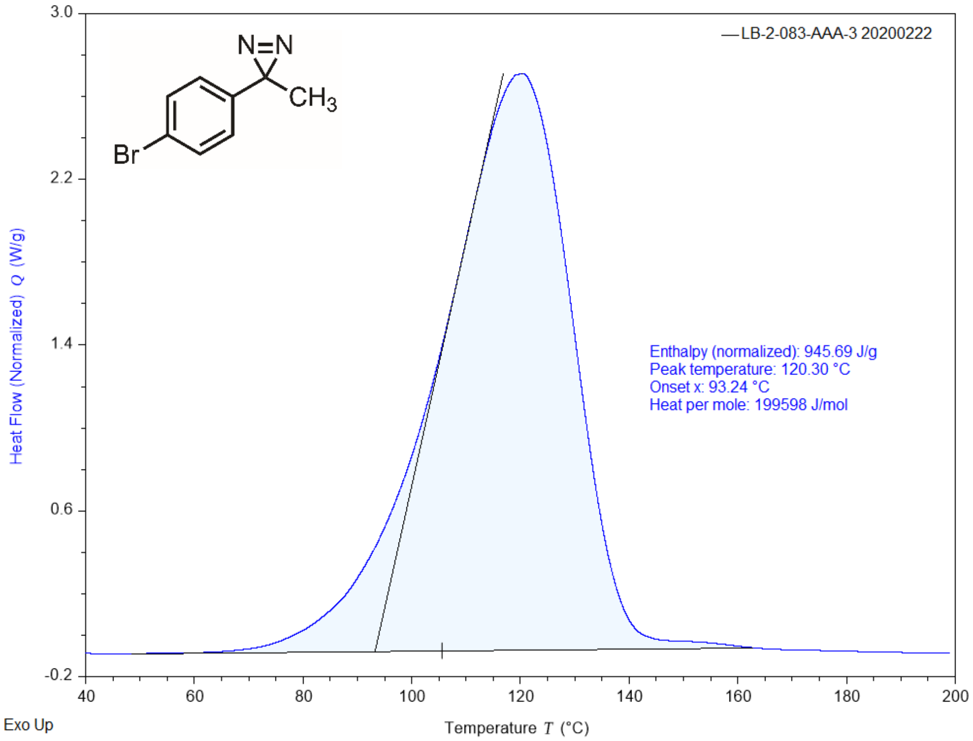

Fig. S99 Representative DSC data for **D17**.

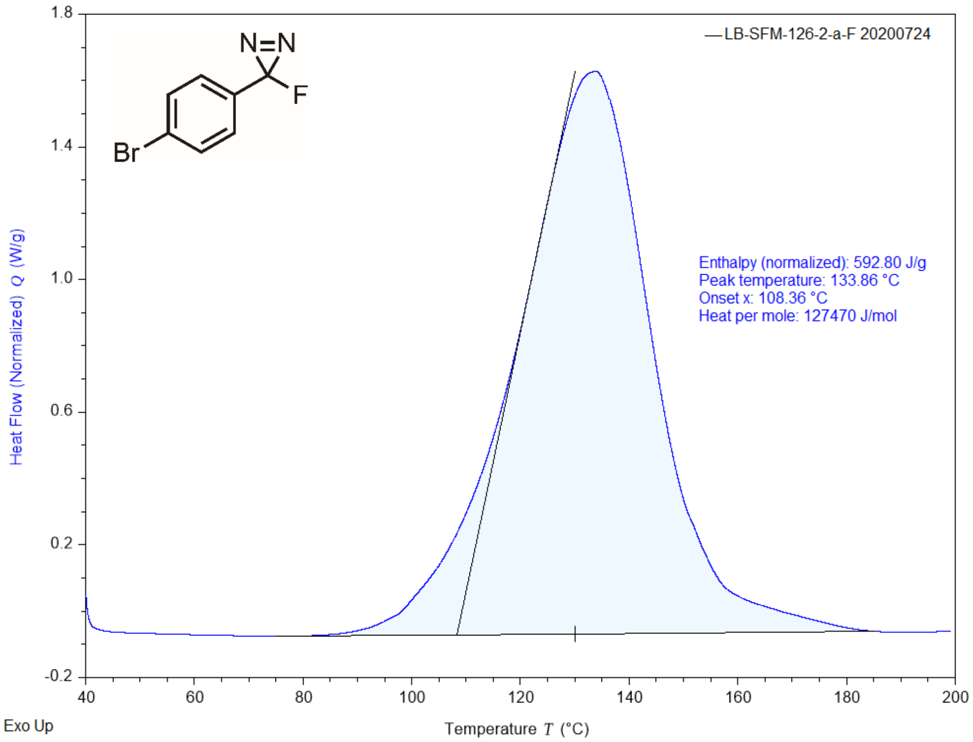

References

1 Y. Zhao and D. G. Truhlar, *Theor. Chem. Acc.*, 2008, **120**, 215–241.
2 S. Grimme, J. Antony, S. Ehrlich and H. Krieg, *J. Chem. Phys.*, 2010, **132**, 154104.
3 Gaussian 16, Revision C.01, M. J. Frisch, G. W. Trucks, H. B. Schlegel, G. E. Scuseria, M. A. Robb, J. R. Cheeseman, G. Scalmani, V. Barone, G. A. Petersson, H. Nakatsuji, X. Li, M. Caricato, A. V. Marenich, J. Bloino, B. G. Janesko, R. Gomperts, B. Mennucci, H. P. Hratchian, J. V. Ortiz, A. F. Izmaylov, J. L. Sonnenberg, D. Williams-Young, F. Ding, F. Lipparini, F. Egidi, J. Goings, B. Peng, A. Petrone, T. Henderson, D. Ranasinghe, V. G. Zakrzewski, J. Gao, N. Rega, G. Zheng, W. Liang, M. Hada, M. Ehara, K. Toyota, R. Fukuda, J. Hasegawa, M. Ishida, T. Nakajima, Y. Honda, O. Kitao, H. Nakai, T. Vreven, K. Throssell, J. A. Montgomery, Jr., J. E. Peralta, F. Ogliaro, M. J. Bearpark, J. J. Heyd, E. N. Brothers, K. N. Kudin, V. N. Staroverov, T. A. Keith, R. Kobayashi, J. Normand, K. Raghavachari, A. P. Rendell, J. C. Burant, S. S. Iyengar, J. Tomasi, M. Cossi, J. M. Millam, M. Klene, C. Adamo, R. Cammi, J. W. Ochterski, R. L. Martin, K. Morokuma, O. Farkas, J. B. Foresman and D. J. Fox, Gaussian, Inc., Wallingford CT, 2016.
4 (*a*) C. Riplinger and F. Neese, *J. Chem. Phys.*, 2013, **138**, 034106; (*b*) C. Riplinger, B. Sandhoefer, A. Hansen and F. Neese, *J. Chem. Phys.*, 2013, **139**, 134101; (*c*) M. Saitow, U. Becker, C. Riplinger, E. F. Valeev and F. Neese, *J. Chem. Phys.*, 2017, **146**, 164105.
5 W. Schneider, S. Lehnhausenm and F. Neese, ORCA Manual Version 4.2.1; 2020; p 72.
6 (*a*) F. Neese, *WIREs Comput. Mol. Sci.*, 2018, **8**, e1327; (*b*) F. Neese, *WIREs Comput. Mol. Sci.*, 2012, **2**, 73–78.
7 V. K. Prasad, Z. Pei, S. Edelmann, A. Otero-de-la-Roza and G. A. DiLabio, BH9, a New Comprehensive Benchmark Dataset for Barrier Heights and Reaction Energies: Assessment of Density Functional Approximations and Basis Set Incompleteness Potentials. https://doi.org/10.33774/chemrxiv-2021-mdjwx.
8 B. Raimer and T. Lindel, *Chem. Eur. J.*, 2013, **19**, 6551–6555.
9 M. B. Johansen, O. R. Gedde, T. S. Mayer and T. Skrydstrup, *Org. Lett.*, 2020, **22**, 4068–4072.
10 X. Gao, R. Cheng, Y.-Y. Xiao, X.-L. Wan and X. Zhang, *Chem*, 2019, **5**, 2987–2999.
11 C.B. Kelly, M. A. Mercadante, E. R. Carnaghan, M. J. Doherty, D.C. Fager, J. J. Hauck, A. E. MacInnis, L. J. Tilley and N. E. Leadbeater, *Eur. J. Org. Chem.*, 2015, 4071–4076.
12 T. Bender, M. Huss, H. Wieczorek, S. Grond and P. von Zezschwitz, *Eur. J. Org. Chem.*, 2007, 3870–3878.
13 A. Blencowe, K. Cosstick and W. Hayes, *New J. Chem.*, 2006, **30**, 53–58.
14 F. Shi, J. K. Shen, D. Chen, K. Fog, K. Thirstrup, M. Hentzer, J.-J. Karlsson, V. Menon, K. A. Jones, K. E. Smith and G. Smith, *ACS Med. Chem. Lett.*, 2011, **2**, 303–306.
15 T. Tomohiro, A. Yamamoto, Y. Tatsumi and Y. Hatanaka, *Chem. Commun.*, 2013, **49**, 11551–11553.
16 L.-H. Zou, D. L. Priebbenow, L. Wang, J. Mottweiler and C. Bolm, *Adv. Synth. Catal.*, 2013, **355**, 2558–2563.
17 W. Wu, Q. Tian, T. Chen and Z. Weng, *Chem. Eur. J.*, 2016, **22**, 16455–16458.
18 H. Nakashima, M. Hashimoto, Y. Sadakane, T. Tomohiro and Y. Hatanaka, *J. Am. Chem. Soc.*, 2006, **128**, 15092–15093.
19 S. Kwiatkowski, P. J. Crocker, A. J. Chavan, I. Nobuyuki, B. E. Haley, D. S. Watt and R. Ho, *Tetrahedron Lett.*, 1990, **31**, 2093–2096.
20 D. Naumann, M. Finke, H. Lange, W. Dukat and W. Tyrra, *J. Fluorine Chem.*, 1992, **56**, 215–237.
21 Y. Hatanaka, M. Hashimoto and Y. Kanaoka, *J. Am. Chem. Soc.*, 1998, **120**, 453–454.
22 J. Terpinski, D. Z. Denney, R. Beveridge, D. P. Cox and R. A. Moss, *Magn. Reson. Chem*., 1987, **25**, 923–927.
23 R. A. Moss, J. Terpinski, D. P. Cox, D. Z. Denny and K. Krogh-Jespersen, *J. Am. Chem. Soc.*, 1985, **107**, 2743–2748.
24 Z. Liu, S. Cao, W. Yu, J. Wu, F. Yi, E. A. Anderson and X. Bi, *Chem*, 2020, **6**, 2110–2124.
25 J. Brunner, H. Senn and F. M. Richards, *J. Biol. Chem.*, 1980, **255**, 3313–3318.
26 M. L. Lepage, C. Simhadri, C. Liu, M. Takaffoli, L. Bi, B. Crawford, A. S. Milani and J. E. Wulff, *Science*, 2019, **366**, 875–878.
