# Supplementary material for: Structure–function relationships in aryl diazirines reveal optimal design features to maximize C–H insertion
Source: Chem Sci. 2021 Aug 10;12(36):12138–48. doi: 10.1039/d1sc03631a (PMC8457397; doi:10.1039/d1sc03631a)
Supplement: SC-012-D1SC03631A-s015 [file SC-012-D1SC03631A-s015.docx]

(1). Unless stated elsewhere, all structures were obtained from M06-2X-D3/6-31G(d,p) in gas phase. For alpha-Cl_4-CH3_triplet-insertion-TS and alpha-F_4-CH3_triplet-insertion-TS, the additional keyword "nosymm" is required to ensure only one imaginary frequency of the transition state.

(2). For coordinates of optimized structures in .xyz files, the format is shown below:

*electronic energy*

*enthalpy*

*Gibbs free energy*

*number of atoms*

*charge multiplicity*

*Cartesian coordinates*
